# Screening of potential antiviral molecules against equid herpesvirus-1 using cellular impedance measurement: Dataset of 2,891 compounds.

**DOI:** 10.1016/j.dib.2020.106492

**Published:** 2020-11-05

**Authors:** Côme Thieulent, Christine Fortier, Hélène Munier-Lehmann, Peggy Suzanne, Patrick Dallemagne, Stephan Zientara, Aymeric Hans, Romain Paillot, Pierre-Olivier Vidalain, Stéphane Pronost, Erika Hue

**Affiliations:** a LABÉO Frank Duncombe, 14280 Saint-Contest, France; b Normandie Univ, UNICAEN, BIOTARGEN EA7450, 14280 Saint-Contest, France; c Normandie Univ, UNICAEN, ImpedanCELL, 14280 Saint-Contest, France; d Institut Pasteur, Unité de Chimie et Biocatalyse, CNRS UMR 3523, 75015 Paris, France; e Normandie Univ, UNICAEN, CERMN, 14000 Caen, France; f Université Paris-Est, Laboratoire de Santé Animale, ANSES, INRA, ENVA, UMR 1161 Virologie, 94700 Maisons-Alfort, France; g ANSES, Laboratoire de pathologie équine de Dozulé, Unité de virologie et parasitologie équine, 14430 Dozulé, France; h CIRI, Centre International de Recherche en Infectiologie, Univ Lyon, Inserm U1111, Université Claude Bernard Lyon 1, CNRS UMR5308, ENS de Lyon, F-69007, Lyon, France; i Equipe Chimie et Biologie, Modélisation et Immunologie pour la Thérapie (CBMIT), Université Paris Descartes, CNRS UMR 8601, 75006 Paris, France

**Keywords:** Real-time cell assay, Chemical library screening, Antiviral, Equid herpesvirus-1

## Abstract

Data presented in this article are associated with the research article “Identification of antiviral compounds against equid herpesvirus-1 using real-time cell assay screening: efficacy of decitabine and valganciclovir alone and in combination” [1]. These data correspond to the *in vitro* screening of 2,891 potential antiviral compounds against equid herpesvirus-1 (EHV-1) based on impedance measurements using the xCELLigence® RTCA MP System. This dataset includes compounds from three different libraries: i) 1,199 compounds from the Prestwick® Chemical Library, which contains mostly US Food and Drug Administration approved drugs (Prestwick® Chemical, Illkirch, France); ii) 1,651 compounds from the Centre d'Etudes et de Recherche sur le Médicament de Normandie (CERMN, Caen, France); iii) 41 compounds (called herein in-house antiviral library) selected for their effects against different human viruses. Compounds effective against EHV-1 were selected using the area under normalised curves (AUC_n_) and the time required for the Cell Index to decrease by 50% after virus infection (CIT_50_). The full dataset from the screen is made publicly available for further analyses.

## Specifications Table

**Table:** 

Subject	Virology, Biology
Specific subject area	Antiviral compounds tested against equid herpesvirus-1
Type of data	Table
	Figure
	Supplementary file (excel)
How data were acquired	The antiviral screening dataset of compounds against equid herpesvirus-1 was acquired using impedance measurements with xCELLigence® RTCA MP system (ACEA Biosciences Inc., San Diego, CA, USA). Data were analysed with RTCA software 2.0 and Microsoft Excel (v. 2010, Microsoft Corporation, Washington, USA).
Data format	Raw data: Microsoft Excel
	Analysed data: Microsoft Excel
	Analysed data: figure
Parameters for data collection	Equine dermal fibroblasts were infected with the EHV-1 Kentucky D (KyD) strain (ATCC® VR700™) at a MOI of 0.01 and incubated at 37 °C and 5% CO_2_ in the presence of tested compounds. This study includes 2,891 compounds from three different libraries: i) 1,199 compounds from the Prestwick® Chemical Library, which contains mostly US Food and Drug Administration approved drugs (Prestwick Chemical, Illkirch, France); ii) 1,651 compounds from the Centre d'Etudes et de Recherche sur le Médicament de Normandie (CERMN, Caen, France); iii) 41 compounds (called herein in-house antiviral library) selected for their effects against different human viruses. The screening by impedancemetry was performed on EHV-1 KyD-infected E. Derm cells as a model and using the RTCA MP system (ACEA Biosciences Inc.) as previously described by Thieulent et al., 2019 [2]. The area under normalised curves (AUC_n_) and the time required for the Cell Index to decrease by 50% after virus infection (CIT_50_) were analysed with RTCA software 2.0 and Microsoft Excel (v. 2010, Microsoft Corporation, Washington, USA).
Description of data collection	All data collected originate from impedance measurements during antiviral assays against EHV-1 using the xCELLigence® RTCA MP system (ACEA Biosciences Inc). According to the manufacturer's recommendations, Cell index (CI) values were first normalised to match the last time point before cell infection using the RTCA software version 2.0 (ACEA Biosciences) prior to data analysis. Finally, four representative values were calculated for each culture conditions: area under normalised curves (AUC_n_; [3]),% AUC compared to control cells, time required for the CI to decrease by 50% after virus infection (CIT_50_; [4]) and Delta CIT_50_. The quality of the screening in 96-well format was assessed using the Z’-factor calculation ([5]) based on AUC_n_ values between 0 and 120 h post-infection (hpi).
Data source location	Institution: LABÉO, Normandie Univ, UNICAEN, BIOTARGEN EA7450, ImpedanCELL
	City/Town/Region: Saint-Contest
	Country: France
	Latitude and longitude (and GPS coordinates, if possible) for collected samples/data: 49.201981, −0.395943
Data accessibility	The dataset is available with this article.
Related research article	C. Thieulent, E. Hue, G. Sutton, C. Fortier, P. Dallemagne, S. Zientara, H. Munier-Lehmann, A. Hans, R. Paillot, P-O. Vidalain, S. Pronost.
	Identification of antiviral compounds against equid herpesvirus-1 using real-time cell assay screening: efficacy of decitabine and valganciclovir alone and in combination.
	Antiviral research.
	https://doi.org/10.1016/j.antiviral.2020.104931.

## Value of the Data

• The dataset describes the antiviral activity of 2,891 compounds against equid herpesvirus-1 (EHV-1) and completes the limited number of active molecules previously described.
• These data would help research of antiviral compounds, especially against herpesviruses.
• These data may influence the development or the repositioning of compounds against herpesviruses.
• This method of medium/high-throughput screening and analysis can be adapted to identify antiviral compounds effective against multiple other viruses.
• The dataset is related to an equid herpesvirus but could be informative to viruses infecting other animal species, including humans, as part of the One-Health concept.

## 1 Data Description

**Fig. 1:**
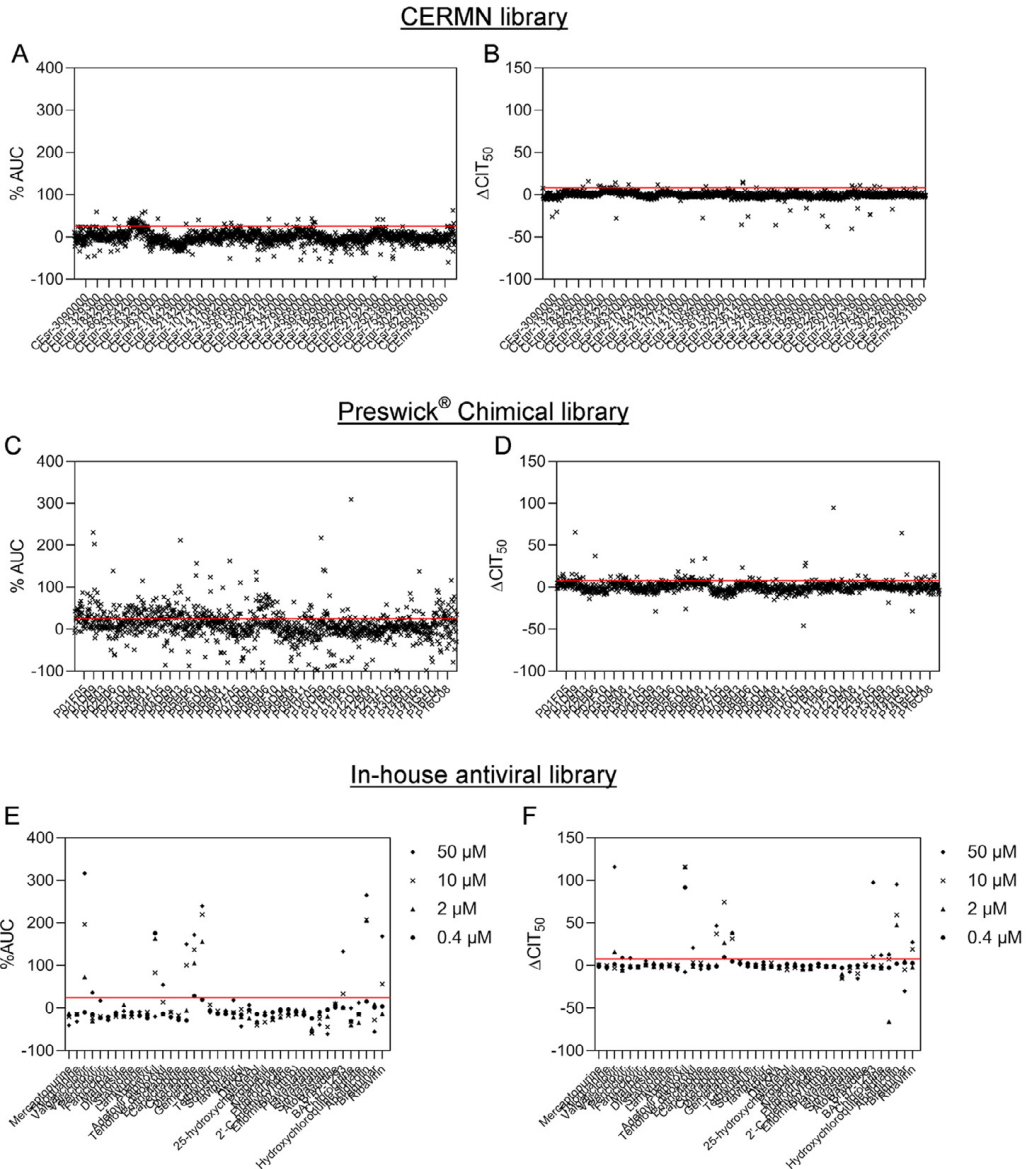
Dataset screening of 2,891 chemical compounds from three libraries to identify EHV-1 inhibitors. The CERMN library (A, B) was screened at 10 µM, Prestwick® Chemical library (C, D) at 10 µg/mL and in-house antiviral library (E, F) at 4 concentrations (0.4, 2, 10 and 50 µM). The percentage of area under normalised Cell Index (CI) from 0 to 96 hpi of treated cells compared to mock-treated cells (%AUC_n_) were reported graphically for each library. The red line represents the cut-off 25 % from which compound could identified as potential antiviral compounds against EHV-1 (A, C, E). The increase of the time required for the CI to decrease by 50 % after virus infection (CIT_50_) of treated cells compared to mock-treated cells (ΔCIT_50_) were reported graphically for each library. The red line represents the cut-off 8 h of delay from which compound could identified as potential antiviral compounds against EHV-1 (B, D, F). The original figure can be found online in Supplementary figure 1 ([1]) at https://doi.org/10.1016/j.antiviral.2020.104931.

Results from the screen with RTCA system are presented in Tables 1, 2 and 3. For each compound, we provide the area under normalised Cell Index curve from 0 to 96 hpi (AUC_n_), the%AUC compared to AUC of untreated, EHV-1-infected control wells, the time required for the cell index to decrease by 50 % after virus infection (CIT_50_) and the ΔCIT_50_ value obtained by comparing results for treated *vs* untreated, EHV-1-infected control wells. The Z’-factor reflects the quality of the screening in 96-well format. Table 1 shows the values obtained with the 1,199 compounds from the Prestwick® Chemical Library, containing mostly US Food and Drug Administration approved drugs (Prestwick Chemical, Illkirch, France). Raw data for the Prestwick® Chemical Library are presented in Supplementary File 1. Table 2 shows the values obtained with the 1,651 compounds from the Centre d'Etudes et de Recherche sur le Médicament de Normandie (CERMN, Caen, France). Raw data for the CERMN Library are presented in Supplementary File 2. Table 3 shows the values obtained with the 41 compounds (called herein in-house antiviral library) selected for their effects against different human viruses. Raw data for the in-house antiviral library are presented in Supplementary File 3. Data shown in Fig. 1 correspond to%AUC and ΔCIT_50_ values for individual compounds from each library.

**Table 1:** Screening of 1,199 compounds from the Prestwick® Chemical Library (Prestwick Chemical, Illkirch, France) at 10 µg/mL. For each compound, the following values are provided: the area under normalised Cell Index curve (AUC_n_), the %AUC_n_ compared to AUC_n_ of untreated infected cells, the time required for the cell index to decrease by 50 % after virus infection (CIT_50_) and the ΔCIT_50_ value obtained by comparing results for treated *vs* untreated, EHV-1-infected control wells. The Z’-factor was used to assess the quality of the screening in 96-well format. For untreated mock-infected and untreated infected cells, the mean ± SD of AUC_n_ is reported. *N.D., not determinable.*

Compound code	Z'-factor	AUC_n_	%AUC_n_	CIT_50_	ΔCIT_50_
P01 untreated mock-infected cells	0.55	224,80 ± 22,76	N.D.	N.D.	N.D.
P01 untreated infected cells	0.55	55,76 ± 2,46	N.D.	46:07:49	N.D.
P01A02	0.55	51.06	−8.41	44:04:18	−2.06
P01B02	0.55	79.62	42.80	51:28:21	5.34
P01C02	0.55	56.79	1.86	45:10:49	−0.95
P01D02	0.55	70.74	26.88	50:33:38	4.43
P01E02	0.55	68.24	22.39	44:54:22	−1.22
P01F02	0.55	70.62	26.66	56:06:02	9.97
P01G02	0.55	80.60	44.56	51:42:43	5.58
P01H02	0.55	73.27	31.41	47:53:08	1.76
P01A03	0.55	83.56	49.87	50:33:38	4.43
P01B03	0.55	73.60	32.00	51:00:11	4.87
P01C03	0.55	81.38	45.95	57:35:17	11.46
P01D03	0.55	64.97	16.52	48:21:33	2.23
P01E03	0.55	65.93	18.26	46:46:10	0.64
P01F03	0.55	53.17	−4.65	47:17:41	1.16
P01G03	0.55	78.27	40.37	51:14:10	5.11
P01H03	0.55	74.58	33.76	50:39:34	4.53
P01A04	0.55	90.10	61.60	49:08:43	3.01
P01B04	0.55	72.59	30.20	50:27:16	4.32
P01C04	0.55	85.75	53.80	59:13:28	13.09
P01D04	0.55	93.30	67.34	61:46:15	15.64
P01E04	0.55	52.51	−5.81	45:45:07	−0.38
P01F04	0.55	78.29	40.42	57:03:22	10.93
P01G04	0.55	77.35	38.73	47:36:32	1.48
P01H04	0.55	85.19	52.79	52:14:28	6.11
P01A05	0.55	104.46	87.35	55:40:41	9.55
P01B05	0.55	66.46	19.21	48:26:47	2.32
P01C05	0.55	60.73	8.93	44:06:15	−2.03
P01D05	0.55	66.69	19.61	48:24:48	2.28
P01E05	0.55	67.40	20.89	47:44:15	1.61
P01F05	0.55	59.57	6.85	47:17:24	1.16
P01G05	0.55	65.27	17.07	51:28:23	5.34
P01H05	0.55	69.43	24.53	53:52:57	7.75
P01A06	0.55	56.35	1.07	46:25:25	0.29
P01B06	0.55	66.45	19.17	48:21:40	2.23
P01C06	0.55	62.35	11.83	48:22:10	2.24
P01D06	0.55	64.67	15.98	49:56:56	3.82
P01E06	0.55	66.40	19.09	49:56:56	3.82
P01F06	0.55	72.34	29.75	49:00:18	2.87
P01G06	0.55	65.86	18.13	47:36:31	1.48
P01H06	0.55	102.80	84.38	53:20:54	7.22
P01A07	0.55	81.72	46.57	49:35:06	3.45
P01B07	0.55	75.27	34.99	52:02:02	5.90
P01C07	0.55	72.60	30.20	49:26:35	3.31
P01D07	0.55	57.34	2.85	47:15:10	1.12
P01E07	0.55	80.16	43.78	49:58:18	3.84
P01F07	0.55	82.86	48.61	53:22:54	7.25
P01G07	0.55	72.35	29.76	51:15:25	5.13
P01H07	0.55	90.93	63.08	61:49:31	15.70
P01A08	0.55	100.32	79.92	51:25:30	5.29
P01B08	0.55	60.05	7.71	45:05:39	−1.04
P01C08	0.55	58.73	5.34	49:15:27	3.13
P01D08	0.55	62.21	11.57	54:09:11	8.02
P01E08	0.55	56.71	1.72	46:50:16	0.71
P01F08	0.55	74.97	34.45	50:08:25	4.01
P01G08	0.55	58.18	4.35	52:42:08	6.57
P01H08	0.55	97.29	74.50	51:42:07	5.57
P01A09	0.55	64.23	15.20	43:05:23	−3.04
P01B09	0.55	184.48	230.88	111:55:07	65.79
P01C09	0.55	56.08	0.58	48:24:42	2.28
P01D09	0.55	67.12	20.38	48:02:20	1.91
P01E09	0.55	71.85	28.87	52:06:29	5.98
P01F09	0.55	169.02	203.15	61:31:24	15.39
P01G09	0.55	107.87	93.48	61:57:29	15.83
P01H09	0.55	44.02	−21.05	53:25:41	7.30
P01A10	0.55	91.80	64.64	54:57:45	8.83
P01B10	0.55	67.73	21.48	43:59:03	−2.15
P01C10	0.55	58.92	5.68	45:04:39	−1.05
P01D10	0.55	50.25	−9.87	46:24:32	0.28
P01E10	0.55	61.00	9.41	44:35:20	−1.54
P01F10	0.55	57.20	2.60	47:36:42	1.48
P01G10	0.55	104.18	86.84	56:39:07	10.52
P01H10	0.55	74.09	32.88	44:18:07	−1.83
P01A11	0.55	92.00	65.01	49:57:59	3.84
P01B11	0.55	86.63	55.37	56:43:26	10.59
P01C11	0.55	67.86	21.71	51:26:20	5.31
P01D11	0.55	66.90	19.99	48:25:35	2.30
P01E11	0.55	62.26	11.66	46:25:09	0.29
P01F11	0.55	56.65	1.61	46:44:49	0.62
P01G11	0.55	60.43	8.37	47:09:36	1.03
P01H11	0.55	82.48	47.93	45:28:30	−0.66
P02 untreated mock-infected cells	0.60	173.87 ± 12.20	N.D.	N.D.	N.D.
P02 untreated infected cells	0.60	52.59 ± 3.87	N.D.	48:34:24	N.D.
P02A02	0.60	51.75	−1.61	40:35:35	−7.69
P02B02	0.60	63.87	21.44	45:55:37	−2.36
P02C02	0.60	84.03	59.77	60:04:32	11.79
P02D02	0.60	67.83	28.97	49:32:20	1.25
P02E02	0.60	53.63	1.96	41:34:55	−6.70
P02F02	0.60	56.17	6.79	44:18:50	−3.97
P02G02	0.60	64.75	23.11	47:24:24	−0.88
P02H02	0.60	54.53	3.69	44:01:16	−4.27
P02A03	0.60	55.67	5.86	49:32:20	1.25
P02B03	0.60	54.76	4.12	42:58:10	−5.32
P02C03	0.60	72.13	37.14	51:59:01	3.70
P02D03	0.60	60.44	14.92	44:57:24	−3.33
P02E03	0.60	74.57	41.79	44:40:49	−3.61
P02F03	0.60	53.45	1.63	45:39:07	−2.63
P02G03	0.60	55.53	5.58	42:55:37	−5.36
P02H03	0.60	54.43	3.48	42:02:18	−6.25
P02A04	0.60	54.65	3.91	45:56:41	−2.34
P02B04	0.60	53.49	1.70	44:28:34	−3.81
P02C04	0.60	64.47	22.58	50:16:02	1.98
P02D04	0.60	67.30	27.96	46:01:14	−2.27
P02E04	0.60	55.27	5.09	44:20:08	−3.95
P02F04	0.60	66.38	26.21	46:55:07	−1.37
P02G04	0.60	61.12	16.21	34:29:20	−13.80
P02H04	0.60	53.38	1.50	44:28:55	−3.80
P02A05	0.60	56.15	6.76	43:03:15	−5.23
P02B05	0.60	43.76	−16.79	42:14:15	−6.05
P02C05	0.60	64.63	22.89	48:21:24	0.07
P02D05	0.60	65.78	25.07	47:14:20	−1.05
P02E05	0.60	41.27	−21.54	40:05:15	−8.20
P02F05	0.60	60.42	14.88	44:34:59	−3.70
P02G05	0.60	60.38	14.81	46:20:48	−1.94
P02H05	0.60	51.98	−1.16	40:27:08	−7.83
P02A06	0.60	73.65	40.03	51:51:41	3.57
P02B06	0.60	26.50	−49.61	N.D.	N.D.
P02C06	0.60	57.12	8.60	46:53:45	−1.39
P02D06	0.60	53.89	2.46	46:54:44	−1.37
P02E06	0.60	54.38	3.40	46:54:44	−1.37
P02F06	0.60	54.56	3.74	41:20:07	−6.95
P02G06	0.60	59.24	12.63	43:54:06	−4.38
P02H06	0.60	40.82	−22.38	N.D.	N.D.
P02A07	0.60	125.87	139.32	85:30:13	37.22
P02B07	0.60	68.78	30.78	46:45:55	−1.52
P02C07	0.60	83.12	58.04	51:21:11	3.07
P02D07	0.60	54.38	3.40	42:16:53	−6.01
P02E07	0.60	20.06	−61.85	N.D.	N.D.
P02F07	0.60	20.94	−60.18	N.D.	N.D.
P02G07	0.60	66.55	26.53	N.D.	N.D.
P02H07	0.60	45.95	−12.64	41:46:54	−6.51
P02A08	0.60	76.50	45.45	45:52:50	−2.41
P02B08	0.60	41.26	−21.56	N.D.	N.D.
P02C08	0.60	54.92	4.42	48:46:42	0.49
P02D08	0.60	48.08	−8.59	41:06:35	−7.18
P02E08	0.60	44.03	−16.28	43:15:18	−5.03
P02F08	0.60	51.67	−1.76	42:07:41	−6.16
P02G08	0.60	68.68	30.59	48:14:55	−0.04
P02H08	0.60	55.40	5.34	43:51:32	−4.43
P02A09	0.60	64.71	23.04	49:48:25	1.52
P02B09	0.60	51.78	−1.55	43:34:58	−4.70
P02C09	0.60	59.51	13.14	46:40:26	−1.61
P02D09	0.60	57.96	10.20	42:31:31	−5.76
P02E09	0.60	82.35	56.57	56:04:34	7.79
P02F09	0.60	55.36	5.26	43:55:01	−4.37
P02G09	0.60	58.35	10.93	47:06:52	−1.17
P02H09	0.60	50.26	−4.45	43:58:40	−4.31
P02A10	0.60	58.73	11.66	43:49:23	−4.46
P02B10	0.60	60.26	14.58	52:25:59	4.15
P02C10	0.60	62.98	19.74	49:02:48	0.76
P02D10	0.60	62.34	18.54	44:10:00	−4.12
P02E10	0.60	44.96	−14.52	39:30:10	−8.78
P02F10	0.60	51.89	−1.35	41:34:17	−6.72
P02G10	0.60	59.70	13.51	46:25:06	−1.87
P02H10	0.60	56.58	7.58	45:09:27	−3.13
P02A11	0.60	61.98	17.84	44:32:31	−3.74
P02B11	0.60	58.32	10.89	N.D.	N.D.
P02C11	0.60	58.03	10.33	48:56:56	0.66
P02D11	0.60	70.76	34.54	40:02:08	−8.25
P02E11	0.60	72.62	38.08	42:33:42	−5.72
P02F11	0.60	49.05	−6.74	41:02:32	−7.24
P02G11	0.60	63.53	20.79	47:43:15	−0.57
P02H11	0.60	55.01	4.60	47:23:07	−0.90
P03 untreated mock-infected cells	0.82	192.72 ± 4.32	N.D.	N.D.	N.D.
P03 untreated infected cells	0.82	56.35 ± 3.75	N.D.	40:23:40	N.D.
P03A02	0.82	71.76	27.34	46:52:04	6.47
P03B02	0.82	84.77	50.43	N.D.	N.D.
P03C02	0.82	65.99	17.10	43:14:34	2.85
P03D02	0.82	33.83	−39.98	N.D.	N.D.
P03E02	0.82	34.23	−39.26	N.D.	N.D.
P03F02	0.82	68.31	21.22	43:19:17	2.93
P03G02	0.82	62.36	10.67	40:36:43	0.22
P03H02	0.82	78.89	40.00	46:01:37	5.63
P03A03	0.82	87.47	55.22	53:05:34	12.70
P03B03	0.82	81.81	45.17	44:48:22	4.41
P03C03	0.82	51.17	−9.20	38:21:52	−2.03
P03D03	0.82	66.57	18.14	44:49:52	4.44
P03E03	0.82	70.67	25.41	43:46:33	3.38
P03F03	0.82	55.86	−0.88	37:52:44	−2.52
P03G03	0.82	86.78	54.00	43:29:15	3.09
P03H03	0.82	84.38	49.74	46:22:54	5.99
P03A04	0.82	53.55	−4.97	47:28:40	7.08
P03B04	0.82	67.88	20.45	44:01:53	3.64
P03C04	0.82	66.75	18.46	45:04:06	4.67
P03D04	0.82	60.86	7.99	40:58:43	0.58
P03E04	0.82	88.74	57.48	45:47:14	5.39
P03F04	0.82	71.55	26.98	44:13:42	3.83
P03G04	0.82	62.03	10.08	39:58:48	−0.41
P03H04	0.82	76.86	36.39	45:51:44	5.47
P03A05	0.82	64.48	14.42	45:29:41	5.10
P03B05	0.82	65.53	16.29	44:39:16	4.26
P03C05	0.82	28.52	−49.39	N.D.	N.D.
P03D05	0.82	49.66	−11.87	36:51:47	−3.53
P03E05	0.82	82.76	46.87	44:25:41	4.03
P03F05	0.82	56.03	−0.57	39:53:23	−0.50
P03G05	0.82	81.04	43.81	42:35:20	2.19
P03H05	0.82	63.31	12.34	38:58:46	−1.42
P03A06	0.82	86.03	52.67	52:49:29	12.43
P03B06	0.82	48.53	−13.88	35:01:43	−5.37
P03C06	0.82	68.11	20.86	44:32:02	4.14
P03D06	0.82	56.49	0.24	41:13:49	0.84
P03E06	0.82	60.36	7.11	41:13:49	0.84
P03F06	0.82	78.57	39.42	45:32:18	5.14
P03G06	0.82	75.84	34.59	45:38:12	5.24
P03H06	0.82	99.48	76.52	54:50:08	14.44
P03A07	0.82	74.76	32.66	47:56:22	7.54
P03B07	0.82	83.36	47.93	46:49:49	6.44
P03C07	0.82	68.92	22.31	42:06:39	1.72
P03D07	0.82	52.02	−7.69	39:18:19	−1.09
P03E07	0.82	61.14	8.50	41:25:10	1.03
P03F07	0.82	63.78	13.18	44:08:56	3.75
P03G07	0.82	52.02	−7.69	41:36:54	1.22
P03H07	0.82	77.95	38.33	48:34:14	8.18
P03A08	0.82	66.96	18.82	45:02:41	4.65
P03B08	0.82	95.55	69.56	50:33:16	10.16
P03C08	0.82	77.89	38.23	45:13:30	4.83
P03D08	0.82	53.18	−5.63	38:38:40	−1.75
P03E08	0.82	121.07	114.85	50:41:49	10.30
P03F08	0.82	63.63	12.92	43:49:59	3.44
P03G08	0.82	88.27	56.64	48:33:48	8.17
P03H08	0.82	91.76	62.83	50:39:05	10.26
P03A09	0.82	83.83	48.75	52:09:11	11.76
P03B09	0.82	82.64	46.64	45:34:57	5.19
P03C09	0.82	60.95	8.16	41:50:14	1.44
P03D09	0.82	55.94	−0.73	37:37:32	−2.77
P03E09	0.82	71.44	26.77	41:10:52	0.79
P03F09	0.82	65.76	16.69	41:57:44	1.57
P03G09	0.82	71.49	26.85	42:27:33	2.06
P03H09	0.82	63.35	12.41	44:54:53	4.52
P03A10	0.82	66.95	18.80	46:22:50	5.99
P03B10	0.82	82.33	46.11	48:06:38	7.72
P03C10	0.82	63.73	13.08	40:48:06	0.41
P03D10	0.82	72.33	28.36	42:28:57	2.09
P03E10	0.82	63.27	12.27	39:18:48	−1.08
P03F10	0.82	64.37	14.23	39:05:55	−1.30
P03G10	0.82	75.08	33.23	41:59:42	1.60
P03H10	0.82	67.81	20.33	43:43:15	3.33
P03A11	0.82	61.19	8.59	45:20:36	4.95
P03B11	0.82	62.04	10.09	39:42:18	−0.69
P03C11	0.82	47.04	−16.53	36:10:19	−4.22
P03D11	0.82	63.05	11.88	42:13:16	1.83
P03E11	0.82	63.89	13.37	42:00:01	1.61
P03F11	0.82	52.47	−6.90	34:17:10	−6.11
P03G11	0.82	63.67	12.98	39:25:22	−0.97
P03H11	0.82	82.02	45.56	46:43:20	6.33
P04 untreated mock-infected cells	0.85	197.50 ± 3.42	N.D.	N.D.	N.D.
P04 untreated infected cells	0.85	47.18 ± 3.94	N.D.	41:50:22	N.D.
P04A02	0.85	72.65	53.98	45:51:22	4.02
P04B02	0.85	55.98	18.66	37:48:05	−4.04
P04C02	0.85	54.79	16.13	39:37:44	−2.21
P04D02	0.85	61.37	30.07	40:57:52	−0.87
P04E02	0.85	67.17	42.37	40:19:34	−1.51
P04F02	0.85	61.90	31.20	38:47:03	−3.06
P04G02	0.85	79.73	69.00	43:10:36	1.34
P04H02	0.85	66.22	40.37	36:59:56	−4.84
P04A03	0.85	58.53	24.06	41:24:04	−0.44
P04B03	0.85	58.36	23.69	39:20:42	−2.49
P04C03	0.85	50.95	7.99	34:31:36	−7.31
P04D03	0.85	40.67	−13.79	54:00:36	12.17
P04E03	0.85	64.96	37.69	41:03:53	−0.77
P04F03	0.85	55.55	17.73	37:10:25	−4.67
P04G03	0.85	57.43	21.74	35:45:03	−6.09
P04H03	0.85	81.86	73.51	46:06:23	4.27
P04A04	0.85	41.50	−12.04	37:41:56	−4.14
P04B04	0.85	52.08	10.39	37:10:40	−4.66
P04C04	0.85	69.17	46.62	42:52:28	1.04
P04D04	0.85	58.11	23.18	40:35:12	−1.25
P04E04	0.85	72.34	53.32	47:20:21	5.50
P04F04	0.85	57.31	21.47	37:06:42	−4.73
P04G04	0.85	56.99	20.80	39:20:27	−2.50
P04H04	0.85	66.79	41.56	44:58:40	3.14
P04A05	0.85	60.17	27.53	39:10:46	−2.66
P04B05	0.85	58.53	24.06	37:19:05	−4.52
P04C05	0.85	46.68	−1.06	37:33:07	−4.29
P04D05	0.85	71.90	52.40	41:07:07	−0.72
P04E05	0.85	67.60	43.28	42:07:06	0.28
P04F05	0.85	60.52	28.29	39:08:18	−2.70
P04G05	0.85	48.17	2.11	35:18:35	−6.53
P04H05	0.85	64.12	35.91	39:57:08	−1.89
P04A06	0.85	61.05	29.41	41:01:08	−0.82
P04B06	0.85	49.68	5.30	37:12:18	−4.63
P04C06	0.85	51.36	8.86	35:22:20	−6.47
P04D06	0.85	60.48	28.20	39:28:04	−2.37
P04E06	0.85	58.53	24.06	39:28:04	−2.37
P04F06	0.85	70.50	49.44	42:43:53	0.89
P04G06	0.85	67.78	43.68	38:55:40	−2.91
P04H06	0.85	83.20	76.36	41:24:26	−0.43
P04A07	0.85	58.40	23.79	36:26:48	−5.39
P04B07	0.85	28.28	−40.05	43:01:02	1.18
P04C07	0.85	57.57	22.02	38:22:44	−3.46
P04D07	0.85	44.83	−4.98	33:21:03	−8.49
P04E07	0.85	6.97	−85.24	N.D.	N.D.
P04F07	0.85	52.11	10.46	37:25:50	−4.41
P04G07	0.85	70.81	50.09	44:56:24	3.10
P04H07	0.85	55.17	16.93	38:00:20	−3.83
P04A08	0.85	31.91	−32.36	N.D.	N.D.
P04B08	0.85	34.14	−27.64	48:19:21	6.48
P04C08	0.85	64.62	36.97	41:41:35	−0.15
P04D08	0.85	74.78	58.51	46:00:49	4.17
P04E08	0.85	79.63	68.79	45:01:24	3.18
P04F08	0.85	58.55	24.11	39:40:05	−2.17
P04G08	0.85	60.82	28.92	39:55:19	−1.92
P04H08	0.85	74.23	57.33	44:02:38	2.20
P04A09	0.85	72.88	54.47	40:43:22	−1.12
P04B09	0.85	61.12	29.54	42:15:24	0.42
P04C09	0.85	60.25	27.71	39:27:12	−2.39
P04D09	0.85	52.48	11.24	36:10:08	−5.67
P04E09	0.85	54.47	15.46	38:33:47	−3.28
P04F09	0.85	45.83	−2.87	35:27:00	−6.39
P04G09	0.85	68.10	44.34	46:09:19	4.32
P04H09	0.85	23.09	−51.05	N.D.	N.D.
P04A10	0.85	60.25	27.71	35:53:46	−5.94
P04B10	0.85	43.27	−8.29	33:10:47	−8.66
P04C10	0.85	65.58	39.00	42:59:24	1.15
P04D10	0.85	68.39	44.97	41:50:09	0.00
P04E10	0.85	51.18	8.48	34:49:08	−7.02
P04F10	0.85	41.40	−12.26	13:03:04	−28.79
P04G10	0.85	61.10	29.51	37:31:29	−4.31
P04H10	0.85	66.20	40.31	37:52:16	−3.97
P04A11	0.85	69.04	46.35	40:49:14	−1.02
P04B11	0.85	45.53	−3.50	34:26:01	−7.41
P04C11	0.85	47.52	0.73	42:53:06	1.05
P04D11	0.85	87.63	85.75	46:51:51	5.02
P04E11	0.85	48.78	3.39	35:54:20	−5.93
P04F11	0.85	51.77	9.73	37:30:33	−4.33
P04G11	0.85	59.04	25.13	38:52:16	−2.97
P04H11	0.85	76.57	62.29	44:05:15	2.25
P05 untreated mock-infected cells	0.68	215.55 ± 11.20	N.D.	N.D.	N.D.
P05 untreated infected cells	0.68	52.16 ± 6.37	N.D.	47:43:17	N.D.
P05A02	0.68	68.32	30.97	51:38:12	3.92
P05B02	0.68	56.50	8.32	52:57:55	5.24
P05C02	0.68	46.66	−10.55	46:50:38	−0.88
P05D02	0.68	54.93	5.30	49:20:40	1.62
P05E02	0.68	53.72	2.99	48:42:54	0.99
P05F02	0.68	58.27	11.71	50:42:54	2.99
P05G02	0.68	68.52	31.35	50:32:44	2.82
P05H02	0.68	105.56	102.36	56:31:05	8.80
P05A03	0.68	70.52	35.19	51:09:05	3.43
P05B03	0.68	73.86	41.59	52:51:35	5.14
P05C03	0.68	55.07	5.56	46:48:27	−0.91
P05D03	0.68	162.81	212.12	55:14:00	7.51
P05E03	0.68	98.43	88.70	46:36:54	−1.11
P05F03	0.68	72.88	39.72	50:13:00	2.50
P05G03	0.68	70.21	34.60	50:42:42	2.99
P05H03	0.68	72.43	38.86	50:25:41	2.71
P05A04	0.68	53.76	3.06	46:58:25	−0.75
P05B04	0.68	68.87	32.02	50:55:35	3.21
P05C04	0.68	62.65	20.11	52:04:12	4.35
P05D04	0.68	57.41	10.05	48:50:32	1.12
P05E04	0.68	40.68	−22.01	N.D.	N.D.
P05F04	0.68	51.89	−0.53	46:39:16	−1.07
P05G04	0.68	79.85	53.07	54:52:55	7.16
P05H04	0.68	73.52	40.95	51:15:56	3.54
P05A05	0.68	63.71	22.14	44:07:41	−3.59
P05B05	0.68	52.69	1.01	47:04:52	−0.64
P05C05	0.68	51.40	−1.46	47:23:04	−0.34
P05D05	0.68	54.80	5.06	50:50:38	3.12
P05E05	0.68	43.92	−15.80	43:41:22	−4.03
P05F05	0.68	55.86	7.09	47:17:34	−0.43
P05G05	0.68	61.63	18.14	53:04:16	5.35
P05H05	0.68	56.36	8.04	50:42:35	2.99
P05A06	0.68	59.69	14.43	50:06:45	2.39
P05B06	0.68	45.10	−13.53	48:40:57	0.96
P05C06	0.68	71.31	36.70	51:29:58	3.78
P05D06	0.68	89.55	71.67	51:13:33	3.50
P05E06	0.68	24.36	−53.30	N.D.	N.D.
P05F06	0.68	49.20	−5.69	50:52:28	3.15
P05G06	0.68	6.45	−87.64	N.D.	N.D.
P05H06	0.68	64.90	24.41	48:24:47	0.69
P05A07	0.68	67.19	28.80	51:21:57	3.64
P05B07	0.68	58.18	11.54	47:29:31	−0.23
P05C07	0.68	45.34	−13.07	46:33:14	−1.17
P05D07	0.68	57.27	9.79	51:35:40	3.87
P05E07	0.68	57.19	9.65	50:23:01	2.66
P05F07	0.68	59.93	14.89	51:38:59	3.93
P05G07	0.68	44.35	−14.97	N.D.	N.D.
P05H07	0.68	47.89	−8.19	41:08:13	−6.58
P05A08	0.68	58.29	11.74	46:58:37	−0.74
P05B08	0.68	55.35	6.10	48:23:27	0.67
P05C08	0.68	43.25	−17.08	49:26:06	1.71
P05D08	0.68	54.34	4.17	51:19:22	3.60
P05E08	0.68	49.04	−6.00	50:21:05	2.63
P05F08	0.68	63.72	22.16	53:47:48	6.08
P05G08	0.68	80.68	54.66	60:05:45	12.37
P05H08	0.68	52.80	1.22	N.D.	N.D.
P05A09	0.68	61.16	17.24	48:53:04	1.16
P05B09	0.68	75.90	45.50	48:38:40	0.92
P05C09	0.68	70.97	36.05	58:39:04	10.93
P05D09	0.68	66.99	28.42	52:32:29	4.82
P05E09	0.68	119.03	128.19	66:20:29	18.62
P05F09	0.68	47.40	−9.14	43:54:30	−3.81
P05G09	0.68	133.70	156.31	56:32:25	8.82
P05H09	0.68	58.62	12.39	46:24:37	−1.31
P05A10	0.68	55.78	6.93	46:10:57	−1.54
P05B10	0.68	67.02	28.48	51:06:03	3.38
P05C10	0.68	80.84	54.98	55:57:13	8.23
P05D10	0.68	53.06	1.73	50:21:55	2.64
P05E10	0.68	59.48	14.03	48:03:25	0.34
P05F10	0.68	55.96	7.28	48:13:33	0.50
P05G10	0.68	57.37	9.98	48:08:18	0.42
P05H10	0.68	72.34	38.68	58:16:44	10.56
P05A11	0.68	53.13	1.86	47:39:57	−0.06
P05B11	0.68	65.81	26.17	55:53:24	8.17
P05C11	0.68	56.79	8.88	51:08:45	3.42
P05D11	0.68	56.77	8.82	52:19:17	4.60
P05E11	0.68	44.19	−15.29	49:33:52	1.84
P05F11	0.68	70.24	34.65	54:52:24	7.15
P05G11	0.68	60.71	16.38	50:21:13	2.63
P05H11	0.68	61.53	17.96	51:09:37	3.44
P06 untreated mock-infected cells	0.71	182.91 ± 10.37	N.D.	N.D.	N.D.
P06 untreated infected cells	0.71	42.31 ± 3.33	N.D.	39:58:52	N.D.
P06A02	0.71	49.11	16.07	43:45:03	3.77
P06B02	0.71	51.26	21.15	43:18:17	3.32
P06C02	0.71	63.71	50.59	53:25:04	13.44
P06D02	0.71	57.68	36.34	46:10:47	6.20
P06E02	0.71	38.01	−10.16	14:20:30	−25.64
P06F02	0.71	55.53	31.26	47:00:29	7.03
P06G02	0.71	59.30	40.17	47:39:03	7.67
P06H02	0.71	47.40	12.04	38:03:37	−1.92
P06A03	0.71	58.68	38.69	44:50:06	4.85
P06B03	0.71	14.24	−66.34	N.D.	N.D.
P06C03	0.71	52.77	24.73	44:56:50	4.97
P06D03	0.71	53.94	27.51	38:44:01	−1.25
P06E03	0.71	60.88	43.89	49:49:34	9.84
P06F03	0.71	54.23	28.19	47:52:04	7.89
P06G03	0.71	30.65	−27.56	N.D.	N.D.
P06H03	0.71	51.52	21.78	45:43:41	5.75
P06A04	0.71	48.53	14.72	41:44:27	1.76
P06B04	0.71	46.61	10.18	42:18:14	2.32
P06C04	0.71	47.01	11.11	44:34:57	4.60
P06D04	0.71	55.35	30.83	49:43:13	9.74
P06E04	0.71	48.13	13.77	47:21:51	7.38
P06F04	0.71	46.29	9.42	43:17:27	3.31
P06G04	0.71	60.09	42.04	51:46:59	11.80
P06H04	0.71	48.99	15.81	44:23:59	4.42
P06A05	0.71	54.96	29.90	47:21:32	7.38
P06B05	0.71	94.73	123.92	71:33:12	31.57
P06C05	0.71	80.54	90.38	51:58:37	12.00
P06D05	0.71	45.20	6.84	42:26:02	2.45
P06E05	0.71	51.18	20.98	45:59:27	6.01
P06F05	0.71	58.14	37.42	46:55:54	6.95
P06G05	0.71	49.01	15.85	42:57:44	2.98
P06H05	0.71	50.90	20.32	46:08:11	6.16
P06A06	0.71	38.22	−9.66	39:27:16	−0.53
P06B06	0.71	42.19	−0.27	43:44:15	3.76
P06C06	0.71	43.62	3.10	41:46:27	1.79
P06D06	0.71	43.59	3.02	40:53:52	0.92
P06E06	0.71	54.47	28.76	40:53:52	0.92
P06F06	0.71	50.40	19.12	46:45:58	6.79
P06G06	0.71	47.36	11.95	48:42:25	8.73
P06H06	0.71	44.68	5.60	43:31:37	3.55
P06A07	0.71	50.35	19.02	46:30:44	6.53
P06B07	0.71	48.21	13.95	43:54:37	3.93
P06C07	0.71	49.38	16.71	46:00:01	6.02
P06D07	0.71	31.01	−26.70	N.D.	N.D.
P06E07	0.71	52.44	23.95	47:19:44	7.35
P06F07	0.71	53.65	26.80	44:24:38	4.43
P06G07	0.71	43.48	2.78	46:30:01	6.52
P06H07	0.71	29.32	−30.70	N.D.	N.D.
P06A08	0.71	47.89	13.19	44:14:44	4.26
P06B08	0.71	49.88	17.90	46:13:16	6.24
P06C08	0.71	41.64	−1.57	46:15:28	6.28
P06D08	0.71	48.12	13.75	36:55:24	−3.06
P06E08	0.71	61.02	44.24	39:51:05	−0.13
P06F08	0.71	57.34	35.52	50:26:56	10.47
P06G08	0.71	50.18	18.61	46:50:11	6.86
P06H08	0.71	58.12	37.38	46:59:46	7.02
P06A09	0.71	51.18	20.98	44:16:48	4.30
P06B09	0.71	41.99	−0.74	43:00:17	3.02
P06C09	0.71	19.33	−54.31	N.D.	N.D.
P06D09	0.71	18.13	−57.14	N.D.	N.D.
P06E09	0.71	53.08	25.48	42:26:04	2.45
P06F09	0.71	47.10	11.33	42:34:19	2.59
P06G09	0.71	49.85	17.82	46:11:19	6.21
P06H09	0.71	39.36	−6.98	43:33:36	3.58
P06A10	0.71	84.39	99.48	74:23:49	34.42
P06B10	0.71	51.83	22.50	44:18:10	4.32
P06C10	0.71	43.00	1.64	43:08:58	3.17
P06D10	0.71	49.64	17.34	47:27:47	7.48
P06E10	0.71	55.90	32.14	47:38:38	7.66
P06F10	0.71	18.43	−56.44	N.D.	N.D.
P06G10	0.71	48.55	14.76	44:24:41	4.43
P06H10	0.71	48.44	14.49	42:46:27	2.79
P06A11	0.71	45.25	6.96	43:00:13	3.02
P06B11	0.71	44.74	5.74	46:24:21	6.42
P06C11	0.71	53.55	26.59	49:18:44	9.33
P06D11	0.71	53.89	27.38	46:41:37	6.71
P06E11	0.71	52.38	23.81	46:29:19	6.51
P06F11	0.71	56.55	33.67	47:53:11	7.91
P06G11	0.71	41.65	−1.55	41:59:45	2.01
P06H11	0.71	8.63	−79.61	N.D.	N.D.
P07 untreated mock-infected cells	0.54	191.67 ± 5.99	N.D.	N.D.	N.D.
P07 untreated infected cells	0.54	69.77 ± 12.53	N.D.	52:37:50	N.D.
P07A02	0.54	55.35	−20.68	51:52:58	−0.75
P07B02	0.54	63.63	−8.81	51:58:26	−0.66
P07C02	0.54	70.07	0.43	50:08:19	−2.49
P07D02	0.54	107.71	54.36	56:28:23	3.84
P07E02	0.54	81.09	16.22	45:03:18	−7.58
P07F02	0.54	81.39	16.64	44:57:36	−7.67
P07G02	0.54	183.19	162.54	65:13:08	12.59
P07H02	0.54	73.48	5.31	49:42:58	−2.91
P07A03	0.54	62.11	−10.98	53:34:47	0.95
P07B03	0.54	99.13	42.07	46:48:12	−5.83
P07C03	0.54	83.09	19.08	45:24:08	−7.23
P07D03	0.54	72.73	4.24	51:45:19	−0.88
P07E03	0.54	79.68	14.19	43:51:48	−8.77
P07F03	0.54	74.09	6.18	51:49:41	−0.80
P07G03	0.54	67.21	−3.67	44:37:00	−8.01
P07H03	0.54	65.85	−5.63	43:50:33	−8.79
P07A04	0.54	61.80	−11.43	43:58:56	−8.65
P07B04	0.54	59.24	−15.10	47:22:04	−5.26
P07C04	0.54	57.85	−17.09	44:44:31	−7.89
P07D04	0.54	78.41	12.38	51:31:39	−1.10
P07E04	0.54	66.97	−4.02	46:19:06	−6.31
P07F04	0.54	58.73	−15.83	42:16:54	−10.35
P07G04	0.54	85.80	22.97	45:30:54	−7.12
P07H04	0.54	91.37	30.95	44:42:13	−7.93
P07A05	0.54	72.74	4.25	46:25:17	−6.21
P07B05	0.54	58.86	−15.65	47:43:05	−4.91
P07C05	0.54	69.20	−0.82	45:31:46	−7.10
P07D05	0.54	64.68	−7.30	52:00:11	−0.63
P07E05	0.54	48.76	−30.12	49:51:13	−2.78
P07F05	0.54	53.40	−23.47	42:11:58	−10.43
P07G05	0.54	87.25	25.04	47:52:43	−4.75
P07H05	0.54	76.63	9.82	46:16:36	−6.35
P07A06	0.54	75.90	8.78	53:36:46	0.98
P07B06	0.54	88.66	27.06	59:04:02	6.44
P07C06	0.54	95.48	36.83	60:54:00	8.27
P07D06	0.54	61.75	−11.50	49:14:44	−3.39
P07E06	0.54	60.82	−12.84	49:14:44	−3.39
P07F06	0.54	76.04	8.98	48:51:32	−3.77
P07G06	0.54	77.19	10.64	49:19:17	−3.31
P07H06	0.54	147.26	111.05	47:35:28	−5.04
P07A07	0.54	95.61	37.03	49:59:45	−2.63
P07B07	0.54	59.19	−15.17	42:45:14	−9.88
P07C07	0.54	57.82	−17.13	45:54:24	−6.72
P07D07	0.54	16.78	−75.94	N.D.	N.D.
P07E07	0.54	72.21	3.49	49:03:54	−3.57
P07F07	0.54	68.99	−1.12	48:37:02	−4.01
P07G07	0.54	55.73	−20.13	40:14:57	−12.38
P07H07	0.54	21.15	−69.69	N.D.	N.D.
P07A08	0.54	23.23	−66.71	N.D.	N.D.
P07B08	0.54	68.36	−2.03	43:55:41	−8.70
P07C08	0.54	38.00	−45.54	39:23:08	−13.25
P07D08	0.54	59.11	−15.28	42:26:48	−10.18
P07E08	0.54	67.28	−3.58	48:53:59	−3.73
P07F08	0.54	66.44	−4.78	45:13:17	−7.41
P07G08	0.54	68.72	−1.51	46:29:14	−6.14
P07H08	0.54	62.98	−9.74	46:16:45	−6.35
P07A09	0.54	78.27	12.17	50:53:44	−1.74
P07B09	0.54	65.70	−5.85	44:34:09	−8.06
P07C09	0.54	63.48	−9.01	48:56:57	−3.68
P07D09	0.54	120.95	73.34	53:31:34	0.90
P07E09	0.54	59.37	−14.91	42:12:48	−10.42
P07F09	0.54	75.92	8.81	46:29:49	−6.13
P07G09	0.54	71.68	2.73	50:11:53	−2.43
P07H09	0.54	78.02	11.82	45:09:03	−7.48
P07A10	0.54	33.49	−52.01	N.D.	N.D.
P07B10	0.54	83.72	19.99	52:40:18	0.04
P07C10	0.54	59.43	−14.83	47:09:56	−5.47
P07D10	0.54	69.63	−0.21	49:18:10	−3.33
P07E10	0.54	74.78	7.18	46:38:29	−5.99
P07F10	0.54	74.52	6.80	45:07:57	−7.50
P07G10	0.54	66.58	−4.58	41:20:03	−11.30
P07H10	0.54	99.09	42.01	48:26:05	−4.20
P07A11	0.54	65.71	−5.82	50:50:04	−1.80
P07B11	0.54	74.74	7.12	49:53:25	−2.74
P07C11	0.54	61.29	−12.16	48:02:16	−4.59
P07D11	0.54	66.71	−4.39	44:26:40	−8.19
P07E11	0.54	73.24	4.97	46:13:06	−6.41
P07F11	0.54	68.54	−1.77	43:34:31	−9.06
P07G11	0.54	0.52	−99.26	N.D.	N.D.
P07H11	0.54	86.23	23.59	46:42:36	−5.92
P08 untreated mock-infected cells	0.58	234.11 ± 18.80	N.D.	N.D.	N.D.
P08 untreated infected cells	0.58	60.66 ± 5.19	N.D.	43:40:48	N.D.
P08A02	0.58	66.36	9.39	42:27:39	−1.22
P08B02	0.58	82.87	36.62	49:28:11	5.79
P08C02	0.58	74.12	22.19	44:26:43	0.77
P08D02	0.58	77.71	28.12	46:14:25	2.56
P08E02	0.58	33.63	−44.55	N.D.	N.D.
P08F02	0.58	46.34	−23.61	45:02:31	1.36
P08G02	0.58	71.54	17.93	46:28:49	2.80
P08H02	0.58	66.96	10.38	43:05:25	−0.59
P08A03	0.58	102.84	69.54	50:38:36	6.96
P08B03	0.58	143.03	135.80	41:09:40	−2.52
P08C03	0.58	132.33	118.16	46:02:12	2.36
P08D03	0.58	68.05	12.19	44:46:07	1.09
P08E03	0.58	77.45	27.68	49:43:17	6.04
P08F03	0.58	51.85	−14.53	45:47:55	2.12
P08G03	0.58	57.97	−4.44	38:56:27	−4.74
P08H03	0.58	69.85	15.15	41:09:37	−2.52
P08A04	0.58	89.58	47.68	44:51:34	1.18
P08B04	0.58	109.52	80.55	45:15:12	1.57
P08C04	0.58	101.25	66.93	39:46:21	−3.91
P08D04	0.58	90.81	49.71	49:51:18	6.17
P08E04	0.58	71.35	17.63	47:19:29	3.64
P08F04	0.58	68.35	12.68	66:56:20	23.26
P08G04	0.58	64.19	5.83	40:36:12	−3.08
P08H04	0.58	90.63	49.41	48:12:59	4.54
P08A05	0.58	101.31	67.01	48:23:24	4.71
P08B05	0.58	89.45	47.46	50:07:05	6.44
P08C05	0.58	93.64	54.37	50:04:00	6.39
P08D05	0.58	65.62	8.19	43:17:26	−0.39
P08E05	0.58	1.96	−96.76	N.D.	N.D.
P08F05	0.58	12.21	−79.87	N.D.	N.D.
P08G05	0.58	67.40	11.12	43:21:51	−0.32
P08H05	0.58	106.15	75.00	42:38:27	−1.04
P08A06	0.58	92.21	52.02	47:32:36	3.86
P08B06	0.58	70.57	16.34	48:49:49	5.15
P08C06	0.58	79.18	30.53	44:25:33	0.75
P08D06	0.58	62.82	3.57	48:31:41	4.85
P08E06	0.58	79.33	30.78	48:31:41	4.85
P08F06	0.58	97.87	61.35	48:29:26	4.81
P08G06	0.58	66.57	9.75	40:12:17	−3.48
P08H06	0.58	110.86	82.75	48:13:25	4.54
P08A07	0.58	95.77	57.88	44:47:49	1.12
P08B07	0.58	91.94	51.56	45:40:24	1.99
P08C07	0.58	104.10	71.61	46:52:32	3.20
P08D07	0.58	71.44	17.77	48:13:38	4.55
P08E07	0.58	63.97	5.46	45:56:03	2.25
P08F07	0.58	106.48	75.54	55:17:19	11.61
P08G07	0.58	66.17	9.08	46:24:30	2.73
P08H07	0.58	96.28	58.72	45:38:47	1.97
P08A08	0.58	107.82	77.75	48:40:04	4.99
P08B08	0.58	104.22	71.81	45:33:29	1.88
P08C08	0.58	65.65	8.23	N.D.	N.D.
P08D08	0.58	71.17	17.32	47:36:53	3.93
P08E08	0.58	63.01	3.88	44:30:37	0.83
P08F08	0.58	98.05	61.64	54:31:32	10.85
P08G08	0.58	70.61	16.40	44:12:06	0.52
P08H08	0.58	67.83	11.83	40:38:36	−3.04
P08A09	0.58	73.65	21.42	46:19:07	2.64
P08B09	0.58	70.26	15.82	45:26:15	1.76
P08C09	0.58	63.28	4.32	43:11:34	−0.49
P08D09	0.58	94.33	55.52	44:37:10	0.94
P08E09	0.58	61.69	1.70	47:14:19	3.56
P08F09	0.58	58.02	−4.35	42:08:50	−1.53
P08G09	0.58	100.35	65.44	41:27:24	−2.22
P08H09	0.58	84.20	38.81	48:36:15	4.92
P08A10	0.58	59.48	−1.94	49:14:08	5.56
P08B10	0.58	68.19	12.41	41:25:45	−2.25
P08C10	0.58	74.39	22.64	45:18:12	1.62
P08D10	0.58	56.73	−6.47	44:11:40	0.51
P08E10	0.58	62.00	2.22	37:59:18	−5.69
P08F10	0.58	73.11	20.53	48:47:17	5.11
P08G10	0.58	70.31	15.91	42:06:01	−1.58
P08H10	0.58	70.29	15.87	41:45:03	−1.93
P08A11	0.58	42.82	−29.41	N.D.	N.D.
P08B11	0.58	80.97	33.49	53:53:55	10.22
P08C11	0.58	67.18	10.75	44:21:23	0.68
P08D11	0.58	99.74	64.43	47:53:25	4.21
P08E11	0.58	68.53	12.98	48:29:42	4.81
P08F11	0.58	63.90	5.34	45:05:08	1.41
P08G11	0.58	63.90	5.35	40:36:29	−3.07
P08H11	0.58	85.63	41.16	48:26:30	4.76
P09 untreated mock-infected cells	0.56	284.10 ± 27.03	N.D.	N.D.	N.D.
P09 untreated infected cells	0.56	61.25 ± 5.35	N.D.	46:25:57	N.D.
P09A02	0.56	76.21	24.43	48:02:41	1.61
P09B02	0.56	70.20	14.62	41:14:35	−5.19
P09C02	0.56	55.82	−8.86	45:02:13	−1.40
P09D02	0.56	51.61	−15.73	41:37:56	−4.80
P09E02	0.56	53.87	−12.05	44:42:44	−1.72
P09F02	0.56	57.59	−5.98	44:44:02	−1.70
P09G02	0.56	77.75	26.95	49:44:14	3.30
P09H02	0.56	65.51	6.95	41:25:29	−5.01
P09A03	0.56	41.78	−31.79	42:08:29	−4.29
P09B03	0.56	70.74	15.50	46:37:40	0.20
P09C03	0.56	70.86	15.69	46:43:11	0.29
P09D03	0.56	60.47	−1.27	48:27:00	2.02
P09E03	0.56	76.46	24.83	51:20:29	4.91
P09F03	0.56	84.60	38.12	52:17:48	5.86
P09G03	0.56	63.43	3.57	45:27:04	−0.98
P09H03	0.56	75.92	23.96	46:53:51	0.47
P09A04	0.56	47.24	−22.87	36:11:32	−10.24
P09B04	0.56	74.62	21.83	47:26:26	1.01
P09C04	0.56	59.54	−2.80	42:09:24	−4.28
P09D04	0.56	58.76	−4.06	42:48:22	−3.63
P09E04	0.56	47.81	−21.94	42:38:31	−3.79
P09F04	0.56	64.35	5.07	47:05:17	0.66
P09G04	0.56	50.78	−17.10	41:12:13	−5.23
P09H04	0.56	47.55	−22.37	40:29:31	−5.94
P09A05	0.56	72.02	17.58	40:53:38	−5.54
P09B05	0.56	66.73	8.94	42:27:09	−3.98
P09C05	0.56	53.54	−12.59	42:14:47	−4.19
P09D05	0.56	57.27	−6.49	45:28:30	−0.96
P09E05	0.56	58.96	−3.74	46:16:15	−0.16
P09F05	0.56	48.58	−20.68	40:09:05	−6.28
P09G05	0.56	61.82	0.94	43:38:32	−2.79
P09H05	0.56	65.24	6.51	43:42:48	−2.72
P09A06	0.56	71.41	16.58	43:24:31	−3.02
P09B06	0.56	55.83	−8.84	44:03:17	−2.38
P09C06	0.56	54.89	−10.38	42:45:48	−3.67
P09D06	0.56	42.69	−30.31	47:23:08	0.95
P09E06	0.56	64.59	5.46	47:23:08	0.95
P09F06	0.56	50.94	−16.83	53:17:19	6.86
P09G06	0.56	14.63	−76.12	N.D.	N.D.
P09H06	0.56	52.29	−14.62	43:35:09	−2.85
P09A07	0.56	20.51	−66.52	N.D.	N.D.
P09B07	0.56	35.20	−42.54	31:28:58	−14.95
P09C07	0.56	48.88	−20.20	42:15:55	−4.17
P09D07	0.56	57.74	−5.73	44:54:46	−1.52
P09E07	0.56	39.26	−35.90	N.D.	N.D.
P09F07	0.56	49.68	−18.89	43:09:30	−3.27
P09G07	0.56	64.27	4.93	49:00:41	2.58
P09H07	0.56	100.65	64.33	55:56:47	9.51
P09A08	0.56	53.17	−13.19	46:59:30	0.56
P09B08	0.56	70.94	15.83	40:13:25	−6.21
P09C08	0.56	57.53	−6.07	43:59:13	−2.45
P09D08	0.56	31.91	−47.91	34:35:33	−11.84
P09E08	0.56	62.18	1.52	48:16:47	1.85
P09F08	0.56	66.10	7.91	46:15:08	−0.18
P09G08	0.56	61.29	0.07	45:01:27	−1.41
P09H08	0.56	1.13	−98.16	N.D.	N.D.
P09A09	0.56	55.72	−9.03	44:34:05	−1.86
P09B09	0.56	37.80	−38.28	38:17:08	−8.15
P09C09	0.56	34.48	−43.71	37:24:34	−9.02
P09D09	0.56	61.12	−0.21	43:57:20	−2.48
P09E09	0.56	44.92	−26.67	41:32:44	−4.89
P09F09	0.56	70.50	15.11	50:32:10	4.10
P09G09	0.56	24.31	−60.32	N.D.	N.D.
P09H09	0.56	47.46	−22.51	38:29:29	−7.94
P09A10	0.56	57.05	−6.86	44:21:49	−2.07
P09B10	0.56	56.21	−8.23	45:03:48	−1.37
P09C10	0.56	78.00	27.34	47:21:08	0.92
P09D10	0.56	54.41	−11.16	40:45:55	−5.67
P09E10	0.56	64.28	4.95	50:09:37	3.73
P09F10	0.56	42.27	−30.98	45:37:22	−0.81
P09G10	0.56	52.00	−15.10	41:53:33	−4.54
P09H10	0.56	62.80	2.53	43:05:28	−3.34
P09A11	0.56	66.61	8.76	45:48:27	−0.62
P09B11	0.56	58.91	−3.83	40:31:39	−5.90
P09C11	0.56	53.00	−13.47	41:18:44	−5.12
P09D11	0.56	57.03	−6.89	42:20:13	−4.10
P09E11	0.56	47.85	−21.87	42:58:33	−3.46
P09F11	0.56	54.11	−11.66	44:29:00	−1.95
P09G11	0.56	38.71	−36.79	33:42:14	−12.73
P09H11	0.56	60.57	−1.11	42:58:10	−3.46
P10 untreated mock-infected cells	0.59	294.24 ± 26.36	N.D.	N.D.	N.D.
P10 untreated infected cells	0.59	50.08 ± 6.86	N.D.	46:19:34	N.D.
P10A02	0.59	63.70	27.18	46:40:36	0.35
P10B02	0.59	69.06	37.88	46:21:30	0.03
P10C02	0.59	64.63	29.05	46:33:52	0.24
P10D02	0.59	65.26	30.30	44:09:07	−2.17
P10E02	0.59	45.74	−8.68	43:22:22	−2.95
P10F02	0.59	62.80	25.39	49:12:38	2.88
P10G02	0.59	38.66	−22.81	36:38:49	−9.68
P10H02	0.59	38.43	−23.27	39:23:10	−6.94
P10A03	0.59	39.35	−21.43	40:14:27	−6.09
P10B03	0.59	50.98	1.79	43:54:53	−2.41
P10C03	0.59	58.82	17.44	45:51:49	−0.46
P10D03	0.59	41.03	−18.08	44:01:30	−2.30
P10E03	0.59	46.59	−6.97	40:07:36	−6.20
P10F03	0.59	45.62	−8.91	42:36:07	−3.72
P10G03	0.59	8.14	−83.75	N.D.	N.D.
P10H03	0.59	72.02	43.80	47:16:09	0.94
P10A04	0.59	45.57	−9.02	42:50:00	−3.49
P10B04	0.59	63.69	27.17	44:38:53	−1.68
P10C04	0.59	42.40	−15.35	38:07:01	−8.21
P10D04	0.59	79.21	58.16	47:40:19	1.35
P10E04	0.59	57.16	14.13	46:32:56	0.22
P10F04	0.59	62.86	25.51	44:51:49	−1.46
P10G04	0.59	76.78	53.31	45:33:46	−0.76
P10H04	0.59	64.88	29.55	51:21:53	5.04
P10A05	0.59	34.59	−30.93	36:20:54	−9.98
P10B05	0.59	36.83	−26.46	35:39:28	−10.67
P10C05	0.59	50.73	1.29	45:55:41	−0.40
P10D05	0.59	46.43	−7.29	45:21:08	−0.97
P10E05	0.59	56.05	11.91	43:43:18	−2.60
P10F05	0.59	44.86	−10.43	37:49:38	−8.50
P10G05	0.59	41.24	−17.66	38:23:30	−7.93
P10H05	0.59	60.77	21.33	44:52:48	−1.45
P10A06	0.59	48.38	−3.41	41:23:49	−4.93
P10B06	0.59	3.00	−94.02	N.D.	N.D.
P10C06	0.59	53.68	7.18	41:32:53	−4.78
P10D06	0.59	44.40	−11.35	42:24:22	−3.92
P10E06	0.59	28.36	−43.38	42:24:22	−3.92
P10F06	0.59	37.12	−25.89	39:12:14	−7.12
P10G06	0.59	42.26	−15.63	37:33:41	−8.76
P10H06	0.59	0.65	−98.71	N.D.	N.D.
P10A07	0.59	57.88	15.56	46:28:10	0.14
P10B07	0.59	53.58	6.98	42:26:01	−3.89
P10C07	0.59	1.67	−96.67	N.D.	N.D.
P10D07	0.59	37.51	−25.11	47:11:47	0.87
P10E07	0.59	50.02	−0.13	41:24:03	−4.93
P10F07	0.59	42.16	−15.82	39:03:05	−7.27
P10G07	0.59	39.03	−22.07	41:47:24	−4.54
P10H07	0.59	59.39	18.58	47:04:07	0.74
P10A08	0.59	60.39	20.58	39:13:48	−7.10
P10B08	0.59	55.63	11.08	39:13:36	−7.10
P10C08	0.59	38.15	−23.83	38:19:04	−8.01
P10D08	0.59	60.53	20.85	43:24:28	−2.92
P10E08	0.59	52.32	4.47	43:27:48	−2.86
P10F08	0.59	158.99	217.45	N.D.	N.D.
P10G08	0.59	43.88	−12.40	41:50:06	−4.49
P10H08	0.59	48.96	−2.25	47:05:25	0.76
P10A09	0.59	56.97	13.74	45:46:27	−0.55
P10B09	0.59	62.12	24.03	44:03:11	−2.27
P10C09	0.59	59.96	19.72	71:51:03	25.52
P10D09	0.59	58.25	16.32	47:13:31	0.90
P10E09	0.59	121.16	141.92	75:20:35	29.02
P10F09	0.59	66.87	33.52	52:51:43	6.54
P10G09	0.59	43.44	−13.27	36:00:49	−10.31
P10H09	0.59	43.74	−12.67	45:05:30	−1.23
P10A10	0.59	54.96	9.73	43:19:28	−3.00
P10B10	0.59	119.98	139.55	52:49:34	6.50
P10C10	0.59	71.39	42.54	49:30:54	3.19
P10D10	0.59	55.88	11.58	46:36:10	0.28
P10E10	0.59	48.46	−3.25	40:53:46	−5.43
P10F10	0.59	57.13	14.07	43:28:01	−2.86
P10G10	0.59	69.29	38.34	48:21:55	2.04
P10H10	0.59	68.13	36.03	46:42:18	0.38
P10A11	0.59	66.46	32.71	45:22:26	−0.95
P10B11	0.59	5.31	−89.40	N.D.	N.D.
P10C11	0.59	49.86	−0.44	41:07:00	−5.21
P10D11	0.59	53.01	5.84	45:55:42	−0.40
P10E11	0.59	51.47	2.77	42:47:20	−3.54
P10F11	0.59	43.73	−12.68	43:21:50	−2.96
P10G11	0.59	23.71	−52.65	N.D.	N.D.
P10H11	0.59	90.18	80.06	55:03:21	8.73
P11 untreated mock-infected cells	0.79	235.76 ± 9.34	N.D.	N.D.	N.D.
P11 untreated infected cells	0.79	53.01 ± 3.26	N.D.	42:13:36	N.D.
P11A02	0.79	64.21	21.12	46:16:25	4.05
P11B02	0.79	54.02	1.89	45:08:34	2.92
P11C02	0.79	54.50	2.80	42:01:55	−0.19
P11D02	0.79	61.01	15.08	45:55:24	3.70
P11E02	0.79	20.39	−61.54	N.D.	N.D.
P11F02	0.79	47.39	−10.61	41:25:38	−0.80
P11G02	0.79	55.21	4.14	41:21:32	−0.87
P11H02	0.79	7.23	−86.35	N.D.	N.D.
P11A03	0.79	64.21	21.12	42:43:22	0.50
P11B03	0.79	54.02	1.89	44:47:28	2.56
P11C03	0.79	54.50	2.80	41:04:50	−1.15
P11D03	0.79	61.01	15.08	42:23:44	0.17
P11E03	0.79	20.39	−61.54	51:15:05	9.02
P11F03	0.79	47.39	−10.61	46:04:55	3.86
P11G03	0.79	55.21	4.14	42:41:09	0.46
P11H03	0.79	7.23	−86.35	42:54:57	0.69
P11A04	0.79	58.41	10.17	42:55:17	0.69
P11B04	0.79	51.11	−3.59	42:20:48	0.12
P11C04	0.79	47.35	−10.68	42:02:35	−0.18
P11D04	0.79	55.42	4.55	46:03:40	3.83
P11E04	0.79	55.82	5.29	44:13:05	1.99
P11F04	0.79	56.64	6.84	39:42:13	−2.52
P11G04	0.79	58.65	10.64	44:03:24	1.83
P11H04	0.79	55.95	5.55	41:03:29	−1.17
P11A05	0.79	53.01	0.00	34:22:56	−7.84
P11B05	0.79	49.30	−7.01	42:23:30	0.17
P11C05	0.79	55.65	4.98	45:12:39	2.98
P11D05	0.79	53.31	0.56	44:30:05	2.27
P11E05	0.79	56.38	6.35	47:01:18	4.80
P11F05	0.79	45.00	−15.12	40:44:54	−1.48
P11G05	0.79	45.88	−13.45	40:58:27	−1.25
P11H05	0.79	52.21	−1.51	41:27:11	−0.77
P11A06	0.79	59.10	11.49	36:16:59	−5.94
P11B06	0.79	64.86	22.35	46:52:59	4.66
P11C06	0.79	52.58	−0.83	46:22:02	4.14
P11D06	0.79	64.93	22.48	45:22:24	3.15
P11E06	0.79	50.03	−5.62	45:22:24	3.15
P11F06	0.79	63.17	19.16	39:32:43	−2.68
P11G06	0.79	13.48	−74.57	N.D.	N.D.
P11H06	0.79	51.96	−1.98	40:16:43	−1.95
P11A07	0.79	76.29	43.91	43:55:19	1.70
P11B07	0.79	55.92	5.49	36:44:32	−5.48
P11C07	0.79	48.53	−8.46	45:19:12	3.09
P11D07	0.79	52.88	−0.25	43:11:29	0.96
P11E07	0.79	50.48	−4.77	43:36:23	1.38
P11F07	0.79	45.54	−14.10	40:03:53	−2.16
P11G07	0.79	47.54	−10.32	42:18:25	0.08
P11H07	0.79	66.66	25.75	46:37:20	4.40
P11A08	0.79	58.11	9.61	37:38:24	−4.59
P11B08	0.79	52.86	−0.29	39:00:32	−3.22
P11C08	0.79	51.33	−3.17	43:06:52	0.89
P11D08	0.79	54.11	2.08	44:39:03	2.42
P11E08	0.79	55.71	5.08	46:45:48	4.54
P11F08	0.79	53.69	1.28	45:47:57	3.57
P11G08	0.79	46.48	−12.32	38:17:23	−3.94
P11H08	0.79	54.60	2.99	40:55:37	−1.30
P11A09	0.79	78.22	47.55	38:03:23	−4.17
P11B09	0.79	62.26	17.44	44:55:09	2.69
P11C09	0.79	12.19	−77.00	N.D.	N.D.
P11D09	0.79	64.47	21.62	48:20:41	6.12
P11E09	0.79	43.00	−18.89	36:19:14	−5.91
P11F09	0.79	57.07	7.64	44:51:47	2.64
P11G09	0.79	55.02	3.78	42:25:29	0.20
P11H09	0.79	68.66	29.52	42:59:40	0.77
P11A10	0.79	49.58	−6.47	46:27:00	4.22
P11B10	0.79	60.84	14.76	50:08:19	7.91
P11C10	0.79	63.35	19.50	49:04:53	6.85
P11D10	0.79	216.98	309.29	136:59:42	94.77
P11E10	0.79	57.07	7.66	43:21:09	1.13
P11F10	0.79	16.96	−68.02	N.D.	N.D.
P11G10	0.79	52.95	−0.12	40:42:28	−1.52
P11H10	0.79	43.33	−18.26	41:38:04	−0.59
P11A11	0.79	48.93	−7.71	42:40:42	0.45
P11B11	0.79	60.67	14.45	44:42:13	2.48
P11C11	0.79	47.42	−10.55	42:36:43	0.39
P11D11	0.79	51.53	−2.79	45:27:19	3.23
P11E11	0.79	38.44	−27.49	42:53:24	0.66
P11F11	0.79	54.88	3.53	41:23:07	−0.84
P11G11	0.79	44.11	−16.79	N.D.	N.D.
P11H11	0.79	49.89	−5.89	38:46:34	−3.45
P12 untreated mock-infected cells	0.85	262.91 ± 3.58	N.D.	N.D.	N.D.
P12 untreated infected cells	0.85	55.56 ± 6.70	N.D.	44:17:43	N.D.
P12A02	0.85	70.99	27.77	45:46:20	1.48
P12B02	0.85	56.71	2.06	45:50:02	1.54
P12C02	0.85	42.83	−22.92	41:21:13	−2.94
P12D02	0.85	64.70	16.45	48:51:55	4.57
P12E02	0.85	49.62	−10.70	42:38:56	−1.65
P12F02	0.85	48.59	−12.55	45:19:50	1.04
P12G02	0.85	47.93	−13.74	42:39:40	−1.63
P12H02	0.85	50.82	−8.54	43:07:25	−1.17
P12A03	0.85	49.97	−10.07	41:57:35	−2.34
P12B03	0.85	52.71	−5.14	44:53:42	0.60
P12C03	0.85	49.02	−11.78	48:49:43	4.53
P12D03	0.85	32.32	−41.82	N.D.	N.D.
P12E03	0.85	44.00	−20.81	39:17:25	−5.00
P12F03	0.85	57.74	3.91	53:53:46	9.60
P12G03	0.85	53.11	−4.42	43:18:22	−0.99
P12H03	0.85	63.55	14.38	49:05:49	4.80
P12A04	0.85	56.69	2.03	39:28:51	−4.81
P12B04	0.85	62.24	12.01	50:06:50	5.82
P12C04	0.85	46.75	−15.87	40:06:40	−4.18
P12D04	0.85	56.10	0.97	45:19:01	1.02
P12E04	0.85	54.62	−1.69	44:32:59	0.25
P12F04	0.85	52.14	−6.16	45:20:49	1.05
P12G04	0.85	49.54	−10.85	45:23:20	1.09
P12H04	0.85	58.72	5.67	43:13:42	−1.07
P12A05	0.85	66.82	20.25	38:53:51	−5.40
P12B05	0.85	57.63	3.73	42:34:05	−1.73
P12C05	0.85	59.04	6.25	42:46:14	−1.52
P12D05	0.85	46.82	−15.74	43:01:53	−1.26
P12E05	0.85	55.57	0.01	42:33:11	−1.74
P12F05	0.85	56.07	0.92	39:15:39	−5.03
P12G05	0.85	52.93	−4.73	43:19:38	−0.97
P12H05	0.85	71.32	28.36	50:02:31	5.75
P12A06	0.85	1.83	−96.70	N.D.	N.D.
P12B06	0.85	67.06	20.68	42:32:11	−1.76
P12C06	0.85	56.63	1.93	44:47:20	0.49
P12D06	0.85	47.21	−15.03	38:37:43	−5.67
P12E06	0.85	9.01	−83.78	N.D.	N.D.
P12F06	0.85	57.03	2.64	36:52:51	−7.41
P12G06	0.85	50.34	−9.40	43:15:35	−1.04
P12H06	0.85	61.35	10.42	46:25:45	2.13
P12A07	0.85	49.35	−11.18	39:15:53	−5.03
P12B07	0.85	53.83	−3.12	42:26:59	−1.85
P12C07	0.85	63.99	15.17	51:39:17	7.36
P12D07	0.85	43.43	−21.84	40:07:58	−4.16
P12E07	0.85	46.92	−15.56	46:12:31	1.91
P12F07	0.85	51.75	−6.87	40:02:34	−4.25
P12G07	0.85	48.97	−11.86	37:39:37	−6.63
P12H07	0.85	54.10	−2.64	38:27:48	−5.83
P12A08	0.85	63.64	14.54	38:24:04	−5.89
P12B08	0.85	55.25	−0.56	43:14:04	−1.06
P12C08	0.85	49.92	−10.15	43:50:19	−0.46
P12D08	0.85	51.35	−7.59	45:58:24	1.68
P12E08	0.85	61.94	11.47	47:19:20	3.03
P12F08	0.85	51.05	−8.13	43:37:05	−0.68
P12G08	0.85	14.89	−73.20	N.D.	N.D.
P12H08	0.85	68.92	24.03	46:24:20	2.11
P12A09	0.85	56.31	1.34	48:51:32	4.56
P12B09	0.85	40.10	−27.83	43:48:03	−0.49
P12C09	0.85	48.29	−13.09	49:38:19	5.34
P12D09	0.85	47.96	−13.69	44:27:18	0.16
P12E09	0.85	42.27	−23.92	43:39:16	−0.64
P12F09	0.85	58.67	5.60	48:00:58	3.72
P12G09	0.85	52.90	−4.79	43:20:29	−0.95
P12H09	0.85	61.42	10.54	43:34:59	−0.71
P12A10	0.85	59.30	6.73	58:19:14	14.03
P12B10	0.85	49.55	−10.82	44:08:50	−0.15
P12C10	0.85	48.10	−13.43	46:13:14	1.93
P12D10	0.85	46.37	−16.54	43:58:59	−0.31
P12E10	0.85	45.00	−19.02	37:36:37	−6.68
P12F10	0.85	50.76	−8.65	43:04:31	−1.22
P12G10	0.85	67.36	21.23	44:26:02	0.14
P12H10	0.85	56.52	1.72	44:07:37	−0.17
P12A11	0.85	54.97	−1.08	44:05:41	−0.20
P12B11	0.85	51.79	−6.79	42:18:16	−1.99
P12C11	0.85	56.03	0.85	48:07:59	3.84
P12D11	0.85	55.04	−0.94	47:23:54	3.10
P12E11	0.85	42.29	−23.89	43:13:57	−1.06
P12F11	0.85	46.95	−15.50	43:24:39	−0.88
P12G11	0.85	57.69	3.83	44:25:06	0.12
P12H11	0.85	59.40	6.90	43:55:32	−0.37
P13 untreated mock-infected cells	0.87	240.87 ± 6.33	N.D.	N.D.	N.D.
P13 untreated infected cells	0.87	45.97 ± 2.20	N.D.	40:36:49	N.D.
P13A02	0.87	49.26	7.15	43:58:51	3.37
P13B02	0.87	57.56	25.21	43:38:14	3.02
P13C02	0.87	41.33	−10.08	42:14:35	1.63
P13D02	0.87	39.97	−13.05	41:43:23	1.11
P13E02	0.87	41.15	−10.49	41:24:39	0.80
P13F02	0.87	42.64	−7.24	38:55:58	−1.68
P13G02	0.87	48.18	4.81	41:21:52	0.75
P13H02	0.87	61.46	33.71	53:29:12	12.87
P13A03	0.87	44.69	−2.79	43:07:36	2.51
P13B03	0.87	60.56	31.74	47:20:41	6.73
P13C03	0.87	48.01	4.44	44:08:28	3.53
P13D03	0.87	45.84	−0.28	43:05:53	2.48
P13E03	0.87	48.40	5.28	48:13:03	7.60
P13F03	0.87	43.22	−5.97	41:26:46	0.83
P13G03	0.87	48.61	5.76	42:04:35	1.46
P13H03	0.87	54.65	18.88	45:49:47	5.22
P13A04	0.87	31.31	−31.90	N.D.	N.D.
P13B04	0.87	58.30	26.84	48:56:41	8.33
P13C04	0.87	73.03	58.88	48:53:04	8.27
P13D04	0.87	34.13	−25.75	N.D.	N.D.
P13E04	0.87	51.41	11.85	45:25:13	4.81
P13F04	0.87	49.67	8.05	46:32:05	5.92
P13G04	0.87	59.37	29.15	45:47:38	5.18
P13H04	0.87	52.62	14.48	43:34:03	2.95
P13A05	0.87	48.10	4.65	43:23:47	2.78
P13B05	0.87	46.32	0.76	42:59:25	2.38
P13C05	0.87	47.50	3.34	42:02:43	1.43
P13D05	0.87	46.84	1.90	45:03:12	4.44
P13E05	0.87	37.83	−17.71	39:23:49	−1.22
P13F05	0.87	35.96	−21.77	41:14:45	0.63
P13G05	0.87	53.06	15.42	42:24:53	1.80
P13H05	0.87	51.37	11.75	42:11:05	1.57
P13A06	0.87	53.69	16.80	46:48:29	6.19
P13B06	0.87	48.28	5.03	44:36:33	4.00
P13C06	0.87	45.27	−1.52	42:51:25	2.24
P13D06	0.87	50.07	8.93	45:23:56	4.79
P13E06	0.87	49.83	8.40	45:23:56	4.79
P13F06	0.87	43.34	−5.72	41:55:23	1.31
P13G06	0.87	50.14	9.07	45:09:23	4.54
P13H06	0.87	49.32	7.30	43:57:53	3.35
P13A07	0.87	50.22	9.24	42:34:14	1.96
P13B07	0.87	43.19	−6.05	38:27:50	−2.15
P13C07	0.87	28.92	−37.09	N.D.	N.D.
P13D07	0.87	46.03	0.13	42:59:45	2.38
P13E07	0.87	43.07	−6.29	38:38:37	−1.97
P13F07	0.87	50.01	8.79	40:20:53	−0.27
P13G07	0.87	45.84	−0.28	41:43:31	1.11
P13H07	0.87	48.25	4.97	40:49:27	0.21
P13A08	0.87	45.47	−1.08	41:13:32	0.61
P13B08	0.87	53.40	16.16	44:55:54	4.32
P13C08	0.87	0.35	−99.24	N.D.	N.D.
P13D08	0.87	49.11	6.84	38:49:42	−1.79
P13E08	0.87	46.15	0.40	40:32:28	−0.07
P13F08	0.87	66.58	44.85	44:56:23	4.33
P13G08	0.87	56.56	23.04	43:24:52	2.80
P13H08	0.87	49.77	8.27	43:31:39	2.91
P13A09	0.87	43.73	−4.87	44:28:42	3.86
P13B09	0.87	50.38	9.60	41:18:47	0.70
P13C09	0.87	47.11	2.48	41:58:24	1.36
P13D09	0.87	47.53	3.39	44:13:15	3.61
P13E09	0.87	46.41	0.95	39:25:20	−1.19
P13F09	0.87	57.49	25.07	44:13:45	3.62
P13G09	0.87	55.82	21.44	43:13:33	2.61
P13H09	0.87	40.07	−12.84	43:04:27	2.46
P13A10	0.87	48.58	5.69	44:47:35	4.18
P13B10	0.87	48.50	5.51	42:51:05	2.24
P13C10	0.87	49.52	7.73	41:25:20	0.81
P13D10	0.87	54.81	19.24	47:07:39	6.51
P13E10	0.87	50.82	10.56	43:52:42	3.26
P13F10	0.87	47.57	3.48	39:34:29	−1.04
P13G10	0.87	51.66	12.38	41:01:39	0.41
P13H10	0.87	51.40	11.81	43:44:03	3.12
P13A11	0.87	58.29	26.80	49:09:11	8.54
P13B11	0.87	45.30	−1.44	40:28:18	−0.14
P13C11	0.87	45.83	−0.30	41:23:07	0.77
P13D11	0.87	49.10	6.81	42:25:21	1.81
P13E11	0.87	45.22	−1.63	39:48:05	−0.81
P13F11	0.87	51.83	12.76	38:05:42	−2.52
P13G11	0.87	49.62	7.94	42:21:46	1.75
P13H11	0.87	45.54	−0.92	22:16:26	−18.34
P14 untreated mock-infected cells	0.52	309.86 ± 39.49	N.D.	N.D.	N.D.
P14 untreated infected cells	0.52	48.43 ± 2.22	N.D.	43:27:35	N.D.
P14A02	0.52	52.72	8.85	44:38:04	1.17
P14B02	0.52	73.67	52.12	43:51:33	0.40
P14C02	0.52	40.91	−15.52	40:24:35	−3.05
P14D02	0.52	48.61	0.38	46:32:27	3.08
P14E02	0.52	51.23	5.78	46:26:48	2.99
P14F02	0.52	52.43	8.25	43:12:44	−0.25
P14G02	0.52	43.29	−10.62	41:07:01	−2.34
P14H02	0.52	62.55	29.16	48:53:27	5.43
P14A03	0.52	54.97	13.49	44:45:45	1.30
P14B03	0.52	50.22	3.70	44:30:09	1.04
P14C03	0.52	62.72	29.50	47:45:24	4.30
P14D03	0.52	40.77	−15.83	40:31:55	−2.93
P14E03	0.52	89.42	84.64	51:59:07	8.53
P14F03	0.52	59.10	22.03	39:12:13	−4.26
P14G03	0.52	54.66	12.87	43:31:04	0.06
P14H03	0.52	53.67	10.83	44:57:40	1.50
P14A04	0.52	82.86	71.08	49:20:13	5.88
P14B04	0.52	46.67	−3.63	44:15:49	0.80
P14C04	0.52	54.84	13.24	45:15:37	1.80
P14D04	0.52	50.00	3.24	43:36:22	0.15
P14E04	0.52	48.35	−0.17	41:31:01	−1.94
P14F04	0.52	29.50	−39.09	N.D.	N.D.
P14G04	0.52	48.95	1.08	43:48:02	0.34
P14H04	0.52	54.11	11.72	43:09:01	−0.31
P14A05	0.52	69.33	43.15	40:30:05	−2.96
P14B05	0.52	48.62	0.38	43:37:58	0.17
P14C05	0.52	52.92	9.28	42:07:36	−1.33
P14D05	0.52	45.45	−6.16	41:29:52	−1.96
P14E05	0.52	48.36	−0.15	43:47:32	0.33
P14F05	0.52	46.99	−2.97	44:41:58	1.24
P14G05	0.52	47.47	−1.99	43:44:17	0.28
P14H05	0.52	46.29	−4.42	42:22:02	−1.09
P14A06	0.52	31.97	−34.00	40:42:24	−2.75
P14B06	0.52	51.69	6.73	45:17:03	1.82
P14C06	0.52	45.87	−5.29	42:25:44	−1.03
P14D06	0.52	54.73	13.02	46:36:33	3.15
P14E06	0.52	44.23	−8.68	46:36:33	3.15
P14F06	0.52	47.36	−2.21	42:08:41	−1.32
P14G06	0.52	50.51	4.29	46:29:03	3.02
P14H06	0.52	44.83	−7.44	41:43:30	−1.73
P14A07	0.52	68.89	42.25	43:05:12	−0.37
P14B07	0.52	115.04	137.53	108:06:35	64.65
P14C07	0.52	37.93	−21.68	39:17:19	−4.17
P14D07	0.52	52.56	8.53	42:09:59	−1.29
P14E07	0.52	52.50	8.41	43:55:34	0.47
P14F07	0.52	32.41	−33.08	40:44:29	−2.72
P14G07	0.52	55.27	14.12	45:05:19	1.63
P14H07	0.52	45.13	−6.81	43:26:11	−0.02
P14B08	0.52	59.00	21.82	44:15:55	0.81
P14C08	0.52	43.28	−10.64	38:44:35	−4.72
P14D08	0.52	51.86	7.07	45:10:32	1.72
P14E08	0.52	50.46	4.19	39:34:00	−3.89
P14F08	0.52	65.84	35.95	48:40:19	5.21
P14G08	0.52	52.21	7.81	44:29:41	1.03
P14H08	0.52	50.50	4.28	42:59:07	−0.47
P14A09	0.52	42.45	−12.35	40:19:31	−3.13
P14B09	0.52	53.00	9.43	46:02:10	2.58
P14C09	0.52	57.31	18.34	58:42:38	15.25
P14D09	0.52	44.64	−7.82	42:06:58	−1.34
P14E09	0.52	53.45	10.36	41:53:46	−1.56
P14F09	0.52	36.48	−24.68	43:05:16	−0.37
P14G09	0.52	49.07	1.32	42:42:19	−0.75
P14H09	0.52	48.89	0.95	43:12:15	−0.26
P14A10	0.52	0.79	−98.37	N.D.	N.D.
P14B10	0.52	53.55	10.57	45:02:09	1.58
P14C10	0.52	50.86	5.02	46:12:45	2.75
P14D10	0.52	44.39	−8.35	43:40:06	0.21
P14E10	0.52	38.76	−19.96	36:47:35	−6.67
P14F10	0.52	42.43	−12.39	39:24:03	−4.06
P14G10	0.52	37.65	−22.25	35:18:15	−8.16
P14H10	0.52	49.55	2.31	35:39:56	−7.79
P14A11	0.52	50.23	3.71	40:51:12	−2.61
P14B11	0.52	50.38	4.02	44:12:07	0.74
P14C11	0.52	38.68	−20.14	39:52:39	−3.58
P14D11	0.52	36.22	−25.21	14:50:19	−28.62
P14E11	0.52	45.31	−6.44	40:56:35	−2.52
P14F11	0.52	44.12	−8.90	41:37:27	−1.84
P14G11	0.52	37.02	−23.57	N.D.	N.D.
P14H11	0.52	49.96	3.17	42:34:10	−0.89
P16 untreated mock-infected cells	0.65	325.19 ± 19.16	N.D.	N.D.	N.D.
P16 untreated infected cells	0.65	72.55 ± 10.12	N.D.	48:07:24	N.D.
P16A02	0.65	22.74	−68.66	N.D.	N.D.
P16B02	0.65	65.78	−9.34	47:20:12	−0.79
P16C02	0.65	46.70	−35.63	N.D.	N.D.
P16D02	0.65	15.71	−78.34	N.D.	N.D.
P16E02	0.65	80.53	10.99	51:17:28	3.17
P16F02	0.65	82.54	13.76	48:15:35	0.14
P16G02	0.65	78.92	8.78	N.D.	N.D.
P16H02	0.65	92.61	27.64	59:06:09	10.98
P16A03	0.65	100.87	39.04	52:19:16	4.20
P16B03	0.65	87.64	20.80	52:07:04	3.99
P16C03	0.65	88.51	21.99	49:08:25	1.02
P16D03	0.65	101.15	39.42	52:43:34	4.60
P16E03	0.65	65.01	−10.39	42:41:04	−5.44
P16F03	0.65	74.18	2.24	48:10:46	0.06
P16G03	0.65	72.93	0.52	41:15:17	−6.87
P16H03	0.65	81.03	11.69	47:16:49	−0.84
P16A04	0.65	100.19	38.09	48:06:50	−0.01
P16B04	0.65	117.36	61.76	49:18:48	1.19
P16C04	0.65	139.51	92.29	51:35:19	3.47
P16D04	0.65	51.78	−28.63	54:09:56	6.04
P16E04	0.65	80.54	11.01	46:28:05	−1.66
P16F04	0.65	64.21	−11.51	47:25:47	−0.69
P16G04	0.65	136.34	87.91	58:52:37	10.75
P16H04	0.65	94.13	29.75	43:43:36	−4.40
P16A05	0.65	123.50	70.23	57:08:50	9.02
P16B05	0.65	47.28	−34.83	N.D.	N.D.
P16C05	0.65	106.68	47.04	N.D.	N.D.
P16D05	0.65	82.80	14.13	53:18:05	5.18
P16E05	0.65	36.21	−50.09	N.D.	N.D.
P16F05	0.65	112.15	54.58	49:08:38	1.02
P16G05	0.65	33.24	−54.19	N.D.	N.D.
P16H05	0.65	85.15	17.36	50:27:34	2.34
P16A06	0.65	84.58	16.58	44:36:58	−3.51
P16B06	0.65	62.51	−13.84	45:38:16	−2.49
P16C06	0.65	80.23	10.58	47:10:24	−0.95
P16D06	0.65	118.79	63.73	60:30:56	12.39
P16E06	0.65	58.75	−19.03	60:30:56	12.39
P16F06	0.65	90.81	25.16	48:30:57	0.39
P16G06	0.65	104.23	43.66	47:23:24	−0.73
P16H06	0.65	99.63	37.33	51:55:42	3.80
P16A07	0.65	94.94	30.85	49:17:53	1.17
P16B07	0.65	91.68	26.36	45:17:25	−2.83
P16C07	0.65	99.82	37.58	50:13:50	2.11
P16D07	0.65	85.81	18.27	47:22:31	−0.75
P16E07	0.65	94.82	30.70	47:22:13	−0.75
P16F07	0.65	72.78	0.32	40:58:06	−7.16
P16G07	0.65	77.03	6.17	51:15:17	3.13
P16H07	0.65	88.31	21.72	42:39:59	−5.46
P16A08	0.65	92.80	27.91	57:39:01	9.53
P16B08	0.65	39.48	−45.59	42:29:12	−5.64
P16C08	0.65	66.18	−8.78	42:40:52	−5.44
P16D08	0.65	77.07	6.23	48:35:04	0.46
P16E08	0.65	104.06	43.43	52:53:57	4.78
P16F08	0.65	115.42	59.09	51:57:14	3.83
P16G08	0.65	58.49	−19.39	39:31:57	−8.59
P16H08	0.65	47.76	−34.18	44:14:45	−3.88
P16A09	0.65	97.25	34.05	46:32:16	−1.59
P16B09	0.65	60.32	−16.86	47:33:56	−0.56
P16C09	0.65	65.14	−10.22	48:21:49	0.24
P16D09	0.65	84.42	16.36	44:58:28	−3.15
P16E09	0.65	90.70	25.01	52:29:13	4.36
P16F09	0.65	69.02	−4.87	39:40:11	−8.45
P16G09	0.65	156.75	116.05	60:23:56	12.28
P16H09	0.65	110.18	51.86	52:06:08	3.98
P16A10	0.65	123.72	70.53	62:49:52	14.71
P16B10	0.65	83.79	15.49	51:37:10	3.50
P16C10	0.65	54.08	−25.46	49:52:16	1.75
P16D10	0.65	87.47	20.56	45:57:42	−2.16
P16E10	0.65	15.30	−78.91	N.D.	N.D.
P16F10	0.65	119.75	65.06	51:05:19	2.97
P16G10	0.65	75.11	3.53	46:02:46	−2.08
P16H10	0.65	129.15	78.01	48:27:01	0.33
P16A11	0.65	104.71	44.32	51:04:28	2.95
P16B11	0.65	37.29	−48.61	N.D.	N.D.
P16C11	0.65	86.83	19.68	50:46:23	2.65
P16D11	0.65	90.03	24.09	47:51:31	−0.26
P16E11	0.65	82.22	13.32	47:23:57	−0.72
P16F11	0.65	68.28	−5.88	42:23:46	−5.73
P16G11	0.65	50.99	−29.73	39:41:53	−8.43
P16H11	0.65	97.78	34.77	44:40:25	−3.45

**Table 2:** Screening of 1,651 compounds from the Centre d'Etudes et de Recherche sur le Médicament de Normandie (CERMN, Caen, France) at 10 µM. For each compound, the following values are provided: the area under normalised Cell Index curve (AUC_n_), the %AUC_n_ compared to AUC_n_ of untreated infected cells, the time required for the cell index to decrease by 50 % after virus infection (CIT_50_) and the ΔCIT_50_ value obtained by comparing results for treated *vs* untreated, EHV-1-infected control wells. The Z’-factor was used to assess the quality of the screening in 96-well format. For untreated mock-infected and untreated infected cells, the mean ± SD of AUC_n_ is reported. *N.D., not determinable.*

Compound name	Z'-factor	AUC_n_	%AUC_n_	CIT_50_	ΔCIT_50_
C01 untreated mock-infected cells	0.79	200.89 ± 6.27	N.D	N.D	N.D
C02 untreated infected cells	0.79	51.05 ± 4.22	N.D	44:33:17	N.D
CEmr-2967200	0.79	56.69	11.07	48:39:22	8.10
CEsr-2708000	0.79	52.15	2.16	38:48:07	−1.75
CEmr-1642100	0.79	39.72	−22.19	39:29:39	−1.06
CEsr-2713000	0.79	55.45	8.63	35:57:21	−4.60
CEsr-2704000	0.79	46.60	−8.72	37:15:38	−3.29
CEmr-1506400	0.79	48.37	−5.25	36:36:37	−3.94
CEsr-2036000	0.79	43.53	−14.72	36:56:02	−3.62
CEsr-4857000	0.79	52.24	2.34	39:12:32	−1.35
CEsr-4858000	0.79	47.02	−7.90	35:59:38	−4.56
CEsr-4860000	0.79	48.96	−4.09	37:10:03	−3.39
CEmr-2558600	0.79	49.41	−3.21	38:08:38	−2.41
CEsr-6941000	0.79	46.78	−8.36	36:38:04	−3.92
CEsr-6305000	0.79	44.48	−12.87	37:47:31	−2.76
CEsr-6698000	0.79	44.33	−13.17	35:50:51	−4.71
CEsr-2564000	0.79	44.20	−13.41	34:46:48	−5.77
CEmr-2242400	0.79	63.72	24.82	43:21:50	2.81
CEmr-2693000	0.79	52.83	3.49	38:37:51	−1.92
CEmr-2577700	0.79	53.33	4.47	40:23:24	−0.16
CEmr-2242100	0.79	54.45	6.67	39:09:09	−1.40
CEmr-3182300	0.79	50.08	−1.90	34:51:04	−5.70
CEmr-3069500	0.79	43.52	−14.75	35:22:53	−5.17
CEmr-3069600	0.79	51.01	−0.08	42:37:14	2.07
CEmr-3068600	0.79	33.97	−33.44	40:06:15	−0.45
CEsr-5054000	0.79	52.80	3.44	42:24:57	1.86
CEsr-2719000	0.79	56.08	9.85	41:18:31	0.75
CEsr-1342000	0.79	63.70	24.79	40:25:46	−0.13
CEsr-4369000	0.79	58.90	15.39	38:35:06	−1.97
CEsr-7174000	0.79	50.34	−1.38	42:41:45	2.14
CEsr-7401000	0.79	45.89	−10.10	36:40:35	−3.88
CEsr-6415000	0.79	40.92	−19.84	37:33:34	−3.00
CEsr-5002000	0.79	49.14	−3.74	38:55:38	−1.63
CEmr-2502000	0.79	63.05	23.51	43:14:07	2.68
CEsr-2566000	0.79	53.90	5.59	42:18:19	1.75
CEsr-2551000	0.79	48.42	−5.15	39:47:43	−0.76
CEsr-2544000	0.79	43.63	−14.52	34:49:23	−5.73
CEsr-6413000	0.79	44.91	−12.02	36:09:32	−4.40
CEmr-2870400	0.79	48.05	−5.87	38:41:06	−1.87
CEmr-2601100	0.79	40.93	−19.82	36:50:42	−3.71
CEsr-1038000	0.79	60.27	18.06	39:46:43	−0.78
CEsr-1308000	0.79	40.11	−21.43	14:38:13	−25.92
CEsr-4292000	0.79	47.61	−6.74	36:20:51	−4.21
CEsr-4896000	0.79	52.33	2.51	37:03:48	−3.49
CEsr-4204000	0.79	42.26	−17.22	36:07:04	−4.44
CEmr-3132500	0.79	44.51	−12.80	35:58:10	−4.59
CEmr-2570900	0.79	43.35	−15.08	36:36:36	−3.94
CEmr-1729400	0.79	49.42	−3.18	36:57:57	−3.59
CEmr-3181200	0.79	44.23	−13.35	38:34:10	−1.99
CEsr-5549000	0.79	63.74	24.87	40:06:23	−0.45
CEmr-2394700	0.79	51.17	0.24	39:55:28	−0.63
CEsr-3090000	0.79	48.33	−5.32	37:44:08	−2.82
CEsr-6822000	0.79	44.49	−12.85	35:18:37	−5.24
CEmr-1920800	0.79	46.05	−9.78	37:43:17	−2.83
CEmr-1513300	0.79	42.29	−17.16	35:02:13	−5.52
CEsr-4726000	0.79	46.24	−9.41	35:58:18	−4.58
CEmr-1457400	0.79	50.74	−0.60	37:28:11	−3.08
CEsr-4650000	0.79	55.51	8.74	40:43:20	0.17
CEsr-6179000	0.79	56.03	9.76	39:14:55	−1.31
CEmr-2552200	0.79	51.47	0.83	37:17:46	−3.26
CEsr-7571000	0.79	27.25	−46.62	20:13:52	−20.32
CEsr-4882000	0.79	51.60	1.09	37:15:03	−3.30
CEsr-4879000	0.79	48.65	−4.69	36:59:24	−3.56
CEmr-2300900	0.79	55.73	9.18	37:13:41	−3.33
CEsr-5700000	0.79	54.11	6.00	39:48:19	−0.75
CEmr-2586800	0.79	63.18	23.77	42:59:43	2.44
CEmr-2778300	0.79	56.78	11.24	37:50:50	−2.71
CEsr-6469000	0.79	61.87	21.21	44:39:04	4.10
CEmr-1812200	0.79	56.76	11.19	38:31:24	−2.03
CEsr-6818000	0.79	59.06	15.70	40:26:12	−0.12
CEsr-4888000	0.79	43.02	−15.72	34:04:00	−6.49
CEmr-2512300	0.79	46.01	−9.86	37:45:17	−2.80
CEmr-3115100	0.79	53.23	4.27	41:16:17	0.72
CEsr-6001000	0.79	50.86	−0.36	38:38:41	−1.91
CEsr-5875000	0.79	53.20	4.22	36:55:13	−3.63
CEsr-5519000	0.79	56.73	11.13	36:18:41	−4.24
CEmr-1329700	0.79	57.09	11.85	38:18:43	−2.24
CEmr-1457700	0.79	54.98	7.71	37:34:28	−2.98
CEsr-7263000	0.79	54.95	7.65	39:02:18	−1.52
CEsr-4898000	0.79	52.49	2.83	36:40:49	−3.87
CEmr-2553000	0.79	56.68	11.03	39:39:55	−0.89
CEmr-1943500	0.79	49.36	−3.31	37:41:09	−2.87
C02 untreated mock-infected cells	0.878037237	202.44 ± 2.58	N.D	N.D	N.D
C02 untreated infected cells	0.88	48.35 ± 3.69	N.D	36:51:03	N.D
CEsr-3085000	0.88	60.77	25.69	39:57:43	3.11
CEmr-2558400	0.88	54.15	12.00	40:16:44	3.43
CEsr-5906000	0.88	51.04	5.57	40:22:53	3.53
CEsr-3914000	0.88	49.56	2.51	35:48:05	−1.05
CEsr-4161000	0.88	47.91	−0.91	39:49:00	2.97
CEmr-3137100	0.88	51.75	7.04	39:27:38	2.61
CEsr-5677000	0.88	26.91	−44.33	39:02:34	2.19
CEsr-2541000	0.88	51.69	6.91	40:22:52	3.53
CEsr-1443000	0.88	46.54	−3.73	37:40:31	0.82
CEsr-1286000	0.88	49.79	2.99	38:18:48	1.46
CEsr-2487000	0.88	53.01	9.65	40:55:53	4.08
CEmr-3394000	0.88	60.09	24.29	41:17:40	4.44
CEsr-7181000	0.88	45.20	−6.52	35:08:23	−1.71
CEmr-2362000	0.88	51.19	5.88	38:31:53	1.68
CEmr-2502200	0.88	54.54	12.82	41:10:11	4.32
CEsr-1214000	0.88	77.09	59.46	40:56:01	4.08
CEmr-1813300	0.88	60.16	24.43	42:10:29	5.32
CEsr-4153000	0.88	50.79	5.05	39:54:15	3.05
CEmr-3398000	0.88	47.86	−1.00	37:54:18	1.05
CEmr-1329300	0.88	44.66	−7.63	36:09:30	−0.69
CEsr-2529000	0.88	49.23	1.83	38:27:46	1.61
CEsr-3856000	0.88	52.62	8.84	39:27:56	2.61
CEsr-3654000	0.88	52.48	8.55	39:37:10	2.77
CEmr-1365200	0.88	51.46	6.44	41:39:58	4.82
CEsr-4989000	0.88	51.48	6.48	38:38:51	1.80
CEsr-7295000	0.88	56.79	17.46	42:48:58	5.97
CEsr-4428000	0.88	51.04	5.57	38:58:56	2.13
CEsr-3924000	0.88	47.22	−2.33	37:41:13	0.84
CEsr-2749000	0.88	44.26	−8.45	37:23:14	0.54
CEmr-3189600	0.88	32.74	−32.28	35:53:18	−0.96
CEsr-5612000	0.88	49.03	1.41	36:35:00	−0.27
CEsr-1477000	0.88	51.69	6.91	39:05:07	2.23
CEsr-3228000	0.88	44.62	−7.70	37:15:03	0.40
CEsr-5049000	0.88	46.91	−2.96	40:06:14	3.25
CEmr-22848a0	0.88	55.30	14.39	40:00:00	3.15
CEmr-1724100	0.88	44.90	−7.13	35:22:57	−1.47
CEmr-1724000	0.88	50.43	4.32	38:03:44	1.21
CEsr-3207000	0.88	51.62	6.76	36:52:47	0.03
CEmr-1678500	0.88	52.38	8.35	40:04:20	3.22
CEmr-2792400	0.88	57.17	18.26	42:23:16	5.54
CEmr-2872100	0.88	44.27	−8.43	37:55:52	1.08
CEsr-5826000	0.88	52.65	8.91	37:26:50	0.60
CEsr-5804000	0.88	47.19	−2.38	37:46:45	0.93
CEsr-3558000	0.88	46.81	−3.17	36:28:06	−0.38
CEsr-3569000	0.88	50.64	4.74	38:34:59	1.73
CEmr-2325500	0.88	58.03	20.04	38:23:30	1.54
CEsr-5558000	0.88	50.58	4.62	38:43:28	1.87
CEmr-3201900	0.88	55.88	15.58	40:45:41	3.91
CEmr-3093700	0.88	54.80	13.36	41:18:48	4.46
CEsr-5547000	0.88	46.33	−4.17	36:54:09	0.05
CEsr-3437000	0.88	44.12	−8.74	36:15:37	−0.59
CEsr-2333000	0.88	47.50	−1.75	35:48:02	−1.05
CEmr-2387800	0.88	44.32	−8.32	35:53:58	−0.95
CEsr-4918000	0.88	51.87	7.29	37:12:03	0.35
CEmr-2501400	0.88	48.02	−0.68	38:11:53	1.35
CEmr-2876800	0.88	55.91	15.65	41:26:48	4.60
CEsr-1298000	0.88	59.10	22.24	39:57:36	3.11
CEmr-2530500	0.88	54.29	12.30	40:38:11	3.79
CEsr-6975000	0.88	47.04	−2.71	37:54:38	1.06
CEsr-5387000	0.88	43.98	−9.02	34:54:57	−1.93
CEsr-5793000	0.88	44.12	−8.74	37:14:42	0.39
CEsr-5334000	0.88	46.12	−4.60	35:44:16	−1.11
CEmr-2384300	0.88	49.18	1.72	38:56:40	2.09
CEsr-5335000	0.88	42.90	−11.27	39:01:54	2.18
CEmr-2384700	0.88	48.19	−0.32	38:35:16	1.74
CEsr-5398000	0.88	48.34	−0.01	38:33:12	1.70
CEsr-5256000	0.88	50.52	4.51	37:44:12	0.89
CEsr-5265000	0.88	44.41	−8.14	36:59:02	0.13
CEsr-5778000	0.88	45.09	−6.73	37:15:27	0.41
CEmr-1842600	0.88	46.13	−4.58	38:03:37	1.21
CEsr-5294000	0.88	43.96	−9.07	38:15:44	1.41
CEmr-1478200	0.88	48.46	0.24	40:42:36	3.86
CEmr-2108500	0.88	46.06	−4.73	37:42:10	0.85
CEmr-1845900	0.88	50.63	4.73	39:52:53	3.03
CEmr-1676800	0.88	48.29	−0.11	37:51:21	1.01
CEsr-5295000	0.88	44.10	−8.78	36:29:11	−0.36
CEmr-2503400	0.88	52.94	9.50	41:34:31	4.72
CEsr-7169000	0.88	31.79	−34.25	40:28:51	3.63
CEmr-2707200	0.88	50.02	3.47	37:53:27	1.04
CEmr-3139400	0.88	46.43	−3.97	38:18:49	1.46
C03 untreated mock-infected cells	0.65560864	212.29 ± 15.22	N.D	N.D	N.D
C03 untreated infected cells	0.66	46.67 ± 3.79	N.D	37:28:10	N.D
CEmr-2812000	0.66	51.15	9.59	39:30:41	2.04
CEsr-4893000	0.66	47.74	2.28	36:30:53	−0.95
CEsr-2108000	0.66	43.89	−5.97	38:57:24	1.49
CEsr-6198000	0.66	44.34	−5.00	35:48:23	−1.66
CEmr-1506300	0.66	50.25	7.66	36:44:12	−0.73
CEmr-2552300	0.66	50.84	8.92	37:28:26	0.00
CEmr-1848100	0.66	46.67	0.00	36:59:01	−0.49
CEmr-3228900	0.66	39.85	−14.61	35:58:52	−1.49
CEmr-3181300	0.66	46.07	−1.30	46:51:59	9.40
CEsr-5350000	0.66	39.82	−14.68	35:23:20	−2.08
CEsr-2136000	0.66	48.34	3.57	39:42:25	2.24
CEmr-2984900	0.66	44.86	−3.88	36:32:29	−0.93
CEsr-7527000	0.66	47.16	1.04	36:13:08	−1.25
CEsr-5626000	0.66	47.62	2.02	36:58:33	−0.49
CEmr-2134200	0.66	66.37	42.20	42:16:45	4.81
CEsr-1724000	0.66	50.65	8.52	38:19:58	0.86
CEsr-5000000	0.66	38.65	−17.20	39:41:42	2.23
CEsr-2123000	0.66	50.21	7.57	39:38:37	2.17
CEsr-1686000	0.66	51.66	10.68	38:44:13	1.27
CEmr-2758700	0.66	46.64	−0.07	37:13:59	−0.24
CEsr-6220000	0.66	51.83	11.04	36:44:55	−0.72
CEmr-2987700	0.66	46.49	−0.40	36:14:53	−1.22
CEsr-3385000	0.66	47.80	2.41	40:05:44	2.63
CEmr-3391600	0.66	46.38	−0.64	38:58:29	1.51
CEmr-3392600	0.66	47.86	2.55	39:15:44	1.79
CEsr-1427000	0.66	50.15	7.45	38:29:52	1.03
CEsr-7085000	0.66	32.08	−31.27	36:02:08	−1.43
CEsr-7341000	0.66	48.66	4.26	37:43:54	0.26
CEsr-1114000	0.66	48.16	3.18	38:02:25	0.57
CEmr-2558800	0.66	42.82	−8.26	37:50:49	0.38
CEmr-1728800	0.66	47.24	1.21	39:40:58	2.21
CEmr-2661700	0.66	58.69	25.74	53:14:07	15.77
CEmr-2034000	0.66	53.74	15.14	38:37:11	1.15
CEsr-5410000	0.66	52.85	13.23	39:00:43	1.54
CEsr-5422000	0.66	41.23	−11.66	36:17:31	−1.18
CEsr-4265000	0.66	41.88	−10.26	35:13:05	−2.25
CEsr-6644000	0.66	46.27	−0.87	35:32:39	−1.93
CEsr-6642000	0.66	46.51	−0.36	37:51:41	0.39
CEsr-4254000	0.66	39.82	−14.69	39:40:40	2.21
CEsr-6625000	0.66	50.19	7.54	38:20:58	0.88
CEmr-2811100	0.66	46.07	−1.29	37:32:10	0.07
CEmr-2033500	0.66	51.51	10.37	38:45:53	1.30
CEmr-1813200	0.66	45.47	−2.58	39:50:42	2.38
CEmr-1540700	0.66	41.19	−11.75	34:31:55	−2.94
CEmr-1320400	0.66	47.74	2.28	36:27:18	−1.01
CEmr-2299400	0.66	46.60	−0.16	36:39:44	−0.81
CEmr-2397300	0.66	45.62	−2.27	35:51:30	−1.61
CEsr-5770000	0.66	52.96	13.48	39:47:11	2.32
CEsr-2916000	0.66	49.67	6.41	39:11:12	1.72
CEsr-2923000	0.66	51.46	10.25	37:57:02	0.48
CEsr-4948000	0.66	50.40	7.99	38:35:15	1.12
CEsr-7395000	0.66	49.33	5.68	36:28:11	−1.00
CEsr-1403000	0.66	48.89	4.75	39:50:06	2.37
CEsr-1442000	0.66	42.58	−8.76	37:24:46	−0.06
CEsr-5246000	0.66	53.07	13.70	38:21:15	0.88
CEmr-2401700	0.66	50.92	9.11	36:59:41	−0.47
CEmr-2874600	0.66	42.64	−8.64	40:18:53	2.85
CEmr-2092800	0.66	45.54	−2.44	38:03:00	0.58
CEsr-1413000	0.66	36.25	−22.34	35:39:16	−1.81
CEmr-2381100	0.66	49.98	7.08	38:04:41	0.61
CEsr-7400000	0.66	44.89	−3.82	37:50:35	0.37
CEsr-1462000	0.66	37.36	−19.95	38:01:20	0.55
CEmr-2093600	0.66	47.86	2.54	37:12:56	−0.25
CEsr-5223000	0.66	52.23	11.90	38:23:37	0.92
CEsr-1215000	0.66	46.09	−1.26	37:53:46	0.43
CEsr-1287000	0.66	47.52	1.82	37:45:03	0.28
CEsr-7195000	0.66	44.76	−4.11	37:54:10	0.43
CEmr-2382500	0.66	44.07	−5.58	37:41:41	0.23
CEsr-5231000	0.66	54.22	16.17	40:11:39	2.72
CEsr-7403000	0.66	49.33	5.70	38:31:56	1.06
CEmr-2890400	0.66	49.21	5.44	37:22:17	−0.10
CEsr-1680000	0.66	53.92	15.53	38:56:24	1.47
CEsr-2873000	0.66	46.80	0.27	36:12:46	−1.26
CEsr-7255000	0.66	52.70	12.92	37:37:45	0.16
CEsr-6770000	0.66	61.14	31.00	47:14:54	9.78
CEsr-2835000	0.66	48.18	3.22	37:53:21	0.42
CEsr-2851000	0.66	62.68	34.28	37:23:37	−0.08
CEsr-7333000	0.66	45.03	−3.52	38:52:06	1.40
CEsr-6778000	0.66	49.84	6.78	38:17:08	0.82
CEsr-4644000	0.66	54.80	17.40	39:46:02	2.30
C04 untreated mock-infected cells	0.856350614	183.90 ± 5.87	N.D	N.D	N.D
C04 untreated infected cells	0.86	38.23 ± 1.1	N.D	36:31:32	N.D
CEsr-3921000	0.86	53.65	40.33	47:08:49	10.62
CEmr-3153200	0.86	48.65	27.26	42:26:50	5.92
CEsr-5997000	0.86	48.75	27.51	42:22:44	5.85
CEsr-4082000	0.86	43.69	14.27	37:26:24	0.91
CEmr-2141200	0.86	44.90	17.45	38:43:59	2.21
CEmr-3135600	0.86	44.90	17.44	38:52:23	2.35
CEsr-4551000	0.86	49.32	29.00	42:12:31	5.68
CEsr-4850000	0.86	46.69	22.11	42:34:06	6.04
CEmr-3390400	0.86	50.00	30.79	41:39:57	5.14
CEmr-3354200	0.86	51.00	33.41	40:53:59	4.37
CEsr-6645000	0.86	53.53	40.01	41:06:07	4.58
CEmr-2983500	0.86	55.36	44.79	43:47:42	7.27
CEmr-3353000	0.86	49.22	28.75	41:11:19	4.66
CEmr-3351300	0.86	51.73	35.30	44:49:39	8.30
CEmr-2038000	0.86	47.17	23.39	39:24:32	2.88
CEsr-7421000	0.86	49.28	28.91	43:27:48	6.94
CEsr-2351000	0.86	48.92	27.95	42:22:58	5.86
CEmr-1671600	0.86	53.16	39.04	45:19:45	8.80
CEmr-1678000	0.86	52.33	36.89	42:33:55	6.04
CEsr-2703000	0.86	52.29	36.77	42:15:48	5.74
CEsr-2350000	0.86	50.91	33.16	41:53:22	5.36
CEsr-7385000	0.86	50.82	32.92	41:37:20	5.10
CEmr-1679200	0.86	47.44	24.08	40:07:30	3.60
CEmr-2102700	0.86	46.27	21.04	41:23:45	4.87
CEmr-8245000	0.86	37.91	−0.84	39:30:51	2.99
CEsr-5622000	0.86	50.16	31.21	39:50:17	3.31
CEmr-1678100	0.86	48.64	27.22	40:36:55	4.09
CEsr-2620000	0.86	39.93	4.45	39:12:33	2.68
CEsr-2619000	0.86	53.69	40.42	42:20:42	5.82
CEsr-2971000	0.86	51.24	34.03	39:10:41	2.65
CEsr-7372000	0.86	45.34	18.58	39:58:23	3.45
CEmr-2340900	0.86	51.75	35.37	43:27:27	6.93
CEmr-1676700	0.86	41.39	8.27	40:59:16	4.46
CEsr-7383000	0.86	45.28	18.44	40:22:51	3.86
CEsr-5744000	0.86	49.05	28.31	43:13:36	6.70
CEsr-1817000	0.86	46.30	21.11	42:42:57	6.19
CEmr-2584100	0.86	49.17	28.61	42:37:00	6.09
CEmr-2391500	0.86	52.73	37.93	41:50:52	5.32
CEmr-2391600	0.86	45.49	18.98	38:46:45	2.25
CEsr-1520000	0.86	55.51	45.18	44:34:34	8.05
CEsr-1721000	0.86	41.45	8.41	38:24:09	1.88
CEmr-1448100	0.86	45.97	20.25	45:13:19	8.70
CEsr-3830000	0.86	42.24	10.48	39:14:33	2.72
CEsr-1528000	0.86	43.19	12.96	38:47:05	2.26
CEsr-5508000	0.86	45.56	19.16	40:39:07	4.13
CEmr-2506800	0.86	51.98	35.97	44:55:16	8.40
CEsr-1564000	0.86	45.91	20.08	39:37:06	3.09
CEsr-5031000	0.86	47.79	25.01	41:19:03	4.79
CEsr-4914000	0.86	44.25	15.73	39:01:50	2.50
CEsr-3906000	0.86	45.06	17.87	40:49:04	4.29
CEsr-2175000	0.86	36.52	−4.47	37:44:04	1.21
CEsr-1627000	0.86	59.88	56.64	45:37:34	9.10
CEsr-3936000	0.86	45.30	18.49	40:22:46	3.85
CEsr-3947000	0.86	45.06	17.87	40:54:53	4.39
CEmr-2231700	0.86	51.44	34.54	43:46:04	7.24
CEmr-2501700	0.86	44.65	16.79	41:17:54	4.77
CEsr-3929000	0.86	41.66	8.98	39:40:27	3.15
CEsr-1639000	0.86	51.25	34.05	39:57:28	3.43
CEsr-1601000	0.86	50.22	31.36	40:18:26	3.78
CEmr-1632500	0.86	40.14	5.00	38:23:53	1.87
CEsr-5377000	0.86	42.86	12.11	38:28:20	1.95
CEmr-1607500	0.86	48.73	27.45	42:09:26	5.63
CEsr-1646000	0.86	47.45	24.11	39:43:48	3.20
CEsr-2301000	0.86	45.53	19.10	38:58:53	2.46
CEmr-2503300	0.86	61.15	59.94	51:04:54	14.56
CEmr-2036400	0.86	47.80	25.03	40:55:02	4.39
CEmr-1389700	0.86	39.00	2.01	37:11:27	0.67
CEsr-1161000	0.86	47.80	25.02	42:07:58	5.61
CEmr-3189500	0.86	29.91	−21.76	8:39:22	−27.87
CEmr-2810300	0.86	42.07	10.03	39:29:02	2.96
CEsr-6659000	0.86	47.40	23.98	45:24:39	8.89
CEsr-1143000	0.86	42.46	11.06	40:57:04	4.43
CEmr-1810200	0.86	35.65	−6.76	38:55:11	2.39
CEmr-2870600	0.86	48.24	26.18	42:32:48	6.02
CEmr-1713700	0.86	47.09	23.18	40:01:55	3.51
CEsr-1119000	0.86	46.77	22.33	42:49:44	6.30
CEmr-3220400	0.86	40.31	5.43	35:43:46	−0.80
CEsr-7431000	0.86	44.45	16.26	42:13:40	5.70
CEsr-1158000	0.86	45.34	18.60	42:30:42	5.99
CEsr-6547000	0.86	40.05	4.76	41:01:25	4.50
C05 untreated mock-infected cells	0.814647679	190.37 ± 6.24	N.D	N.D	N.D
C05 untreated infected cells	0.81	52.68 ± 2.27	N.D	38:40:55	N.D
CEsr-7478000	0.81	43.45	−17.52	40:50:13	2.16
CEmr-2137600	0.81	47.37	−10.07	39:08:42	0.46
CEsr-7531000	0.81	50.61	−3.93	42:19:23	3.64
CEmr-2981200	0.81	52.26	−0.79	43:33:29	4.88
CEsr-7420000	0.81	45.42	−13.78	42:41:08	4.00
CEsr-2607000	0.81	47.14	−10.50	40:34:50	1.90
CEsr-5019000	0.81	39.07	−25.83	38:03:05	−0.63
CEmr-3012200	0.81	46.74	−11.27	41:16:08	2.59
CEsr-5363000	0.81	34.85	−33.84	40:16:43	1.60
CEmr-3110000	0.81	52.31	−0.70	42:11:19	3.51
CEmr-3016600	0.81	43.23	−17.94	41:16:24	2.59
CEmr-3011300	0.81	46.12	−12.45	41:58:21	3.29
CEsr-5691000	0.81	55.28	4.95	41:09:39	2.48
CEsr-7469000	0.81	36.76	−30.21	36:45:31	−1.92
CEmr-2532800	0.81	59.59	13.13	42:44:53	4.07
CEmr-2932100	0.81	52.04	−1.21	43:07:16	4.44
CEmr-1983400	0.81	58.20	10.49	41:49:19	3.14
CEsr-4480000	0.81	46.65	−11.44	39:37:49	0.95
CEsr-6264000	0.81	43.60	−17.23	39:54:00	1.22
CEmr-2986800	0.81	40.81	−22.53	39:49:08	1.14
CEmr-2574400	0.81	50.93	−3.31	42:08:24	3.46
CEsr-4623000	0.81	50.10	−4.89	43:05:12	4.40
CEsr-4498000	0.81	47.21	−10.38	41:56:20	3.26
CEmr-1272800	0.81	52.78	0.19	40:22:14	1.69
CEsr-4345000	0.81	50.85	−3.47	41:22:24	2.69
CEmr-2135800	0.81	46.47	−11.78	42:07:45	3.45
CEsr-4433000	0.81	45.71	−13.23	38:30:59	−0.17
CEsr-4348000	0.81	47.31	−10.18	39:08:33	0.46
CEsr-4489000	0.81	54.28	3.04	44:26:12	5.75
CEsr-4634000	0.81	47.40	−10.02	39:26:42	0.76
CEsr-6064000	0.81	48.86	−7.24	41:29:23	2.81
CEsr-4314000	0.81	44.88	−14.81	40:12:47	1.53
CEsr-1424000	0.81	55.19	4.78	42:25:23	3.74
CEsr-2119000	0.81	47.63	−9.59	40:04:38	1.40
CEmr-2291200	0.81	50.02	−5.03	42:11:02	3.50
CEsr-4077000	0.81	49.16	−6.67	39:21:27	0.68
CEsr-1634000	0.81	52.59	−0.16	39:47:57	1.12
CEsr-6375000	0.81	53.93	2.38	42:03:11	3.37
CEsr-2624000	0.81	50.28	−4.55	41:15:17	2.57
CEmr-1676900	0.81	44.73	−15.09	39:16:57	0.60
CEsr-1551000	0.81	75.34	43.03	50:57:39	12.28
CEmr-1387900	0.81	54.39	3.26	42:13:18	3.54
CEmr-2585900	0.81	50.11	−4.88	38:57:21	0.27
CEsr-3176000	0.81	45.17	−14.25	42:15:31	3.58
CEsr-3663000	0.81	51.58	−2.08	40:41:25	2.01
CEmr-2783400	0.81	49.42	−6.17	40:25:07	1.74
CEmr-1683000	0.81	50.13	−4.83	42:13:41	3.55
CEmr-1275000	0.81	51.69	−1.87	41:30:08	2.82
CEmr-1332600	0.81	53.25	1.09	42:58:44	4.30
CEsr-6289000	0.81	52.09	−1.11	41:28:29	2.79
CEmr-2108600	0.81	48.21	−8.48	45:15:02	6.57
CEmr-2326000	0.81	49.28	−6.44	42:56:02	4.25
CEmr-2290300	0.81	48.75	−7.45	42:47:53	4.12
CEmr-1274800	0.81	53.36	1.30	41:50:21	3.16
CEmr-hm71700	0.81	50.44	−4.24	41:18:44	2.63
CEsr-2574000	0.81	55.21	4.81	42:15:47	3.58
CEsr-5486000	0.81	46.10	−12.47	39:06:22	0.42
CEsr-6065000	0.81	38.71	−26.51	37:51:29	−0.82
CEsr-2191000	0.81	41.05	−22.07	39:59:17	1.31
CEsr-4401000	0.81	42.77	−18.80	38:29:12	−0.20
CEsr-2125000	0.81	45.65	−13.33	39:12:09	0.52
CEmr-1272000	0.81	46.91	−10.94	39:21:48	0.68
CEmr-1941300	0.81	50.29	−4.53	42:25:14	3.74
CEsr-7440000	0.81	43.02	−18.33	41:01:05	2.34
CEsr-1683000	0.81	47.99	−8.89	39:48:47	1.13
CEmr-1847200	0.81	46.39	−11.94	39:46:27	1.09
CEsr-1559000	0.81	40.61	−22.90	37:00:01	−1.68
CEmr-2587500	0.81	50.51	−4.11	41:08:44	2.46
CEsr-1642000	0.81	40.24	−23.61	38:05:14	−0.59
CEsr-7001000	0.81	62.01	17.71	40:56:34	2.26
CEsr-1685000	0.81	52.99	0.60	40:23:52	1.72
CEmr-3045200	0.81	55.21	4.81	43:35:23	4.91
CEsr-4081000	0.81	46.56	−11.61	43:32:32	4.86
CEsr-1654000	0.81	51.13	−2.93	41:19:30	2.64
CEsr-6358000	0.81	51.66	−1.93	42:09:45	3.48
CEsr-1965000	0.81	46.45	−11.83	40:13:58	1.55
CEsr-2050000	0.81	50.82	−3.52	43:28:33	4.79
CEmr-3015500	0.81	47.32	−10.17	38:43:58	0.05
CEsr-1770000	0.81	56.21	6.70	43:10:52	4.50
CEmr-2107500	0.81	50.93	−3.32	42:47:58	4.12
C06 untreated mock-infected cells	0.62595441	197.79 ± 14.68	N.D	N.D	N.D
C06 untreated infected cells	0.63	54.03 ± 3.24	N.D	41:55:32	N.D
CEsr-2407000	0.63	39.85	−26.25	39:06:04	−2.82
CEmr-2149000	0.63	48.13	−10.93	41:09:17	−0.77
CEmr-2362200	0.63	44.80	−17.09	38:45:04	−3.17
CEsr-7462000	0.63	51.00	−5.62	43:07:40	1.20
CEsr-2383000	0.63	46.01	−14.85	40:19:57	−1.59
CEmr-2969400	0.63	50.66	−6.25	42:13:07	0.29
CEsr-2402000	0.63	55.34	2.43	43:09:04	1.23
CEsr-2638000	0.63	46.27	−14.38	39:37:00	−2.31
CEsr-5553000	0.63	45.45	−15.89	38:53:12	−3.04
CEmr-2980400	0.63	51.12	−5.40	40:41:55	−1.23
CEmr-3021400	0.63	43.73	−19.08	39:29:23	−2.44
CEsr-7360000	0.63	44.48	−17.68	40:52:59	−1.04
CEsr-3043000	0.63	44.75	−17.19	42:09:16	0.23
CEsr-4993000	0.63	42.95	−20.51	40:13:03	−1.71
CEsr-7373000	0.63	47.49	−12.11	40:15:00	−1.68
CEsr-7369000	0.63	46.64	−13.68	39:35:18	−2.34
CEsr-3046000	0.63	43.23	−20.00	39:37:09	−2.31
CEsr-3151000	0.63	40.78	−24.52	38:45:04	−3.17
CEsr-2956000	0.63	48.54	−10.17	39:29:16	−2.44
CEsr-7386000	0.63	42.47	−21.40	39:45:41	−2.16
CEmr-3043000	0.63	47.07	−12.88	41:58:24	0.05
CEmr-3046100	0.63	44.86	−16.98	41:25:39	−0.50
CEsr-7352000	0.63	47.93	−11.29	41:58:55	0.06
CEmr-2364400	0.63	46.28	−14.35	40:08:49	−1.78
CEsr-1122000	0.63	47.88	−11.39	40:10:04	−1.76
CEsr-5821000	0.63	46.55	−13.85	38:53:20	−3.04
CEmr-3092500	0.63	42.81	−20.77	39:27:35	−2.47
CEsr-6995000	0.63	46.36	−14.20	41:01:49	−0.90
CEsr-1167000	0.63	44.06	−18.46	41:12:41	−0.71
CEsr-5286000	0.63	43.39	−19.70	38:46:14	−3.15
CEsr-5287000	0.63	45.79	−15.26	37:13:53	−4.69
CEmr-1891100	0.63	43.80	−18.94	39:15:07	−2.67
CEsr-6827000	0.63	48.78	−9.72	41:59:03	0.06
CEmr-3224300	0.63	35.52	−34.26	41:08:21	−0.79
CEmr-3391700	0.63	47.34	−12.39	39:56:27	−1.98
CEmr-1870000	0.63	42.40	−21.52	39:53:35	−2.03
CEmr-2701200	0.63	40.70	−24.67	38:55:03	−3.01
CEmr-3017800	0.63	50.66	−6.24	42:48:05	0.88
CEmr-3221000	0.63	46.89	−13.22	41:05:30	−0.83
CEsr-5952000	0.63	38.12	−29.46	36:54:30	−5.02
CEsr-5646000	0.63	47.57	−11.97	39:57:16	−1.97
CEsr-7036000	0.63	45.81	−15.22	40:25:39	−1.50
CEsr-5021000	0.63	38.23	−29.24	38:43:46	−3.20
CEmr-2532500	0.63	45.70	−15.41	42:17:39	0.37
CEsr-5707000	0.63	36.02	−33.33	36:35:47	−5.33
CEsr-5777000	0.63	42.71	−20.95	41:31:44	−0.40
CEmr-1414100	0.63	46.19	−14.51	40:34:32	−1.35
CEsr-6619000	0.63	48.40	−10.43	43:05:02	1.16
CEsr-1750000	0.63	44.47	−17.69	40:06:08	−1.82
CEmr-1842900	0.63	43.50	−19.50	38:39:19	−3.27
CEmr-2283500	0.63	35.82	−33.71	36:58:49	−4.95
CEsr-7077000	0.63	39.84	−26.27	39:52:22	−2.05
CEsr-7052000	0.63	42.40	−21.53	40:59:57	−0.93
CEmr-2398200	0.63	50.59	−6.37	43:25:58	1.51
CEsr-7062000	0.63	46.19	−14.52	42:08:35	0.22
CEsr-1839000	0.63	45.73	−15.36	39:09:07	−2.77
CEmr-2576800	0.63	37.53	−30.54	35:56:06	−5.99
CEmr-3187700	0.63	43.34	−19.79	37:47:15	−4.14
CEmr-2138200	0.63	44.38	−17.87	41:19:39	−0.60
CEmr-2585500	0.63	45.29	−16.18	40:10:40	−1.75
CEmr-3184300	0.63	36.37	−32.69	38:28:50	−3.44
CEmr-3183600	0.63	39.00	−27.81	41:02:29	−0.88
CEsr-7108000	0.63	53.14	−1.65	40:42:33	−1.22
CEsr-7025000	0.63	41.87	−22.50	38:40:46	−3.25
CEmr-2107200	0.63	42.96	−20.50	39:58:44	−1.95
CEsr-6947000	0.63	50.45	−6.63	41:26:53	−0.48
CEsr-7466000	0.63	36.44	−32.56	36:41:40	−5.23
CEmr-1981400	0.63	47.21	−12.63	41:16:38	−0.65
CEsr-7010000	0.63	45.80	−15.24	41:09:23	−0.77
CEmr-2794900	0.63	44.48	−17.67	38:00:35	−3.92
CEsr-2968000	0.63	46.22	−14.47	39:56:24	−1.99
CEsr-7518000	0.63	42.18	−21.93	38:10:01	−3.76
CEmr-1617300	0.63	43.28	−19.90	39:41:21	−2.24
CEsr-6955000	0.63	50.13	−7.23	41:24:37	−0.52
CEmr-3224400	0.63	41.50	−23.19	39:22:13	−2.56
CEmr-1473700	0.63	44.39	−17.84	39:05:59	−2.83
CEsr-1999000	0.63	45.73	−15.37	38:16:14	−3.65
CEmr-1843100	0.63	39.85	−26.25	36:57:38	−4.96
CEsr-7279000	0.63	39.04	−27.75	38:56:05	−2.99
CEmr-1554200	0.63	50.63	−6.31	45:46:31	3.85
C07 untreated mock-infected cells	0.8693	186.41 ± 4.44	N.D	N.D	N.D
C07 untreated infected cells	0.87	54.55 ± 1.31	N.D	37:21:37	N.D
CEsr-6091000	0.87	54.52	−0.07	38:15:35	0.90
CEmr-1371700	0.87	53.27	−2.35	40:43:23	3.36
CEsr-6362000	0.87	71.33	30.76	49:16:16	11.91
CEmr-2232500	0.87	50.23	−7.93	39:06:13	1.74
CEsr-4565000	0.87	47.08	−13.71	38:21:00	0.99
CEmr-1711900	0.87	57.05	4.57	38:44:45	1.39
CEsr-4278000	0.87	54.73	0.32	38:17:42	0.93
CEsr-2077000	0.87	52.57	−3.64	36:16:23	−1.09
CEmr-2532400	0.87	54.48	−0.14	39:38:12	2.28
CEsr-4279000	0.87	54.44	−0.21	38:46:34	1.42
CEsr-2053000	0.87	36.07	−33.88	35:10:09	−2.19
CEsr-2112000	0.87	47.93	−12.15	40:15:18	2.89
CEsr-2054000	0.87	50.04	−8.28	37:47:24	0.43
CEsr-6966000	0.87	58.74	7.67	37:13:52	−0.13
CEsr-1673000	0.87	50.72	−7.02	37:26:44	0.09
CEsr-4259000	0.87	50.13	−8.11	37:13:57	−0.13
CEsr-5812000	0.87	46.47	−14.82	36:59:54	−0.36
CEmr-2501800	0.87	63.41	16.23	44:06:14	6.74
CEmr-2610600	0.87	52.58	−3.62	38:51:26	1.50
CEsr-2142000	0.87	47.41	−13.10	40:16:07	2.91
CEmr-1931700	0.87	51.45	−5.69	37:11:10	−0.17
CEsr-1674000	0.87	54.00	−1.02	39:43:22	2.36
CEsr-2096000	0.87	52.37	−4.00	39:06:08	1.74
CEsr-5182000	0.87	48.39	−11.30	38:43:59	1.37
CEmr-1675400	0.87	49.48	−9.31	38:14:46	0.89
CEmr-2986100	0.87	47.05	−13.75	40:54:54	3.55
CEmr-2555000	0.87	51.92	−4.83	38:20:33	0.98
CEsr-7290000	0.87	56.89	4.29	38:31:13	1.16
CEsr-7574000	0.87	50.29	−7.81	41:14:30	3.88
CEmr-2890700	0.87	32.02	−41.31	38:19:16	0.96
CEmr-2891500	0.87	63.03	15.53	41:29:24	4.13
CEmr-3073800	0.87	52.34	−4.06	38:46:02	1.41
CEmr-3080800	0.87	52.21	−4.30	38:08:51	0.79
CEmr-2600500	0.87	52.00	−4.68	39:10:57	1.82
CEmr-2329600	0.87	54.55	−0.01	42:08:46	4.79
CEsr-5924000	0.87	52.35	−4.03	38:24:44	1.05
CEsr-1605000	0.87	46.51	−14.75	35:53:29	−1.47
CEsr-2251000	0.87	48.58	−10.95	38:51:28	1.50
CEmr-3188000	0.87	50.43	−7.55	38:25:06	1.06
CEsr-5800000	0.87	50.65	−7.16	36:58:47	−0.38
CEmr-2875900	0.87	48.22	−11.60	36:57:09	−0.41
CEsr-5485000	0.87	50.98	−6.56	37:19:48	−0.03
CEmr-2876200	0.87	50.15	−8.08	37:32:21	0.18
CEpa-3	0.87	61.78	13.25	45:09:08	7.79
CEmr-2877100	0.87	60.59	11.07	40:37:43	3.27
CEsr-1591000	0.87	54.47	−0.16	38:44:08	1.38
CEsr-6546000	0.87	52.06	−4.57	39:15:23	1.90
CEsr-1612000	0.87	58.79	7.76	39:05:57	1.74
CEsr-1632000	0.87	59.80	9.62	38:31:56	1.17
CEmr-2664700	0.87	50.25	−7.88	40:39:34	3.30
CEmr-2876100	0.87	50.06	−8.24	39:27:44	2.10
CEsr-5543000	0.87	60.12	10.21	41:22:56	4.02
CEsr-2265000	0.87	50.52	−7.40	39:49:52	2.47
CEsr-5517000	0.87	56.92	4.34	38:41:51	1.34
CEsr-5561000	0.87	53.45	−2.02	38:48:25	1.45
CEsr-5571000	0.87	76.38	40.01	40:39:59	3.31
CEmr-2031900	0.87	53.44	−2.05	40:46:12	3.41
CEsr-3966000	0.87	45.73	−16.18	38:22:38	1.02
CEsr-5837000	0.87	45.96	−15.76	41:12:46	3.85
CEmr-1947400	0.87	52.94	−2.97	37:23:30	0.03
CEsr-4980000	0.87	56.92	4.33	38:29:32	1.13
CEsr-3585000	0.87	55.80	2.29	38:18:45	0.95
CEsr-2285000	0.87	50.78	−6.92	36:27:39	−0.90
CEsr-5343000	0.87	51.42	−5.74	37:35:25	0.23
CEsr-5347000	0.87	44.18	−19.02	37:33:05	0.19
CEmr-1985700	0.87	51.49	−5.61	39:56:11	2.58
CEmr-1278100	0.87	52.81	−3.19	39:39:20	2.30
CEsr-5353000	0.87	54.64	0.16	38:01:02	0.66
CEsr-7480000	0.87	49.45	−9.35	37:39:52	0.30
CEmr-1075400	0.87	55.98	2.62	37:21:53	0.00
CEsr-3597000	0.87	54.65	0.18	39:34:36	2.22
CEmr-6630000	0.87	55.88	2.42	38:03:21	0.70
CEsr-1827000	0.87	62.14	13.91	37:21:43	0.00
CEsr-4592000	0.87	50.56	−7.31	37:32:21	0.18
CEsr-5268000	0.87	48.90	−10.37	37:17:07	−0.08
CEsr-1169000	0.87	47.22	−13.45	37:05:20	−0.27
CEsr-7445000	0.87	56.35	3.30	38:22:40	1.02
CEmr-2587000	0.87	58.29	6.85	39:24:55	2.05
CEmr-2985600	0.87	51.16	−6.22	38:13:29	0.86
CEmr-2386800	0.87	54.23	−0.59	38:03:33	0.70
C08 untreated mock-infected cells	0.74	156.52 ± 8.90	N.D	N.D	N.D
C08 untreated infected cells	0.74	49.02 ± 0.34	N.D	39:02:13	N.D
CEsr-2888000	0.74	54.76	11.70	40:43:52	1.69
CEsr-7038000	0.74	56.52	15.28	39:34:10	0.53
CEsr-2870000	0.74	49.61	1.20	39:45:25	0.72
CEsr-1682000	0.74	49.47	0.92	38:52:54	−0.16
CEsr-4845000	0.74	49.10	0.17	40:10:23	1.14
CEsr-4773000	0.74	49.15	0.26	39:19:26	0.29
CEsr-6768000	0.74	47.39	−3.34	38:26:43	−0.59
CEsr-1670000	0.74	54.61	11.41	39:17:33	0.26
CEmr-1621200	0.74	50.35	2.71	38:55:13	−0.12
CEsr-7191000	0.74	49.41	0.80	37:37:45	−1.41
CEsr-6878000	0.74	45.81	−6.54	38:17:48	−0.74
CEsr-6024000	0.74	47.50	−3.11	37:29:45	−1.54
CEsr-6523000	0.74	46.38	−5.38	37:14:06	−1.80
CEmr-1846000	0.74	46.81	−4.51	38:18:22	−0.73
CEmr-1322600	0.74	51.95	5.97	37:00:11	−2.03
CEsr-6861000	0.74	56.90	16.08	42:57:40	3.92
CEsr-6912000	0.74	47.18	−3.76	35:30:25	−3.53
CEsr-6931000	0.74	49.17	0.30	36:56:00	−2.10
CEmr-2645700	0.74	44.01	−10.23	31:57:23	−7.08
CEsr-1300001	0.74	51.79	5.64	39:46:38	0.74
CEsr-6934000	0.74	50.59	3.21	40:07:31	1.09
CEsr-6017000	0.74	51.45	4.95	40:05:38	1.06
CEsr-1481000	0.74	50.19	2.38	38:33:23	−0.48
CEsr-2942000	0.74	46.34	−5.48	37:29:52	−1.54
CEsr-6928000	0.74	46.85	−4.44	36:32:46	−2.49
CEmr-1997000	0.74	48.47	−1.14	36:39:54	−2.37
CEmr-2704600	0.74	46.38	−5.38	38:19:59	−0.70
CEsr-5527000	0.74	50.73	3.49	38:56:47	−0.09
CEmr-1561400	0.74	53.15	8.43	40:09:11	1.12
CEsr-4828000	0.74	48.29	−1.49	38:39:48	−0.37
CEmr-2580500	0.74	47.79	−2.51	40:20:45	1.31
CEsr-5326000	0.74	43.45	−11.36	39:46:03	0.73
CEsr-4830000	0.74	53.29	8.70	38:34:08	−0.47
CEsr-4836000	0.74	46.73	−4.67	38:10:11	−0.87
CEmr-1216000	0.74	51.31	4.67	38:29:48	−0.54
CEmr-2874400	0.74	42.17	−13.98	39:23:15	0.35
CEsr-4838000	0.74	47.73	−2.64	36:00:08	−3.03
CEsr-4809000	0.74	48.60	−0.86	36:57:05	−2.09
CEsr-4833000	0.74	47.81	−2.48	38:30:05	−0.54
CEsr-1414000	0.74	41.55	−15.25	35:08:44	−3.89
CEmr-2637900	0.74	46.73	−4.67	38:03:05	−0.99
CEmr-3052600	0.74	56.89	16.05	38:22:00	−0.67
CEmr-3066000	0.74	47.64	−2.82	38:30:35	−0.53
CEmr-2282300	0.74	52.95	8.00	35:34:22	−3.46
CEmr-3065800	0.74	53.92	9.99	39:40:36	0.64
CEmr-9479000	0.74	58.68	19.70	38:48:53	−0.22
CEsr-2184000	0.74	46.53	−5.08	36:45:27	−2.28
CEmr-1843500	0.74	47.51	−3.09	37:36:24	−1.43
CEmr-3065200	0.74	55.76	13.75	39:51:59	0.83
CEsr-6622000	0.74	46.23	−5.70	36:23:22	−2.65
CEmr-2323700	0.74	45.53	−7.13	37:45:47	−1.27
CEmr-3091000	0.74	46.05	−6.06	37:49:48	−1.21
CEmr-3065400	0.74	56.33	14.90	40:50:06	1.80
CEsr-4221000	0.74	45.84	−6.49	39:17:28	0.25
CEsr-7417000	0.74	43.01	−12.27	37:18:09	−1.73
CEsr-4260000	0.74	50.13	2.25	40:42:58	1.68
CEsr-6121000	0.74	49.28	0.53	38:28:06	−0.57
CEsr-2151000	0.74	40.66	−17.07	37:39:32	−1.38
CEsr-6852000	0.74	48.03	−2.03	37:04:57	−1.95
CEsr-4222000	0.74	47.12	−3.89	37:43:14	−1.32
CEsr-1079000	0.74	49.09	0.13	38:06:25	−0.93
CEsr-4251000	0.74	47.31	−3.49	45:11:38	6.16
CEsr-2207000	0.74	44.70	−8.83	36:59:11	−2.05
CEsr-1004000	0.74	43.69	−10.87	36:38:29	−2.40
CEmr-3064200	0.74	54.21	10.59	38:46:37	−0.26
CEsr-6131000	0.74	51.89	5.85	39:17:32	0.26
CEmr-2985200	0.74	46.64	−4.86	36:34:01	−2.47
CEmr-1458701	0.74	53.38	8.89	39:55:06	0.88
CEmr-2986600	0.74	48.41	−1.26	39:10:09	0.13
CEmr-2575600	0.74	51.66	5.39	38:51:23	−0.18
CEsr-3379000	0.74	42.15	−14.01	39:13:20	0.19
CEmr-3098500	0.74	40.18	−18.05	36:43:59	−2.30
CEsr-6394000	0.74	49.06	0.08	38:14:34	−0.79
CEmr-2636600	0.74	55.21	12.62	39:30:54	0.48
CEsr-2642000	0.74	44.42	−9.39	38:24:56	−0.62
CEsr-1550000	0.74	55.45	13.11	40:29:16	1.45
CEsr-5667000	0.74	46.72	−4.70	36:37:56	−2.40
CEsr-2195000	0.74	53.68	9.50	39:46:02	0.73
CEsr-5709000	0.74	46.80	−4.53	37:12:32	−1.83
CEmr-2286700	0.74	56.25	14.75	38:24:24	−0.63
C09 untreated mock-infected cells	0.836	160.87 ± 5.24	N.D	N.D	N.D
C09 untreated infected cells	0.84	51.12 ± 0.76	N.D	39:10:05	N.D
CEmr-1502800	0.84	59.93	17.22	41:40:55	2.51
CEsr-4507000	0.84	56.64	10.80	39:21:05	0.18
CEmr-2328000	0.84	55.56	8.67	37:23:47	−1.77
CEsr-4402000	0.84	49.03	−4.09	38:00:16	−1.16
CEsr-7399000	0.84	56.01	9.56	39:04:40	−0.09
CEsr-4407000	0.84	59.33	16.05	39:22:24	0.21
CEmr-2392700	0.84	55.31	8.18	39:03:20	−0.11
CEmr-2392500	0.84	66.93	30.92	41:07:48	1.96
CEmr-1502600	0.84	59.22	15.84	41:17:27	2.12
CEmr-2108200	0.84	49.76	−2.67	38:02:49	−1.12
CEsr-2058000	0.84	53.02	3.71	39:12:39	0.04
CEmr-1508100	0.84	49.87	−2.45	36:39:05	−2.52
CEsr-4626000	0.84	52.71	3.10	40:22:30	1.21
CEsr-3343000	0.84	56.59	10.69	37:16:44	−1.89
CEmr-1503400	0.84	63.61	24.43	40:37:00	1.45
CEmr-2323100	0.84	54.31	6.23	36:38:05	−2.53
CEmr-2109800	0.84	53.82	5.28	36:53:10	−2.28
CEpa-5	0.84	53.18	4.01	37:38:32	−1.53
CEmr-3393400	0.84	55.09	7.76	39:00:23	−0.16
CEsr-2445000	0.84	53.94	5.51	38:41:31	−0.48
CEmr-1446400	0.84	50.87	−0.50	37:09:29	−2.01
CEsr-2492000	0.84	56.12	9.77	38:38:49	−0.52
CEsr-4689000	0.84	54.58	6.76	38:45:48	−0.40
CEsr-5048000	0.84	41.23	−19.36	38:47:49	−0.37
CEsr-2520000	0.84	60.04	17.44	38:45:13	−0.41
CEmr-1442700	0.84	53.60	4.85	37:41:21	−1.48
CEsr-3475000	0.84	52.15	2.01	39:52:56	0.71
CEmr-2037900	0.84	54.63	6.86	39:53:23	0.72
CEsr-3965000	0.84	50.34	−1.53	36:36:53	−2.55
CEsr-5078000	0.84	57.98	13.41	38:32:47	−0.62
CEmr-2244800	0.84	58.54	14.50	45:21:07	6.18
CEsr-7088000	0.84	25.14	−50.83	11:46:39	−27.39
CEsr-3953000	0.84	49.35	−3.47	35:05:14	−4.08
CEmr-1385100	0.84	53.54	4.73	40:02:05	0.87
CEmr-2532100	0.84	58.32	14.08	42:45:06	3.58
CEsr-2477000	0.84	44.34	−13.27	38:27:44	−0.71
CEmr-3221400	0.84	68.45	33.88	42:30:47	3.34
CEsr-1054000	0.84	64.55	26.26	41:40:14	2.50
CEsr-5509000	0.84	55.84	9.22	39:46:36	0.61
CEmr-3063400	0.84	56.49	10.49	39:52:06	0.70
CEpa-13	0.84	47.96	−6.19	36:28:24	−2.69
CEmr-1571000	0.84	53.90	5.43	37:38:16	−1.53
CEmr-2247700	0.84	43.45	−15.01	38:23:18	−0.78
CEsr-2006000	0.84	50.83	−0.58	39:34:52	0.41
CEmr-3063700	0.84	55.62	8.79	49:02:11	9.87
CEmr-2530300	0.84	49.60	−2.99	38:55:04	−0.25
CEsr-6742000	0.84	49.53	−3.12	43:46:20	4.60
CEsr-2470000	0.84	57.52	12.51	39:57:58	0.80
CEmr-3222900	0.84	50.72	−0.80	39:09:25	−0.01
CEmr-1848500	0.84	49.99	−2.22	38:34:29	−0.59
CEsr-3172000	0.84	62.66	22.56	42:58:08	3.80
CEmr-2241300	0.84	53.18	4.03	41:40:03	2.50
CEsr-3958000	0.84	53.04	3.76	39:43:58	0.56
CEsr-2469000	0.84	60.42	18.18	39:58:39	0.81
CEmr-2364800	0.84	54.59	6.78	38:10:14	−1.00
CEsr-4863000	0.84	59.04	15.48	38:38:55	−0.52
CEmr-hm63500	0.84	59.03	15.46	38:45:38	−0.41
CEmr-1407200	0.84	57.47	12.41	39:13:34	0.06
CEsr-3587000	0.84	47.45	−7.19	35:50:11	−3.33
CEsr-3586000	0.84	59.20	15.79	42:55:16	3.75
CEmr-1671400	0.84	57.46	12.39	40:52:13	1.70
CEmr-2396200	0.84	50.43	−1.35	38:13:29	−0.94
CEmr-2700600	0.84	54.87	7.34	39:46:24	0.61
CEmr-1811900	0.84	55.66	8.88	39:43:47	0.56
CEsr-3659000	0.84	49.73	−2.72	38:01:56	−1.14
CEsr-4461000	0.84	62.04	21.34	38:38:45	−0.52
CEmr-2811300	0.84	55.62	8.80	36:37:45	−2.54
CEmr-2150000	0.84	59.03	15.47	40:18:34	1.14
CEsr-1868000	0.84	49.51	−3.16	37:19:22	−1.85
CEsr-1913000	0.84	61.42	20.14	36:47:15	−2.38
CEmr-1676100	0.84	59.19	15.79	39:15:01	0.08
CEmr-1502100	0.84	64.74	26.63	38:33:28	−0.61
CEsr-4590000	0.84	46.52	−9.00	37:26:46	−1.72
CEsr-4602000	0.84	54.09	5.80	40:04:53	0.91
CEsr-1865000	0.84	59.23	15.85	40:36:00	1.43
CEsr-1874000	0.84	56.21	9.96	40:26:00	1.27
CEmr-2391200	0.84	51.73	1.19	39:31:27	0.36
CEsr-1910000	0.84	64.01	25.21	38:53:02	−0.28
CEsr-1920000	0.84	53.61	4.85	42:26:39	3.28
CEmr-2396000	0.84	57.55	12.56	39:47:18	0.62
C10 untreated mock-infected cells	0.82	142.83 ± 4.21	N.D	N.D	N.D
C10 untreated infected cells	0.82	36.03 ± 2.22	N.D	34:21:36	N.D
CEmr-3183500	0.82	29.63	−17.77	36:35:51	1.20
CEsr-1385000	0.82	38.16	5.92	39:39:14	4.26
CEsr-1879000	0.82	36.42	1.09	39:16:29	3.88
CEsr-5583000	0.82	37.96	5.35	38:22:26	2.98
CEsr-2038000	0.82	40.27	11.78	38:51:42	3.47
CEmr-2881800	0.82	36.83	2.22	35:46:55	0.39
CEsr-2266000	0.82	35.99	−0.12	34:19:04	−1.08
CEsr-4083000	0.82	35.38	−1.80	34:24:14	−0.99
CEmr-2395800	0.82	32.54	−9.68	34:22:51	−1.02
CEmr-3187900	0.82	38.29	6.26	37:43:06	2.32
CEsr-1878000	0.82	38.60	7.13	35:36:08	0.21
CEmr-3188900	0.82	40.25	11.72	38:47:40	3.40
CEsr-4091000	0.82	37.50	4.09	34:30:57	−0.88
CEmr-1440100	0.82	39.95	10.88	37:07:44	1.73
CEsr-4106000	0.82	37.11	3.00	36:18:00	0.90
CEmr-2440400	0.82	34.62	−3.90	34:34:45	−0.82
CEmr-3370000	0.82	27.71	−23.09	34:39:18	−0.74
CEsr-4783000	0.82	36.99	2.67	37:10:56	1.79
CEmr-2574200	0.82	33.80	−6.18	36:01:56	0.64
CEmr-3119900	0.82	36.88	2.35	39:53:58	4.50
CEmr-2707900	0.82	38.46	6.74	37:27:27	2.06
CEmr-2612200	0.82	37.93	5.26	37:35:53	2.20
CEmr-1841900	0.82	37.02	2.74	35:52:19	0.48
CEmr-3265600	0.82	38.57	7.06	38:21:33	2.96
CEsr-1254000	0.82	32.27	−10.44	34:04:01	−1.33
CEsr-1711000	0.82	36.84	2.24	37:26:20	2.04
CEmr-3119000	0.82	33.88	−5.98	35:15:32	−0.14
CEpa-58	0.82	37.32	3.59	36:39:19	1.26
CEsr-6475000	0.82	32.61	−9.49	37:18:31	1.91
CEsr-6158000	0.82	35.29	−2.05	34:20:38	−1.05
CEpa-54	0.82	37.15	3.10	36:04:35	0.68
CEsr-6087000	0.82	37.32	3.58	36:50:52	1.45
CEsr-6515000	0.82	34.90	−3.13	34:59:50	−0.40
CEpa-45	0.82	39.19	8.78	36:00:00	0.60
CEsr-6298000	0.82	36.89	2.38	35:59:01	0.59
CEmr-2587700	0.82	39.96	10.90	36:21:13	0.96
CEsr-6494000	0.82	35.68	−0.97	36:01:43	0.63
CEsr-6255000	0.82	35.41	−1.73	34:07:02	−1.28
CEmr-2582900	0.82	42.90	19.05	38:37:21	3.23
CEmr-1843900	0.82	31.28	−13.18	33:19:09	−2.08
CEsr-3831000	0.82	33.61	−6.73	34:10:45	−1.22
CEmr-2399900	0.82	43.63	21.10	38:40:43	3.28
CEsr-4928000	0.82	35.11	−2.54	35:07:22	−0.27
CEmr-2381200	0.82	35.43	−1.66	33:05:09	−2.31
CEmr-1095900	0.82	35.75	−0.79	31:40:53	−3.72
CEmr-2090800	0.82	41.26	14.52	38:20:17	2.94
CEsr-4308000	0.82	33.50	−7.01	33:56:46	−1.45
CEmr-2301100	0.82	38.38	6.52	37:24:40	2.01
CEsr-1260000	0.82	39.14	8.63	38:06:48	2.72
CEmr-2385800	0.82	38.00	5.47	37:39:29	2.26
CEmr-2381500	0.82	40.05	11.16	38:40:07	3.27
CEmr-2383700	0.82	32.42	−10.01	34:46:01	−0.63
CEsr-4340000	0.82	37.05	2.82	34:44:53	−0.65
CEmr-1558200	0.82	41.12	14.12	37:14:07	1.84
CEsr-6129000	0.82	40.31	11.88	38:37:21	3.23
CEsr-7298000	0.82	37.87	5.12	35:35:33	0.20
CEmr-1400600	0.82	41.86	16.19	42:54:17	7.51
CEsr-2508000	0.82	38.70	7.40	37:26:12	2.04
CEmr-3180100	0.82	35.74	−0.81	42:19:09	6.92
CEmr-2550300	0.82	34.95	−3.00	34:58:37	−0.42
CEsr-7449000	0.82	42.53	18.03	40:26:59	5.05
CEmr-2388800	0.82	40.71	13.00	38:04:30	2.68
CEmr-1843800	0.82	39.40	9.35	38:28:43	3.08
CEsr-7446000	0.82	35.85	−0.50	36:54:35	1.51
CEmr-1632100	0.82	38.21	6.04	38:40:08	3.27
CEmr-2244700	0.82	40.69	12.94	39:17:26	3.89
CEsr-6047000	0.82	39.60	9.92	37:39:01	2.25
CEsr-2089000	0.82	37.58	4.31	38:26:39	3.05
CEmr-1733600	0.82	40.56	12.57	38:30:05	3.11
CEmr-3014200	0.82	39.01	8.27	38:02:04	2.64
CEsr-2587000	0.82	29.36	−18.50	29:37:51	−5.77
CEmr-8800000	0.82	25.27	−29.88	31:56:41	−3.45
CEsr-1068000	0.82	16.72	−53.61	29:28:03	−5.93
CEsr-4381000	0.82	38.63	7.21	39:57:47	4.57
CEsr-4393000	0.82	38.22	6.07	36:42:24	1.31
CEmr-2264900	0.82	36.27	0.65	35:41:36	0.30
CEmr-3096200	0.82	43.05	19.50	40:04:26	4.68
CEsr-4998000	0.82	39.95	10.89	36:44:23	1.34
CEsr-3107000	0.82	41.55	15.32	39:27:04	4.05
CEmr-3202200	0.82	40.76	13.14	37:22:11	1.97
C11 untreated mock-infected cells	0.78	154.01 ± 7.20	N.D	N.D	N.D
C11 untreated infected cells	0.78	39.72 ± 1.17	N.D	35:14:43	N.D
CEsr-5668000	0.78	35.60	−10.37	34:08:38	−3.63
CEsr-3001000	0.78	41.95	5.63	40:38:41	2.87
CEsr-3124000	0.78	27.53	−30.68	32:57:47	−4.81
CEsr-2983000	0.78	33.71	−15.12	36:57:00	−0.83
CEmr-1456000	0.78	38.65	−2.69	39:57:52	2.19
CEsr-4596000	0.78	34.54	−13.05	35:35:16	−2.19
CEmr-2394000	0.78	37.71	−5.07	38:36:20	0.83
CEsr-4103000	0.78	43.00	8.25	38:19:34	0.55
CEsr-3022000	0.78	37.14	−6.50	34:15:44	−3.51
CEsr-4652000	0.78	40.25	1.34	38:56:32	1.17
CEsr-3805000	0.78	37.84	−4.73	36:24:19	−1.37
CEsr-7041000	0.78	42.14	6.09	39:31:45	1.75
CEsr-2762000	0.78	40.93	3.04	37:51:17	0.08
CEsr-5204000	0.78	35.78	−9.91	36:28:06	−1.31
CEsr-3806000	0.78	35.14	−11.54	34:50:48	−2.93
CEsr-3853000	0.78	38.21	−3.79	36:16:50	−1.49
CEmr-1678600	0.78	40.20	1.20	36:53:51	−0.88
CEsr-7028000	0.78	40.63	2.28	37:33:29	−0.22
CEsr-5163000	0.78	30.19	−24.00	32:28:09	−5.31
CEsr-5130000	0.78	27.78	−30.06	31:50:42	−5.93
CEmr-2969300	0.78	39.44	−0.71	35:42:51	−2.06
CEsr-3896000	0.78	33.02	−16.86	37:27:16	−0.32
CEmr-2966300	0.78	37.77	−4.91	39:27:47	1.69
CEsr-1549000	0.78	35.37	−10.94	34:35:41	−3.18
CEsr-1456000	0.78	37.20	−6.35	36:16:59	−1.49
CEmr-2631900	0.78	35.26	−11.23	33:41:13	−4.09
CEsr-2798000	0.78	38.46	−3.18	33:42:23	−4.07
CEmr-3046200	0.78	38.91	−2.05	37:14:35	−0.53
CEsr-2804000	0.78	41.74	5.09	35:41:35	−2.08
CEsr-2709000	0.78	36.88	−7.15	36:02:22	−1.74
CEsr-3702000	0.78	36.60	−7.86	39:33:34	1.78
CEsr-1315000	0.78	33.69	−15.19	36:22:08	−1.41
CEsr-2875000	0.78	43.14	8.60	39:43:25	1.95
CEmr-2991300	0.78	39.94	0.56	35:49:40	−1.95
CEmr-2247200	0.78	30.14	−24.12	2:12:52	−35.56
CEsr-2779000	0.78	33.23	−16.35	34:33:20	−3.22
CEmr-3040000	0.78	36.38	−8.42	34:05:51	−3.68
CEsr-2868000	0.78	35.54	−10.52	35:53:18	−1.89
CEsr-1262000	0.78	39.38	−0.86	37:35:11	−0.19
CEmr-2989700	0.78	56.43	42.07	52:52:23	15.10
CEmr-3055700	0.78	47.85	20.47	51:38:39	13.87
CEsr-3346000	0.78	33.95	−14.53	32:48:25	−4.97
CEsr-1289000	0.78	38.35	−3.45	35:31:47	−2.25
CEmr-3059900	0.78	33.54	−15.55	34:30:33	−3.27
CEmr-1982700	0.78	39.32	−1.00	36:18:50	−1.46
CEsr-1405000	0.78	39.34	−0.96	37:08:34	−0.63
CEsr-1292000	0.78	34.61	−12.86	34:11:21	−3.59
CEpa-21	0.78	43.98	10.73	38:01:47	0.25
CEsr-2417000	0.78	37.33	−6.01	35:34:16	−2.20
CEmr-2361400	0.78	41.91	5.51	42:39:39	4.89
CEsr-7473000	0.78	31.11	−21.69	34:22:40	−3.40
CEmr-3018300	0.78	34.41	−13.36	34:51:26	−2.92
CEmr-2266400	0.78	37.48	−5.63	36:06:16	−1.67
CEsr-1825000	0.78	42.51	7.03	35:17:55	−2.48
CEmr-3012400	0.78	37.03	−6.78	37:24:49	−0.36
CEsr-6626000	0.78	33.47	−15.74	32:21:50	−5.41
CEpa-24	0.78	44.59	12.25	41:08:05	3.36
CEmr-3094000	0.78	31.64	−20.34	32:52:52	−4.89
CEmr-2660600	0.78	33.23	−16.35	33:45:06	−4.02
CEmr-3046600	0.78	37.75	−4.95	39:05:52	1.32
CEmr-3015100	0.78	36.61	−7.84	32:58:43	−4.80
CEsr-7065000	0.78	21.78	−45.17	11:58:06	−25.81
CEmr-3094600	0.78	40.89	2.95	39:41:51	1.92
CEsr-6949000	0.78	42.58	7.20	35:12:39	−2.56
CEmr-3013300	0.78	38.53	−2.99	35:40:17	−2.10
CEmr-2758000	0.78	44.53	12.10	39:20:27	1.57
CEsr-2224000	0.78	41.36	4.12	37:21:03	−0.42
CEmr-1501300	0.78	39.26	−1.15	36:55:34	−0.85
CEsr-2424000	0.78	41.56	4.64	36:55:32	−0.85
CEsr-2389000	0.78	35.95	−9.50	36:58:33	−0.80
CEsr-2635000	0.78	38.40	−3.32	39:59:15	2.21
CEsr-2426000	0.78	39.91	0.49	37:31:10	−0.26
CEsr-2420000	0.78	39.98	0.64	34:02:57	−3.73
CEsr-2401000	0.78	37.11	−6.58	35:40:57	−2.09
CEsr-5109000	0.78	38.62	−2.78	34:58:30	−2.80
CEsr-2380000	0.78	37.13	−6.52	33:23:08	−4.39
CEmr-2326200	0.78	37.70	−5.08	34:12:36	−3.57
CEsr-5176000	0.78	31.41	−20.91	34:27:48	−3.31
CEsr-1259000	0.78	41.08	3.43	39:01:33	1.25
CEmr-2889900	0.78	41.62	4.77	37:09:55	−0.61
C12 untreated mock-infected cells	0.82	134.02 ± 2.13	N.D	N.D	N.D
C12 untreated infected cells	0.82	39.26 ± 3.41	N.D	34:11:46	N.D
CEsr-5345000	0.82	41.14	4.80	38:19:04	−0.56
CEmr-3093500	0.82	34.32	−12.58	36:11:36	−2.69
CEmr-2049200	0.82	38.17	−2.78	39:36:44	0.73
CEsr-4970000	0.82	36.58	−6.82	36:22:09	−2.51
CEsr-3582000	0.82	40.54	3.26	37:30:27	−1.37
CEsr-6540000	0.82	40.33	2.73	38:29:21	−0.39
CEsr-3957000	0.82	37.96	−3.32	37:10:21	−1.71
CEsr-4615000	0.82	42.16	7.38	39:59:38	1.11
CEmr-1943400	0.82	39.65	0.99	34:42:08	−4.18
CEsr-5765000	0.82	45.63	16.21	39:17:31	0.41
CEsr-4022000	0.82	38.16	−2.81	37:12:22	−1.67
CEsr-3584000	0.82	39.75	1.26	34:59:22	−3.89
CEsr-1907000	0.82	45.97	17.08	38:33:21	−0.33
CEsr-3768000	0.82	43.35	10.42	38:50:25	−0.04
CEmr-1631300	0.82	38.64	−1.57	36:54:48	−1.97
CEmr-1831800	0.82	40.36	2.80	38:34:24	−0.31
CEsr-2960000	0.82	41.42	5.50	47:25:59	8.55
CEsr-3548000	0.82	33.54	−14.57	34:09:26	−4.72
CEmr-1271000	0.82	39.39	0.33	36:28:03	−2.41
CEsr-7345000	0.82	37.70	−3.98	38:13:36	−0.65
CEsr-2959000	0.82	40.81	3.95	41:33:16	2.67
CEsr-7293000	0.82	36.89	−6.05	37:36:30	−1.27
CEsr-2901000	0.82	39.02	−0.62	35:18:22	−3.57
CEsr-7348000	0.82	35.89	−8.57	35:38:07	−3.25
CEsr-2936000	0.82	37.26	−5.08	35:10:17	−3.71
CEsr-3549000	0.82	37.30	−4.99	37:59:20	−0.89
CEsr-2248000	0.82	36.62	−6.73	33:52:10	−5.01
CEsr-5820000	0.82	43.30	10.29	42:10:23	3.29
CEsr-2974000	0.82	38.33	−2.36	34:00:54	−4.87
CEmr-2023800	0.82	34.83	−11.29	37:10:23	−1.71
CEsr-2479000	0.82	38.18	−2.75	36:04:43	−2.80
CEsr-7461000	0.82	39.91	1.67	37:52:28	−1.01
CEsr-6077000	0.82	41.92	6.78	38:12:21	−0.68
CEsr-5856000	0.82	38.09	−2.98	34:31:33	−4.36
CEsr-2488000	0.82	33.21	−15.42	33:49:20	−5.06
CEmr-2028200	0.82	37.96	−3.30	35:00:15	−3.88
CEsr-6162000	0.82	50.25	28.01	41:24:16	2.52
CEsr-2501000	0.82	36.98	−5.82	36:31:39	−2.35
CEsr-2159000	0.82	41.57	5.88	35:16:56	−3.60
CEsr-1940000	0.82	35.21	−10.31	37:25:57	−1.45
CEsr-5861000	0.82	39.63	0.94	39:01:26	0.14
CEsr-2490000	0.82	37.41	−4.72	33:39:29	−5.22
CEsr-7419000	0.82	36.41	−7.26	37:01:46	−1.85
CEsr-5862000	0.82	38.23	−2.62	36:23:42	−2.49
CEmr-2559000	0.82	32.89	−16.22	32:57:50	−5.92
CEsr-6658000	0.82	36.34	−7.45	36:40:07	−2.21
CEsr-5580000	0.82	36.51	−7.02	35:17:20	−3.59
CEsr-4981000	0.82	45.68	16.35	42:04:53	3.20
CEsr-2735000	0.82	44.16	12.48	39:44:27	0.86
CEsr-5817000	0.82	41.09	4.65	35:47:12	−3.09
CEsr-3235	0.82	35.64	−9.21	36:58:49	−1.90
CEsr-3265000	0.82	38.83	−1.08	35:14:37	−3.64
CEmr-1415000	0.82	42.29	7.71	36:01:14	−2.86
CEsr-1361000	0.82	39.88	1.57	36:14:29	−2.64
CEsr-2724000	0.82	38.55	−1.82	35:12:32	−3.67
CEsr-2579000	0.82	29.82	−24.05	37:47:15	−1.09
CEsr-2546000	0.82	40.60	3.41	37:05:01	−1.80
CEsr-3257000	0.82	38.30	−2.44	36:19:40	−2.55
CEsr-6195000	0.82	36.70	−6.51	40:22:23	1.49
CEsr-7101000	0.82	40.78	3.88	34:48:23	−4.07
CEsr-3736000	0.82	41.06	4.59	35:59:33	−2.89
CEsr-3248000	0.82	42.54	8.36	36:58:37	−1.90
CEsr-3259000	0.82	38.88	−0.96	36:00:26	−2.87
CEsr-1398000	0.82	37.46	−4.58	40:02:41	1.16
CEsr-3438000	0.82	39.86	1.52	37:52:54	−1.00
CEsr-3119000	0.82	38.31	−2.41	36:27:10	−2.43
CEmr-2702400	0.82	40.48	3.12	36:44:26	−2.14
CEmr-3170200	0.82	48.56	23.70	44:00:24	5.13
CEsr-5727000	0.82	37.44	−4.65	37:20:14	−1.54
CEsr-2790000	0.82	38.74	−1.33	36:22:49	−2.50
CEsr-2834000	0.82	37.83	−3.64	35:57:42	−2.92
CEsr-2739000	0.82	37.37	−4.82	37:30:37	−1.37
CEsr-2792000	0.82	40.01	1.90	38:28:30	−0.41
CEsr-2770000	0.82	41.50	5.70	36:59:50	−1.88
CEsr-2757000	0.82	42.16	7.37	39:04:10	0.19
CEsr-2856000	0.82	42.93	9.35	38:46:09	−0.11
CEsr-7096000	0.82	47.08	19.91	40:53:27	2.01
CEsr-2784000	0.82	39.64	0.97	35:34:49	−3.30
CEsr-5760000	0.82	41.76	6.36	36:49:44	−2.05
CEsr-2752000	0.82	45.04	14.71	41:16:10	2.39
C13 untreated mock-infected cells	0.64	128.15 ± 9.55	N.D	N.D	N.D
C13 untreated infected cells	0.64	36.71 ± 1.48	N.D	35:00:51	N.D
CEmr-2240100	0.64	46.00	25.32	39:20:56	2.54
CEhm-7100000	0.64	44.05	20.00	38:53:23	2.08
CEmr-1811300	0.64	42.12	14.73	38:28:19	1.66
CEsr-4872000	0.64	41.41	12.81	36:29:54	−0.31
CEsr-1968000	0.64	40.91	11.45	39:21:44	2.55
CEmr-1998800	0.64	52.00	41.65	43:09:47	6.35
CEsr-4202000	0.64	44.27	20.60	37:43:45	0.92
CEmr-3010400	0.64	41.70	13.60	37:42:13	0.89
CEsr-7564000	0.64	41.04	11.81	35:01:43	−1.78
CEmr-1942700	0.64	42.44	15.62	37:46:55	0.97
CEsr-1816000	0.64	44.70	21.77	38:44:38	1.93
CEsr-4886000	0.64	41.52	13.12	40:52:54	4.07
CEmr-2142200	0.64	36.05	−1.79	40:19:57	3.52
CEsr-6866000	0.64	37.10	1.07	35:13:06	−1.59
CEmr-3010600	0.64	39.23	6.87	36:22:34	−0.43
CEsr-2093000	0.64	42.73	16.40	37:10:53	0.37
CEmr-3046300	0.64	39.93	8.79	39:15:42	2.45
CEsr-7104000	0.64	16.97	−53.78	0:54:48	−35.90
CEsr-3285000	0.64	36.15	−1.53	34:27:54	−2.34
CEsr-3828000	0.64	36.18	−1.43	34:27:40	−2.35
CEsr-7094000	0.64	28.28	−22.97	34:30:11	−2.31
CEsr-4130000	0.64	30.88	−15.88	33:28:25	−3.34
CEsr-3837000	0.64	40.84	11.26	36:09:50	−0.65
CEsr-2829000	0.64	42.94	16.97	40:42:06	3.89
CEsr-3927000	0.64	43.72	19.11	38:07:59	1.32
CEsr-5740000	0.64	40.74	10.98	35:43:59	−1.08
CEmr-2621100	0.64	29.18	−20.50	31:04:20	−5.74
CEsr-4173000	0.64	41.45	12.91	36:02:00	−0.78
CEsr-4126000	0.64	43.07	17.33	34:30:28	−2.30
CEsr-3657000	0.64	36.40	−0.83	34:33:13	−2.26
CEmr-3188600	0.64	42.48	15.73	33:38:48	−3.16
CEsr-4129000	0.64	23.43	−36.17	30:58:19	−5.84
CEmr-2106500	0.64	38.85	5.84	39:14:52	2.44
CEmr-2988100	0.64	31.40	−14.45	33:23:33	−3.42
CEsr-4086000	0.64	42.46	15.67	34:38:18	−2.17
CEsr-1576000	0.64	39.84	8.53	34:56:00	−1.88
CEsr-4559000	0.64	36.86	0.42	34:10:55	−2.63
CEmr-1505700	0.64	39.29	7.04	35:57:38	−0.85
CEsr-4553000	0.64	38.24	4.18	33:54:23	−2.90
CEsr-4568000	0.64	36.85	0.39	36:35:09	−0.22
CEmr-2107400	0.64	41.53	13.15	35:46:15	−1.04
CEsr-4544000	0.64	43.94	19.71	37:44:58	0.94
CEmr-2663600	0.64	41.89	14.11	35:41:46	−1.11
CEsr-2569000	0.64	38.34	4.45	33:31:56	−3.28
CEmr-2286800	0.64	36.70	−0.02	33:05:32	−3.72
CEmr-2663400	0.64	36.69	−0.04	33:30:06	−3.31
CEmr-2638300	0.64	37.70	2.69	37:10:33	0.37
CEmr-1332700	0.64	36.29	−1.13	34:22:16	−2.44
CEsr-3466000	0.64	30.87	−15.91	32:49:35	−3.98
CEsr-5930000	0.64	36.76	0.14	35:09:34	−1.65
CEsr-6389000	0.64	37.79	2.94	37:22:46	0.57
CEsr-6157000	0.64	43.40	18.25	35:26:12	−1.37
CEsr-2612000	0.64	35.67	−2.82	33:37:48	−3.18
CEsr-5941000	0.64	43.26	17.86	34:25:59	−2.38
CEmr-3142000	0.64	35.26	−3.93	36:46:48	−0.03
CEsr-6811000	0.64	33.69	−8.21	28:29:39	−8.32
CEmr-2551900	0.64	42.30	15.25	36:37:25	−0.19
CEsr-7030000	0.64	36.67	−0.09	33:59:27	−2.82
CEsr-4917000	0.64	39.11	6.55	36:00:21	−0.80
CEsr-1692000	0.64	45.51	23.99	36:58:17	0.16
CEsr-5907000	0.64	39.07	6.44	34:19:43	−2.48
CEsr-1417000	0.64	36.29	−1.14	34:08:00	−2.68
CEsr-3557000	0.64	31.10	−15.27	33:35:47	−3.21
CEsr-3813000	0.64	39.71	8.17	39:06:01	2.29
CEsr-3855000	0.64	52.83	43.93	43:23:37	6.58
CEsr-1303000	0.64	40.43	10.14	36:01:55	−0.78
CEsr-4397000	0.64	36.58	−0.36	33:35:28	−3.22
CEsr-4966000	0.64	40.24	9.62	36:32:33	−0.27
CEsr-1435000	0.64	35.92	−2.14	35:21:53	−1.45
CEsr-2805000	0.64	47.79	30.20	37:08:17	0.33
CEsr-3922000	0.64	40.59	10.59	35:00:00	−1.81
CEsr-4151000	0.64	39.16	6.68	39:08:22	2.33
CEmr-3370500	0.64	49.82	35.73	39:38:11	2.83
CEmr-1870900	0.64	43.84	19.44	36:07:37	−0.68
CEsr-7265000	0.64	40.40	10.06	35:08:43	−1.66
CEmr-2570400	0.64	40.71	10.92	35:03:55	−1.74
CEsr-5730000	0.64	39.51	7.63	35:34:34	−1.23
CEsr-5642000	0.64	41.51	13.08	34:06:28	−2.70
CEmr-3189800	0.64	24.98	−31.94	17:58:35	−18.83
CEmr-2661100	0.64	49.63	35.22	42:52:05	6.06
C14 untreated mock-infected cells	0.64	138.85 ± 4.39	N.D	N.D	N.D
C14 untreated infected cells	0.64	42.15 ± 7.36	N.D	33:54:55	N.D
CEsr-4006000	0.64	36.51	−13.39	36:00:30	−0.38
CEmr-2583800	0.64	43.59	3.42	37:01:19	0.64
CEsr-3993000	0.64	37.95	−9.98	37:07:49	0.74
CEsr-4025000	0.64	38.13	−9.56	34:42:22	−1.68
CEmr-2038500	0.64	39.30	−6.76	37:07:33	0.74
CEmr-2325000	0.64	46.24	9.69	36:55:28	0.54
CEsr-4024000	0.64	37.98	−9.90	35:51:21	−0.53
CEsr-3519000	0.64	42.58	1.01	38:52:30	2.49
CEmr-2585600	0.64	46.70	10.79	40:52:16	4.48
CEsr-3956000	0.64	38.66	−8.28	35:15:11	−1.13
CEsr-5838000	0.64	45.60	8.17	36:53:09	0.50
CEmr-1544600	0.64	36.62	−13.12	34:47:58	−1.59
CEmr-3064600	0.64	42.54	0.93	35:19:22	−1.06
CEsr-1013000	0.64	39.71	−5.80	39:34:24	3.19
CEsr-3975000	0.64	45.17	7.16	38:55:49	2.54
CEmr-1685000	0.64	20.39	−51.62	34:42:23	−1.68
CEsr-6465000	0.64	38.82	−7.90	36:00:53	−0.37
CEmr-3393900	0.64	43.14	2.35	36:03:57	−0.32
CEmr-1846900	0.64	41.34	−1.93	36:20:25	−0.05
CEmr-3081900	0.64	43.35	2.83	38:07:04	1.73
CEsr-6222000	0.64	40.52	−3.88	35:17:09	−1.10
CEmr-2553900	0.64	38.63	−8.36	34:50:31	−1.54
CEmr-2507800	0.64	44.86	6.42	41:44:42	5.36
CEmr-2581900	0.64	46.93	11.34	40:02:36	3.66
CEmr-2571900	0.64	36.84	−12.61	33:46:53	−2.60
CEmr-2372800	0.64	41.07	−2.56	38:56:17	2.55
CEsr-2668000	0.64	40.20	−4.62	34:45:04	−1.64
CEsr-4953000	0.64	40.88	−3.02	35:44:33	−0.64
CEmr-2241000	0.64	44.28	5.05	42:10:02	5.78
CEsr-7232000	0.64	41.30	−2.02	34:15:24	−2.13
CEsr-5090000	0.64	36.18	−14.18	34:13:27	−2.16
CEsr-1485000	0.64	41.85	−0.71	39:02:39	2.66
CEsr-3006000	0.64	38.47	−8.74	36:27:09	0.07
CEsr-1537000	0.64	36.76	−12.79	33:42:46	−2.67
CEmr-8283000	0.64	46.63	10.62	34:43:17	−1.67
CEsr-5969000	0.64	36.06	−14.46	32:57:48	−3.42
CEsr-4485000	0.64	35.32	−16.21	32:34:13	−3.82
CEsr-1883000	0.64	40.32	−4.35	35:23:21	−1.00
CEap-2000000	0.64	36.74	−12.84	34:56:25	−1.45
CEmr-2631700	0.64	41.70	−1.09	38:53:15	2.50
CEmr-2506400	0.64	45.30	7.45	38:06:34	1.72
CEsr-2694000	0.64	39.35	−6.65	34:41:58	−1.69
CEsr-4526000	0.64	40.85	−3.08	36:19:28	−0.06
CEsr-3419000	0.64	34.38	−18.43	31:54:01	−4.49
CEmr-2147000	0.64	38.12	−9.56	34:27:52	−1.92
CEmr-2800500	0.64	43.19	2.47	34:15:39	−2.13
CEsr-2494000	0.64	31.02	−26.40	33:24:39	−2.98
CEsr-1693000	0.64	41.20	−2.26	36:36:50	0.23
CEmr-3228000	0.64	42.35	0.46	35:45:06	−0.63
CEsr-2611000	0.64	43.01	2.02	36:58:39	0.59
CEsr-2614000	0.64	37.92	−10.05	37:47:08	1.40
CEmr-2230000	0.64	38.69	−8.21	34:19:23	−2.06
CEsr-1778000	0.64	38.58	−8.47	34:22:54	−2.00
CEsr-1851000	0.64	38.97	−7.56	35:26:14	−0.95
CEsr-1822000	0.64	39.00	−7.48	36:49:10	0.43
CEsr-1752000	0.64	39.74	−5.73	38:23:49	2.01
CEmr-2708200	0.64	37.79	−10.36	36:48:10	0.42
CEsr-6311000	0.64	37.15	−11.86	34:46:16	−1.62
CEsr-6012000	0.64	33.47	−20.59	34:56:29	−1.45
CEsr-1829000	0.64	42.95	1.88	34:22:23	−2.01
CEmr-3223100	0.64	43.85	4.03	40:16:33	3.89
CEsr-3839000	0.64	40.67	−3.52	36:12:04	−0.19
CEsr-1837000	0.64	42.04	−0.26	36:23:34	0.01
CEmr-3222800	0.64	39.00	−7.49	38:16:42	1.89
CEmr-3463800	0.64	36.71	−12.92	37:36:32	1.22
CEmr-2613900	0.64	39.41	−6.51	37:10:27	0.79
CEmr-3464000	0.64	17.92	−57.49	20:26:01	−15.95
CEmr-3413400	0.64	39.20	−7.01	35:15:44	−1.12
CEsr-6888000	0.64	35.97	−14.68	33:25:35	−2.96
CEmr-2575300	0.64	38.54	−8.56	35:37:17	−0.77
CEmr-2614000	0.64	42.30	0.35	35:07:42	−1.26
CEmr-3274200	0.64	25.85	−38.68	33:28:27	−2.91
CEmr-3462400	0.64	38.55	−8.55	37:26:55	1.06
CEmr-3461000	0.64	39.36	−6.62	38:54:59	2.53
CEmr-3268100	0.64	32.58	−22.72	32:03:43	−4.32
CEmr-3337700	0.64	37.56	−10.90	36:18:30	−0.08
CEmr-2708400	0.64	35.28	−16.31	34:18:31	−2.08
CEmr-3271600	0.64	34.40	−18.40	34:40:38	−1.71
CEmr-3272000	0.64	29.68	−29.60	30:23:28	−6.00
CEmr-3463400	0.64	37.16	−11.85	34:44:20	−1.65
C15 untreated mock-infected cells	0.69	150.10 ± 8.93	N.D	N.D	N.D
C15 untreated infected cells	0.69	52.67 ± 1.16	N.D	41:12:09	N.D
CEmr-2301300	0.69	43.94	−16.58	38:12:40	−4.61
CEsr-7299000	0.69	49.50	−6.01	37:43:18	−5.10
CEmr-3053500	0.69	47.47	−9.88	39:18:17	−3.52
CEsr-5912000	0.69	47.44	−9.93	40:57:34	−1.87
CEmr-2707500	0.69	44.95	−14.65	41:21:42	−1.46
CEmr-2331800	0.69	43.48	−17.44	40:49:34	−2.00
CEsr-2518000	0.69	58.31	10.72	45:49:10	2.99
CEmr-1622200	0.69	46.77	−11.21	38:17:35	−4.53
CEsr-2646000	0.69	38.61	−26.69	38:03:05	−4.77
CEsr-2649000	0.69	54.90	4.24	43:18:35	0.48
CEsr-5516000	0.69	50.26	−4.58	41:03:18	−1.77
CEsr-1832000	0.69	47.40	−10.01	43:35:41	0.77
CEsr-7304000	0.69	45.33	−13.93	39:51:35	−2.97
CEmr-3052700	0.69	48.97	−7.03	40:55:23	−1.90
CEsr-7155000	0.69	48.66	−7.61	41:33:01	−1.28
CEsr-4927000	0.69	48.65	−7.63	39:37:54	−3.19
CEsr-3414000	0.69	47.39	−10.02	40:06:08	−2.72
CEsr-6870000	0.69	49.33	−6.34	41:51:13	−0.97
CEmr-2284300	0.69	49.43	−6.14	42:08:35	−0.68
CEsr-2640000	0.69	46.94	−10.88	41:28:11	−1.36
CEsr-1808000	0.69	48.74	−7.47	41:43:42	−1.10
CEsr-3405000	0.69	48.15	−8.58	39:47:06	−3.04
CEsr-2320000	0.69	43.89	−16.67	39:01:01	−3.81
CEsr-1517000	0.69	47.70	−9.44	40:51:37	−1.97
CEsr-3399000	0.69	51.76	−1.73	41:08:39	−1.68
CEsr-4438000	0.69	48.93	−7.10	39:34:41	−3.25
CEsr-4625000	0.69	46.78	−11.18	40:42:08	−2.12
CEsr-4422000	0.69	46.49	−11.74	39:34:36	−3.25
CEsr-4495000	0.69	51.93	−1.41	41:21:49	−1.46
CEsr-5809000	0.69	49.74	−5.56	40:38:30	−2.18
CEsr-1229000	0.69	49.54	−5.94	40:53:35	−1.93
CEsr-4364000	0.69	45.83	−12.98	38:50:51	−3.98
CEmr-1845600	0.69	43.30	−17.78	38:30:53	−4.31
CEsr-4639000	0.69	44.13	−16.21	40:28:55	−2.34
CEmr-2554300	0.69	48.03	−8.81	38:58:53	−3.84
CEsr-5747000	0.69	53.33	1.25	45:21:13	2.53
CEsr-4622000	0.69	47.58	−9.67	39:09:39	−3.67
CEsr-3634000	0.69	50.17	−4.75	42:48:52	−0.01
CEsr-2639000	0.69	46.88	−11.00	38:34:16	−4.25
CEsr-4195000	0.69	48.48	−7.96	39:11:03	−3.64
CEsr-4482000	0.69	47.79	−9.26	41:38:25	−1.19
CEsr-4175000	0.69	53.04	0.71	41:36:24	−1.22
CExr-hm18900	0.69	44.76	−15.01	39:31:16	−3.30
CEsr-3624000	0.69	47.11	−10.56	43:16:19	0.45
CEsr-4146000	0.69	50.11	−4.86	40:51:09	−1.97
CEsr-4193000	0.69	49.47	−6.08	38:54:35	−3.92
CEsr-4368000	0.69	39.14	−25.69	38:18:22	−4.52
CEsr-3838000	0.69	53.35	1.30	41:18:48	−1.51
CEsr-4149000	0.69	41.20	−21.78	37:48:38	−5.02
CEmr-1812500	0.69	55.48	5.34	46:11:20	3.36
CEmr-3184900	0.69	37.77	−28.28	40:30:14	−2.32
CEsr-4165000	0.69	52.24	−0.81	42:52:21	0.05
CEsr-3681000	0.69	51.15	−2.89	43:37:46	0.80
CEsr-7324000	0.69	33.57	−36.27	17:38:10	−25.19
CEsr-2004000	0.69	49.05	−6.87	40:11:48	−2.63
CEmr-1407201	0.69	48.76	−7.41	39:26:54	−3.38
CEsr-6085000	0.69	48.85	−7.25	40:36:52	−2.21
CEmr-1450000	0.69	41.35	−21.50	43:46:46	0.95
CEmr-2636700	0.69	44.47	−15.57	43:18:34	0.48
CEsr-618000	0.69	53.20	1.01	42:58:44	0.15
CEsr-5813000	0.69	52.87	0.37	43:33:16	0.73
CEsr-6239000	0.69	49.21	−6.57	40:52:28	−1.95
CEsr-5683000	0.69	51.29	−2.62	47:53:25	5.06
CEsr-1867000	0.69	52.67	0.00	38:17:44	−4.53
CEsr-2032000	0.69	52.01	−1.26	38:12:40	−4.61
CEmr-1607700	0.69	60.55	14.97	47:38:57	4.82
CEsr-5582000	0.69	50.81	−3.53	40:46:20	−2.05
CEmr-2703200	0.69	54.10	2.72	43:45:15	0.93
CEmr-2037700	0.69	53.09	0.80	42:25:36	−0.40
CEsr-5358000	0.69	37.70	−28.42	38:00:16	−4.82
CEsr-5682000	0.69	51.68	−1.88	40:34:39	−2.25
CEsr-1186000	0.69	52.99	0.60	40:56:52	−1.88
CEsr-4088000	0.69	48.14	−8.60	41:24:45	−1.41
CEmr-1214300	0.69	50.56	−4.01	40:57:23	−1.87
CEmr-2533200	0.69	53.03	0.68	43:06:13	0.28
CEmr-3220100	0.69	30.75	−41.62	5:28:30	−37.35
CEsr-2061000	0.69	52.38	−0.55	43:37:40	0.80
CEsr-1572000	0.69	54.38	3.25	43:51:26	1.03
CEsr-1337000	0.69	41.02	−22.12	37:23:07	−5.44
CEsr-6226000	0.69	51.42	−2.38	43:46:55	0.96
C16 untreated mock-infected cells	0.66	138.98 ± 6.86	N.D	N.D	N.D
C16 untreated infected cells	0.66	51.37 ± 3.05	N.D	40:03:01	N.D
CEmr-3200100	0.66	49.39	−3.86	42:22:03	−1.05
CEsr-2746000	0.66	56.36	9.72	41:08:17	−2.28
CEsr-4155000	0.66	45.81	−10.83	40:31:10	−2.90
CEmr-1943200	0.66	52.69	2.57	45:19:47	1.92
CEpa-46	0.66	45.05	−12.31	39:56:25	−3.47
CEsr-1927000	0.66	52.75	2.68	46:57:18	3.54
CEpa-65	0.66	50.00	−2.66	42:51:40	−0.55
CEsr-2801000	0.66	42.81	−16.67	42:40:35	−0.74
CEsr-5799000	0.66	47.83	−6.88	38:54:57	−4.50
CEmr-2023600	0.66	55.62	8.26	45:13:43	1.81
CEsr-7045000	0.66	53.42	3.99	44:06:30	0.69
CEsr-6310000	0.66	49.92	−2.83	43:08:22	−0.28
CEsr-7453000	0.66	46.43	−9.61	41:58:50	−1.43
CEsr-2153000	0.66	45.15	−12.11	42:51:17	−0.56
CEsr-3747000	0.66	52.94	3.06	41:09:32	−2.26
CEsr-2437000	0.66	51.94	1.10	38:37:10	−4.80
CEmr-1618400	0.66	44.64	−13.10	37:41:51	−5.72
CEsr-1291000	0.66	50.69	−1.32	42:22:17	−1.04
CEsr-6521000	0.66	46.79	−8.92	40:04:18	−3.34
CEsr-6283000	0.66	46.07	−10.32	41:09:09	−2.26
CEsr-7442000	0.66	46.12	−10.23	41:13:28	−2.19
CEsr-5723000	0.66	45.52	−11.39	41:10:17	−2.24
CEsr-1130000	0.66	42.95	−16.40	38:59:06	−4.43
CEsr-3100000	0.66	49.67	−3.31	43:34:04	0.15
CEsr-1107000	0.66	37.53	−26.94	41:54:24	−1.51
CEsr-1324000	0.66	51.29	−0.16	43:09:24	−0.26
CEmr-1634500	0.66	45.77	−10.90	40:13:28	−3.19
CEsr-1625000	0.66	50.69	−1.33	46:02:14	2.62
CEsr-4243000	0.66	54.36	5.82	42:19:09	−1.10
CEsr-5674000	0.66	45.45	−11.52	39:18:44	−4.10
CEsr-3337000	0.66	49.65	−3.35	39:25:13	−3.99
CEsr-3328000	0.66	44.34	−13.69	37:49:45	−5.59
CEsr-1156000	0.66	46.82	−8.86	38:21:37	−5.05
CEsr-3395000	0.66	46.04	−10.37	40:22:00	−3.05
CEmr-2094200	0.66	48.59	−5.41	40:40:42	−2.74
CEmr-hm68400	0.66	48.02	−6.51	42:07:42	−1.29
CEmr-1289300	0.66	58.96	14.77	39:55:49	−3.48
CEmr-2291300	0.66	44.55	−13.28	41:35:43	−1.82
CEsr-4078000	0.66	47.21	−8.10	40:46:48	−2.63
CEmr-1615300	0.66	53.39	3.93	42:14:00	−1.18
CEsr-3560000	0.66	54.34	5.78	44:13:07	0.80
CEsr-4135000	0.66	57.59	12.11	43:39:59	0.25
CEmr-2332900	0.66	54.80	6.68	43:40:55	0.27
CEmr-1613500	0.66	40.63	−20.92	41:22:10	−2.05
CEsr-3623000	0.66	39.38	−23.34	43:34:18	0.16
CEsr-4169000	0.66	31.88	−37.93	38:32:34	−4.87
CEsr-3324000	0.66	37.19	−27.61	37:04:12	−6.34
CEsr-1748000	0.66	50.12	−2.43	39:06:54	−4.30
CEsr-6578000	0.66	55.26	7.58	38:43:19	−4.69
CEmr-2607000	0.66	50.41	−1.86	39:11:26	−4.22
CEmr-2774900	0.66	48.54	−5.52	39:54:02	−3.51
CEmr-2776200	0.66	51.31	−0.11	41:24:08	−2.01
CEmr-2248200	0.66	47.14	−8.23	41:27:34	−1.96
CEmr-2466700	0.66	57.61	12.14	45:46:15	2.36
CEmr-2667700	0.66	46.92	−8.67	40:18:08	−3.11
CEmr-2024900	0.66	49.60	−3.45	40:06:18	−3.31
CEmr-2320500	0.66	47.03	−8.45	39:42:26	−3.71
CEmr-2025800	0.66	48.67	−5.26	42:04:38	−1.34
CEsr-6836000	0.66	42.17	−17.90	40:57:51	−2.45
CEmr-2603400	0.66	32.50	−36.74	38:23:40	−5.02
CEmr-2607100	0.66	44.62	−13.13	41:33:56	−1.85
CEsr-6617000	0.66	51.58	0.42	41:05:20	−2.33
CEmr-2090400	0.66	46.60	−9.29	40:00:03	−3.41
CEsr-2590000	0.66	51.31	−0.13	40:51:33	−2.56
CEmr-2180200	0.66	48.03	−6.51	39:58:55	−3.43
CEmr-2603300	0.66	47.11	−8.29	42:46:31	−0.64
CEsr-2575000	0.66	54.10	5.32	45:21:57	1.95
CEsr-2481000	0.66	53.80	4.73	43:08:02	−0.28
CEmr-2631300	0.66	48.42	−5.74	43:48:56	0.40
CEsr-6231000	0.66	52.63	2.46	41:09:50	−2.25
CEsr-7287000	0.66	49.62	−3.41	40:58:18	−2.44
CEmr-2281900	0.66	50.52	−1.66	43:19:08	−0.10
CEmr-2634200	0.66	54.07	5.26	40:21:17	−3.06
CEsr-5565000	0.66	44.65	−13.09	42:13:41	−1.19
CEsr-2441000	0.66	53.12	3.40	43:09:53	−0.25
CEmr-1275600	0.66	47.36	−7.81	42:07:55	−1.28
CEsr-6097000	0.66	49.25	−4.13	41:37:40	−1.79
CEsr-1311000	0.66	54.71	6.51	45:10:25	1.76
CEsr-5811000	0.66	54.99	7.05	43:05:33	−0.32
CEsr-5936000	0.66	47.81	−6.93	39:59:50	−3.42
C17 untreated mock-infected cells	0.74	135.02 ± 5.51	N.D	N.D	N.D
C17 untreated infected cells	0.74	46.33 ± 2.22	N.D	39:15:38	N.D
CEmr-3463500	0.74	44.11	−4.80	38:54:39	−2.49
CEmr-3413000	0.74	51.18	10.48	44:28:43	3.08
CEmr-2872400	0.74	46.37	0.08	42:59:45	1.60
CEmr-3463600	0.74	41.01	−11.47	45:19:08	3.92
CEsr-5733000	0.74	49.90	7.70	41:01:27	−0.37
CEmr-2708700	0.74	52.09	12.43	42:00:06	0.60
CEsr-6280000	0.74	55.14	19.02	41:27:39	0.06
CEmr-3461200	0.74	47.82	3.22	41:09:37	−0.24
CEmr-3462100	0.74	50.55	9.11	44:04:13	2.67
CEmr-3462900	0.74	49.61	7.09	44:56:39	3.55
CEmr-3463700	0.74	49.54	6.93	43:16:02	1.87
CEsr-6254000	0.74	48.74	5.21	42:09:47	0.76
CEsr-6939000	0.74	50.20	8.35	43:12:07	1.80
CEmr-2579100	0.74	44.29	−4.40	38:33:34	−2.84
CEmr-2572300	0.74	49.45	6.74	42:26:36	1.05
CEmr-2025601	0.74	45.99	−0.73	41:12:02	−0.20
CEmr-2961400	0.74	1.38	−97.02	0:59:43	−40.40
CEmr-2964100	0.74	56.09	21.07	52:02:44	10.65
CEmr-2742300	0.74	54.52	17.69	43:27:09	2.05
CEmr-2792300	0.74	53.77	16.06	42:49:53	1.43
CEmr-2391000	0.74	53.37	15.20	42:28:28	1.08
CEmr-2634600	0.74	51.00	10.09	42:07:28	0.73
CEmr-2968500	0.74	54.70	18.06	44:20:45	2.95
CEmr-2962200	0.74	48.85	5.44	48:06:52	6.72
CEmr-3074100	0.74	53.37	15.20	44:35:27	3.19
CEsr-2760000	0.74	55.41	19.61	42:10:15	0.77
CEsr-6434000	0.74	51.35	10.83	43:43:47	2.33
CEmr-2969900	0.74	50.40	8.78	41:13:23	−0.18
CEmr-3068100	0.74	65.55	41.50	50:31:06	9.12
CEmr-2690100	0.74	46.09	−0.51	41:46:48	0.38
CEsr-6470000	0.74	54.25	17.09	44:04:25	2.68
CEsr-6863000	0.74	56.28	21.47	44:55:09	3.52
CEmr-3101700	0.74	56.03	20.94	46:44:10	5.34
CEmr-2430500	0.74	52.03	12.31	42:22:20	0.97
CEsr-5312000	0.74	48.19	4.01	40:53:50	−0.50
CEsr-5412000	0.74	45.42	−1.95	37:00:50	−4.38
CEmr-1723400	0.74	56.62	22.22	44:15:55	2.87
CEmr-6678000	0.74	57.24	23.56	44:12:26	2.81
CEmr-2668700	0.74	47.54	2.62	40:42:08	−0.70
CEsr-3204000	0.74	42.54	−8.19	38:13:20	−3.18
CEsr-7463000	0.74	45.42	−1.97	39:03:31	−2.34
CEsr-6100000	0.74	27.21	−41.28	25:11:03	−16.21
CEmr-2886900	0.74	48.87	5.47	41:52:07	0.47
CEsr-6650000	0.74	42.86	−7.48	38:22:38	−3.02
CEmr-2810000	0.74	46.30	−0.06	41:01:11	−0.38
CEsr-3215000	0.74	53.04	14.48	43:00:54	1.62
CEmr-1671900	0.74	39.93	−13.80	38:58:05	−2.43
CEsr-5310000	0.74	48.29	4.23	41:23:41	0.00
CEmr-1215300	0.74	56.95	22.92	43:09:41	1.76
CEsr-4224000	0.74	51.86	11.94	42:32:05	1.14
CEsr-5610000	0.74	51.74	11.67	42:29:05	1.09
CEmr-2146600	0.74	65.36	41.08	52:18:01	10.90
CEsr-2970000	0.74	55.60	20.02	44:54:55	3.52
CEmr-3053400	0.74	52.13	12.52	44:00:48	2.62
CEsr-1199000	0.74	50.32	8.61	45:23:38	4.00
CEmr-3017000	0.74	52.25	12.79	45:05:40	3.70
CEsr-6980000	0.74	48.84	5.42	43:40:13	2.27
CEmr-3227900	0.74	46.21	−0.26	41:27:23	0.06
CEsr-7047000	0.74	42.48	−8.30	39:40:51	−1.72
CEsr-7076000	0.74	53.05	14.51	40:29:04	−0.91
CEmr-3098100	0.74	45.07	−2.71	42:02:31	0.64
CEmr-3015800	0.74	52.11	12.48	43:08:32	1.74
CEmr-3014800	0.74	50.40	8.78	43:24:33	2.01
CEmr-1989100	0.74	50.59	9.20	40:46:06	−0.63
CEmr-2361000	0.74	57.44	23.98	44:11:00	2.79
CEsr-6968000	0.74	49.75	7.39	41:00:03	−0.40
CEsr-5823000	0.74	57.02	23.07	43:28:23	2.07
CEmr-8242000	0.74	50.90	9.86	40:39:53	−0.73
CEmr-2022800	0.74	44.11	−4.78	39:52:34	−1.52
CEmr-2503900	0.74	48.20	4.04	40:30:49	−0.88
CEsr-2166000	0.74	46.74	0.89	42:57:21	1.56
CEsr5868000	0.74	49.32	6.45	41:27:13	0.06
CEmr-2362900	0.74	49.50	6.83	40:13:27	−1.17
CEsr-2471000	0.74	49.47	6.78	39:50:05	−1.56
CEsr-5810000	0.74	46.48	0.32	41:35:02	0.19
CEsr-4681000	0.74	51.46	11.07	42:41:04	1.29
CEsr-5159000	0.74	50.81	9.66	41:35:32	0.19
CEmr-2500900	0.74	43.14	−6.88	40:31:31	−0.87
CEsr-2986000	0.74	45.13	−2.59	41:15:34	−0.14
CEmr-2792600	0.74	48.88	5.50	40:48:22	−0.59
C18 untreated mock-infected cells	0.52	144.70 ± 11.46	N.D	N.D	N.D
C18 untreated infected cells	0.52	54.02 ± 2.92	N.D	43:17:18	N.D
CEmr-2390700	0.52	51.60	−4.48	44:57:54	−0.73
CEsr-7201000	0.52	54.09	0.13	43:33:54	−2.13
CEmr-2699500	0.52	50.89	−5.78	50:09:04	4.45
CEsr-4757000	0.52	54.06	0.08	45:51:13	0.16
CEsr-7202000	0.52	54.11	0.17	46:22:47	0.68
CEmr-3000200	0.52	51.05	−5.50	45:05:42	−0.60
CEmr-3053000	0.52	49.56	−8.25	43:04:57	−2.62
CEmr-2435800	0.52	59.14	9.48	52:35:09	6.89
CEmr-1561000	0.52	55.42	2.60	45:15:22	−0.44
CEsr-7294000	0.52	61.06	13.04	48:50:21	3.14
CEmr-2962500	0.52	44.72	−17.22	46:31:58	0.83
CEsr-7235000	0.52	51.42	−4.81	44:44:32	−0.96
CEmr-3053700	0.52	53.18	−1.56	46:10:32	0.48
CEsr-7228000	0.52	29.52	−45.36	22:31:44	−23.17
CEsr-7223000	0.52	26.26	−51.39	21:46:11	−23.93
CEsr-7289000	0.52	50.90	−5.78	45:24:24	−0.29
CEmr-2980700	0.52	54.43	0.77	43:53:04	−1.81
CEsr-7238000	0.52	57.08	5.67	43:06:02	−2.60
CEsr-7214000	0.52	37.74	−30.13	50:33:16	4.86
CEsr-2015000	0.52	52.99	−1.91	42:44:42	−2.95
CEmr-2980100	0.52	51.84	−4.04	45:20:56	−0.35
CEmr-2533600	0.52	53.76	−0.48	45:41:06	−0.01
CEsr-4739000	0.52	58.78	8.82	46:08:40	0.45
CEsr-4770000	0.52	51.08	−5.44	43:27:41	−2.24
CEsr-4733000	0.52	55.97	3.61	44:04:33	−1.62
CEsr-4654000	0.52	52.19	−3.39	42:51:32	−2.84
CEmr-1997500	0.52	52.76	−2.34	43:09:57	−2.53
CEsr-4769000	0.52	54.98	1.78	44:22:38	−1.32
CEmr-2340700	0.52	49.15	−9.01	44:42:24	−0.99
CEsr-4628000	0.52	56.35	4.32	46:19:58	0.63
CEsr-1155000	0.52	49.06	−9.18	42:50:52	−2.85
CEmr-2105500	0.52	61.71	14.23	50:12:36	4.51
CEmr-2870900	0.52	54.00	−0.03	55:44:04	10.04
CEsr-1295000	0.52	55.39	2.54	45:19:14	−0.38
CEmr-1545600	0.52	54.91	1.65	46:44:04	1.04
CEsr-1061000	0.52	58.46	8.22	46:36:18	0.91
CEmr-2697500	0.52	49.93	−7.57	42:36:27	−3.09
CEsr-6040000	0.52	59.81	10.72	49:01:05	3.32
CEsr-1171000	0.52	55.40	2.56	42:59:20	−2.71
CEsr-7549000	0.52	50.05	−7.34	44:22:49	−1.32
CEsr-1031000	0.52	54.79	1.43	42:59:57	−2.70
CEsr-1145000	0.52	56.60	4.77	46:49:06	1.12
CEmr-1621300	0.52	55.99	3.65	46:40:36	0.98
CEsr-7066000	0.52	57.10	5.71	47:16:45	1.58
CEsr-7152000	0.52	54.72	1.30	44:03:51	−1.63
CEsr-7127000	0.52	56.95	5.43	43:30:22	−2.19
CEsr-6317000	0.52	46.39	−14.12	44:13:45	−1.47
CEsr-2581000	0.52	50.48	−6.55	45:53:13	0.19
CEsr-2586000	0.52	51.63	−4.42	43:55:43	−1.77
CEsr-6390000	0.52	55.96	3.59	45:38:59	−0.05
CEsr-6969000	0.52	56.66	4.88	45:35:13	−0.11
CEmr-2733800	0.52	61.10	13.11	47:56:02	2.24
CEsr-2527000	0.52	57.04	5.60	46:00:56	0.32
CEsr-6320000	0.52	56.51	4.62	46:55:44	1.23
CEsr-7128000	0.52	42.14	−21.99	54:45:12	9.06
CEsr-3746000	0.52	54.00	−0.04	46:04:55	0.38
CEsr-2568000	0.52	1.85	25.00	−1.70368691	−1.31
CEsr-5085000	0.52	44.37	−17.86	44:08:10	−1.56
CEsr-6387000	0.52	48.02	−11.10	42:18:53	−3.38
CEmr-2331600	0.52	54.74	1.33	45:00:51	−0.68
CEmr-1945600	0.52	54.97	1.77	44:36:40	−1.09
CEmr-2293500	0.52	51.69	−4.31	42:48:07	−2.90
CEsr-6693000	0.52	59.83	10.75	48:38:30	2.94
CEmr-1675800	0.52	56.81	5.18	46:29:20	0.79
CEmr-2331700	0.52	58.64	8.56	44:56:00	−0.76
CEmr-1208500	0.52	56.80	5.14	44:57:51	−0.73
CEsr-2226000	0.52	55.39	2.54	44:26:46	−1.25
CEmr-1329200	0.52	59.44	10.05	45:31:05	−0.18
CEsr-6686000	0.52	57.56	6.56	43:03:08	−2.65
CEmr-2105300	0.52	56.66	4.89	48:09:11	2.45
CEsr-4218000	0.52	54.56	1.00	47:39:45	1.96
CEsr-3787000	0.52	54.21	0.35	42:31:57	−3.17
CEsr-6688000	0.52	60.95	12.83	46:07:03	0.42
CEmr-2103500	0.52	46.82	−13.33	42:08:21	−3.56
CEmr-6668000	0.52	52.36	−3.07	43:30:09	−2.20
CEmr-2330600	0.52	53.51	−0.93	43:00:01	−2.70
CEmr-1546000	0.52	49.77	−7.87	42:28:02	−3.23
CEsr-5018000	0.52	52.39	−3.01	46:13:51	0.53
CEmr-1949600	0.52	51.49	−4.68	44:51:11	−0.85
CEsr-2601000	0.52	57.87	7.12	45:12:59	−0.48
C19 untreated mock-infected cells	0.67	127.33 ± 8.37	N.D	N.D	N.D
C19 untreated infected cells	0.67	41.72 ± 0.95	N.D	33:53:05	N.D
CEsr-3029000	0.67	37.32	−10.54	37:51:19	1.35
CEsr-3050000	0.67	42.50	1.86	37:08:27	0.63
CEsr-5040000	0.67	37.46	−10.22	38:46:11	2.26
CEsr-2291000	0.67	38.71	−7.22	33:58:54	−2.53
CEsr-1599000	0.67	44.51	6.69	38:11:41	1.69
CEsr-3093000	0.67	42.16	1.05	37:10:38	0.67
CEsr-5111000	0.67	41.54	−0.43	36:31:18	0.01
CEsr-1583000	0.67	41.11	−1.47	36:31:00	0.01
CEsr-3070000	0.67	40.40	−3.17	37:10:26	0.67
CEmr-3023600	0.67	40.27	−3.47	36:28:16	−0.04
CEsr-5112000	0.67	40.71	−2.43	37:46:13	1.26
CEsr-5845000	0.67	46.93	12.48	42:25:46	5.92
CEsr-5619000	0.67	40.78	−2.26	36:05:21	−0.42
CEpa-18	0.67	40.61	−2.67	36:22:06	−0.14
CEsr-1073000	0.67	38.15	−8.56	33:53:01	−2.62
CEsr-5171000	0.67	34.39	−17.57	34:29:21	−2.02
CEmr-2532200	0.67	46.39	11.18	39:35:59	3.09
CEsr-1586000	0.67	42.25	1.26	36:46:43	0.27
CEsr-1024000	0.67	44.16	5.83	39:05:38	2.59
CEsr-1240000	0.67	45.42	8.86	38:26:59	1.94
CEmr-2506700	0.67	41.34	−0.92	42:09:41	5.65
CEsr-1074000	0.67	44.16	5.84	37:14:24	0.73
CEsr-7126000	0.67	39.11	−6.26	33:53:49	−2.61
CEmr-2505900	0.67	40.86	−2.06	36:00:37	−0.50
CEsr-1250000	0.67	42.63	2.18	37:05:17	0.58
CEsr-4979000	0.67	40.50	−2.92	35:26:57	−1.06
CEmr-2434900	0.67	46.21	10.74	40:51:54	4.36
CEmr-2871700	0.67	33.15	−20.55	19:30:47	−16.99
CEsr-7199000	0.67	35.55	−14.79	38:32:28	2.03
CEsr-6579000	0.67	45.93	10.08	38:35:41	2.09
CEsr-2991000	0.67	40.18	−3.69	36:10:59	−0.32
CEsr-2348000	0.67	37.09	−11.09	32:30:34	−4.00
CEsr-3041000	0.67	44.47	6.59	37:23:17	0.88
CEsr-3069000	0.67	41.71	−0.03	35:28:10	−1.04
CEsr-4983000	0.67	38.52	−7.69	35:49:09	−0.69
CEsr-5110000	0.67	39.64	−5.00	34:22:30	−2.13
CEsr-3083000	0.67	30.59	−26.68	35:50:41	−0.66
CEmr-2364300	0.67	39.77	−4.69	35:17:23	−1.22
CEsr-1225000	0.67	43.28	3.72	37:25:59	0.93
CEsr-4909000	0.67	34.89	−16.38	36:26:55	−0.06
CEsr-6505000	0.67	43.46	4.17	39:25:17	2.91
CEsr-3705000	0.67	35.26	−15.50	40:04:12	3.56
CEsr-3741000	0.67	42.73	2.40	36:59:50	0.49
CEmr-2577800	0.67	42.39	1.61	40:18:49	3.81
CEmr-2491200	0.67	35.22	−15.60	35:48:44	−0.69
CEsr-3717000	0.67	42.52	1.90	35:31:56	−0.97
CEsr-6249000	0.67	40.53	−2.86	35:50:39	−0.66
CEsr-3751000	0.67	42.15	1.02	36:05:23	−0.42
CEmr-2578900	0.67	40.05	−4.00	37:25:39	0.92
CEsr-3443000	0.67	42.15	1.02	35:51:22	−0.65
CEmr-2578100	0.67	37.63	−9.81	36:50:03	0.33
CEsr-3721000	0.67	42.65	2.23	38:44:57	2.24
CEsr-3446000	0.67	43.51	4.28	36:04:30	−0.43
CEmr-1622900	0.67	39.31	−5.80	34:38:16	−1.87
CEsr-3494000	0.67	40.59	−2.70	36:38:33	0.14
CEsr-3762000	0.67	42.35	1.51	36:29:50	−0.01
CEsr-1611000	0.67	39.87	−4.44	38:38:27	2.13
CEsr-3461000	0.67	41.37	−0.85	35:41:45	−0.81
CEsr-5122000	0.67	40.21	−3.62	39:06:12	2.60
CEsr-6276000	0.67	40.76	−2.30	36:28:46	−0.03
CEsr-3493000	0.67	40.95	−1.84	35:47:28	−0.72
CEsr-6443000	0.67	30.13	−27.78	36:18:30	−0.20
CEsr-5652000	0.67	42.03	0.73	37:04:51	0.57
CEsr-6126000	0.67	38.11	−8.66	33:54:32	−2.60
CEmr-2557200	0.67	40.76	−2.31	38:11:17	1.68
CEsr-6445000	0.67	39.47	−5.41	36:06:36	−0.40
CEsr-6148000	0.67	40.08	−3.94	35:58:33	−0.53
CEmr-2632800	0.67	37.18	−10.90	35:59:41	−0.51
CEmr-2288100	0.67	44.51	6.69	36:46:21	0.27
CEmr-2740700	0.67	40.35	−3.28	34:27:46	−2.04
CEsr-5673000	0.67	37.85	−9.28	36:59:08	0.48
CEmr-3250900	0.67	43.59	4.47	38:38:45	2.14
CEmr-2741600	0.67	41.44	−0.67	38:22:36	1.87
CEmr-2584500	0.67	42.25	1.27	36:33:28	0.05
CEsr-618400	0.67	41.76	0.09	43:50:01	7.33
CEmr-2742200	0.67	38.09	−8.72	36:48:00	0.29
CEsr-1622000	0.67	39.82	−4.55	38:21:15	1.85
CEsr-4658000	0.67	40.29	−3.43	39:02:22	2.53
CEmr-1401400	0.67	40.97	−1.80	36:30:04	−0.01
CEmr-2435300	0.67	40.56	−2.79	38:47:23	2.28
C20 untreated mock-infected cells	0.72	136.01 ± 7.86	N.D	N.D	N.D
C20 untreated infected cells	0.72	41.83 ± 0.79	N.D	31:51:58	N.D
CEmr-2691200	0.72	37.52	−10.31	36:20:43	−0.20
CEmr-1727100	0.72	41.62	−0.52	36:44:40	0.20
CEmr-1845800	0.72	40.41	−3.39	36:42:19	0.16
CEsr-2264000	0.72	41.97	0.32	36:16:57	−0.26
CEmr-2635500	0.72	37.77	−9.72	34:49:42	−1.72
CEsr-3617000	0.72	42.33	1.19	37:09:22	0.61
CEmr-1847600	0.72	42.10	0.64	36:32:50	0.00
CEsr-3674000	0.72	34.32	−17.97	41:10:16	4.62
CEsr-1947000	0.72	38.37	−8.29	37:38:37	1.10
CEsr-3653000	0.72	37.96	−9.26	35:58:46	−0.57
CEsr-5736000	0.72	45.13	7.87	39:12:03	2.65
CEsr-3677000	0.72	42.43	1.42	37:13:04	0.67
CEsr-3650000	0.72	36.90	−11.79	35:08:27	−1.41
CEmr-2987200	0.72	37.83	−9.57	36:31:32	−0.02
CEmr-2401400	0.72	39.80	−4.87	35:42:09	−0.84
CEsr-3693000	0.72	41.20	−1.52	37:08:34	0.60
CEmr-3040700	0.72	38.52	−7.92	36:24:26	−0.14
CEsr-7418000	0.72	32.99	−21.14	40:10:59	3.64
CEmr-3392800	0.72	40.98	−2.04	36:19:48	−0.22
CEmr-1479000	0.72	40.03	−4.30	35:42:13	−0.84
CEmr-2504900	0.72	42.31	1.15	37:17:48	0.75
CEmr-3395500	0.72	36.36	−13.09	34:12:50	−2.33
CEmr-1440900	0.72	37.72	−9.84	34:10:05	−2.38
CEsr-7023000	0.72	37.59	−10.13	33:38:15	−2.91
CEsr-6449000	0.72	42.24	0.97	37:57:48	1.42
CEsr-5118000	0.72	40.54	−3.10	36:24:59	−0.13
CEmr-2285600	0.72	42.24	0.98	37:09:11	0.61
CEsr-6751000	0.72	41.68	−0.36	35:38:27	−0.91
CEmr-3097600	0.72	38.86	−7.11	36:12:54	−0.33
CEsr-6946000	0.72	36.72	−12.22	32:10:56	−4.36
CEsr-6596000	0.72	36.59	−12.53	33:06:01	−3.45
CEmr-2328600	0.72	48.31	15.48	38:16:13	1.72
CEmr-1634400	0.72	38.17	−8.77	35:00:45	−1.53
CEsr-6096000	0.72	42.05	0.53	35:58:15	−0.58
CEmr-1844400	0.72	40.23	−3.83	37:29:24	0.94
CEsr-3579000	0.72	39.50	−5.58	37:12:26	0.66
CEmr-2431900	0.72	45.38	8.48	39:50:28	3.29
CEmr-2433900	0.72	42.23	0.94	37:30:04	0.95
CEmr-2430600	0.72	42.58	1.78	37:00:34	0.46
CEmr-2435000	0.72	46.81	11.89	36:47:06	0.24
CEsr-6165000	0.72	37.61	−10.09	34:24:59	−2.13
CEmr-1762300	0.72	38.28	−8.50	36:34:59	0.04
CEmr-2435600	0.72	47.40	13.31	44:35:24	8.04
CEsr-7016000	0.72	37.09	−11.35	36:44:53	0.20
CEmr-3203200	0.72	38.01	−9.13	34:18:05	−2.25
CEsr-1787000	0.72	39.79	−4.87	35:42:02	−0.85
CEsr-4751000	0.72	38.23	−8.62	37:40:23	1.13
CEsr-4752000	0.72	39.55	−5.46	34:36:57	−1.93
CEsr-2877000	0.72	37.47	−10.43	35:16:48	−1.27
CEsr-2128000	0.72	43.38	3.69	35:35:47	−0.95
CEsr-4761000	0.72	43.06	2.94	35:49:35	−0.72
CEsr-1946000	0.72	45.27	8.23	36:38:32	0.10
CEmr-1384800	0.72	40.05	−4.27	35:54:04	−0.65
CEsr-3427000	0.72	43.81	4.72	36:24:31	−0.14
CEsr-1498000	0.72	37.58	−10.16	33:22:04	−3.18
CEmr-1334000	0.72	44.83	7.17	38:14:04	1.69
CEsr-2276000	0.72	41.54	−0.71	38:18:40	1.76
CEsr-1515000	0.72	41.87	0.09	37:01:08	0.47
CEsr-5948000	0.72	34.79	−16.83	37:48:19	1.26
CEsr-1527000	0.72	41.90	0.17	35:30:17	−1.04
CEsr-5972000	0.72	38.95	−6.88	35:04:16	−1.48
CEsr-1547000	0.72	41.89	0.14	35:45:19	−0.79
CEsr-5974000	0.72	41.66	−0.41	36:13:43	−0.32
CEmr-1507400	0.72	40.23	−3.82	34:38:20	−1.91
CEmr-1511500	0.72	37.20	−11.08	34:54:40	−1.64
CEmr-2100100	0.72	37.21	−11.05	35:25:01	−1.13
CEmr-3263100	0.72	39.93	−4.54	36:19:32	−0.22
CEmr-1618000	0.72	32.62	−22.02	35:41:52	−0.85
CEsr-7428000	0.72	39.37	−5.89	35:29:14	−1.06
CEsr-6457000	0.72	44.30	5.90	36:44:32	0.20
CEmr-1506900	0.72	40.27	−3.75	36:08:35	−0.40
CEsr-4661000	0.72	37.63	−10.06	33:08:45	−3.40
CEmr-2243900	0.72	36.52	−12.69	35:32:01	−1.01
CEmr-2965500	0.72	39.27	−6.12	35:52:26	−0.67
CEmr-1500800	0.72	39.20	−6.30	39:26:46	2.90
CEmr-2968600	0.72	37.32	−10.78	34:55:20	−1.62
CEmr-2388200	0.72	36.91	−11.76	35:24:23	−1.14
CEmr-3264000	0.72	38.44	−8.11	36:17:24	−0.26
CEsr-6750000	0.72	40.03	−4.31	35:53:48	−0.65
CEmr-2031800	0.72	41.31	−1.26	36:07:38	−0.42
C21 untreated mock-infected cells	0.68	121.79 ± 4.41	N.D	N.D	N.D
C21 untreated infected cells	0.68	40.10 ± 1.60	N.D	37:02:50	N.D
CEmr-1870900	0.68	39.93	−4.54	36:36:04	−1.56
CEsr-6210000	0.68	42.19	0.86	37:50:29	−0.32
CEsr-6217000	0.68	43.69	4.43	36:59:24	−1.17
CEsr-6237000	0.68	38.57	−7.79	35:08:36	−3.02
CEsr-6238000	0.68	41.02	−1.95	36:35:35	−1.57
CEsr-6265000	0.68	42.22	0.92	37:29:20	−0.67
CEsr-6990000	0.68	37.32	−10.80	37:39:04	−0.51
C22 untreated mock-infected cells	0.95	87.80 ± 0.86	N.D	N.D	N.D
C22 untreated infected cells	0.95	37.36 ± 0.39	N.D	35:11:02	N.D
CEmr-3267300	0.95	33.80	−9.53	36:19:49	1.15
CEmr-3123500	0.95	39.85	6.66	38:08:15	2.95
CEmr-3484600	0.95	46.38	24.12	43:02:01	7.85
CEmr-3540300	0.95	36.45	−2.44	36:46:18	1.59
CEmr-3540900	0.95	47.23	26.41	42:51:09	7.67
CEmr-3541100	0.95	36.38	−2.64	38:02:07	2.85
C23 untreated mock-infected cells	0.81	92.16 ± 5.50	N.D	N.D	N.D
C23 untreated infected cells	0.81	41.58 ± 0.30	N.D	37:48:19	N.D
CEmr-3266100	0.81	16.52	−60.27	33:49:27	−3.98
CEmr-3411000	0.81	31.01	−25.41	0:27:01	−37.35
CEmr-3410400	0.81	39.05	−6.07	46:16:06	3.00
CEmr-3410900	0.81	43.20	3.90	40:05:19	2.28
CEmr-3411100	0.81	39.58	−4.79	38:51:00	1.04
CEsr-1088000	0.81	33.97	−18.29	37:17:11	−0.52
C24 untreated mock-infected cells	0.94	111.24 ± 13.06	N.D	N.D	N.D
C24 untreated infected cells	0.94	38.27 ± 1.68	N.D	39:38:12	N.D
CEmr-1982700	0.94	38.68	1.06	39:58:22	0.34
CEmr-3222500	0.94	35.28	−7.81	35:31:27	−4.11
CEmr-3261900	0.94	40.63	6.15	42:50:00	3.20
CEmr-3262100	0.94	46.53	21.58	45:07:04	5.48
CEmr-3262300	0.94	43.05	12.47	41:12:22	1.57
CEmr-3266400	0.94	40.79	6.56	42:50:56	3.21
C25 untreated mock-infected cells	0.86	110.62 ± 2.41	N.D	N.D	N.D
C25 untreated infected cells	0.86	36.83 ± 6.33	N.D	39:22:44	N.D
CEmr-3266500	0.86	40.71	10.52	43:33:36	3.66
CEmr-3266700	0.86	39.42	7.01	43:58:16	4.08
CEmr-3266800	0.86	45.81	24.36	43:18:45	3.42
CEmr-3267000	0.86	41.12	11.63	44:01:42	4.13
CEmr-3267200	0.86	40.48	9.90	40:21:36	0.46
CEmr-3267600	0.86	41.18	11.79	46:43:26	6.83
C26 untreated mock-infected cells	0.86	101.52 ± 2.66	N.D	N.D	N.D
C26 untreated infected cells	0.86	32.59 ± 3.77	N.D	38:16:03	N.D
CEmr-3267700	0.86	40.97	25.73	45:23:46	4.47
CEmr-3267800	0.86	39.55	21.38	43:16:26	−15.94
CEmr-3267900	0.86	53.04	62.78	6:24:17	10.62
CEmr-3268000	0.86	21.56	−33.83	18:19:35	−19.94
CEmr-3269600	0.86	36.81	12.95	38:57:13	0.69
CEmr-3269800	0.86	42.93	31.75	43:15:14	4.99
C27 untreated mock-infected cells	0.87	111.24 ± 13.73	N.D	N.D	N.D
C27 untreated infected cells	0.87	44.12 ± 0.07	N.D	40:59:13	N.D
CEsr-233100	0.87	47.76	8.25	40:17:57	−0.69
CEsr-1824000	0.87	35.98	−18.44	37:38:31	−3.34
CEsr-1077000	0.87	40.70	−7.74	44:27:13	3.47
CEsr--1075000	0.87	45.12	2.27	42:52:12	1.88
CEmr-3411200	0.87	39.45	−10.59	43:59:57	3.01
CEmr-3269900	0.87	38.50	−12.74	39:17:16	−1.70
C28 untreated mock-infected cells	0.73	92.91 ± 6.21	N.D	N.D	N.D
C28 untreated infected cells	0.73	37.86 ± 0.36	N.D	38:52:58	N.D
CEsr-1057000	0.73	37.93	0.15	37:51:08	−1.03
CEmr-3269700	0.73	23.47	−32.63	17:24:46	−21.47
CEmr-3123300	0.73	29.65	−18.62	33:05:10	−5.80
C29 untreated mock-infected cells	0.98	94.21 ± 8.18	N.D	N.D	N.D
C29 untreated infected cells	0.98	41.33 ± 4.01	N.D	45:42:21	N.D
CEmr-3222400	0.98	23.47	−40.48	29:49:30	−19.43
CEmr-3261500	0.98	29.65	−26.48	45:36:20	−3.65
C30 untreated mock-infected cells	0.57	106.17 ± 6.57	N.D	N.D	N.D
C30 untreated infected cells	0.57	41.18 ± 6.69	N.D	51:23:32	N.D
CEmr-3261600	0.57	45.80	10.46	44:16:01	−7.13
CEmr-3269400	0.57	48.90	17.48	53:03:57	1.67
CEmr-3410500	0.57	51.22	22.75	51:22:46	−0.01

**Table 3:** Screening of 41 compounds (in-house antiviral library) selected for their effects against different human viruses at 4 concentrations (0.4, 2, 10 and 50 µM). For each compound, the following values are provided: the area under normalised Cell Index curve (AUC_n_), the %AUC_n_ compared to AUC_n_ of untreated infected cells, the time required for the cell index to decrease by 50 % after virus infection (CIT_50_) and the ΔCIT_50_ value obtained by comparing results for treated *vs* untreated, EHV-1-infected control wells. The Z’-factor was used to assess the quality of the screening in 96-well format. For untreated mock-infected and untreated infected cells, the mean ± SD of AUC_n_ is reported. *N.D., not determinable.*

Compound name	Supplier	Z'-factor	AUC_n_				%AUC_n_				CIT_50_				ΔCIT_50_			
			50 µM	10 µM	2 µM	0.4 µM	50 µM	10 µM	2 µM	0.4 µM	50 µM	10 µM	2 µM	0.4 µM	50 µM	10 µM	2 µM	0.4 µM
A1 untreated mock-infected cells		0.74	156.79 ± 9.26				N.D.				N.D.				N.D.			
A1 untreated infected cells		0.74	57.08 ± 2.59				N.D.				31:52:27				N.D.			
Mercaptopurine	Target Mol	0.74	41.98	49.59	52.52	50.67	−39.72	−19.72	−12.00	−16.87	30:04:57	32:51:02	33:41:02	32:24:07	−1.76	1.01	1.84	0.56
Thioguanine	Target Mol	0.74	45.22	51.94	51.43	51.39	−31.22	−13.54	−14.86	−14.98	28:14:30	32:05:21	31:41:42	30:27:49	−3.60	0.24	−0.15	−1.38
Valganciclovir	Target Mol	0.74	177.47	131.87	84.84	53.52	316.71	196.74	73.03	−9.38	147:44:31	28:32:34	47:53:27	33:25:08	115.90	−3.30	16.05	1.57
Valaciclovir	Target Mol	0.74	70.89	48.32	45.80	51.60	36.33	−23.04	−29.68	−14.44	41:13:30	28:47:46	26:26:57	32:02:04	9.38	−3.05	−5.40	0.19
Penciclovir	Target Mol	0.74	63.88	49.84	49.04	48.81	17.89	−19.06	−21.16	−21.75	40:47:08	30:46:02	32:17:14	29:24:29	8.94	−1.08	0.44	−2.44
Famciclovir	Target Mol	0.74	46.39	48.66	47.51	49.39	−28.13	−22.16	−25.17	−20.23	29:21:08	30:34:39	29:56:22	31:01:37	−2.49	−1.27	−1.91	−0.82
Maribavir	Target Mol	0.74	51.30	49.71	51.46	53.00	−15.22	−19.40	−14.79	−10.74	37:21:43	32:49:24	32:38:31	32:55:22	5.52	0.98	0.80	1.08
Didanosine	Target Mol	0.74	54.52	49.67	60.00	49.97	−6.74	−19.52	7.68	−18.72	29:37:46	30:05:36	34:56:53	33:17:07	−2.22	−1.75	3.10	1.44
Stavudine	Target Mol	0.74	50.28	49.44	52.49	53.54	−17.89	−20.11	−12.08	−9.33	31:04:33	29:27:03	31:30:50	32:47:16	−0.77	−2.39	−0.33	0.94
Lamivudine	Target Mol	0.74	53.84	52.80	51.53	50.36	−8.52	−11.26	−14.60	−17.67	32:23:37	33:09:48	31:39:41	32:07:56	0.55	1.32	−0.18	0.29
Abacavir	Target Mol	0.74	50.57	53.67	52.64	48.42	−17.14	−8.99	−11.68	−22.80	26:56:17	32:21:46	32:27:26	29:59:41	−4.91	0.52	0.61	−1.85
Adefovir dipivoxil	Target Mol	0.74	49.71	88.67	119.24	123.95	−19.41	83.10	163.52	175.92	24:16:02	147:44:31	147:44:31	123:32:51	−7.58	115.90	115.90	91.70
Tenofovir disoproxil	Target Mol	0.74	77.88	62.32	52.05	52.19	54.70	13.76	−13.25	−12.87	52:52:57	35:46:29	30:56:57	33:05:05	21.04	3.93	−0.90	1.24
Emtricitabine	Target Mol	0.74	48.89	53.46	54.12	49.47	−21.56	−9.54	−7.78	−20.04	27:48:05	35:02:51	32:51:10	32:05:11	−4.04	3.20	1.01	0.24
Capecitabine	Target Mol	0.74	47.51	50.66	50.25	46.70	−25.17	−16.89	−17.99	−27.30	31:08:32	30:10:49	32:57:59	28:44:35	−0.70	−1.66	1.12	−3.10
Cytarabine	Target Mol	0.74	114.38	95.55	55.16	46.30	150.72	101.19	−5.05	−28.36	78:35:53	69:03:43	32:45:25	31:02:17	46.75	37.22	0.91	−0.81
Decitabine	Target Mol	0.74	122.51	109.26	97.17	68.10	172.12	137.26	105.46	28.98	42:05:06	106:37:57	58:35:58	41:39:02	10.24	74.79	26.75	9.81
A2 untreated mock-infected cells		0.73	146.42 ± 9.06				N.D.				N.D.				N.D.			
A2 untreated infected cells		0.73	38.01 ± 3.00				N.D.				33:04:09				N.D.			
Gemcitabine	Target Mol	0.73	129.33	121.67	97.34	45.70	240.22	220.08	156.07	20.23	69:52:01	63:04:07	70:06:21	36:30:58	38.38	31.58	38.62	5.03
Cidofovir	Target Mol	0.73	34.46	41.04	36.73	35.92	−9.35	7.98	−3.37	−5.49	32:43:20	36:28:21	36:30:37	33:49:02	1.23	4.98	5.02	2.33
Telbivudine	Target Mol	0.73	34.09	35.57	33.98	33.41	−10.31	−6.43	−10.62	−12.10	30:24:48	31:52:08	33:40:39	33:54:47	−1.08	0.38	2.19	2.42
Sofosbuvir	Target Mol	0.73	35.77	34.00	34.62	33.15	−5.89	−10.55	−8.92	−12.78	33:19:36	32:03:00	30:19:53	30:46:50	1.84	0.56	−1.16	−0.71
Favipiravir	Target Mol	0.73	45.22	34.29	30.23	34.02	18.95	−9.79	−20.47	−10.50	35:46:39	30:56:40	28:26:22	32:07:41	4.29	−0.55	−3.05	0.64
Arbidol	Target Mol	0.73	21.90	37.15	30.50	32.27	−42.38	−2.28	−19.77	−15.10	29:57:22	34:39:23	30:17:39	30:33:03	−1.53	3.17	−1.20	−0.94
DMXAA	Target Mol	0.73	40.75	35.18	29.18	36.33	7.19	−7.45	−23.23	−4.42	27:48:47	28:31:30	29:32:20	31:11:59	−3.68	−2.96	−1.95	−0.29
25-hydroxycholesterol	Cayman Europe	0.73	26.08	22.82	26.23	32.94	−31.40	−39.98	−31.00	−13.36	32:01:30	26:34:31	31:03:54	33:18:11	0.53	−4.91	−0.43	1.81
Proguanil	BIONET / Key Organics Ltd.	0.73	34.42	25.80	33.49	31.77	−9.44	−32.12	−11.89	−16.43	33:44:21	28:58:16	32:57:16	30:15:33	2.25	−2.52	1.46	−1.23
Nelarabine	AK Scientific, Inc.	0.73	30.10	27.45	28.15	34.38	−20.82	−27.77	−25.95	−9.56	29:56:14	28:35:11	27:17:35	31:39:20	−1.55	−2.90	−4.20	0.17
Fluorouracile	AK Scientific, Inc.	0.73	35.76	33.34	30.24	36.59	−5.91	−12.29	−20.44	−3.74	28:55:46	28:58:17	27:42:45	31:51:42	−2.56	−2.52	−3.78	0.37
2′-C-methylcytidine	Ark Phar, Inc.	0.73	32.38	31.50	34.42	36.75	−14.82	−17.12	−9.45	−3.33	30:04:02	31:56:57	31:51:26	33:33:39	−1.42	0.46	0.37	2.07
Eflornithin (dfmo)	Sigma	0.73	36.12	32.43	33.31	34.90	−4.99	−14.68	−12.37	−8.18	29:22:23	30:34:14	31:31:43	31:13:14	−2.12	−0.92	0.04	−0.27
Pravastatin	Sigma	0.73	31.27	33.45	36.29	32.66	−17.73	−11.99	−4.54	−14.09	29:58:56	29:52:34	30:38:38	31:06:08	−1.51	−1.61	−0.85	−0.39
Fluvastatin	Sigma	0.73	16.39	15.74	19.93	29.11	−56.89	−58.58	−47.57	−23.41	16:48:54	16:34:24	21:53:32	28:40:01	−14.67	−14.92	−9.60	−2.82
Simvastatin	Sigma	0.73	23.44	28.62	33.11	34.55	−38.34	−24.72	−12.89	−9.11	24:01:37	28:39:11	29:49:59	31:03:16	−7.46	−2.84	−1.66	−0.44
Atorvastatin	Sigma	0.73	15.15	21.30	30.83	36.62	−60.13	−43.97	−18.89	−3.67	16:19:45	22:17:20	29:43:25	31:49:47	−15.16	−9.20	−1.77	0.34
A3 untreated mock-infected cells		0.7	136.03 ± 11.19				N.D.				N.D.				N.D.			
A3 untreated infected cells		0.7	49.72 ± 1.34				N.D.				31:53:38				N.D.			
Brivudine	MedChemExpress	0.7	53.56	53.58	54.40	50.35	10.10	10.15	12.32	1.68	32:17:37	33:47:56	33:09:00	31:41:08	−0.13	1.38	0.73	−0.74
BAY 57–1293	MedChemExpress	0.7	100.32	62.45	50.45	50.31	133.13	33.49	1.94	1.57	130:02:11	42:46:56	30:20:20	32:00:42	97.61	10.36	−2.08	−0.41
A4 untreated mock-infected cells		0.61	147.97 ± 5.78				N.D.				N.D.				N.D.			
A4 untreated infected cells		0.61	55.26 ± 5.74				N.D.				45:06:29				N.D.			
Chloroquine	Sigma	0.61	55.41	43.43	40.23	43.76	0.40	−31.11	−39.53	−30.26	57:21:37	45:32:25	42:11:31	43:08:51	12.25	0.43	−2.92	−1.96
Hydroxychloroquine sulfate	MedChemExpress	0.61	60.03	49.60	42.39	49.96	12.55	−14.90	−33.85	−13.95	58:09:18	52:26:41	40:18:29	42:33:29	13.05	7.34	−65.58	−2.55
A5 untreated mock-infected cells		0.66	149.38 ± 8.96				N.D.				N.D.				N.D.			
A5 untreated infected cells		0.66	53.33 ± 7.05				N.D.				42:10:51				N.D.			
Aphidicolin	Santa Cruz Biotechnology	0.66	154.19	132.47	131.57	59.30	265.34	208.20	205.81	15.71	137:40:02	103:18:19	91:15:23	45:44:20	95.49	59.69	47.64	2.12
A6 untreated mock-infected cells		0.78	29.88 ± 4.16				N.D.				N.D.				N.D.			
A6 untreated infected cells		0.78	109.64 ± 4.03				N.D.				30:12:49				N.D.			
Brequinar	Adooq Bioscience	0.78	8.93	19.30	33.42	30.61	−55.11	−27.84	9.33	1.94	0:22:31	25:28:27	36:12:01	33:25:59	−29.84	−4.74	5.99	3.22
Ribavirin	MedChemExpress	0.78	94.17	51.39	25.11	31.45	169.13	56.60	−12.55	4.13	57:34:04	49:31:47	28:18:30	32:55:08	27.35	19.32	−1.91	2.71

## 2 Experimental Design, Materials and Methods

1 Cell line and EHV-1 strain

Equine dermal fibroblasts (E. Derm, NBL-6 ATCC® CCL-57, Manassas, VA) were cultivated in Eagle's Minimum Essential Medium (EMEM; ATCC®) supplemented with 10 % foetal bovine serum (Eurobio, Courtaboeuf, France), 100 IU/mL penicillin, 0.1 mg/mL streptomycin and 0.25 μg/mL amphotericin B (Eurobio) at 37 °C and 5 % CO2. The screening of antiviral compounds was performed against EHV-1 Kentucky D (KyD) strain (ATCC® VR700™) at a MOI of 0.01.

1 Compounds

Three different libraries including 2,891 compounds were screened in this study: i) 1,199 compounds from the Prestwick® Chemical Library (Prestwick Chemical, Illkirch, France), containing mostly US Food and Drug Administration approved drugs provided at 2 mg/mL in DMSO; ii) 1,651 compounds from the Centre d'Etudes et de Recherche sur le Médicament de Normandie (CERMN, Caen, France) provided at 10 mM in DMSO; iii) 41 compounds dissolved at 10 mM in DMSO (named herein in-house antiviral library) selected for their effects against different human viruses .

1 Screening of compound libraries using the RTCA system

The screening was performed with the RTCA MP system (ACEA Biosciences Inc., San Diego, CA, USA) as previously described [2]. This system measured the impedance signal by the electronic sensor analyser with 6 independent positions for 96-well E-plates (ACEA Biosciences). The impedance signal was derived in a dimensionless value, called Cell Index (CI) and graphically represented [2].

The background readings were obtained with EMEM supplemented in each well of the E-plate view PET (ACEA Biosciences). Cells were then seeded at a density of 1.2 × 10^4^ per well incubated at room temperature for 30 min, and then placed onto the RTCA-MP station located in the incubator (adapted from [6]). CI values were measured automatically every minute during the first 10 hrs, and then every 15 min for the next 14 hrs.

After 24 hrs of incubation, medium was removed and cells were infected with EHV-1 KyD strain with or without chemical compounds. Control cells were treated with 0.5 % DMSO in presence or absence of the virus. Plates were put back onto the RTCA MP station, and the CI values were recorded every 10 min during 120 h.

The screening was performed under blind conditions and 80 compounds were tested by plate at a final concentration of 10 µg/mL (Preswitck® Chemical Library), 10 µM (CERMN library) or 50, 10, 2 and 0.4 µM (in-house antiviral library) in 0.5 % DMSO. Each plate includes the controls required for calculation of the Z’-factor [5] based on AUC_n_ values between 0 and 120 h post-infection (hpi). The formula is: Z’ ==1–3 × (σ^+^ + σ^-^) ÷(μ^+^ – μ^-^), where σ^+^ and σ^-^ correspond to standard deviations of AUC_n_ for uninfected and infected cells with DMSO, respectively; and μ^+^ and μ^-^ correspond to means of AUC_n_ for uninfected and infected cells with DMSO, respectively. Only plates presenting a Z’-factor higher than 0.5 were validated for further analysis as previously described by Thieulent et al. (2019) [2]. CI values were first normalised using the RTCA software version 2.0 (ACEA Biosciences) prior to data analysis according to the manufacturer's recommendations. For each compound, the area under normalised Cell Index (CI) curves was calculated from 0 to 96 hrs post-infection (hpi) (AUC_n_; [3]). The%AUC_n_ was calculated with formula:

$\%\text{AUCn}=\frac{\left(\text{AUCn}\;\text{compound}-\;\text{AUCn}\;\text{untreated}\;\text{cells}\;\text{infected}\right)}{\text{AUCn}\;\text{untreated}\;\text{cells}\;\text{infected}}\times 100$. The time required for the CI to decrease by 50% after virus infection was also determined (CIT_50_; [4]), and compared with untreated cells infected with EHV-1 KyD strain (ΔCIT_50_ = CIT_50_ compound - CIT_50_ untreated cells infected).

## Declaration of Competing Interest

The authors declare that they have no known competing financial interests or personal relationships which have, or could be perceived to have, influenced the work reported in this article.

## Appendix Supplementary Materials

Image, application 1

Image, application 2

Image, application 3

Image, application 4
