# Supplementary material for: Screening of potential antiviral molecules against equid herpesvirus-1 using cellular impedance measurement: Dataset of 2,891 compounds
Source: Data Brief. 2020 Nov 5;33:106492. doi: 10.1016/j.dib.2020.106492 (PMC7689375; doi:10.1016/j.dib.2020.106492)
Supplement: Supplementary file 4 [file mmc4.pdf]

Antiviral Research

Identification of antiviral compounds against equid herpesvirus-1 using real-time cell assay screening: efficacy of decitabine and valganciclovir alone or in combination.

--Manuscript Draft--

**Manuscript Number:**	AVR-D-20-00404R1
**Article Type:**	Research paper
**Section/Category:**	Veterinary viral diseases
**Keywords:**	Real-time cell assay; chemical library screening; Antiviral; Equid herpesvirus-1; ganciclovir; decitabine
**Manuscript Region of Origin:**	Europe
**Abstract:**	Equid herpesvirus-1 infections cause respiratory, neurological and reproductive syndromes. Despite preventive treatments with vaccines, resurgence of EHV-1 infection still constitutes a major threat to equine industry. However, no antiviral compound is available to treat infected horses. In this study, 2,891 compounds were screened against EHV-1 using impedance measurement. 22 compounds have been found to be effective in vitro against EHV-1. Valganciclovir, ganciclovir, decitabine, aphidicolin, idoxuridine and pritelivir (BAY 57-1293) are the most effective compounds identified, and their antiviral potency was further assessed on E. Derm, RK13 and EEK cells and against 3 different field strains of EHV-1 (ORF30 2254A/G/C) . We also provide evidences of synergistic interactions between valganciclovir and decitabine in our in vitro antiviral assay as determined by MacSynergy II, isobologramm and Chou-Talalay methods. Finally, we showed that deoxycytidine reverts the antiviral effect of decitabine, thus supporting some competition at the level of nucleoside phosphorylation by deoxycytidine kinase and/or DNA synthesis. Deoxycitidine analogues, like decitabine, is a family of compounds identified for the first time with promising antiviral efficacy against herpesviruses.

Powered by Editorial Manager® and ProduXion Manager® from Aries Systems Corporation

Title:

Identification of antiviral compounds against equid herpesvirus-1 using real-time cell assay screening: efficacy of decitabine and valganciclovir alone or in combination.

Formatted: Numbering: Continuous

List of authors:

Côme Thieulent [1,2], Erika Hue [1,2,3], Gabrielle Sutton [1,2], Christine Fortier [1,2,3], Patrick Dallemagne [4], Stephan Zientara [5], Hélène Munier-Lehmann [6], Aymeric Hans [7], Romain Paillot[1,2], Pierre-Olivier Vidalain [8,9] and Stéphane Pronost [1,2,3,§]

[1] LABÉO Frank Duncombe, 14280 Saint-Contest, France.

[2] Normandie Univ, UNICAEN, BIOTARGEN EA7450, 14280 Saint-Contest, France.

[3] Normandie Univ, UNICAEN, ImpedanCELL, 14280 Saint-Contest, France.

[4] Normandie Univ, UNICAEN, CERMN, 14000 Caen, France.

[5] Université Paris-Est, Laboratoire de Santé Animale, ANSES, INRAE, ENVA, UMR 1161 Virologie, 94700 Maisons-Alfort, France.

[6] Institut Pasteur, Unité de Chimie et Biocatalyse, CNRS UMR 3523, 75015 Paris, France.

[7] ANSES, Laboratoire de santé animale, site de Normandie, PhEED Unit, 14430 Goustranville, France.

[8] CIRI, Centre International de Recherche en Infectiologie, Univ Lyon, Inserm U1111, Université Claude Bernard Lyon 1, CNRS UMR5308, ENS de Lyon, F-69007, Lyon, France.

[9] Equipe Chimie et Biologie, Modélisation et Immunologie pour la Thérapie (CBMIT), Université Paris Descartes, CNRS UMR 8601, 75006 Paris, France.

[§] Corresponding Author
Tel: +33 (0) 2 31 47 19 54
Fax: +33 (0) 2 31 47 19 00
Email: stephane.pronost@laboratoire-labeo.fr
LABÉO Frank Duncombe
1 route de Rosel, 14280 SAINT-CONTEST, France

Abstract

Equid herpesvirus-1 infections cause respiratory, neurological and reproductive syndromes. Despite preventive treatments with vaccines, resurgence of EHV-1 infection still constitutes a major threat to equine industry. However, no antiviral compound is available to treat infected horses. In this study, 2,891~~7~~ compounds were screened against EHV-1 using impedance measurement. 22 compounds ~~were identified~~have been found to be effective *in vitro* against EHV-1. ~~(val)~~Valganciclovir, ganciclovir, decitabine, aphidicolin, idoxuridine and pritelivir (BAY 57-1293) are the most effective compounds identified, and their antiviral potency ~~were demonstrated~~was further assessed on E. Derm, RK13 and EEK cells and against 3 different field strains of EHV-1 (ORF30 2254A/G/C). ~~Valganciclovir~~We also provide evidences of synergistic interactions between valganciclovir and decitabine ~~are the only combination tested that showed a synergistic effect~~ in our *in vitro* ~~using~~antiviral assay as determined by MacSynergy II, isobologramm and Chou-Talalay methods. Finally, ~~this study demonstrated that~~we showed that deoxycytidine reverts the antiviral effect of decitabine ~~needs to be~~

~~phosphorylated~~, thus supporting some competition at the level of nucleoside phosphorylation by deoxycytidine kinase ~~in order to be active against EHV-1~~and/or DNA synthesis. Deoxycitidine analogues, like decitabine, is a family of compounds identified for the first time with promising antiviral efficacy against herpesviruses.

Keywords

Real-time cell assay; Chemical library screening; Antiviral; Equid herpesvirus-1; ganciclovir; decitabine; synergism

1. Introduction

Herpesviruses (order *Herpesvirales*, family *Herpesviridae*) are enveloped viruses with a linear, double-stranded DNA genome of 125-290 kb. Among the five equid herpesviruses (EHV-1 to 5) frequently isolated in horses, EHV-1 is the most pathogenic and is endemic worldwide. EHV-1 infection in horses is associated with several clinical signs of disease, from usually mild respiratory distress, cough and discharge, to more severe secondary forms of diseases such as abortion, neonatal foal death and equine herpes myeloencephalopathy (EHM) (Allen, 2002). The prevalence of latent EHV-1 is estimated to be greater than 60% in horse population (Lunn et al., 2009).

Several vaccines are available against EHV-1. Their use ~~reduce~~reduces clinical signs of respiratory disease and virus shedding, which limits the extent of outbreaks. However, the protection provided against the secondary forms of the disease presents some limitations. While EHV-1 induced abortion storms have been ~~prevented~~reduced since the introduction of vaccination three decades ago, none of the commercially available EHV-1 vaccines have demonstrated its efficacy to prevent EHM. EHV-1 vaccine coverage is often too low to

provide effective herd immunity. In this context, outbreaks still occur worldwide in horse populations. A recent outbreak reported in France in 2018 (Sutton et al., 2019), led to the cancellation of more than 200 horse competitions, thus generating large economic losses for the French equine industry. To complement prevention measures, such as vaccination and biosecurity, the use of antiviral treatment is sometimes considered ~~to prevent~~ for the treatment of severe ~~forms of~~ EHV-1 ~~induced disease~~diseases, especially EHM. The occasional use of aciclovir during EHV-1 outbreaks has been reported (Friday et al., 2000; Henninger et al., 2007; Murray et al., 1998) but the therapeutic efficacy of this compound is difficult to assess in the absence of untreated animals as a control. Two experimental infections in horses treated with valaciclovir, an aciclovir pro-drug, have also shown divergent results (Garre et al., 2009; Maxwell et al., 2008).

EHV-1 is an alphaherpesvirus genetically closely related to herpes simplex virus type 1 (HHV-1) and varicella zoster virus (HHV-3) for which antiviral therapies are available. However, the emergence of human herpesvirus strains resistant to antiviral treatments, such as aciclovir, has motivated researches for new antiviral therapies (Jiang et al., 2016) and helicase primase inhibitors seem to be good candidates (James et al., 2015; Kleymann et al., 2002). Over the last two decades, drug repositioning has proven to be an effective strategy to meet therapeutic needs with nearly a hundred drugs repositioned since (Jourdan et al., 2020). Even in absence of approved EHV-1 antiviral treatment for practitioners, few antiviral molecules have been studied against EHV-1 *in vitro* and correspond to those already used in human medicine against herpesviruses such as aciclovir, ganciclovir, cidofovir and penciclovir (Maxwell, 2017; Vissani et al., 2016). Other compounds such as aphidicolin (Goodman et al., 2007), A-5021 (Glorieux et al., 2012), quercetin (Ferreira et al., 2018; Gravina et al., 2011) and the histone demethylase inhibitor OG-L002 (Tallmadge et al., 2018) have been studied

against EHV-1 in different cell culture models. However, these molecules have never been tested in a standardised cellular model allowing proper comparisons.

We have recently developed a standardised Real-Time Cell Analysis (RTCA) model for evaluating the effect of antiviral compounds against EHV-1. This system relies on the measurement of cellular impedance in culture wells, which reflects cellular adhesion and proliferation. Results are expressed as Cell Index (CI) that enables a standardised and accurate analysis of EHV-1 cytopathic effects. This system has proven successful to determine the efficacy of molecules against EHV-1 such as spironolactone (Thieulent et al., 2019). In the present work, a chemical library of 2,891~~7~~ compounds comprising new chemical entities and FDA-approved drugs has been screened by impedancemetry to identify compounds against the EHV-1 Kentucky D (KyD) reference strain. As some associations between a DNA polymerase (ORF30) genotype (G/A at position 2254) and the type of disease have been reported by several studies (Goodman et al., 2007; Lunn et al., 2009; Nugent et al., 2006; Pronost et al., 2010), active molecules identified were subsequently tested against a panel of EHV-1 strains (A_{2254} or G_{2254}), including the newly identified EHV-1 ORF30 variant (C_{2254}) (Paillot et al., 2020). Decitabine was one of the most effective molecules identified, and the mode of action of this cytidine analogue has been further investigated.

2. Materials and methods

2.1. Cell lines

Equine dermal fibroblasts (E. Derm, NBL-6 ATCC® CCL-57, Manassas, VA), equine embryonic kidney cells (EEK, kindly provided by Merial, France) and rabbit kidney cells (RK13, ATCC® CCL-37™) were used in this study. E. Derm cells were maintained in Eagle's Minimum Essential Medium (ATCC®) and seeded at 1.2×10^4 cells/well in 96-well plates. EEK cells were maintained in MEM Alpha (Biowest, Nuaillé, France) supplemented with 2%

Lactalbumin hydrolysate (Sigma, St. Quentin Fallavier, France), 1% L-glutamine (Eurobio, Courtaboeuf, France), 0.5% D-Glucose (Sigma) and seeded at 1.2×10^4 cells/well in 96-well plates. RK13 cells were maintained in EMEM with Earle’s salts (Eurobio) supplemented with 1% L-glutamine (Eurobio) and seeded at 4.8×10^4 cells/well for 96-well plates. All media contained 10% fetal bovine serum (Eurobio), 100 IU/mL penicillin, 0.1 mg/mL streptomycin and 0.25 µg/mL amphotericin B (Eurobio) and were cultivated at 37 °C and 5% CO_2.

2.2. EHV-1 strains

The EHV-1 Kentucky D (KyD) strain (ATCC® VR700™) was used as the EHV-1 reference strain for compound screening and subsequently to confirm the antiviral effect of selected hits in the different cell lines. In addition, three French EHV-1 strains were also used in this study, including the ORF30 G_{2254} EHV-1 strain (FR-38991) isolated in 2009 from a horse with neurological disorders (LABÉO, France; nasal swab), the ORF30 A_{2254} EHV-1 strain (FR-6815) isolated in 2013 from lung biopsies of an aborted foetus (LABÉO, France) and the ORF30 C_{2254} EHV-1 strain (FR-56628) isolated in 2018 from PBMC of a horse with respiratory disorders (LABÉO, France) (Paillot et al., 2020). E. Derm and RK13 cells were infected with the KyD strain at MOIs of 0.01 and 0.04, respectively. EEK cells were infected with the four different EHV-1 strains at a MOI of 0.05.

2.3. Compounds

This study includes 2,891~~7~~ compounds from three different libraries: i) 1,199~~200~~ compounds from the Prestwick® Chemical Library, containing mostly US Food and Drug Administration approved drugs (Prestwick Chemical, Illkirch, France) provided at 2 mg/mL in DMSO; ii) 1,651~~60~~ compounds from the Centre d’Etudes et de Recherche sur le Médicament de Normandie (CERMN, Caen, France) provided at 10 mM in DMSO; iii) ~~37~~41 compounds (called herein in-house antiviral library) selected for their effects against different human viruses and dissolved at 10 mM in DMSO (Supplementary Table 1). RG108

(MedChemExpress) was dissolved at 20 mM in DMSO. All compounds were stored at -20°C before used.

2.4. Screening of compound libraries using the RTCA system

The screening by impedancemetry was performed with EHV-1 KyD-infected E. Derm cells using the RTCA MP system (ACEA Biosciences, Montigny le Bretonneux, France Inc., San Diego, CA, USA) as previously described (Thieulent et al., 2019). ControlsControl cells were treated with 0.5% DMSO in presence or absence of the virus. The screening was performed under blind conditions and 80 compounds were tested by plate at a final concentration of 10 µg/mL (Preswitck® Chemical Library), 10 µM (CERMN library) or 50, 10, 2 and 0.4 µM (in-house antiviral library) in 0.5% DMSO. Each plate includes the controls required for calculation of the Z'-factor (Zhang et al., 1999). Only plates with a Z' factor upper than 0.5 were considered for further analysis as previously described by Thieulent et al. (2019). For each compound, the area under normalised Cell Index (CI) curves was calculated from 0 to 96 hours post-infection (hpi) (AUCn; (Pan et al., 2013). The time required for the CI to decrease by 50% after virus infection was also determined (CIT_{50}; (Fang et al., 2011), and compared with controls. All the details are presented in Data in Brief (Thieulent et al., *submitted*). Any increase in these two parameters reflects some protection of E. Derm cells from EHV-1 induced cytopathic effects. The cut-off determined for a molecule to be considered with an antiviral potential were (i) the AUCn increasing by 25%, and (ii) the CIT_{50} being delayed by >8 h as compared to non-treated cells (Thieulent et al., 2019).

Formatted: Font: Italic

After the screening, dose-response curves were obtained for each selected compound by using percentage of inhibition calculation. The following formula was used: Inhibition (%) = 100 × [1 – (a – b) ÷ (b – c)], where a corresponds to the value of infected cells treated with different concentrations of compounds, whereas b and c correspond to values obtained for mock-infected and mock-treated cells, respectively.

2.5. Viral quantitation by qPCR assay

Cells were seeded and treated in 96-well plates as described in part 2.4. At 48 hpi, plates were frozen at -20°C to allow virus load quantitation in culture wells. After one cycle of freeze/thaw, nucleic acids were extracted using the QIAamp® Viral RNA Mini Kit (Qiagen, Courtaboeuf, France) according to the manufacturer's instructions and stored at -20 °C until used. Quantitative PCR for EHV-1 was processed as previously described (Thieulent et al., 2019). Each thermal cycling was performed on a QuantStudio™ 12 K Flex Real- Time PCR System (Life Technologies).

2.6. Toxicity measurement

Cells were seeded in white opaque 96-well plates and after 24 h of culture, were treated with compounds. Cell viability was measured at 48 h post-treatment (hpt) by impedancemetry and ATP measurement using the CellTiter Glo® Luminescent Cell Viability Kit (CTG; Promega, Charbonnière-les-bains, France), according to the manufacturer's instructions. Luminescence signal was acquired using an Infinite® M200 luminometer (Tecan, Lyon, France).

2.7. Research of synergistic effects between compounds against EHV-1

Drug combinations were tested on EHV-1 KyD-infected E. Derm cells using impedancemetry as a read out. For each combination, the two selected drugs were prepared separately by 2-fold serial dilution and mixed in 96-well plates to create an 8 by 10 matrix of single and combined diluted drugs. For each compound, the dilution range was designed to have the ~~IC_{50}~~EC_{50} in the middle of the range, and the highest concentration inferior to the ~~I~~EC_{90}. In each plate, infected and non-infected cells with 1% DMSO were used as positive and negative controls, respectively. Synergistic or antagonistic effects were determined with the MacSynergy II program using first the Bliss independence model (Prichard and Shipman, 1990) applied on AUC_n values. This software calculated the volume of synergy/antagonism produced by the drug combination in a 95% confidence interval. Volumes were given as the

area under a dose-response curve in the two dimensional situation (µM² %) and interpretation was made as previously described by Prichard et al. (1990). Values of 0-25, 25-50, 50-100, and >100 µM² % in either a positive or negative direction were defined as additive, minor synergy or antagonism, moderate synergy or antagonism, and strong synergy or antagonism, respectively.

Isobologram analysis and the Chou-Talalay method using the Loewe additivity model were used to confirm synergistic effect firstly observed by MacSynergy II program. Isobolograms were built as previously described by Feng et al. (2009) from ~~IC_{50}~~EC_{50} values obtained by impedance measurement. The Chou-Talalay method is based on the median-effect equation and computed by CompuSyn software version 1.0 (ComboSyn, Inc., Paramus, New Jersey) (Chou and Talalay, 1984). The software extrapolated a combination index representing the interaction between two drugs from the percentage of inhibition of $\log_{10}$ viral genome copies number produce in presence of each drug alone and in combination. The weighted average combination index (CI_{wt}) value was calculated as previously described (Drouot et al., 2016).

2.8. Statistical analysis

~~IC_{50}~~EC_{50} and CC_{50} values were calculated using a non-linear regression dose response inhibition curve (GraphPad Prism® software 6.0; La Jolla, CA, USA). The Selectivity Index (SI) was determined for each compound using the following formula: SI = CC_{50}/~~IC_{50}.~~ ~~IC_{50}~~EC_{50}. Spearman’s correlation test and values ~~were compared by~~comparison using ANOVA with Tukey post hoc test were both statistically evaluated using GraphPad Prism® software.

3. Results

3.1. Screening and selection of the most effective compounds against EHV-1

~~The~~To identify novel antiviral compounds against EHV-1, three chemical libraries (*i.e.* Prestwick~~ chemical~~® Chemical, CERMN and in-house antiviral libraries~~y~~; see Material and Methods for details) were screened ~~against EHV-1~~by impedance-based Real-Time Cell Analysis (RTCA) using equine dermal (E. Derm) cells infected with EHV-1 KyD strain as ~~described in the Materials and Methods section.~~model (Supplementary Figure 1). Z'-factor values of the 38 screening 96-well plates were between 0.52 to 0.91, with a median of 0.71, insuring the robustness of our assay. ~~From~~Based on CIT_{50} and AUC_n values, 25 out of 2,891~~7~~ compounds~~ tested, 25~~ were identified with a potential antiviral activity (hit detection rate of 0.9%) against EHV-1 (14, 1 and 10 in the three chemical libraries, respectively).

These molecules were then evaluated in dose-response assays on E. Derm cells by two-fold serial dilution (50 to 0.1 µM) by qPCR assay and impedance measurement. Antiviral properties were confirmed for 22 out of the 25 compounds (Table 1~~).~~), and EC_{50} values were precisely determined by both qPCR and RTCA for 16 of them (EC_{50}s<50 µM). The R factor of 0.841 and P-value <0.001 obtained by Spearman's correlation showed a good correlation between EC_{50} values obtained by qPCR and RTCA for these 16 molecules (Figure 1). Among these compounds, eight were selected as they complied with more stringent criteria: (i) the absence of toxicity on E. Derm cells at all the concentrations tested (CC_{50} > 50µM) and (ii) ~~IC_{50}~~EC_{50} values below 50 µM at all time points between 48 and 120 hpi (Figure ~~1).~~2A). Dose-responses curves obtained by qPCR and RTCA at 48 hpi for the eight compounds are presented in Figure 2B. Three of these compounds were acyclic guanosine analogues (aciclovir, ACV; ganciclovir, GCV; and ganciclovir prodrug valganciclovir, VGCV) ~~inhibitors of~~inhibiting the viral DNA polymerase, two were deoxycytidine analogues (decitabine, DTB; gemcitabine, GTB) used for ~~inhibitors of cancers~~tumor growth inhibition

Formatted: Font color: Auto

Formatted: English (United Kingdom)

by ~~cellular DNA~~ incorporation in cellular DNA, and one was a deoxyuridine analogue (idoxuridine, IDU) ~~also~~ targeting~~an inhibitor of the~~ viral DNA synthesis~~ polymerase~~. Aphidicolin (APD), a tetracyclic diterpene antibiotic, and pritelivir (BAY 57-1293), an inhibitor of the HHV-1 helicase-primase complex, were also selected.

3.2. Efficacy of eight selected compounds on different EHV-1 strains and cell lines

The antiviral activity of the eight selected compounds was further studied against EHV-1 KyD using two other cellular models: an equine cell line (EEK) and a ~~rodent~~rabbit cell line (RK13) and results were compared with data obtained on E. Derm cells (~~Table 2~~Figure 3A). Gemcitabine did not show any antiviral effect at tested concentrations on EEK and RK13 cells (~~IC_{50}~~EC_{50} > 50 µM). ~~The~~With EC_{50} ranging from 21.6 to 31.0 µM, aciclovir shows a weak antiviral activity ~~of aciclovir obtained~~ on ~~RK13 cells (31.0 µM) was comparable with~~ the ~~result obtained on E. Derm cells (30.1 µM) and showed no effect on EEK cells (IC_{50} > 50 µM).~~three cell models. The six other compounds (aphidicolin, pritelivir, decitabine, idoxuridine, ganciclovir and valganciclovir) showed a good efficacy on the three cell lines.

The efficacy of the eight selected compounds was tested against three different EHV-1 strains with distinct ORF30 genotypes (A/G/C_{2254}) on EEK cells (~~Table 3~~Figure 3B). This study confirmed the low activity of gemcitabine (~~IC_{50}~~EC_{50} > 50 µM). Aciclovir showed a lower activity against the FR-38991 strain (G_{2254}: 36.0 µM) when compared with its activity against FR-6815 (A_{2254}: 14.2 µM) or FR-56628 (C_{2254}: 7.9 µM). Quite similarly, pritelivir and idoxuridine showed a lower activity against FR-38991 strain (2.7 µM and 5.7 µM, respectively) when compared with its activity against FR-6815 (0.9 µM and 1.5 µM, respectively) or FR-56628 (1.0 µM and 1.5 µM, respectively). Consistent results were obtained with the other molecules across all EHV-1 strains.

3.3. Research of synergistic effect and antiviral activity of the valganciclovir/decitabine combination

Dual-combinations were tested between valganciclovir, one of the best ~~candidate~~candidates, and four other compounds (aphidicolin, pritelivir, decitabine and idoxuridine) for synergistic or antagonistic effects against EHV-1. Using MacSynergy II analysis, only the valganciclovir/decitabine combination showed a synergistic effect that is illustrated by the strong signal above additive effects in the matrix of drug interactions (Figure ~~2A~~4A). The synergy volume of 63.24 µM² % obtained supports a moderate synergy (Table ~~1~~2). The peak of synergy was reached when both compounds were used at 0.63 µM (1:1 ratio). Likewise, evaluation of the combination valganciclovir/decitabine by the isobologram method indicated synergy with ADA values of -0.30 ($p < 0.001$) (Figure ~~2B~~4B). Results obtained by impedancemetry were also confirmed by viral genome copy number measurement at 48 hpi and median-effect analysis for concentrations of valganciclovir and decitabine used alone or in combination at a 1:1 ratio. A synergistic effect was observed for the valganciclovir/decitabine combination as assessed by a weighted average combination index (CI_{wt}) of 0.20 (Figure ~~2C~~4C). The three other combinations (valganciclovir/~~APB~~aphidicolin, valganciclovir/ pritelivir, valganciclovir/ idoxuridine) tested were additive when measured by MacSynergy II method (Table ~~1~~2) and were not tested by isobologram nor median-effect analysis. No cytotoxicity was observed at the maximal drug combinations tested for the four different combinations (Supplementary Figure ~~1~~2).

3.4. Decitabine pre-treatment did not confer cell resistance to EHV-1 replication.

Although valganciclovir was developed as an antiviral against herpesviruses in the first place, this is not the case of decitabine. Indeed, decitabine is an anticancer agent which induces hypomethylation after integration in cellular DNA (Liu et al., 2007). To evaluate whether decitabine integration in target cell DNA provides protection from EHV-1 infection, cells

were treated overnight with decitabine before infection and/or just after infection. Both results obtained by cell impedance measurement (Figure ~~3A~~5A) and EHV-1 viral load measurement (Figure ~~3A~~5B) showed that decitabine pre-treatment did not protect cells from CPE formation and virus replication. A post-infection ~~decitabine~~ treatment with decitabine was required to observe some significant inhibition of EHV-1 replication. The effect of RG108, another well know DNA methyltransferase inhibitor, was then tested against EHV-1 on E. Derm cells. RG108 did not show any antiviral effect when assessed by impedance measurement (Figure ~~4A~~6A) or virus load quantitation (Figure ~~4B~~6B). Altogether, this suggests that cellular DNA hypomethylation does not account for the inhibition of EHV-1 by decitabine.

3.5. Deoxycytidine competitively inhibits the antiviral effect of decitabine

~~decitabine~~Decitabine is a deoxycytidine analogue and a pro-drug that must be successively phosphorylated by components of the deoxyribonucleoside salvage pathway involving deoxycytidine kinase (DCK~~) to be integrated in target cell DNA~~), CMP monophosphate kinase (in particular CMPK1) and nucleotide diphosphate kinases (Momparler, 2005; Stresemann and Lyko, 2008). ~~Interestingly, it~~It has been shown that high levels of deoxycytidine (dC) can reverse the anticancer activity of gemcitabine, another ~~deoxycytidine~~dC analogue, by competition for DCK-mediated phosphorylation, the rate-limiting step in dC phosphorylation to dCTP (Halbrook et al., 2019). ~~EHV-1-infected E.~~We thus tested if dC could similarly reverse the inhibitory effects of decitabine on EHV-1. Infected E. Derm cells were treated with decitabine in the presence of high concentrations of ~~deoxycytidine (~~dC~~)~~ or other nucleosides including cytidine, uridine, adenosine, guanosine (Figure ~~5A~~7A). Of all tested nucleosides, only dC blocked the antiviral activity of decitabine. This result was confirmed by microscopic observations and impedancemetry as dC reversed the ~~cell-protective~~antiviral effect of decitabine against EHV-1 (Figure ~~5B and C).~~ 7B and 7C).

Altogether, these results demonstrate a competition of decitabine and dC for the same metabolic pathway in our *in vitro* infection model.

4. Discussion

In this study, 2,891~~7~~ compounds were screened against EHV-1 by impedancemetry as previously described (Thieulent et al., 2019), and 22 compounds were identified for their antiviral properties against this virus. AUC_n values coupled to CIT_{50} calculation were the two major criteria for filtering raw data and identify hits. The antiviral effect of selected compounds was confirmed by dose-response assay using both impedancemetry and viral load quantitation. As the readouts were not the same, ~~IC_{50}~~EC_{50} values obtained with these two methods differed as previously reported (Piret et al., 2016; Thieulent et al., 2019). ~~Among~~However, the good correlation observed between EC_{50} values obtained by RTCA and qPCR demonstrates the pertinence of RTCA for antiviral evaluation. Among the 22 compounds ~~effective against~~inhibiting EHV-1, eight molecules were selected for further evaluations using stringent criteria, including ~~IC_{50}~~EC_{50} values below 50 µM over time and lack of cytotoxicity when used at 50 µM.

Ganciclovir and aciclovir are approved medications to treat herpesviruses and were previously shown to be effective against EHV-1 *in vitro* (Garre et al., 2007; Thieulent et al., 2019). ~~valganciclovir~~Valganciclovir, the pro-drug and valine ester of ganciclovir, presents here an antiviral activity against EHV-1 similar to ganciclovir. ~~Idoxuridine is a well know antiviral compound against herpesviruses in human such as HHV-1 and HHV-2 and also different animal species such as feline herpesvirus type-1 (De Clercq and Li, 2016; Maggs and Clarke, 2004).~~ Pritelivir is another antiviral drug developed to treat herpesviruses. It is an inhibitor of the helicase-primase complex of herpesviruses discovered in 2002 (Kleymann et al., 2002). ~~It does not require any activation step unlike other nucleoside analogues such as ganciclovir.~~ The antiviral effect of pritelivir was previously reported against HHV-1 and HHV-2 (Betz et

al., 2002). However, this study is the first demonstrating the antiviral effect of this molecule against ~~EHV-1. Decitabine and gemcitabine are two deoxycytidine analogues~~equid herpesviruses and in particular against EHV-1. It would be interesting to evaluate the antiviral effect of these compounds against other equid herpesviruses, such as EHV-3. Idoxuridine is also a well-known antiviral compound against human herpesviruses such as HHV-1 and HHV-2, and is also active against different animal herpesviruses such as feline herpesvirus type-1 (De Clercq and Li, 2016; Maggs and Clarke, 2004). Interestingly, idoxuridine is one of the three deoxyuridine analogues, together with brivudine and trifluridine, which have been used for decades against herpes simplex viruses (De Clercq and Li, 2016). Idoxuridine and trifluridine have showed a good efficacy against EHV-1 without toxicity in our cellular model, whereas brivudine was inactive (data not shown). Brivudine is the only one that needs to be specifically phosphorylated by viral thymidine kinase (TK) to become active (De Clercq and Li, 2016), suggesting that EHV-1 TK is unable to phosphorylate brivudine, which is in line with previous reports (De Clercq, 1984; Kit et al., 1987). Maribavir is also a new nucleoside analogue in development against human cytomegalovirus (HHV-5, a betaherpesvirus) that was tested in our screen and was inactive against EHV-1 (data not show) (Price and Prichard, 2011). This result is in line with the lack of activity against the alphaherpesviruses HHV-1, HHV-2 and HHV-3 (Williams et al., 2003). Finally, our study showed that three deoxycytidine analogues, *i.e.* decitabine, gemcitabine and cytarabine, are all effective against EHV-1 infection *in vitro*. Decitabine and gemcitabine are respectively used in the treatment of acute myeloid leukemia (He et al., 2017) and recurrent ovarian cancer (Berg et al., 2019~~), respectively. To~~). Gemcitabine was previously characterized for its antiviral activity against a broad spectrum of RNA viruses (Shin et al., 2018). It has also been reported to be effective against HHV-1 (Denisova et al., 2012). However, to our knowledge, ~~this~~our work is the first ~~report showing that deoxycytidine analogues inhibit~~reporting the

~~replication of a herpesvirus. In this study, we have discovered that~~antiviral effect of decitabine~~, gemcitabine and cytarabine are all effective against EHV-1 infection, at least *in vitro*.~~ against herpesvirus. Of these three ~~compounds~~deoxycitidine analogues, cytarabine has the lowest activity with an ~~IC_{50}~~EC_{50} of 4.1 µM as determined by qPCR assay on E. Derm cells. Decitabine and gemcitabine were more potent EHV-1 inhibitors in this cellular model with ~~IC_{50}s~~EC_{50}s of 1.1 µM and 0.7 µM, respectively.

Formatted: English (United States)

~~Quite surprisingly, brivudine and maribavir did not show any antiviral effect against EHV-1. Brivudine is one of the three deoxyuridine analogues, together with idoxuridine and trifluridine, that have been used for decades against herpes simplex viruses (De Clercq and Li, 2016). Idoxuridine and trifluridine have showed a good efficacy against EHV-1 without toxicity in our cellular model, whereas brivudine was inactive (data not shown). Brivudine is the only one that needs to be specifically phosphorylated by viral thymidine kinase (TK) to become active (De Clercq and Li, 2016), suggesting that EHV-1 TK is unable to phosphorylate brivudine, which is in line with previous reports (De Clercq, 1984; Kit et al., 1987). Maribavir is a new antiviral drug in development against human cytomegalovirus (HHV-5), a betaherpesvirus (Price and Prichard, 2011). This compound showed no antiviral effect against EHV-1 in our model (data not show), in line with the lack of activity against the alphaherpesviruses HHV-1, HHV-2 and HHV-3 (Williams et al., 2003).~~

The antiviral activity of the 8 most efficient molecules was also validated in three cell lines and against different strains of EHV-1. E. Derm cells and EEK cells are both equine cell lines and most adapted to identify new antiviral compounds in equid species, especially EEK that was derived from a horse foetus that is one of the target of EHV-1 (Léon et al., 2008; Smith et al., 2010). Even though RK13 cells are not equine cells, they have been most frequently used in EHV1 antiviral studies (Azab et al., 2010; de la Fuente et al., 1992; Gibson, 1992; Rollinson, 1987). All compounds except aciclovir and gemcitabine showed some consistent

antiviral activity in the three different cell lines. Aciclovir is the least active of the eight selected compounds, and was inactive when used with EEK cells. More surprisingly, although gemcitabine is very effective on E. Derm cells, it has no antiviral activity on RK13 and EEK cells. This suggests that gemcitabine is not properly phosphorylated ~~in~~by ~~-~~RK13 and EEK cells kinases. In line with this hypothesis, decitabine, which is structurally very close to gemcitabine and also needs to be phosphorylated, is less active on EEK and RK13 cells. Nevertheless, decitabine still exhibits a good efficacy in all three cellular models. The antiviral activity of the eight selected molecules was also evaluated on EEK cells infected with three different EHV-1 strains isolated during outbreaks in France. Each strain exhibits different nucleotide (A/G/C) at position 2254 of ORF-30 (DNA polymerase). ~~IC_{50}~~EC_{50} values of the compounds were close, independently of the strain used. This suggests that the mutations in the palm domain did not affect the effect of the selected molecule~~. This result was concordant~~ in agreement with ~~a~~ previous ~~report when~~reports comparing the susceptibility of A_{2254} and G_{2254} strains~~ A and G at position 2254 of ORF-30.~~ (Garre et al., 2007; Thieulent et al., 2019). Only aciclovir, pritelivir and idoxuridine were slightly less efficient on the FR-38991 (A_{2254}) strain. ~~Surprisingly, no~~No difference of susceptibility was observed between the three strains for aphidicolin treatment. This result differs from a previous report showing that a strain with the G_{2254} genotype is more sensitive to aphidicolin than a strain with the A_{2254} genotype (Goodman et al., 2007).

~~Ganciclovir is~~ In this study, ganciclovir and its prodrug valganciclovir are the most ~~potent of the few~~effective compounds ~~that were already known to be active~~ *in vitro* against EHV-1 ~~infection *in vitro* (Garre et al., 2007; Thieulent et al., 2019). In this study, valganciclovir was compared with ganciclovir in a standardized *in vitro* assay, and results showed similar activities against EHV-1. The~~. Interestingly, the pharmacokinetic of valganciclovir was previously studied in horse (Carmichael et al., 2013), showing 40% bioavailability after oral

administration.~~Actually, this~~ This positions ~~the~~ valganciclovir as the best candidate in our short list of active molecules for ~~therapy of EHV-1~~ treating horses infected ~~horses~~by EHV-1 even if the cost of the molecule could be a limitation. ~~In order to enhance~~Nevertheless, our results should be considered only as a first step in this direction as the predictive value of *in vitro* models based on continuous cell lines is questionable. For example, the antiviral effect of ~~valganciclovir, combination of valganciclovir with aphidicolin, pritelivir, decitabine and idoxuridine were analysed. Only the valganciclovir/decitabine combination showed a synergic effect. Synergy was confirmed by three different methods. In addition, this is~~aciclovir observed *in vitro* on cell lines infected by EHV-1 was not validated *ex vivo* when using respiratory mucosa explants as a model (Glorieux et al., 2012) and was not transposable *in vivo* using valaciclovir, the prodrug of aciclovir (Garre et al., 2009). Although data observed in this study are promising, further investigations are necessary on *ex vivo* models to confirm the antiviral effect of our lead compounds before *in vivo* experiments implementation.

This report is also the first analysis of drug combinations against EHV-1. ~~Some studies have previously documented synergic effects between~~ Of all the combinations tested, only valganciclovir plus decitabine showed a synergic effect. It was previously demonstrated that gemcitabine in association with ganciclovir ~~and other compounds against HHV-5 (Chou et al., 2018; Drew, 2006). Combinations of valganciclovir with idoxuridine and pritelivir were additive. Combination of ganciclovir~~enhanced the antitumoral effects of HSV-TK suicide gene in a synergistic manner (Wang et al., 2016). This result is in agreement with ~~aphidicolin was never tested before. However, aciclovir acted synergistically with aphidicolin against HHV-1 when used at a 1:1 molar ratio (Michaelis et al., 2011). As decitabine acts in synergy with valganciclovir and was never tested against herpesviruses to~~our study.

Formatted: English (United Kingdom)

To our knowledge, ~~the mechanism~~decitabine has never before been reported to inhibit cellular infections by a herpesvirus. This led us to further investigate the mode of action of this

compound~~ was further investigated.~~. The pre-treatment of cells with decitabine did not provide antiviral effects against EHV-1, suggesting that decitabine incorporation in ~~cell~~cellular DNA did not mediate the antiviral effect ~~observed when~~of decitabine. This rather suggests that the antiviral effect of decitabine ~~was administrated to the cell culture after EHV-1 infection. As decitabine is known to induce cellular DNA hypomethylation~~ depend on its incorporation into viral DNA and/or some interference with the viral polymerase. Decitabine is well-known for preventing DNA methylation and this account for its antitumoral properties (Atallah et al., 2007; Schmelz et al., 2005~~),~~). We thus tested the antiviral effect of RG108, ~~another~~ which is a hypomethylation agent acting differently through DNA methyltransferase ~~inhibitor, was also tested against EHV-1.~~inhibition. The absence of RG108 activity against EHV-1 suggests that the ~~inhibitor~~inhibitory effect of decitabine against EHV-1 ~~was~~is not mediated by viral or cellular DNA hypomethylation.

~~Finally,~~ Finally, we showed that the addition of dC to culture medium inhibits decitabine antiviral activity. This strongly suggests that dC and decitabine ~~is a drug that requires to be phosphorylated to act as an anticancer agent alike gemcitabine. This~~channel through the same metabolic pathway, including phosphorylation~~ is~~ -dependent ~~on deoxycytidine kinase (DCK) (Stresemann and Lyko, 2008). The use of deoxycytidine (dC) inhibited~~activation and incorporation into cellular and viral DNA. Based on collected observations, we propose that decitabine ~~antiviral activity, probably by preventing decitabine phosphorylation by DCK through molecular competition. As the initial phosphorylation of decitabine by DCK is required for its activity, it is assumed that the decitabine active form in our model~~ is ~~the triphosphate form. Under this form, decitabine is probably~~ integrated ~~in~~into the EHV-1 DNA ~~during~~and/or jams the viral ~~replication~~polymerase, thus ~~inhibiting~~leading to the inhibition of viral growth. ~~As~~Interestingly, and as opposed to ganciclovir that needs activation by viral TK

Formatted: English (United Kingdom)

(Sullivan et al., 1992), decitabine phosphorylation only relies on cellular kinases to be activated and may represent a good candidate against viral strains resistant to ganciclovir.

In conclusion, the antiviral effect of ganciclovir–/valganciclovir and aphidicolin was confirmed against EHV-1. Most importantly, new EHV-1 inhibitors were identified, including idoxuridine, pritelivir and decitabine. The synergy observed between valganciclovir and decitabine is particularly interesting due to the complementarity of their mode of actions, and further investigations *ex vivo* and *in vivo* are warranted.

Conflict of interest

The authors declare no competing interests.

Acknowledgments

This work was supported by LABÉO, IFCE (Institut Français du Cheval et de l'Equitation, project AMIE), Fonds Eperon (project N87-2014, N07-2015, N07-2016, N13-2017 and N62-2017), Région Normandie (CPER R25 P3) and CENTAURE European project co-funded by Normandy County Council, European Union in the framework of the ERDF-ESF operationnal programme 2014-2020. We would like to thank Laurent Lemaitre from Boehringer Ingelheim, France who kindly provided us with EEK cells. We thank Christophe Denoyelle and Emilie Brotin from ImpedanCELL, Normandie Univ, UNICAEN, Caen, France for interacting and for their technical assistance with RTCA technology. We also thank all collaborators of the SAVE project.

References

Allen, G.P., 2002. Respiratory Infections by Equine Herpesvirus Types 1 and 4, 2002. Int. Vet. Inf. Serv.

Atallah, E., Kantarjian, H., Garcia-Manero, G., 2007. The role of decitabine in the treatment of myelodysplastic syndromes. Expert Opin. Pharmacother. 8, 65–73. https://doi.org/10.1517/14656566.8.1.65

Azab, W., Tsujimura, K., Kato, K., Arii, J., Morimoto, T., Kawaguchi, Y., Tohya, Y., Matsumura, T., Akashi, H., 2010. Characterization of a thymidine kinase-deficient mutant of equine herpesvirus 4 and in vitro susceptibility of the virus to antiviral agents. Antiviral Res. 85, 389–395. https://doi.org/10.1016/j.antiviral.2009.11.007

Berg, T., Nøttrup, T.J., Roed, H., 2019. Gemcitabine for recurrent ovarian cancer - a systematic review and meta-analysis. Gynecol. Oncol. 155, 530–537. https://doi.org/10.1016/j.ygyno.2019.09.026

Betz, U.A.K., Fischer, R., Kleymann, G., Hendrix, M., Rubsamen-Waigmann, H., 2002. Potent In Vivo Antiviral Activity of the Herpes Simplex Virus Primase-Helicase Inhibitor BAY 57-1293. Antimicrob. Agents Chemother. 46, 1766–1772. https://doi.org/10.1128/AAC.46.6.1766-1772.2002

Carmichael, R.J., Whitfield, C., Maxwell, L.K., 2013. Pharmacokinetics of ganciclovir and valganciclovir in the adult horse. J. Vet. Pharmacol. Ther. 36, 441–449. https://doi.org/10.1111/jvp.12029

~~Chou, S., Ercolani, R.J., Derakhchan, K., 2018. Antiviral activity of maribavir in combination with other drugs active against human cytomegalovirus. Antiviral Res. 157, 128–133. https://doi.org/10.1016/j.antiviral.2018.07.013~~

Chou, T.-C., Talalay, P., 1984. Quantitative analysis of dose-effect relationships: the combined effects of multiple drugs or enzyme inhibitors. Adv. Enzyme Regul. 22, 27–55. https://doi.org/10.1016/0065-2571(84)90007-4

De Clercq, E., 1984. The antiviral spectrum of (£)-5-(2-bromovinyl)-2 ’-deoxyuridine. J. Antimicrob. Chemother. 14, 85–95.

De Clercq, E., Li, G., 2016. Approved Antiviral Drugs over the Past 50 Years. Clin. Microbiol. Rev. 29, 695–747. https://doi.org/10.1128/CMR.00102-15

de la Fuente, R., Awan, A.R., Field, H.J., 1992. The acyclic nucleoside analogue penciclovir is a potent inhibitor of equine herpesvirus type 1 (EHV-1) in tissue culture and in a murine model. Antiviral Res. 18, 77–89. ~~https://doi.org/10.1016/0166-3542(92)90007-R~~

Denisova, O.V., Kakkola, L., Feng, L., Stenman, J., Nagaraj, A., Lampe, J., Yadav, B., Aittokallio, T., Kaukinen, P., Ahola, T., Kuivanen, S., Vapalahti, O., Kantele, A., Tynell, J., Julkunen, I., Kallio-Kokko, H., Paavilainen, H., Hukkanen, V., Elliott, R.M., De Brabander, J.K., Saelens, X., Kainov, D.E., 2012. Obatoclax, Saliphenylhalamide, and Gemcitabine Inhibit Influenza A Virus Infection. J. Biol. Chem. 287, 35324–35332.

~~Drew, W., 2006. Is combination antiviral therapy for CMV superior to monotherapy? J. Clin. Virol. 35, 485–488. https://doi.org/10.1016/j.jcv.2005.09.021~~

Drouot, E., Piret, J., Boivin, G., 2016. Artesunate demonstrates in vitro synergism with several antiviral agents against human cytomegalovirus. Antivir. Ther. 21, 535–539. https://doi.org/10.3851/IMP3028

Fang, Y., Ye, P., Wang, X., Xu, X., Reisen, W., 2011. Real-time monitoring of flavivirus induced cytopathogenesis using cell electric impedance technology. J. Virol. Methods 173, 251–258. https://doi.org/10.1016/j.jviromet.2011.02.013

Feng, J.Y., Ly, J.K., Myrick, F., Goodman, D., White, K.L., Svarovskaia, E.S., Borroto-Esoda, K., Miller, M.D., 2009. The triple combination of tenofovir, emtricitabine and

Formatted: French (France)

Formatted: Indent: Left: 0", First line: 0"

Formatted: French (France)

efavirenz shows synergistic anti-HIV-1 activity in vitro: a mechanism of action study. Retrovirology 6, 44. https://doi.org/10.1186/1742-4690-6-44

Ferreira, C.G.T., Campos, M.G., Felix, D.M., Santos, M.R., Carvalho, O.V. de, Diaz, M.A.N., Fietto, J.L.R., Bressan, G.C., Silva-Júnior, A., Almeida, M.R. de, 2018. Evaluation of the antiviral activities of Bacharis dracunculifolia and quercetin on Equid herpesvirus 1 in a murine model. Res. Vet. Sci. 120, 70–77. https://doi.org/10.1016/j.rvsc.2018.09.001

Friday, P.A., Scarratt, W.K., Elvinger, F., Timoney, P.J., Bonda, A., 2000. Ataxia and paresis with equine herpesvirus type 1 infection in a herd of riding school horses. J. Vet. Intern. Med. 14, 197–201.

Garre, B., Gryspeerdt, A., Croubels, S., De Backer, P., Nauwynck, H., 2009. Evaluation of orally administered valacyclovir in experimentally EHV1-infected ponies. Vet. Microbiol. 135, 214–221. https://doi.org/10.1016/j.vetmic.2008.09.062

Garre, B., Vandermeulen, K., Nugent, J., Neyts, J., Croubels, S., Debacker, P., Nauwynck, H., 2007. In vitro susceptibility of six isolates of equine herpesvirus 1 to acyclovir, ganciclovir, cidofovir, adefovir, PMEDAP and foscarnet. Vet. Microbiol. 122, 43–51. https://doi.org/10.1016/j.vetmic.2007.01.004

Gibson, J.S., 1992. The activity of (S)-l-[(3-hydroxy-2-phosphonyl methoxy) propyl] cytosine (HPMPC) against equine herpesvirus- 1 (EHV- 1) in cell cultures, mice and horses. Antiviral Res. 19, 219–232.

Glorieux, S., Vandekerckhove, A.P., Goris, N., Yang, X.-Y., Steukers, L., Van de Walle, G.R., Croubels, S., Neyts, J., Nauwynck, H.J., 2012. Evaluation of the antiviral activity of (1′S,2′R)-9-[[1′,2′-bis(hydroxymethyl)cycloprop-1′-yl]methyl]guanine (A-5021) against equine herpesvirus type 1 in cell monolayers and equine nasal mucosal explants. Antiviral Res. 93, 234–238. https://doi.org/10.1016/j.antiviral.2011.11.016

Goodman, L.B., Loregian, A., Perkins, G.A., Nugent, J., Buckles, E.L., Mercorelli, B., Kydd, J.H., Palù, G., Smith, K.C., Osterrieder, N., Davis-Poynter, N., 2007. A Point Mutation in a Herpesvirus Polymerase Determines Neuropathogenicity. PLoS Pathog. 3, e160. https://doi.org/10.1371/journal.ppat.0030160

Gravina, H.D., Tafuri, N.F., Silva Júnior, A., Fietto, J.L.R., Oliveira, T.T., Diaz, M.A.N., Almeida, M.R., 2011. In vitro assessment of the antiviral potential of trans-cinnamic acid, quercetin and morin against equid herpesvirus 1. Res. Vet. Sci. 91, e158–e162. https://doi.org/10.1016/j.rvsc.2010.11.010

Halbrook, C.J., Pontious, C., Kovalenko, I., Lapienyte, L., Dreyer, S., Lee, H.-J., Thurston, G., Zhang, Y., Lazarus, J., Sajjakulnukit, P., Hong, H.S., Kremer, D.M., Nelson, B.S., Kemp, S., Zhang, L., Chang, D., Biankin, A., Shi, J., Frankel, T.L., Crawford, H.C., Morton, J.P., Pasca di Magliano, M., Lyssiotis, C.A., 2019. Macrophage-Released Pyrimidines Inhibit Gemcitabine Therapy in Pancreatic Cancer. Cell Metab. 29, 1390-1399.e6. https://doi.org/10.1016/j.cmet.2019.02.001

He, P.-F., Zhou, J.-D., Yao, D.-M., Ma, J.-C., Wen, X.-M., Zhang, Z.-H., Lian, X.-Y., Xu, Z.-J., Qian, J., Lin, J., 2017. Efficacy and safety of decitabine in treatment of elderly patients with acute myeloid leukemia: A systematic review and meta-analysis. Oncotarget 8, 41498–41507. https://doi.org/10.18632/oncotarget.17241

Henninger, R.W., Reed, S.M., Saville, W.J., Allen, G.P., Hass, G.F., Kohn, C.W., Sofaly, C., 2007. Outbreak of Neurologic Disease Caused by Equine Herpesvirus-1 at a University Equestrian Center. J. Vet. Intern. Med. 21, 157–165.

James, S., Larson, K., Acosta, E., Prichard, M., 2015. Helicase-Primase as a Target of New Therapies for Herpes Simplex Virus Infections. Clin. Pharmacol. Ther. 97, 66–78. https://doi.org/10.1002/cpt.3

Formatted: French (France)

Formatted: French (France)

Jiang, Y.-C., Feng, H., Lin, Y.-C., Guo, X.-R., 2016. New strategies against drug resistance to herpes simplex virus. Int. J. Oral Sci. 8, 1–6.
Jourdan, J., Bureau, R., Rochais, C., Dallemagne, P., 2020. Drug repositioning: a brief overview. J. Pharm. Pharmacol. jphp.13273. https://doi.org/10.1111/jphp.13273
Kit, S., Ichimura, H., De Clercq, E., 1987. Phosphorylation of nucleoside analogs by equine herpesvirus type 1 pyrimidine deoxyribonucleoside kinase. Antiviral Res. 7, 53–67. https://doi.org/10.1016/0166-3542(87)90039-8
Kleymann, G., Fischer, R., Betz, U.A.K., Hendrix, M., Bender, W., Schneider, U., Handke, G., Eckenberg, P., Hewlett, G., Pevzner, V., Baumeister, J., Weber, O., Henninger, K., Keldenich, J., Jensen, A., Kolb, J., Bach, U., Popp, A., Mäben, J., Frappa, I., Haebich, D., Lockhoff, O., Rübsamen-Waigmann, H., 2002. New helicase-primase inhibitors as drug candidates for the treatment of herpes simplex disease. Nat. Med. 8, 392–398. https://doi.org/10.1038/nm0402-392
Léon, A., Fortier, G., Fortier, C., Freymuth, F., Tapprest, J., Leclercq, R., Pronost, S., 2008. Detection of equine herpesviruses in aborted foetuses by consensus PCR. Vet. Microbiol. 126, 20–29. https://doi.org/10.1016/j.vetmic.2007.06.019
Liu, Z., Liu, S., Xie, Z., Blum, W., Perrotti, D., Paschka, P., Klisovic, R., Byrd, J., Chan, K.K., Marcucci, G., 2007. Characterization of in vitro and in vivo hypomethylating effects of decitabine in acute myeloid leukemia by a rapid, specific and sensitive LC-MS/MS method. Nucleic Acids Res. 35, e31–e31. https://doi.org/10.1093/nar/gkl1156
Lunn, D.P., Davis-Poynter, N., Flaminio, M.J.B.F., Horohov, D.W., Osterrieder, K., Pusterla, N., Townsend, H.G.G., 2009. Equine Herpesvirus-1 Consensus Statement. J. Vet. Intern. Med. 23, 450–461. https://doi.org/10.1111/j.1939-1676.2009.0304.x
Maggs, D.J., Clarke, H.E., 2004. In vitro efficacy of ganciclovir, cidofovir, penciclovir, foscarnet, idoxuridine, and acyclovir against feline herpesvirus type-1. Am. J. Vet. Res. 65, 399–403. https://doi.org/10.2460/ajvr.2004.65.399
Maxwell, L.K., 2017. Antiherpetic Drugs in Equine Medicine. Vet. Clin. North Am. Equine Pract. 33, 99–125. https://doi.org/10.1016/j.cveq.2016.12.002
Maxwell, L.K., Bentz, B.G., Bourne, D.W.A., Erkert, R.S., 2008. Pharmacokinetics of valacyclovir in the adult horse. J. Vet. Pharmacol. Ther. 31, 312–320. https://doi.org/10.1111/j.1365-2885.2008.00957.x
Momparler, R.L., 2005. Pharmacology of 5-Aza-2′-deoxycytidine (decitabine). Seminars in Hematology 42, S9–S16. https://doi.org/10.1053/j.seminhematol.2005.05.002
~~Michaelis, M., Langer, K., Vogel, J.-U., Kreuter, J., Rabenau, H., Doerr, H.-W., Cinatl, J., 2011. In vitro Antiviral Activity of Aphidicolin and its Derivates. Arzneimittelforschung 52, 393–399. https://doi.org/10.1055/s-0031-1299904~~
Murray, M.J., Piero, F., Jeffrey, S.C., Davis, M.S., Furr, M.O., Dubovi, E.J., Mayo, J.A., 1998. Neonatal Equine Herpesvirus Type 1 Infection on a Thoroughbred Breeding Farm. J. Vet. Intern. Med. 12, 36–41. https://doi.org/10.1111/j.1939-1676.1998.tb00494.x
Nugent, J., Birch-Machin, I., Smith, K.C., Mumford, J.A., Swann, Z., Newton, J.R., Bowden, R.J., Allen, G.P., Davis-Poynter, N., 2006. Analysis of Equid Herpesvirus 1 Strain Variation Reveals a Point Mutation of the DNA Polymerase Strongly Associated with Neuropathogenic versus Nonneuropathogenic Disease Outbreaks. J. Virol. 80, 4047–4060. https://doi.org/10.1128/JVI.80.8.4047-4060.2006
Paillot, R., Sutton, G., Thieulent, C., Marcillaud-Pitel, C., Pronost, S., 2020. New EHV-1 variant identified. Vet. Rec. 186, 573.
Pan, T., Huang, B., Zhang, W., Gabos, S., Huang, D.Y., Devendran, V., 2013. Cytotoxicity assessment based on the AUC50 using multi-concentration time-dependent cellular

Formatted: French (France)

Formatted: French (France)

Field Code Changed

response curves. Anal. Chim. Acta 764, 44–52. https://doi.org/10.1016/j.aca.2012.12.047

Piret, J., Goyette, N., Boivin, G., 2016. Novel Method Based on Real-Time Cell Analysis for Drug Susceptibility Testing of Herpes Simplex Virus and Human Cytomegalovirus. J. Clin. Microbiol. 54, 2120–2127. https://doi.org/10.1128/JCM.03274-15

Price, N.B., Prichard, M.N., 2011. Progress in the development of new therapies for herpesvirus infections. Curr. Opin. Virol. 1, 548–554. https://doi.org/10.1016/j.coviro.2011.10.015

Prichard, M.N., Shipman, C., 1990. A three-dimensional model to analyze drug-drug interactions. Antiviral Res. 14, 181–206.

Pronost, S., Léon, A., Legrand, L., Fortier, C., Miszczak, F., Freymuth, F., Fortier, G., 2010. Neuropathogenic and non-neuropathogenic variants of equine herpesvirus 1 in France. Vet. Microbiol. 145, 329–333. https://doi.org/10.1016/j.vetmic.2010.03.031

Rollinson, E.A., 1987. Comparative efficacy of three 2′-fluoropyrimidine nucleosides and 9-(1,3-dihydroxy-2-propoxymethyl)guanine (BW B759U) against pseudorabies and equine rhinopneumonitis virus infection in vitro and in laboratory animals. Antiviral Res. 7, 25–33. https://doi.org/10.1016/0166-3542(87)90036-2

Schmelz, K., Sattler, N., Wagner, M., Lübbert, M., Dörken, B., Tamm, I., 2005. Induction of gene expression by 5-Aza-2′-deoxycytidine in acute myeloid leukemia (AML) and myelodysplastic syndrome (MDS) but not epithelial cells by DNA-methylation-dependent and -independent mechanisms. Leukemia 19, 103–111. https://doi.org/10.1038/sj.leu.2403552

~~Smith~~Shin, H., Kim, C., Cho, S., 2018. Gemcitabine and Nucleos(t)ide Synthesis Inhibitors Are Broad-Spectrum Antiviral Drugs that Activate Innate Immunity. Viruses 10, 1-11. https://doi.org/10.3390/v10040211

Smith, K.L., Allen, G.P., Branscum, A.J., Frank Cook, R., Vickers, M.L., Timoney, P.J., Balasuriya, U.B.R., 2010. The increased prevalence of neuropathogenic strains of EHV-1 in equine abortions. Vet. Microbiol. 141, 5–11. https://doi.org/10.1016/j.vetmic.2009.07.030

Stresemann, C., Lyko, F., 2008. Modes of action of the DNA methyltransferase inhibitors azacytidine and decitabine. Int. J. Cancer 123, 8–13. https://doi.org/10.1002/ijc.23607

Sullivan, V., Talarico, C.L., Stanat, S.C., Davis, M., Coen, D.M., Biron, K.K., 1992. A protein kinase homologue controls phosphorylation of ganciclovir in human cytomegalovirus-infected cells. Nature 358, 162–164.

Sutton, Garvey, Cullinane, Jourdan, Fortier, Moreau, Foursin, Gryspeerdt, Maisonnier, Marcillaud-Pitel, Legrand, Paillot, Pronost, 2019. Molecular Surveillance of EHV-1 Strains Circulating in France during and after the Major 2009 Outbreak in Normandy Involving Respiratory Infection, Neurological Disorder, and Abortion. Viruses 11, 916. https://doi.org/10.3390/v11100916

Tallmadge, R.L., Žygelytė, E., Van de Walle, G.R., Kristie, T.M., Felippe, M.J.B., 2018. Effect of a Histone Demethylase Inhibitor on Equine Herpesvirus-1 Activity In Vitro. Front. Vet. Sci. 5. https://doi.org/10.3389/fvets.2018.00034

Thieulent, C.J., Hue, E.S., Fortier, C.I., Dallemagne, P., Zientara, S., Munier-Lehmann, H., Hans, A., Fortier, G.D., Pitel, P.-H., Vidalain, P.-O., Pronost, S.L., 2019. Screening and evaluation of antiviral compounds against Equid alpha-herpesviruses using an impedance-based cellular assay. Virology 526, 105–116. https://doi.org/10.1016/j.virol.2018.10.013

Vissani, M.A., Thiry, E., Dal Pozzo, F., Barrandeguy, M., 2016. Antiviral agents against equid alphaherpesviruses: Current status and perspectives. Vet. J. 207, 38–44. https://doi.org/10.1016/j.tvjl.2015.06.010

Field Code Changed

Formatted: No underline

Formatted: French (France)

Wang, J., Li, A., Jin, M., Zhang, F., Li, X., 2016. Dual-modality imaging demonstrates the enhanced antitumoral effect of herpes simplex virus-thymidine kinase/ganciclovir plus gemcitabine combination therapy on cholangiocarcinoma. Experimental and Therapeutic Medicine 12, 183–189. https://doi.org/10.3892/etm.2016.3294

Williams, S.L., Hartline, C.B., Kushner, N.L., Harden, E.A., Bidanset, D.J., Drach, J.C., Townsend, L.B., Underwood, M.R., Biron, K.K., Kern, E.R., 2003. In Vitro Activities of Benzimidazole D- and L-Ribonucleosides against Herpesviruses. Antimicrob. Agents Chemother. 47, 2186–2192. https://doi.org/10.1128/AAC.47.7.2186-2192.2003

Zhang, J.-H., Chung, T.D.Y., Oldenburg Kevin R., 1999. A Simple Statistical Parameter for Use in Evaluation and Validation of High Throughput Screening Assays. J. Biomol. Screen. 4, 67–73.

Table 1: List of compounds presenting an antiviral effect against EHV-1 Kyd strain on E. Derm cell.

Data presented in this table are the mean (S.D.) of three independent experiments. ~~IC_{50}: IC_{50}[a]~~EC_{50}: EC_{50}[a] half maximal effective concentration measured by impedance using Real-Time Cell Analysis (RTCA) system or ~~IC_{50}[d]~~EC_{50}[d] qPCR assay.

CC_{50}: CC_{50}[b] half maximal cytotoxic concentration measured by impedance using RTCA system or CC_{50}[e] CellTiter-Glo (CTG) method. "CC_{50} > 50" means that the compound did not show toxicity at the highest concentration tested (50 µM).

SI: Selectivity Index is the ration of CC_{50} obtained by RTCA to ~~IC_{50}~~EC_{50} obtained by RTCA[c] or CC_{50} obtained by CTG to ~~IC_{50}~~EC_{50} obtained by qPCR[f]. If "CC_{50} > 50", an arbitrary value of SI is calculated with 50 µM, but it is probably underestimated.

Bold compounds are the selected compounds in part 3.1 of results.

Table 2: Combination analysis of compounds against EHV-1 KyD strain on E. Derm cells.

[a]Mean volumes of synergy or antagonism are presented based on 95% confidence levels using MacSynergy II method.

Values determined using MacSynergy II software (Prichard and Shipman, 1990) via area under normalised curves (AUC_n) data from 0 to 96 hours post-infection using impedance measurement. Results are obtained from three independently experiments.

~~**Table**~~

Figure 1: Correlation plot between EC_{50} values obtained by qPCR and RTCA from compounds presenting an antiviral effect against EHV-1 Kyd strain (n=16). Compounds with

Formatted: Font color: Auto

an EC_{50} well defined by qPCR or RTCA were included in this calculation (we excluded data with EC_{50} >50µM). Spearman correlation coefficient (R), P-value and confidence interval (Conf.Int.) are presented.

Figure 2: Antiviral effect of selected compounds against EHV-1 KyD strain on E. Derm cells. (**A**) Half maximal effective concentration (EC_{50}) measured at different times post-infection using impedance measurement (48 to 120 hpi). The dotted line represents the cut off EC_{50} value. (**B**) Dose-response curves obtained by qPCR (plain line) and RTCA (dotted line) for the eight retained compounds against EHV-1 at 48 hpi. Results are from three independent experiments.

Figure 3: Antiviral effect of the eight selected compounds on different cell lines and against different EHV-1 strains. (**A**) Susceptibility of EHV-1 KyD strain to the 8 selected antiviral compounds on three different cell lines: E Derm, EEK and RK13.

~~[a]Significant difference between E. Derm and EEK cells ([a]p~~Results are from three independent experiments ($^{*}p < 0.05$, $^{**}p < 0.01$). ~~).~~

~~[b]Significant difference between E. Derm and RK13 cells ([b]p, **p~~

~~[c]Significant difference between E. Derm and EEK cells ([c]p < 0.05).~~

~~[d]Significant difference between E. Derm and RK13 cells ([d]p < 0.01).~~

~~[e]Significant difference between EEK and E. Derm cells ([e]p < 0.01).~~

~~[f]Significant difference between EEK and RK13 cells ([f]p < 0.05).~~

~~[g]Significant difference between RK13 and E. Derm cells ([g]p < 0.01).~~

~~[h]Significant difference between RK13 and EEK cells ([h]p < 0.01).~~

Formatted: Left

~~**Table 4:**~~ antiviral compounds~~: EC50 value were obtained by qPCR on EEK cells.~~

Formatted: Left, Space After: 0 pt, Don't adjust space between Latin and Asian text, Don't adjust space between Asian text and numbers

~~[#]Mutation of amino-acid at position 2254 of the ORF30 (Nugent et al., 2006).~~**B**) Susceptibility of three French isolates of EHV-1 to the 8 selected antiviral compounds. G_{2254} indicates a guanine in ORF30 position 2254 (Aspartic Acid (D_{752}) in position 752 of the protein), A_{2254} indicates an adenine (Asparagine (N_{752})) and C_{2254} indicates a cytidine (Histidine (H_{752})).

~~[a]Significant difference between strains FR-38991 and FR-56628 ([a]p < 0.05).~~

~~[b]Significant difference between strains FR-38991 and FR-6815 ([b]p < 0.01).~~

~~[c]Significant difference between strains FR-38991 and FR-56628 ([c]p~~ EC_{50} values were obtained by qPCR on EEK cells. Results are from three independent experiments ($^{*}p < 0.05$, $^{**}p <$

Formatted: Font: 12 pt

0.01).[d]Significant difference between strains FR-38991 and FR-6815 ([d]$p < 0.01$).
[e]Significant difference between strains FR-38991 and FR-56628 ([e]$p < 0.01$).

Figure 1: Antiviral effect of selected compounds against EHV-1 KyD strain on E. Derm cells at different times post-infection using impedance measurement. Results are obtained from three independent experiments. IC_{50}: half maximal effective concentration. Dotted line represent the cut off IC_{50} value.

Figure 2

Figure 4: Synergistic inhibition of EHV-1 KyD strain replication in E. Derm cells by combination of valganciclovir/decitabine. (**A**) Analysis of interaction of VGCV and DTB using impedance measurement with MacSynergy II software. Peaks of statistically significant (95% confidence level) synergy are shown above the plane in colours from grey to blue, with dark blue indicating a strong synergy. The volume of synergy for this interaction is 63.24, which is interpreted as moderate synergy. Results are obtained from three independently experiments performed using impedance measurement. (**B**) Isobologram analysis of the interaction of VGCV and DTB using impedance measurement. The diagonally dotted line in red represents additivity. Values below and above this line are interpreted as synergy or antagonist, respectively. The ADA value for this interaction is -0.30 ($p < 0.001$) which is interpreted as synergy. Results are obtained from three independently experiments performed using impedance measurement. (**C**) Median-effect analysis table representing the interaction of VGCV and DTB at 1:1 ratio using qPCR assay. Combination Index (CI) was calculated using the Chou and Talalay equation (Chou & Talalay, 1984). CI < 1, CI = 1 and CI > 1 indicate synergism, additive and antagonism, respectively. The weighted CI is calculated as follows: $CI_{wt} = (CI_{50} + 2CI_{75} + 3\ CI_{90} + 4CI_{95})/10$. The CI_{wt} value for this interaction is 0.20 which is interpreted as synergy. Results are obtained from three independently experiments performed using qPCR assay.

Figure ~~3~~5: ~~Decitabine (5 µM) pre-infection or post-infection treatment after infection by EHV-1 KyD strain of E. Derm cells measured at 48 hpi by (**A**) impedance measurement and (**B**) viral genome copies number quantitation. Results are obtained from three independents experiments ($^{***}p < 0.001$).~~Effect of decitabine (5µM) pre-infection or post-infection treatment on E. Derm cells infected with EHV-1 KyD strain measured at 48 hpi by (**A**) impedance measurement and (**B**) viral genome copies number quantitation. Results are from three independent experiments ($^{***}p < 0.001$).

Figure ~~4~~6: Effect of the DNA methyltransferase inhibitor, RG108 and decitabine on E. Derm cells infected ~~by~~ with EHV-1 KyD strain measured at 48 hpi by (**A**) impedance measurement and (**B**) viral genome copies number quantitation~~load quantitation~~. Results are ~~obtained~~ from three ~~independents~~independent experiments.

Figure ~~5~~7: Effect of deoxycytidine (dC) on the antiviral effect of decitabine (DTB) against EHV-1. (**A**) Viral genome copies number produces in the cell culture supernatant at 48 hpi in absence (0 µM) or presence (1.6 and 6.4 µM) of DTB with 100 µM of cytidine, uridine, adenosine, guanosine and dC. Results are obtained from five ~~independents~~independent experiments ($^{*}p < 0.05$). (**B**) Microscopic observation at 48 hpi of E. Derm cells infected or not by EHV-1 KyD strain in presence of DTB (6.4 µM) with or without dC (100 µM) treatment. (**C**) Impedance measurement at 48 hpi of E. Derm cells infected with EHV-1 KyD strain and treated with increased concentrations of DTB in the presence of the indicated concentration of dC. Results are ~~obtained~~ from three ~~independents~~independent experiments.

Supplementary Table 1: Library of selected compounds for their antiviral effects on different famil~~y~~ies of viruses.

~~**Supplementary Figure 1**~~**Supplementary Figure 1:** Screening of 2,891 chemical compounds in order to identify EHV-1 inhibitors. CERMN library was screened at 10 µM, Prestwick® Chemical library was screened at 10 µg/mL and in-house antiviral library were screened at 4

concentrations (0.4, 2, 10 and 50 µM). For each library, the percentage of area under normalised Cell Index (CI) from 0 to 96 hpi of treated cells compared to mock-treated cells (%AUC) and the increase of the time required for the CI to decrease by 50% after virus infection (CIT_{50}) of treated cells compared to mock-treated cells (ΔCIT_{50}) were reported graphically. Compounds allowing an increase of the AUCn by 25% (red line) and a delay of the CIT_{50} by >8 h (red line) as compared to non-treated cells were identified as potential antiviral compounds against EHV-1.

Supplementary Figure 2: Cytotoxicity assay of combinations on equine dermal cells. Toxicity evaluation is measured by luminescence assays using CellTiter Glo® kit. Histogram represents the percentage viability of E. Derm cells treated with the highest concentration of compounds compared to mock-treated cells. Each data corresponds to mean ± SD of three independent experiments.

Formatted: Superscript

CompuSyn Report

Experiment Name: GCV-DTB-qPCR01

Date:

File Name: \\Lfdsrv003\groupe\PR\PR_Theses\CT_SAVE\Résultats ANVIE-SAVE\07_Combinaisons-CCL57\VGCV_DTB_COMBINAISON\CompuSyn qPCR\VGCV-DTB_01.cse

Description

Drug: Valganciclovir (VGCV) [μM]

Drug: Decitabine (DTB) [μM]

Drug Combo: VGCV + DTB (1:1) (COMBI1) (VGCV+DTB [1:1])

Data for Drug: VGCV [μM]

Dose	Effect
5.0	0.6
2.5	0.45
1.25	0.28
0.63	0.17
0.31	0.02
0.16	0.01
0.08	0.01

7 data points entered.

X-int: 0.48835
Y-int: -0.6770 +/- 0.09529
m: 1.38630 +/- 0.15089
Dm: 3.07861
r: 0.97164

Data for Drug: DTB [μM]

Dose	Effect
10.0	0.64
5.0	0.54
2.5	0.34
1.25	0.18
0.63	0.05
0.31	0.01
0.16	0.01
0.08	0.01
0.04	0.01

9 data points entered.

X-int: 0.78939
Y-int: -0.8760 +/- 0.10535
m: 1.10972 +/- 0.13185
Dm: 6.15732
r: 0.95398

Data for Drug Combo: COMBI1 (VGCV+DTB [1:1])

Dose A	Effect
5.0+	0.99
2.5+	0.82
1.25+	0.38
0.63+	0.25
0.31+	0.14
0.16+	0.04
0.08+	0.01

7 data points entered.

X-int: 0.25821
Y-int: -0.5124 +/- 0.13586
m: 1.98439 +/- 0.22385
Dm: 1.81222
r: 0.96963

Dose-Effect Curve

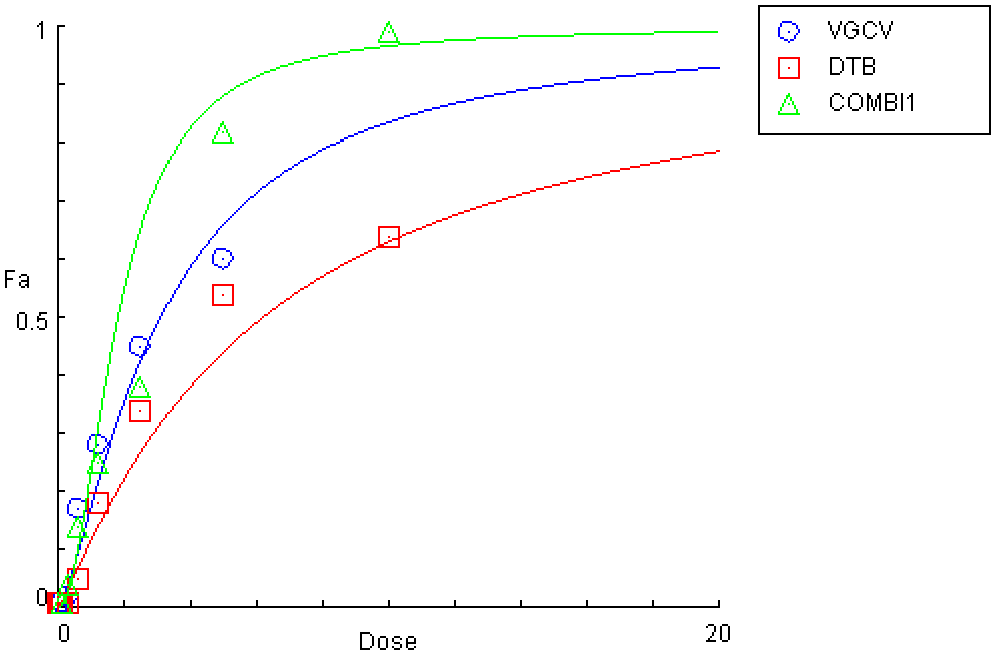

Median-Effect Plot

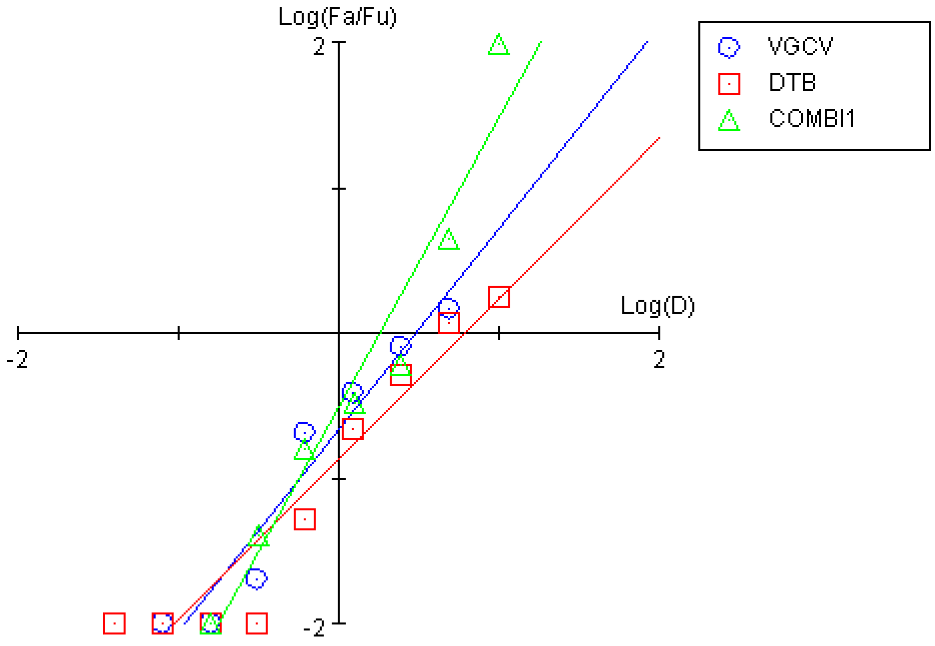

CI Data for Drug Combo: COMBI1 (VGCV+DTB [1:1])

Fa	CI Value	Total Dose
0.05	1.03219	0.41096
0.1	0.82678	0.59888
0.15	0.72224	0.75611
0.2	0.65308	0.90118
0.25	0.60140	1.04178
0.3	0.55990	1.18243
0.35	0.52491	1.32657
0.4	0.49432	1.47732
0.45	0.46682	1.63792
0.5	0.44148	1.81222
0.55	0.41765	2.00507
0.6	0.39476	2.22305
0.65	0.37234	2.47566
0.7	0.34991	2.77746
0.75	0.32691	3.15246
0.8	0.30259	3.64426
0.85	0.27575	4.34349
0.9	0.24403	5.48386
0.95	0.20087	7.99132
0.97	0.17523	10.4466

CI values for actual experimental points:

Total Dose	Fa	CI Value
10.0	0.99	0.07195
5.0	0.82	0.37553
2.5	0.38	0.89357
1.26	0.25	0.72737
0.62	0.14	0.63145

Total Dose	Fa	CI Value
0.32	0.04	0.96998
0.16	0.01	1.53158

Combination Index Plot

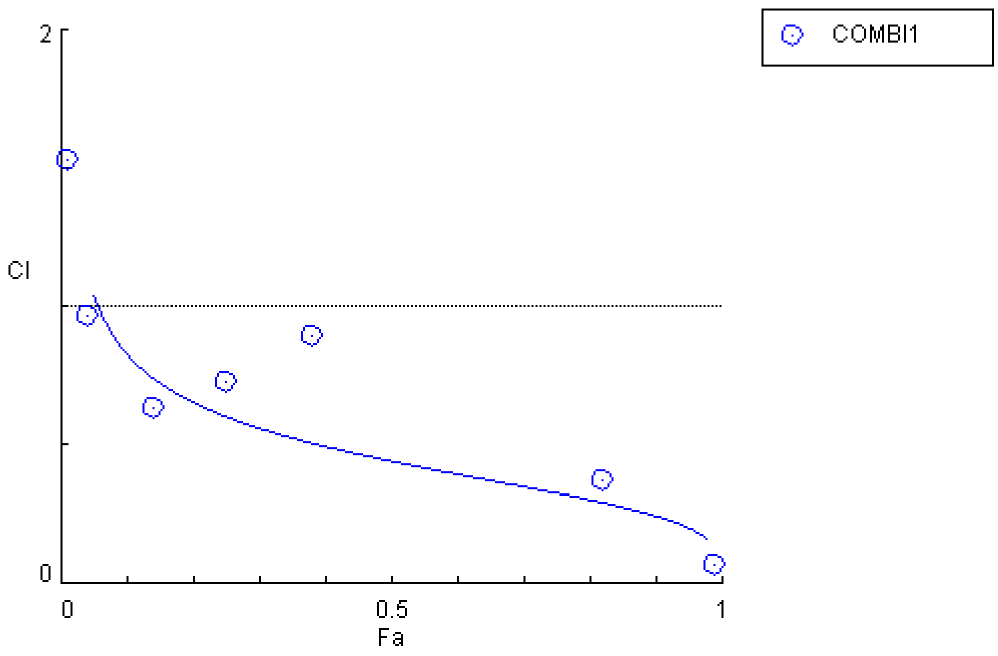

DRI Data for Drug Combo: COMBI1 (VGCV+DTB [1:1])

Fa	Dose VGCV	Dose DTB	DRI VGCV	DRI DTB
0.05	0.36807	0.43358	1.79126	2.11005
0.1	0.63098	0.85015	2.10723	2.83915
0.15	0.88094	1.28987	2.33019	3.41185
0.2	1.13256	1.76545	2.51350	3.91808
0.25	1.39375	2.28793	2.67573	4.39237
0.3	1.67077	2.86943	2.82599	4.85345
0.35	1.96981	3.52474	2.96977	5.31405
0.4	2.29791	4.27278	3.11092	5.78452
0.45	2.66373	5.13875	3.25256	6.27472
0.5	3.07861	6.15732	3.39761	6.79532
0.55	3.55811	7.37777	3.54912	7.35912
0.6	4.12455	8.87305	3.71071	7.98276
0.65	4.81155	10.7561	3.88707	8.68949
0.7	5.67274	13.2126	4.08484	9.51413
0.75	6.80022	16.5707	4.31424	10.5129
0.8	8.36849	21.4747	4.59269	11.7855
0.85	10.7588	29.3926	4.95398	13.5341
0.9	15.0208	44.5953	5.47817	16.2642
0.95	25.7499	87.4411	6.44447	21.8840
0.97	37.7861	141.184	7.23414	27.0295

DRI values calculated at experimental points

Fa	Dose VGCV	Dose DTB	DRI VGCV	DRI DTB
0.99	84.7021	387.008	16.9404	77.4016

Fa	Dose VGCV	Dose DTB	DRI VGCV	DRI DTB
0.82	9.19157	24.1448	3.67663	9.65791
0.38	2.16267	3.96099	1.73014	3.16879
0.25	1.39375	2.28793	2.21231	3.63164
0.14	0.83113	1.19941	2.68106	3.86905
0.04	0.31099	0.35127	1.94369	2.19543
0.01	0.11190	0.09796	1.39870	1.22454

DRI Plot for Combo: COMBI1 (VGCV+DTB [1:1])

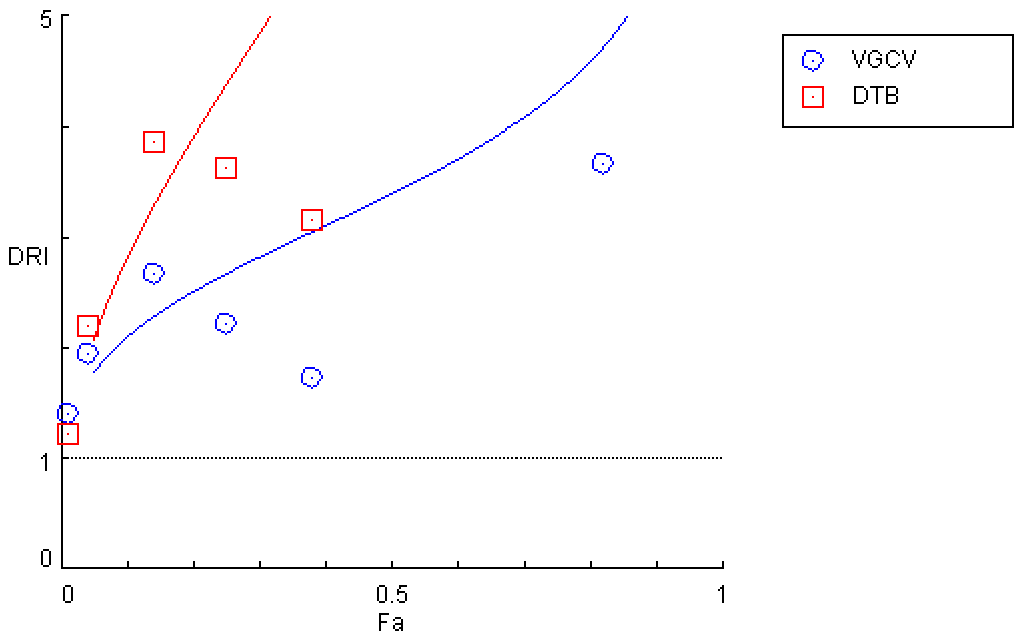

Summary Table

Experiment Name: GCV-DTB-qPCR01

Date:

File Name: \\Lfdsrv003\groupe\PR\PR_Theses\CT_SAVE\Résultats ANVIE-SAVE\07_Combinaisons-CCL57\VGCV_DTB_COMBINAISON\CompuSyn qPCR\VGCV-DTB_01.cse

Description

Drug: Valganciclovir (VGCV) [µM]
Drug: Decitabine (DTB) [µM]
Drug Combo: VGCV + DTB (1:1) (COMBI1) (VGCV+DTB [1:1])

Drug/Combo	Dm	m	r
VGCV	3.07861	1.38630	0.97164
DTB	6.15732	1.10972	0.95398
COMBI1	1.81222	1.98439	0.96963

CI values at:

Combo	ED50	ED75	ED90	ED95
COMBI1	0.44148	0.32691	0.24403	0.20087

Data for Fa = 0.5

Drug/Combo	CI value	Dose VGCV	Dose DTB
VGCV		3.07861	
DTB			6.15732
COMBI1	0.44148	0.90611	0.90611

Data for Fa = 0.75

Drug/Combo	CI value	Dose VGCV	Dose DTB
VGCV		6.80022	
DTB			16.5707
COMBI1	0.32691	1.57623	1.57623

Data for Fa = 0.9

Drug/Combo	CI value	Dose VGCV	Dose DTB
VGCV		15.0208	
DTB			44.5953
COMBI1	0.24403	2.74193	2.74193

Data for Fa = 0.95

Drug/Combo	CI value	Dose VGCV	Dose DTB
VGCV		25.7499	
DTB			87.4411
COMBI1	0.20087	3.99566	3.99566

Data for Fa = 0.97

Drug/Combo	CI value	Dose VGCV	Dose DTB
VGCV		37.7861	
DTB			141.184
COMBI1	0.17523	5.22331	5.22331

CompuSyn Report

**Experiment Name:**	VGCV-DTB-02
**Date:**	191003
**File Name:**	\\Lfdsrv003\groupe\PR\PR_Theses\CT_SAVE\Résultats ANVIE-SAVE\07_Combinaisons-CCL57\VGCV_DTB_COMBINAISON\CompuSyn qPCR\VGCV-DTB_02.cse
**Description**	

**Drug:**	Valganciclovir (VGCV) [µM]
**Drug:**	Decitabine (DTB) [µM]
**Drug Combo:**	VGCV-DTB (1:1) (COMBI2) (VGCV+DTB [1:1])

Data for Drug: VGCV [µM]

Dose	Effect
5.0	0.64
2.5	0.33
1.25	0.21
0.63	0.16
0.31	0.18
0.16	0.13
0.08	0.05

7 data points entered.

**X-int:**	0.66265
**Y-int:**	-0.4511 +/- 0.07449
**m:**	0.68071 +/- 0.11794
**Dm:**	4.59890
**r:**	0.93246

Data for Drug: DTB [µM]

Dose	Effect
10.0	0.8
5.0	0.66
2.5	0.62
1.25	0.36
0.63	0.34
0.31	0.2
0.16	0.04
0.08	0.01
0.04	0.01

9 data points entered.

**X-int:**	0.31951
**Y-int:**	-0.3691 +/- 0.08562
**m:**	1.15528 +/- 0.10716
**Dm:**	2.08692
**r:**	0.97118

Data for Drug Combo: COMBI2 (VGCV+DTB [1:1])

Dose A	Effect
5.0+	0.99
2.5+	0.77
1.25+	0.86
0.63+	0.55
0.31+	0.41
0.16+	0.2

6 data points entered.

X-int: -0.0437

Y-int: 0.06557 +/- 0.17258

m: 1.50009 +/- 0.30312

Dm: 0.90425

r: 0.92715

Dose-Effect Curve

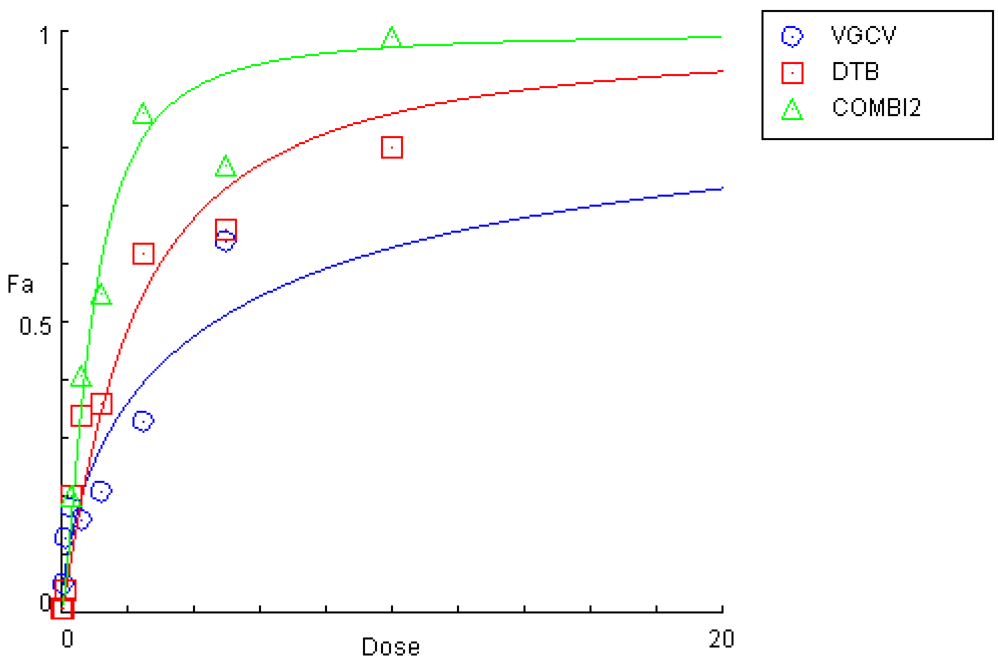

Median-Effect Plot

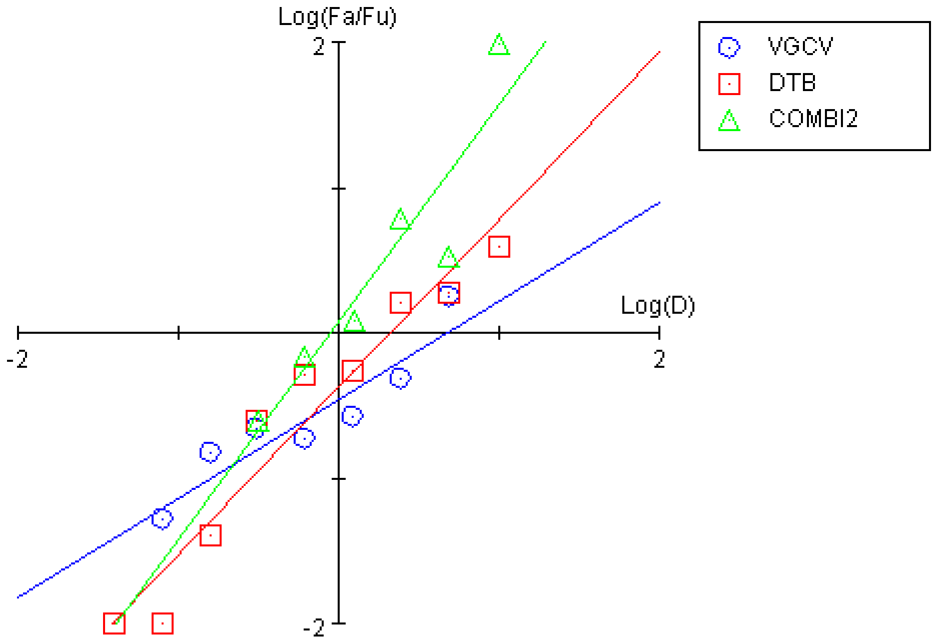

CI Data for Drug Combo: COMBI2 (VGCV+DTB [1:1])

Fa	CI Value	Total Dose
0.05	1.43327	0.12701
0.1	0.90867	0.20901
0.15	0.70140	0.28451
0.2	0.58449	0.35887
0.25	0.50697	0.43474
0.3	0.45047	0.51403
0.35	0.40661	0.59851
0.4	0.37096	0.69008
0.45	0.34096	0.79103
0.5	0.31496	0.90425
0.55	0.29186	1.03368
0.6	0.27086	1.18489
0.65	0.25136	1.36618
0.7	0.23285	1.59071
0.75	0.21482	1.88084
0.8	0.19674	2.27844
0.85	0.17785	2.87394
0.9	0.15678	3.91212
0.95	0.12985	6.43783
0.97	0.11453	9.17616

CI values for actual experimental points:

Total Dose	Fa	CI Value
10.0	0.99	0.04615
5.0	0.77	0.51305
2.5	0.86	0.14333
1.26	0.55	0.35576
0.62	0.41	0.31861

Total Dose	Fa	CI Value
0.32	0.2	0.52118

Combination Index Plot

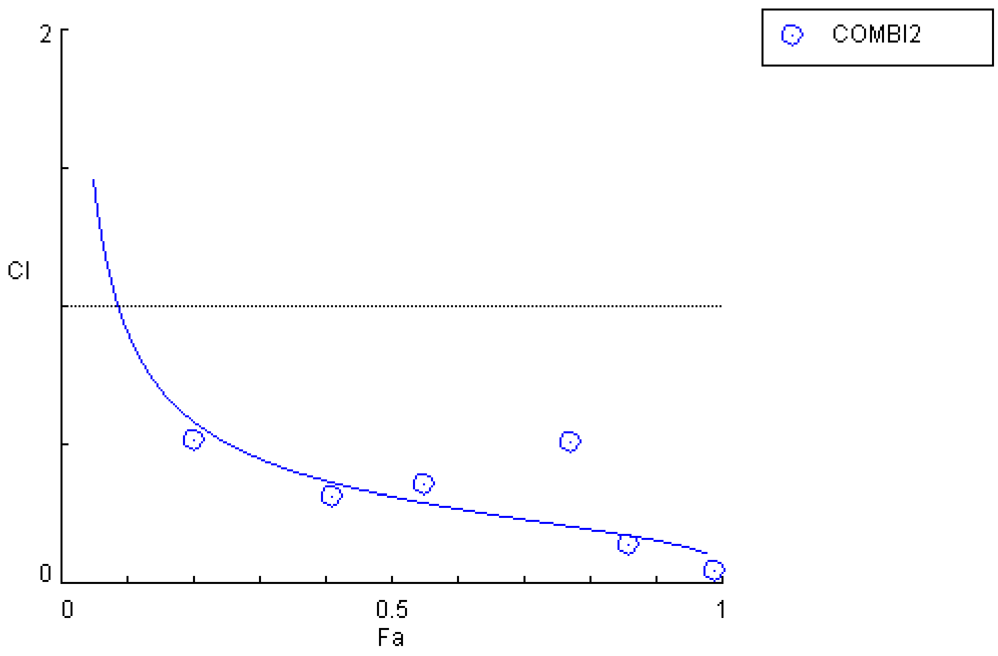

DRI Data for Drug Combo: COMBI2 (VGCV+DTB [1:1])

Fa	Dose VGCV	Dose DTB	DRI VGCV	DRI DTB
0.05	0.06083	0.16317	0.95780	2.56933
0.1	0.18231	0.31155	1.74451	2.98116
0.15	0.35972	0.46498	2.52869	3.26859
0.2	0.60005	0.62859	3.34409	3.50314
0.25	0.91566	0.80633	4.21244	3.70951
0.3	1.32456	1.00228	5.15366	3.89971
0.35	1.85226	1.22123	6.18960	4.08090
0.4	2.53492	1.46921	7.34670	4.25805
0.45	3.42472	1.75416	8.65888	4.43514
0.5	4.59890	2.08692	10.1717	4.61580
0.55	6.17566	2.48281	11.9489	4.80382
0.6	8.34340	2.96435	14.0831	5.00361
0.65	11.4184	3.56629	16.7158	5.22081
0.7	15.9674	4.34534	20.0758	5.46339
0.75	23.0981	5.40130	24.5615	5.74352
0.8	35.2467	6.92858	30.9393	6.08186
0.85	58.7950	9.36657	40.9160	6.51829
0.9	116.010	13.9795	59.3083	7.14675
0.95	347.716	26.6922	108.023	8.29230
0.97	759.324	42.2909	165.499	9.21755

DRI values calculated at experimental points

Fa	Dose VGCV	Dose DTB	DRI VGCV	DRI DTB
0.99	3929.79	111.407	785.958	22.2813
0.77	27.1371	5.93932	10.8549	2.37573

Fa	Dose VGCV	Dose DTB	DRI VGCV	DRI DTB
0.86	66.1943	10.0441	52.9554	8.03532
0.55	6.17566	2.48281	9.80263	3.94096
0.41	2.69427	1.52294	8.69121	4.91273
0.2	0.60005	0.62859	3.75033	3.92870

DRI Plot for Combo: COMBI2 (VGCV+DTB [1:1])

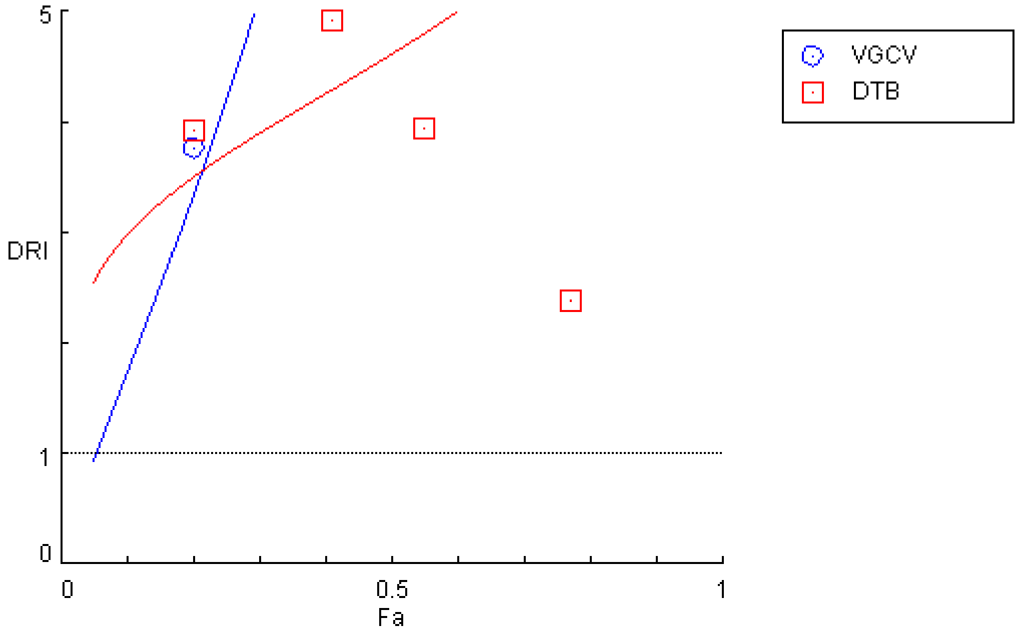

Summary Table

Experiment Name: VGCV-DTB-02

Date: 191003

File Name: \\Lfdsrv003\groupe\PR\PR_Theses\CT_SAVE\Résultats ANVIE-SAVE\07_Combinaisons-CCL57\VGCV_DTB_COMBINAISON\CompuSyn qPCR\VGCV-DTB_02.cse

Description

Drug: Valganciclovir (VGCV) [µM]
Drug: Decitabine (DTB) [µM]
Drug Combo: VGCV-DTB (1:1) (COMBI2) (VGCV+DTB [1:1])

Drug/Combo	Dm	m	r
VGCV	4.59890	0.68071	0.93246
DTB	2.08692	1.15528	0.97118
COMBI2	0.90425	1.50009	0.92715

CI values at:

Combo	ED50	ED75	ED90	ED95
COMBI2	0.31496	0.21482	0.15678	0.12985

Data for Fa = 0.5

Drug/Combo	CI value	Dose VGCV	Dose DTB

Drug/Combo	CI value	Dose VGCV	Dose DTB
VGCV		4.59890	
DTB			2.08692
COMBI2	0.31496	0.45213	0.45213

Data for Fa = 0.75

Drug/Combo	CI value	Dose VGCV	Dose DTB
VGCV		23.0981	
DTB			5.40130
COMBI2	0.21482	0.94042	0.94042

Data for Fa = 0.9

Drug/Combo	CI value	Dose VGCV	Dose DTB
VGCV		116.010	
DTB			13.9795
COMBI2	0.15678	1.95606	1.95606

Data for Fa = 0.95

Drug/Combo	CI value	Dose VGCV	Dose DTB
VGCV		347.716	
DTB			26.6922
COMBI2	0.12985	3.21891	3.21891

Data for Fa = 0.97

Drug/Combo	CI value	Dose VGCV	Dose DTB
VGCV		759.324	
DTB			42.2909
COMBI2	0.11453	4.58808	4.58808

CompuSyn Report

**Experiment Name:**	VGCV-DTB-03
**Date:**	191003
**File Name:**	\\Lfdsrv003\groupe\PR\PR_Theses\CT_SAVE\Résultats ANVIE-SAVE\07_Combinaisons-CCL57\VGCV_DTB_COMBINAISON\CompuSyn qPCR\VGCV-DTB_03.cse
**Description**	

**Drug:**	Valganciclovir (VGCV) [μM]
**Drug:**	Decitabine (DTB) [μM]
**Drug Combo:**	VGCV-DTB (1:1) (COMBI3) (VGCV+DTB [1:1])

Data for Drug: VGCV [μM]

Dose	Effect
5.0	0.46
2.5	0.48
1.25	0.38
0.63	0.09
0.31	0.1
0.16	0.07
0.08	0.01

7 data points entered.

**X-int:**	0.54199
**Y-int:**	-0.5621 +/- 0.10697
**m:**	1.03707 +/- 0.16938
**Dm:**	3.48331
**r:**	0.93932

Data for Drug: DTB [μM]

Dose	Effect
10.0	0.62
5.0	0.66
2.5	0.49
1.25	0.2
0.63	0.13
0.31	0.08
0.16	0.02
0.08	0.02
0.04	0.01

9 data points entered.

**X-int:**	0.59320
**Y-int:**	-0.6128 +/- 0.05749
**m:**	1.03304 +/- 0.07195
**Dm:**	3.91923
**r:**	0.98344

Data for Drug Combo: COMBI3 (VGCV+DTB [1:1])

Dose A	Effect
5.0+	0.99
2.5+	0.87
1.25+	0.76
0.63+	0.32
0.31+	0.29
0.16+	0.24
0.08+	0.01

7 data points entered.

X-int: 0.09141

Y-int: -0.1693 +/- 0.15368

m: 1.85157 +/- 0.25322

Dm: 1.23427

r: 0.95628

Dose-Effect Curve

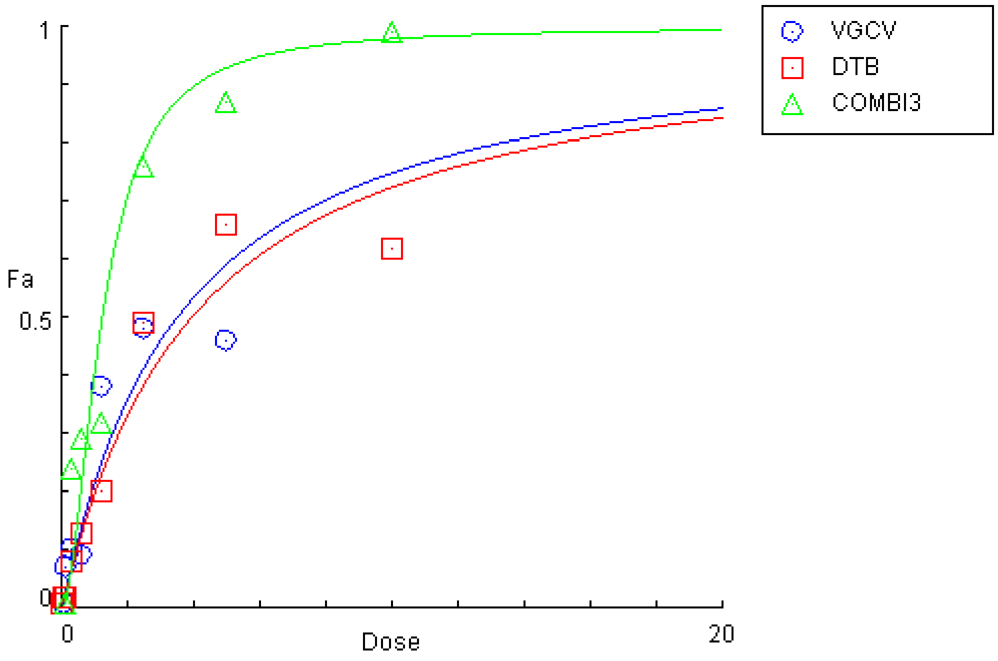

Median-Effect Plot

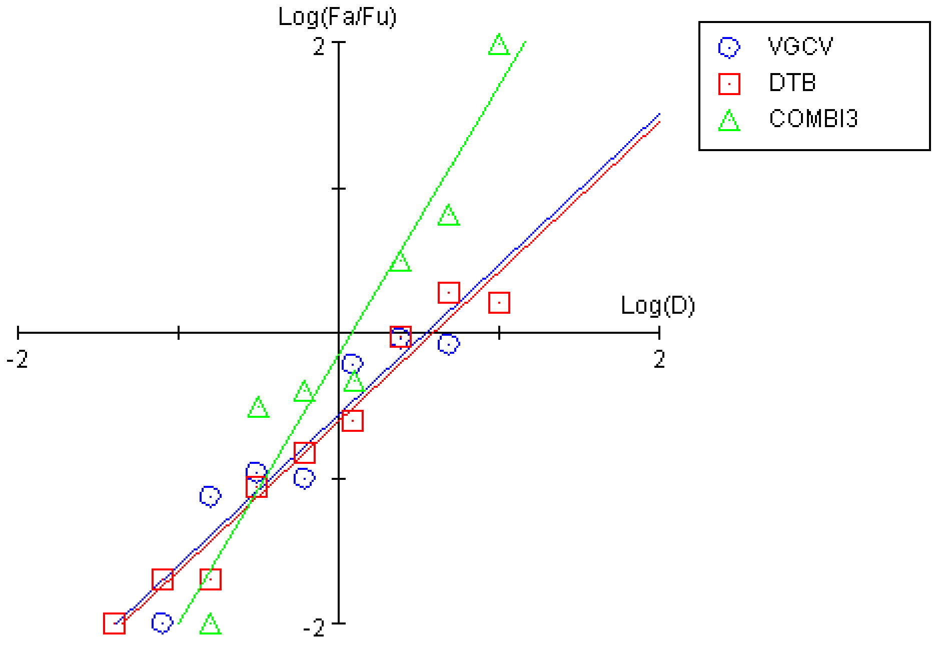

CI Data for Drug Combo: COMBI3 (VGCV+DTB [1:1])

Fa	CI Value	Total Dose
0.05	1.17288	0.25164
0.1	0.85315	0.37674
0.15	0.70056	0.48367
0.2	0.60397	0.58378
0.25	0.53432	0.68191
0.3	0.48007	0.78104
0.35	0.43560	0.88351
0.4	0.39772	0.99153
0.45	0.36449	1.10750
0.5	0.33463	1.23427
0.55	0.30722	1.37556
0.6	0.28156	1.53644
0.65	0.25707	1.72429
0.7	0.23326	1.95051
0.75	0.20958	2.23407
0.8	0.18541	2.60959
0.85	0.15984	3.14971
0.9	0.13126	4.04371
0.95	0.09548	6.05400
0.97	0.07613	8.06763

CI values for actual experimental points:

Total Dose	Fa	CI Value
10.0	0.99	0.03201
5.0	0.87	0.21607
2.5	0.76	0.22259
1.26	0.32	0.70756
0.62	0.29	0.39921

Total Dose	Fa	CI Value
0.32	0.24	0.26418
0.16	0.01	3.67391

Combination Index Plot

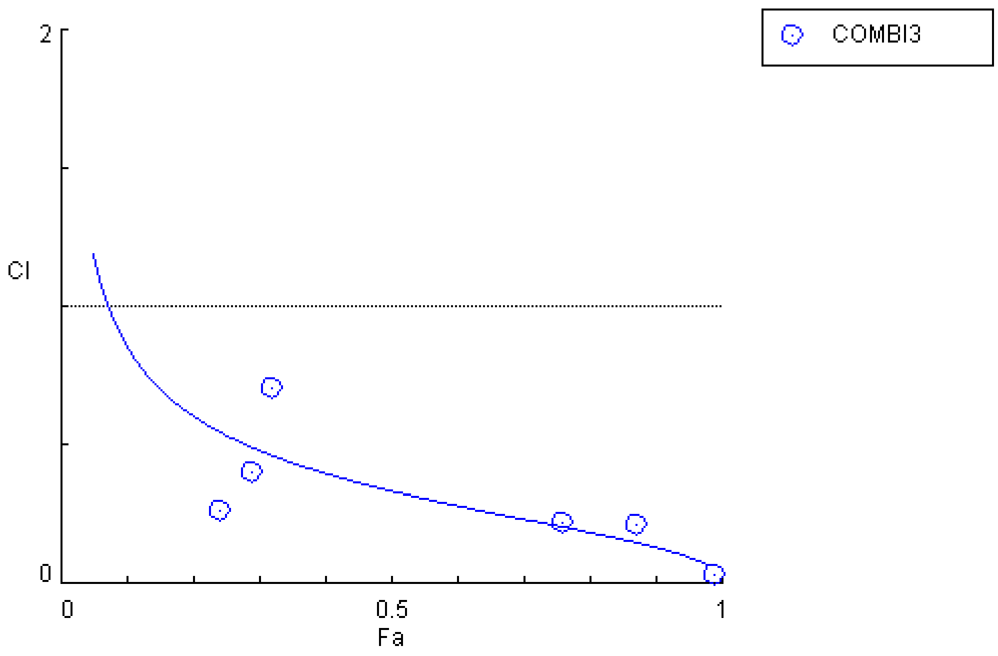

DRI Data for Drug Combo: COMBI3 (VGCV+DTB [1:1])

Fa	Dose VGCV	Dose DTB	DRI VGCV	DRI DTB
0.05	0.20368	0.22664	1.61882	1.80132
0.1	0.41866	0.46717	2.22253	2.48006
0.15	0.65402	0.73108	2.70440	3.02302
0.2	0.91507	1.02422	3.13497	3.50893
0.25	1.20761	1.35312	3.54184	3.96863
0.3	1.53875	1.72581	3.94026	4.41924
0.35	1.91760	2.15255	4.34084	4.87270
0.4	2.35611	2.64692	4.75245	5.33904
0.45	2.87050	3.22729	5.18375	5.82806
0.5	3.48331	3.91923	5.64431	6.35066
0.55	4.22695	4.75952	6.14578	6.92012
0.6	5.14978	5.80310	6.70353	7.55397
0.65	6.32743	7.13589	7.33917	8.27691
0.7	7.88524	8.90039	8.08530	9.12621
0.75	10.0475	11.3518	8.99481	10.1624
0.8	13.2596	14.9971	10.1622	11.4938
0.85	18.5520	21.0105	11.7802	13.3413
0.9	28.9817	32.8796	14.3342	16.2621
0.95	59.5709	67.7734	19.6798	22.3896
0.97	99.4667	113.389	24.6582	28.1097

DRI values calculated at experimental points

Fa	Dose VGCV	Dose DTB	DRI VGCV	DRI DTB
0.99	292.611	334.977	58.5221	66.9953

Fa	Dose VGCV	Dose DTB	DRI VGCV	DRI DTB
0.87	21.7799	24.6816	8.71197	9.87266
0.76	10.5852	11.9617	8.46817	9.56937
0.32	1.68397	1.88934	2.67297	2.99896
0.29	1.46903	1.64731	4.73882	5.31391
0.24	1.14626	1.28413	7.16415	8.02579
0.01	0.04147	0.04585	0.51833	0.57319

DRI Plot for Combo: COMBI3 (VGCV+DTB [1:1])

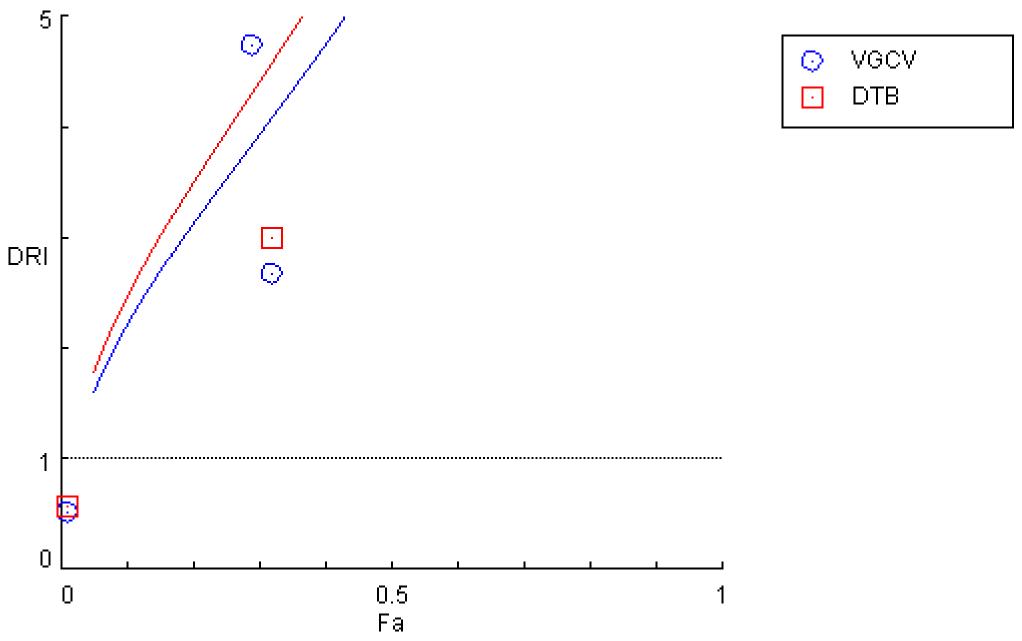

Summary Table

Experiment Name: VGCV-DTB-03

Date: 191003

File Name: \\Lfdsrv003\groupe\PR\PR_Theses\CT_SAVE\Résultats ANVIE-SAVE\07_Combinaisons-CCL57\VGCV_DTB_COMBINAISON\CompuSyn qPCR\VGCV-DTB_03.cse

Description

Drug: Valganciclovir (VGCV) [µM]
Drug: Decitabine (DTB) [µM]
Drug Combo: VGCV-DTB (1:1) (COMBI3) (VGCV+DTB [1:1])

Drug/Combo	Dm	m	r
VGCV	3.48331	1.03707	0.93932
DTB	3.91923	1.03304	0.98344
COMBI3	1.23427	1.85157	0.95628

	CI values at:			
**Combo**	**ED50**	**ED75**	**ED90**	**ED95**
COMBI3	0.33463	0.20958	0.13126	0.09548

Data for Fa = 0.5

Drug/Combo	CI value	Dose VGCV	Dose DTB
VGCV		3.48331	
DTB			3.91923
COMBI3	0.33463	0.61714	0.61714

Data for Fa = 0.75

Drug/Combo	CI value	Dose VGCV	Dose DTB
VGCV		10.0475	
DTB			11.3518
COMBI3	0.20958	1.11703	1.11703

Data for Fa = 0.9

Drug/Combo	CI value	Dose VGCV	Dose DTB
VGCV		28.9817	
DTB			32.8796
COMBI3	0.13126	2.02186	2.02186

Data for Fa = 0.95

Drug/Combo	CI value	Dose VGCV	Dose DTB
VGCV		59.5709	
DTB			67.7734
COMBI3	0.09548	3.02700	3.02700

Data for Fa = 0.97

Drug/Combo	CI value	Dose VGCV	Dose DTB
VGCV		99.4667	
DTB			113.389
COMBI3	0.07613	4.03381	4.03381

Reviewer #1:

In the paper 'Identification of antiviral compounds against equid herpesvirus-1 (EHV-1) using real-time cell assay screening: efficacy of decitabine and valganciclovir alone or in combination', Thieulent at al. have tested a huge battery of compounds that may have an antiviral effect against EHV-1 in three cell types (RK-13, ED, EEK). Since EHV-1 is a champion in evading the immunity, resulting in reproductive and respiratory poblems, antivirals are urgently needed. Therefore, this paper is a very important piece of research.

The work has been performed in an excellent way, with the necessary controls (controlled on cytotoxicity and three different EHV-1 strains). However, there are issues that have to be addressed before it can be published:

1° Introduction - p3, lines 41-50: 'While EHV-1 induced abortion storms have been prevented since the introduction of vaccination three decades ago…'
This is not correct. Abortions still occur in correctly vaccinated mares. This has to be adapted.

We agree with the reviewer and “prevented” has now been replaced by “reduced”.

2° Introduction - p4, lines 58-60: '…the use of antiviral treatment is sometimes considered to prevent severe forms of EHV-1 induced disease…'
This is difficult to follow. What do the authors mean with prevent severe disease? An antiviral will not be used in a preventive way. I think you should mention that one will use it for a treatment.

We agree with the reviewer and “to prevent severe forms of EHV-1 induced disease” has now been replaced by “for the treatment of EHV-1 induced disease”.

3° Material and methods - p6, lines 59-60: Controls cells
Should be 'Control cells'

This has been corrected.

4° Discussion - p13, line 14: 'valganciclovir'
Should be 'Valganciclovir'

This has been corrected.

5° Discussion - The authors should be very critical on their work in continuous cell lines. This should be discussed in the Discussion part. I would like to ask the authors to read the PhD thesis of Barbara Garré. In that work, it was clearly demonstrated that antiviral activity in continuous cell lines cannot be extrapolated to ex vivo work in respiratory mucosa explants and in vivo work.
Barbara Garré. 2008. Pharmacokintetics and clinical efficacy of acyclovir in the treatment of equine herpesvirus type 1 infections, Ghent University (can be downloaded for free from the digitial UGent library).

Indeed, we have to be more critical on results obtained on continuous cell lines. This is now clearly discussed lines 377-384 (Nevertheless, our results should be considered only as a

first step in this direction as the predictive value of *in vitro* models based on continuous cell lines is questionable. For example, the antiviral effect of aciclovir observed *in vitro* on cell lines infected by EHV-1 was not validated *ex vivo* when using respiratory mucosa explants as a model (Glorieux et al., 2012) and was not transposable *in vivo* using valaciclovir, the prodrug of aciclovir (Garre et al., 2009). Although data observed in this study are promising, further investigations are necessary on *ex vivo* models to confirm the antiviral effect of our lead compounds before *in vivo* experiments implementation.).

Reviewer #2:

The manuscript by Thieulent et al presents data on antiviral effect of 8 compounds from preselected effective 22 compounds that were selected from a screening of 2,897 compounds against Equid herpesvirus 1 (strain Kentucky D). Efficiency of antiviral activity was determined by real time cell analysis (RTCA) using the measurement of cellular "impedance" in cell culture for 96h, compared to a non-infected control. This method has been developed by this group in Thieulent et al., 2019 Virology (https://doi.org/10.1016/j.virol.2018.10.013).
The manuscript focuses on decitabine as a novel anti-viral compound that could be used in synergy with valgancyclovir against EHV-1 infection. The authors present interesting though not fully novel data, and needs major revision before publication.

Major concerns:

1. The manuscript relies on a pre-screening using RTCA methodology but there is no data showing any result from that screening of 2,897 compounds.

We agree with the reviewer remark. The data showing the results of the screening of 2,891 compounds are now presented graphically in supplementary figure 1 and detail of this screening is presented in the associated “Data in Brief” article (Screening of potential antiviral molecules against equid herpesvirus-1 using cellular impedance measurement: dataset of 2,891 compounds, C. Thieulent) as suggested by the editor.

2. How robust is the RTCA IC50 measurements compared to IC50 measured by qPCR? The authors should show the RTCA results for the 8 selected compounds and qPCR actual measurements rather than just IC50 values.

We have realised a Spearman correlation between EC50 values obtained by RTCA and qPCR for the 16 compounds presented in Table 1 with EC50s<50µM (Figure 1). The R factor of 0.841 and P-value <0.001 now presented lines 221-223 show a good correlation between EC50 values obtained by qPCR and RTCA. These data show the robustness of RTCA EC50 values. In addition, dose-responses curves obtained by RTCA and qPCR measurements for the 8 selected compounds were added in Figure 2 (Figure 1 in the previous version).

3. Figure 2 shows results of potential synergism between valganciclovir and decitabine, using two algorithm approaches. However, experiment in Fig2C is not clear. It seems cells were incubated with both VGCV and DTB compounds but no condition of single treatment was performed as control. If so, no conclusion can be made on any synergistic effect. This is not clear. The authors should provide in one experiment single-treatments and dual treatments.

Previous figure 2 is now figure 4. The figure 4C is obtained after Chou-Talalay method analysis using CompuSyn software. This method requires all the single treatment values associated with dual-treatments in order to calculate combination indexes (CIs) presented on the figure. We agree with the reviewer that the Chou-Talalay analysis and other procedures to assess synergistic effects were not detailed. This is due to space limitations and because these methods were previously described in literature. However and to address Reviewer's comment, please find attached the reports of the 3 experiments of combination using CompuSyn software (Experiment_01.pdf, Experiment_02.pdf and Experiment_03.pdf). Results allowing to obtained data in figure 4C are highlighted in yellow at the bottom of page 5 in the reports.

4. Figure 4 compares DTB with RG108 (a methyl transferase inhibitor) by RTCA and qPCR. This experiment does not include any control (mock-infected and mock-treated). They should be included.

Antiviral effect of DTB and RG108 are calculated using the percentage of inhibition based on results of mock-infected and mock-treated cells. The formula is now clearly presented in part 2.4 (lines 157-161; Percentage of inhibition was calculated using the following formula: Inhibition (%) = 100 × [1 – (a – b) ÷ (b – c)], where a corresponds to value of infected cells treated with different concentration of compounds, whereas b and c correspond to values obtained for mock-infected and mock-treated cells, respectively).

5. Figure 5 demonstrates that decitabine is a deoxycytidine analog and that the antiviral effect of this compounds is antagonized by deoxycytidine in excess. Although this information is important, it is an obvious hypothesis. Moreover, the abstract states that decitabine needs to be phosphorylated by DCK in order to be active against EHV-1. This is an overstatement. Although the authors refer to a published study showing the requirement of DCK-mediated phosphorylation of decitabine to be incorporated in cellular DNA, there is no data in the current manuscript supporting that such mechanism is required for antiviral effect against EHV-1. In the same line, the graphical abstract is not appropriately reflecting the results of the study. There is no result supporting that dC directly inhibits DCK-mediated phosphorylation of decitabine. The only section where a 'competition' mechanism is mentioned is in the discussion P16. This should be clarified to avoid misinterpretation of the data.

We agree with the reviewer that information presented in previous figure 5 (now Figure 7) is important. To our knowledge, this is the first demonstration that deoxycytidine can antagonize the antiviral effect of DTB and considering that DTB could act through off-target mechanisms, this result is not obvious. Our observation leads to the conclusion that dC is competing with DTB either at the phosphorylation step by DCK which is required for both dC and DTB incorporation in DNA, or a the level of DNA incorporation *per se*.

This was clarified by modifying several sentences to avoid misinterpretation:

- In abstract, "this study **demonstrated** that decitabine needs to be phosphorylated by deoxycytidine kinase in order to be active against EHV-1" has now been replaced by "this study **suggests** that decitabine needs to be phosphorylated by deoxycytidine kinase in order to be active against EHV-1".
- In graphical abstract, we have removed the indication that deoxyxytidine (dC) inhibited DTB effect. We suggest now a competition effect between dC and DTB for DCK as mentioned in discussion P16.

6. P12: the authors conclude that decitabine has a 'cell-protective' effect against EHV-1; but they show that deoxycytidine in excess reverses the antiviral effect. Thus, the effect is only potentially cell protective because EHV-1 replication is impaired and does not protect the cells on its own.

We agree with the reviewer and “cell-protective” has now been replaced by “antiviral effect” (line 299).

7. The authors do not discuss hypotheses related to the efficacy of decitabine as an analog of dC to inhibit EHV-1 replication in culture, and why dC analogs have apparently not been used against other herpesviruses.

This is now discussed in the discussion section.

8. There is no discussion related to the problematic of the absence of effect against latency-associated virus.
It is difficult to discusses the problematic of latency and antiviral treatment because of the absence of pertinent model for EHV-1.

Minor comments:

- Tables: the Excel format is not easily accessible, with footnotes being in the main text and structural information of compounds barely readable.

Structural conformations of compounds in table 1 were re-drawing with Chemdraw and are now more readable.

- P10: "RK" in "RK13" cells stands for "rabbit kidney". These cells are thus not rodent cells as rabbits are not rodents.

This has been corrected.

- Table 2, 3 and 4 would be much more accessible if graphed as bars +/- SD.

We agree with the reviewer and table 2 and 3 are now graphed as bars +/- SD in the new figure 3A and 3B. However, we proposed to maintain in a table form the previous presentation of “Combination analysis of compounds against EHV-1 KyD strain on E. Derm cells” (now table 2).

- P10: how do the authors explain the difference of efficacy in EEK and RK13 compared to E.Derm cells?

The difference of efficacy in different cell models is difficult to access for the different drugs. We proposed lines 355-358 an explanation for DTB and GTB. This point is also discussed in line 377-384.

- Table 4: Additive rather than Additif

This has been corrected.

- The discussion is of poor quality with successive repetitions of the results rather than actual discussion, interpretation and discussion of them.

In concordance with reviewers’ suggestions, the discussion has been carefully revised.

Highlights

- Real-time cell assay screening of 2,891~~7~~ compounds was conducted against EHV-1.

- 22 compounds were identified effective against EHV-1 *in vitro*.

- (Val)ganciclovir, decitabine, aphidicolin, idoxuridine and pritelivir are the most potent *in vitro* against EHV-1.

- Decitabine, a deoxycytidine analogue, demonstrated synergistic effect with valganciclovir against EHV-1.

- Decitabine phosphorylation by deoxycytidine kinase is required to be active.

 Click here to access/download;Graphical Abstract;Graphical abstract.tif

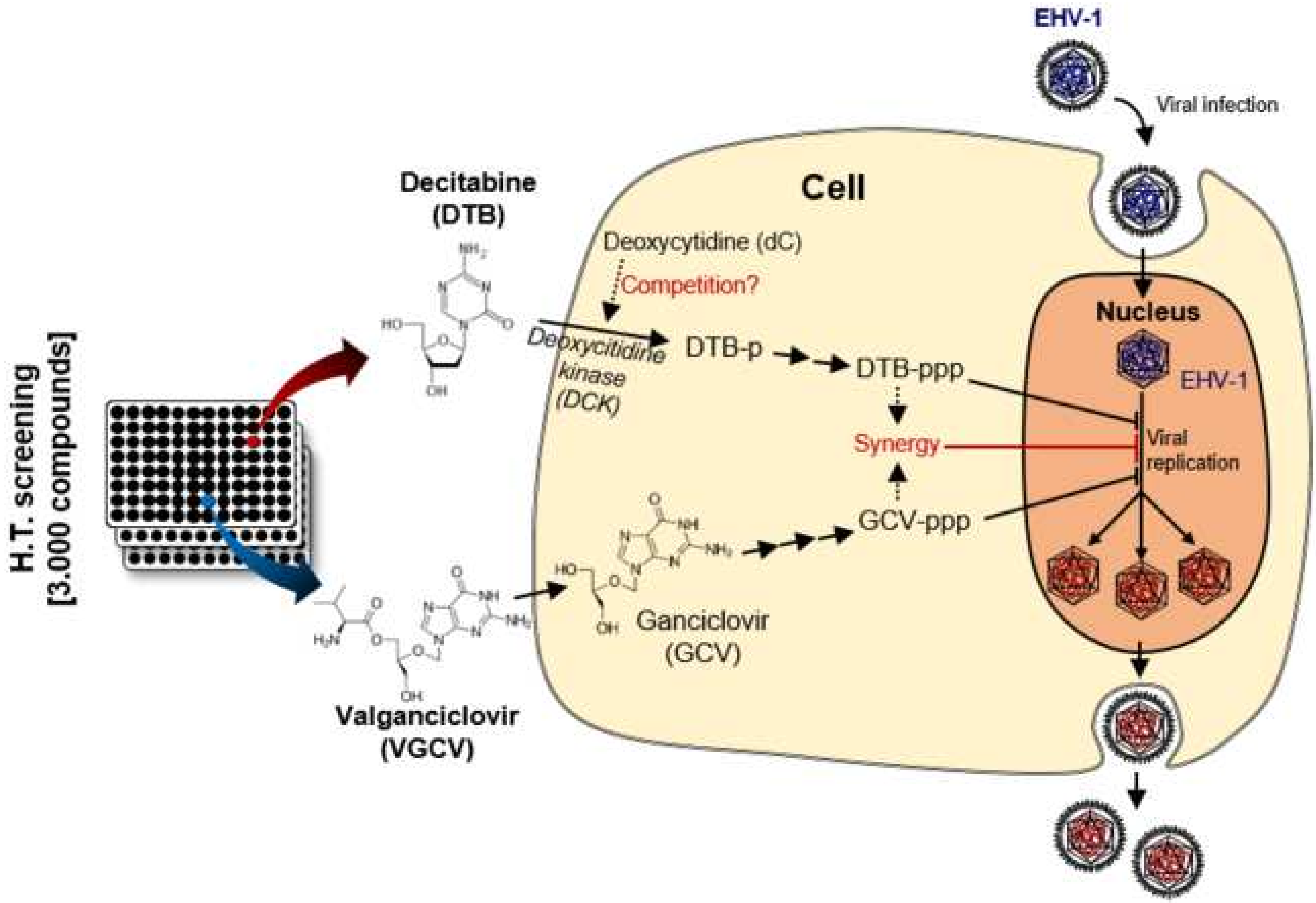

Manuscript File Click here to view linked References

Title:

Identification of antiviral compounds against equid herpesvirus-1 using real-time cell assay screening: efficacy of decitabine and valganciclovir alone or in combination.

List of authors:

Côme Thieulent [1,2], Erika Hue [1,2,3], Gabrielle Sutton [1,2], Christine Fortier [1,2,3], Patrick Dallemagne [4], Stephan Zientara [5], Hélène Munier-Lehmann [6], Aymeric Hans [7], Romain Paillot[1,2], Pierre-Olivier Vidalain [8,9] and Stéphane Pronost [1,2,3,§]

[1] LABÉO Frank Duncombe, 14280 Saint-Contest, France.

[2] Normandie Univ, UNICAEN, BIOTARGEN EA7450, 14280 Saint-Contest, France.

[3] Normandie Univ, UNICAEN, ImpedanCELL, 14280 Saint-Contest, France.

[4] Normandie Univ, UNICAEN, CERMN, 14000 Caen, France.

[5] Université Paris-Est, Laboratoire de Santé Animale, ANSES, INRAE, ENVA, UMR 1161 Virologie, 94700 Maisons-Alfort, France.

[6] Institut Pasteur, Unité de Chimie et Biocatalyse, CNRS UMR 3523, 75015 Paris, France.

[7] ANSES, Laboratoire de santé animale, site de Normandie, PhEED Unit, 14430 Goustranville, France.

[8] CIRI, Centre International de Recherche en Infectiologie, Univ Lyon, Inserm U1111, Université Claude Bernard Lyon 1, CNRS UMR5308, ENS de Lyon, F-69007, Lyon, France.

[9] Equipe Chimie et Biologie, Modélisation et Immunologie pour la Thérapie (CBMIT), Université Paris Descartes, CNRS UMR 8601, 75006 Paris, France.

[§] Corresponding Author

Tel: +33 (0) 2 31 47 19 54

Fax: +33 (0) 2 31 47 19 00

Email: stephane.pronost@laboratoire-labeo.fr

LABÉO Frank Duncombe

1 route de Rosel, 14280 SAINT-CONTEST, France

Abstract

Equid herpesvirus-1 infections cause respiratory, neurological and reproductive syndromes. Despite preventive treatments with vaccines, resurgence of EHV-1 infection still constitutes a major threat to equine industry. However, no antiviral compound is available to treat infected horses. In this study, 2,891 compounds were screened against EHV-1 using impedance measurement. 22 compounds have been found to be effective *in vitro* against EHV-1. Valganciclovir, ganciclovir, decitabine, aphidicolin, idoxuridine and pritelivir (BAY 57-1293) are the most effective compounds identified, and their antiviral potency was further assessed on E. Derm, RK13 and EEK cells and against 3 different field strains of EHV-1 (ORF30 2254A/G/C). We also provide evidences of synergistic interactions between valganciclovir and decitabine in our *in vitro* antiviral assay as determined by MacSynergy II, isobologramm and Chou-Talalay methods. Finally, we showed that deoxycytidine reverts the antiviral effect of decitabine, thus supporting some competition at the level of nucleoside phosphorylation by deoxycytidine kinase and/or DNA synthesis. Deoxycitidine analogues, like decitabine, is a

family of compounds identified for the first time with promising antiviral efficacy against herpesviruses.

Keywords

Real-time cell assay; Chemical library screening; Antiviral; Equid herpesvirus-1; ganciclovir; decitabine; synergism

1. Introduction

Herpesviruses (order *Herpesvirales*, family *Herpesviridae*) are enveloped viruses with a linear, double-stranded DNA genome of 125-290 kb. Among the five equid herpesviruses (EHV-1 to 5) frequently isolated in horses, EHV-1 is the most pathogenic and is endemic worldwide. EHV-1 infection in horses is associated with several clinical signs of disease, from usually mild respiratory distress, cough and discharge, to more severe secondary forms of diseases such as abortion, neonatal foal death and equine herpes myeloencephalopathy (EHM) (Allen, 2002). The prevalence of latent EHV-1 is estimated to be greater than 60% in horse population (Lunn et al., 2009).

Several vaccines are available against EHV-1. Their use reduces clinical signs of respiratory disease and virus shedding, which limits the extent of outbreaks. However, the protection provided against the secondary forms of the disease presents some limitations. While EHV-1 induced abortion storms have been reduced since the introduction of vaccination three decades ago, none of the commercially available EHV-1 vaccines have demonstrated its efficacy to prevent EHM. EHV-1 vaccine coverage is often too low to provide effective herd immunity. In this context, outbreaks still occur worldwide in horse populations. A recent outbreak reported in France in 2018 (Sutton et al., 2019), led to the cancellation of more than

200 horse competitions, thus generating large economic losses for the French equine industry. To complement prevention measures, such as vaccination and biosecurity, the use of antiviral treatment is sometimes considered for the treatment of severe EHV-1 diseases, especially EHM. The occasional use of aciclovir during EHV-1 outbreaks has been reported (Friday et al., 2000; Henninger et al., 2007; Murray et al., 1998) but the therapeutic efficacy of this compound is difficult to assess in the absence of untreated animals as a control. Two experimental infections in horses treated with valaciclovir, an aciclovir pro-drug, have also shown divergent results (Garre et al., 2009; Maxwell et al., 2008).

EHV-1 is an alphaherpesvirus genetically closely related to herpes simplex virus type 1 (HHV-1) and varicella zoster virus (HHV-3) for which antiviral therapies are available. However, the emergence of human herpesvirus strains resistant to antiviral treatments, such as aciclovir, has motivated researches for new antiviral therapies (Jiang et al., 2016) and helicase primase inhibitors seem to be good candidates (James et al., 2015; Kleymann et al., 2002). Over the last two decades, drug repositioning has proven to be an effective strategy to meet therapeutic needs with nearly a hundred drugs repositioned since (Jourdan et al., 2020). Even in absence of approved EHV-1 antiviral treatment for practitioners, few antiviral molecules have been studied against EHV-1 *in vitro* and correspond to those already used in human medicine against herpesviruses such as aciclovir, ganciclovir, cidofovir and penciclovir (Maxwell, 2017; Vissani et al., 2016). Other compounds such as aphidicolin (Goodman et al., 2007), A-5021 (Glorieux et al., 2012), quercetin (Ferreira et al., 2018; Gravina et al., 2011) and the histone demethylase inhibitor OG-L002 (Tallmadge et al., 2018) have been studied against EHV-1 in different cell culture models. However, these molecules have never been tested in a standardised cellular model allowing proper comparisons.

We have recently developed a standardised Real-Time Cell Analysis (RTCA) model for evaluating the effect of antiviral compounds against EHV-1. This system relies on the

measurement of cellular impedance in culture wells, which reflects cellular adhesion and proliferation. Results are expressed as Cell Index (CI) that enables a standardised and accurate analysis of EHV-1 cytopathic effects. This system has proven successful to determine the efficacy of molecules against EHV-1 such as spironolactone (Thieulent et al., 2019). In the present work, a chemical library of 2,891 compounds comprising new chemical entities and FDA-approved drugs has been screened by impedancemetry to identify compounds against the EHV-1 Kentucky D (KyD) reference strain. As some associations between a DNA polymerase (ORF30) genotype (G/A at position 2254) and the type of disease have been reported by several studies (Goodman et al., 2007; Lunn et al., 2009; Nugent et al., 2006; Pronost et al., 2010), active molecules identified were subsequently tested against a panel of EHV-1 strains (A_{2254} or G_{2254}), including the newly identified EHV-1 ORF30 variant (C_{2254}) (Paillot et al., 2020). Decitabine was one of the most effective molecules identified, and the mode of action of this cytidine analogue has been further investigated.

2. Materials and methods

2.1. Cell lines

Equine dermal fibroblasts (E. Derm, NBL-6 ATCC® CCL-57, Manassas, VA), equine embryonic kidney cells (EEK, kindly provided by Merial, France) and rabbit kidney cells (RK13, ATCC® CCL-37™) were used in this study. E. Derm cells were maintained in Eagle's Minimum Essential Medium (ATCC®) and seeded at 1.2×10^4 cells/well in 96-well plates. EEK cells were maintained in MEM Alpha (Biowest, Nuaillé, France) supplemented with 2% Lactalbumin hydrolysate (Sigma, St. Quentin Fallavier, France), 1% L-glutamine (Eurobio, Courtaboeuf, France), 0.5% D-Glucose (Sigma) and seeded at 1.2×10^4 cells/well in 96-well plates. RK13 cells were maintained in EMEM with Earle’s salts (Eurobio) supplemented with 1% L-glutamine (Eurobio) and seeded at 4.8×10^4 cells/well for 96-well plates. All media

contained 10% fetal bovine serum (Eurobio), 100 IU/mL penicillin, 0.1 mg/mL streptomycin and 0.25 µg/mL amphotericin B (Eurobio) and were cultivated at 37 °C and 5% CO_2.

2.2. EHV-1 strains

The EHV-1 Kentucky D (KyD) strain (ATCC® VR700™) was used as the EHV-1 reference strain for compound screening and subsequently to confirm the antiviral effect of selected hits in the different cell lines. In addition, three French EHV-1 strains were also used in this study, including the ORF30 G_{2254} EHV-1 strain (FR-38991) isolated in 2009 from a horse with neurological disorders (LABÉO, France; nasal swab), the ORF30 A_{2254} EHV-1 strain (FR-6815) isolated in 2013 from lung biopsies of an aborted foetus (LABÉO, France) and the ORF30 C_{2254} EHV-1 strain (FR-56628) isolated in 2018 from PBMC of a horse with respiratory disorders (LABÉO, France) (Paillot et al., 2020). E. Derm and RK13 cells were infected with the KyD strain at MOIs of 0.01 and 0.04, respectively. EEK cells were infected with the four different EHV-1 strains at a MOI of 0.05.

2.3. Compounds

This study includes 2,891 compounds from three different libraries: i) 1,199 compounds from the Prestwick® Chemical Library, containing mostly US Food and Drug Administration approved drugs (Prestwick Chemical, Illkirch, France) provided at 2 mg/mL in DMSO; ii) 1,651 compounds from the Centre d'Etudes et de Recherche sur le Médicament de Normandie (CERMN, Caen, France) provided at 10 mM in DMSO; iii) 41 compounds (called herein in-house antiviral library) selected for their effects against different human viruses and dissolved at 10 mM in DMSO (Supplementary Table 1). RG108 (MedChemExpress) was dissolved at 20 mM in DMSO. All compounds were stored at -20°C before used.

2.4. Screening of compound libraries using the RTCA system

The screening by impedancemetry was performed with EHV-1 KyD-infected E. Derm cells using the RTCA MP system (ACEA Biosciences Inc., San Diego, CA, USA) as previously

described (Thieulent et al., 2019). Control cells were treated with 0.5% DMSO in presence or absence of the virus. The screening was performed under blind conditions and 80 compounds were tested by plate at a final concentration of 10 µg/mL (Preswitck® Chemical Library), 10 µM (CERMN library) or 50, 10, 2 and 0.4 µM (in-house antiviral library) in 0.5% DMSO. Each plate includes the controls required for calculation of the Z'-factor (Zhang et al., 1999). Only plates with a Z' factor upper than 0.5 were considered for further analysis as previously described by Thieulent et al. (2019). For each compound, the area under normalised Cell Index (CI) curves was calculated from 0 to 96 hours post-infection (hpi) (AUCn; (Pan et al., 2013). The time required for the CI to decrease by 50% after virus infection was also determined (CIT_{50}; (Fang et al., 2011), and compared with controls. All the details are presented in Data in Brief (Thieulent et al., *submitted*). Any increase in these two parameters reflects some protection of E. Derm cells from EHV-1 induced cytopathic effects. The cut-off determined for a molecule to be considered with an antiviral potential were (i) the AUCn increasing by 25%, and (ii) the CIT_{50} being delayed by >8 h as compared to non-treated cells (Thieulent et al., 2019).

After the screening, dose-response curves were obtained for each selected compound by using percentage of inhibition calculation. The following formula was used: Inhibition (%) = 100 × [1 – (a – b) ÷ (b – c)], where a corresponds to the value of infected cells treated with different concentrations of compounds, whereas b and c correspond to values obtained for mock-infected and mock-treated cells, respectively.

2.5. Viral quantitation by qPCR assay

Cells were seeded and treated in 96-well plates as described in part 2.4. At 48 hpi, plates were frozen at -20°C to allow virus load quantitation in culture wells. After one cycle of freeze/thaw, nucleic acids were extracted using the QIAamp® Viral RNA Mini Kit (Qiagen, Courtaboeuf, France) according to the manufacturer's instructions and stored at -20 °C until

used. Quantitative PCR for EHV-1 was processed as previously described (Thieulent et al., 2019). Each thermal cycling was performed on a QuantStudio™ 12 K Flex Real- Time PCR System (Life Technologies).

2.6. Toxicity measurement

Cells were seeded in white opaque 96-well plates and after 24 h of culture, were treated with compounds. Cell viability was measured at 48 h post-treatment (hpt) by impedancemetry and ATP measurement using the CellTiter Glo® Luminescent Cell Viability Kit (CTG; Promega, Charbonnière-les-bains, France), according to the manufacturer's instructions. Luminescence signal was acquired using an Infinite® M200 luminometer (Tecan, Lyon, France).

2.7. Research of synergistic effects between compounds against EHV-1

Drug combinations were tested on EHV-1 KyD-infected E. Derm cells using impedancemetry as a read out. For each combination, the two selected drugs were prepared separately by 2-fold serial dilution and mixed in 96-well plates to create an 8 by 10 matrix of single and combined diluted drugs. For each compound, the dilution range was designed to have the EC_{50} in the middle of the range, and the highest concentration inferior to the EC_{90}. In each plate, infected and non-infected cells with 1% DMSO were used as positive and negative controls, respectively. Synergistic or antagonistic effects were determined with the MacSynergy II program using first the Bliss independence model (Prichard and Shipman, 1990) applied on AUC_n values. This software calculated the volume of synergy/antagonism produced by the drug combination in a 95% confidence interval. Volumes were given as the area under a dose-response curve in the two dimensional situation (μM^2 %) and interpretation was made as previously described by Prichard et al. (1990). Values of 0-25, 25-50, 50-100, and >100 μM^2 % in either a positive or negative direction were defined as additive, minor synergy or antagonism, moderate synergy or antagonism, and strong synergy or antagonism, respectively.

Isobologram analysis and the Chou-Talalay method using the Loewe additivity model were used to confirm synergistic effect firstly observed by MacSynergy II program. Isobolograms were built as previously described by Feng et al. (2009) from EC_{50} values obtained by impedance measurement. The Chou-Talalay method is based on the median-effect equation and computed by CompuSyn software version 1.0 (ComboSyn, Inc., Paramus, New Jersey) (Chou and Talalay, 1984). The software extrapolated a combination index representing the interaction between two drugs from the percentage of inhibition of $\log_{10}$ viral genome copies number produce in presence of each drug alone and in combination. The weighted average combination index (CI_{wt}) value was calculated as previously described (Drouot et al., 2016).

2.8. Statistical analysis

EC_{50} and CC_{50} values were calculated using a non-linear regression dose response inhibition curve (GraphPad Prism® software 6.0; La Jolla, CA, USA). The Selectivity Index (SI) was determined for each compound using the following formula: SI = CC_{50}/EC_{50}. Spearman's correlation test and values comparison using ANOVA with Tukey post hoc test were both statistically evaluated using GraphPad Prism® software.

3. Results

3.1. Screening and selection of the most effective compounds against EHV-1

To identify novel antiviral compounds against EHV-1, three chemical libraries (*i.e.* Prestwick® Chemical, CERMN and in-house antiviral libraries; see Material and Methods for details) were screened by impedance-based Real-Time Cell Analysis (RTCA) using equine dermal (E. Derm) cells infected with EHV-1 KyD strain as model (Supplementary Figure 1). *Z*'-factor values of the 38 screening 96-well plates were between 0.52 to 0.91, with a median of 0.71, insuring the robustness of our assay. Based on CIT_{50} and AUC_n values, 25 out of

2,891 compounds were identified with a potential antiviral activity (hit detection rate of 0.9%) against EHV-1 (14, 1 and 10 in the three chemical libraries, respectively).

These molecules were then evaluated in dose-response assays on E. Derm cells by two-fold serial dilution (50 to 0.1 µM) by qPCR assay and impedance measurement. Antiviral properties were confirmed for 22 out of the 25 compounds (Table 1), and EC_{50} values were precisely determined by both qPCR and RTCA for 16 of them (EC_{50}s<50 µM). The R factor of 0.841 and P-value <0.001 obtained by Spearman's correlation showed a good correlation between EC_{50} values obtained by qPCR and RTCA for these 16 molecules (Figure 1). Among these compounds, eight were selected as they complied with more stringent criteria: (i) the absence of toxicity on E. Derm cells at all the concentrations tested (CC_{50} > 50µM) and (ii) EC_{50} values below 50 µM at all time points between 48 and 120 hpi (Figure 2A). Dose-responses curves obtained by qPCR and RTCA at 48 hpi for the eight compounds are presented in Figure 2B. Three of these compounds were acyclic guanosine analogues (aciclovir, ACV; ganciclovir, GCV; and ganciclovir prodrug valganciclovir, VGCV) inhibiting the viral DNA polymerase, two were deoxycytidine analogues (decitabine, DTB; gemcitabine, GTB) used for tumor growth inhibition by incorporation in cellular DNA, and one was a deoxyuridine analogue (idoxuridine, IDU) targeting viral DNA synthesis. Aphidicolin (APD), a tetracyclic diterpene antibiotic, and pritelivir (BAY 57-1293), an inhibitor of the HHV-1 helicase-primase complex, were also selected.

3.2. Efficacy of eight selected compounds on different EHV-1 strains and cell lines

The antiviral activity of the eight selected compounds was further studied against EHV-1 KyD using two other cellular models: an equine cell line (EEK) and a rabbit cell line (RK13) and results were compared with data obtained on E. Derm cells (Figure 3A). Gemcitabine did not show any antiviral effect at tested concentrations on EEK and RK13 cells (EC_{50} > 50 µM). With EC_{50} ranging from 21.6 to 31.0 µM, aciclovir shows a weak antiviral activity on

the three cell models. The six other compounds (aphidicolin, pritelivir, decitabine, idoxuridine, ganciclovir and valganciclovir) showed a good efficacy on the three cell lines.

The efficacy of the eight selected compounds was tested against three different EHV-1 strains with distinct ORF30 genotypes (A/G/C_{2254}) on EEK cells (Figure 3B). This study confirmed the low activity of gemcitabine (EC_{50} > 50 µM). Aciclovir showed a lower activity against the FR-38991 strain (G_{2254}: 36.0 µM) when compared with its activity against FR-6815 (A_{2254}: 14.2 µM) or FR-56628 (C_{2254}: 7.9 µM). Quite similarly, pritelivir and idoxuridine showed a lower activity against FR-38991 strain (2.7 µM and 5.7 µM, respectively) when compared with its activity against FR-6815 (0.9 µM and 1.5 µM, respectively) or FR-56628 (1.0 µM and 1.5 µM, respectively). Consistent results were obtained with the other molecules across all EHV-1 strains.

3.3. Research of synergistic effect and antiviral activity of the valganciclovir/decitabine combination

Dual-combinations were tested between valganciclovir, one of the best candidates, and four other compounds (aphidicolin, pritelivir, decitabine and idoxuridine) for synergistic or antagonistic effects against EHV-1. Using MacSynergy II analysis, only the valganciclovir/decitabine combination showed a synergistic effect that is illustrated by the strong signal above additive effects in the matrix of drug interactions (Figure 4A). The synergy volume of 63.24 μM^2 % obtained supports a moderate synergy (Table 2). The peak of synergy was reached when both compounds were used at 0.63 µM (1:1 ratio). Likewise, evaluation of the combination valganciclovir/decitabine by the isobologram method indicated synergy with ADA values of -0.30 ($p < 0.001$) (Figure 4B). Results obtained by impedancemetry were also confirmed by viral genome copy number measurement at 48 hpi and median-effect analysis for concentrations of valganciclovir and decitabine used alone or

in combination at a 1:1 ratio. A synergistic effect was observed for the valganciclovir/decitabine combination as assessed by a weighted average combination index (CI_{wt}) of 0.20 (Figure 4C). The three other combinations (valganciclovir/aphidicolin, valganciclovir/ pritelivir, valganciclovir/ idoxuridine) tested were additive when measured by MacSynergy II method (Table 2) and were not tested by isobologram nor median-effect analysis. No cytotoxicity was observed at the maximal drug combinations tested for the four different combinations (Supplementary Figure 2).

3.4. Decitabine pre-treatment did not confer cell resistance to EHV-1 replication.

Although valganciclovir was developed as an antiviral against herpesviruses in the first place, this is not the case of decitabine. Indeed, decitabine is an anticancer agent which induces hypomethylation after integration in cellular DNA (Liu et al., 2007). To evaluate whether decitabine integration in target cell DNA provides protection from EHV-1 infection, cells were treated overnight with decitabine before infection and/or just after infection. Both results obtained by cell impedance measurement (Figure 5A) and EHV-1 viral load measurement (Figure 5B) showed that decitabine pre-treatment did not protect cells from CPE formation and virus replication. A post-infection treatment with decitabine was required to observe some significant inhibition of EHV-1 replication. The effect of RG108, another well know DNA methyltransferase inhibitor, was then tested against EHV-1 on E. Derm cells. RG108 did not show any antiviral effect when assessed by impedance measurement (Figure 6A) or virus load quantitation (Figure 6B). Altogether, this suggests that cellular DNA hypomethylation does not account for the inhibition of EHV-1 by decitabine.

3.5. Deoxycytidine competitively inhibits the antiviral effect of decitabine

Decitabine is a deoxycytidine analogue and a pro-drug that must be successively phosphorylated by components of the deoxyribonucleoside salvage pathway involving deoxycytidine kinase (DCK), CMP monophosphate kinase (in particular CMPK1) and

nucleotide diphosphate kinases (Momparler, 2005; Stresemann and Lyko, 2008). It has been shown that high levels of deoxycytidine (dC) can reverse the anticancer activity of gemcitabine, another dC analogue, by competition for DCK-mediated phosphorylation, the rate-limiting step in dC phosphorylation to dCTP (Halbrook et al., 2019). We thus tested if dC could similarly reverse the inhibitory effects of decitabine on EHV-1. Infected E. Derm cells were treated with decitabine in the presence of high concentrations of dC or other nucleosides including cytidine, uridine, adenosine, guanosine (Figure 7A). Of all tested nucleosides, only dC blocked the antiviral activity of decitabine. This result was confirmed by microscopic observations and impedancemetry as dC reversed the antiviral effect of decitabine against EHV-1 (Figure 7B and 7C). Altogether, these results demonstrate a competition of decitabine and dC for the same metabolic pathway in our *in vitro* infection model.

4. Discussion

In this study, 2,891 compounds were screened against EHV-1 by impedancemetry as previously described (Thieulent et al., 2019), and 22 compounds were identified for their antiviral properties against this virus. AUC_n values coupled to CIT_{50} calculation were the two major criteria for filtering raw data and identify hits. The antiviral effect of selected compounds was confirmed by dose-response assay using both impedancemetry and viral load quantitation. As the readouts were not the same, EC_{50} values obtained with these two methods differed as previously reported (Piret et al., 2016; Thieulent et al., 2019). However, the good correlation observed between EC_{50} values obtained by RTCA and qPCR demonstrates the pertinence of RTCA for antiviral evaluation. Among the 22 compounds inhibiting EHV-1, eight molecules were selected for further evaluations using stringent criteria, including EC_{50} values below 50 µM over time and lack of cytotoxicity when used at 50 µM.

Ganciclovir and aciclovir are approved medications to treat herpesviruses and were previously shown to be effective against EHV-1 *in vitro* (Garre et al., 2007; Thieulent et al., 2019).

Valganciclovir, the pro-drug and valine ester of ganciclovir, presents here an antiviral activity against EHV-1 similar to ganciclovir. Pritelivir is another antiviral drug developed to treat herpesviruses. It is an inhibitor of the helicase-primase complex of herpesviruses discovered in 2002 (Kleymann et al., 2002). The antiviral effect of pritelivir was previously reported against HHV-1 and HHV-2 (Betz et al., 2002). However, this study is the first demonstrating the antiviral effect of this molecule against equid herpesviruses and in particular against EHV-1. It would be interesting to evaluate the antiviral effect of these compounds against other equid herpesviruses, such as EHV-3. Idoxuridine is also a well-known antiviral compound against human herpesviruses such as HHV-1 and HHV-2, and is also active against different animal herpesviruses such as feline herpesvirus type-1 (De Clercq and Li, 2016; Maggs and Clarke, 2004). Interestingly, idoxuridine is one of the three deoxyuridine analogues, together with brivudine and trifluridine, which have been used for decades against herpes simplex viruses (De Clercq and Li, 2016). Idoxuridine and trifluridine have showed a good efficacy against EHV-1 without toxicity in our cellular model, whereas brivudine was inactive (data not shown). Brivudine is the only one that needs to be specifically phosphorylated by viral thymidine kinase (TK) to become active (De Clercq and Li, 2016), suggesting that EHV-1 TK is unable to phosphorylate brivudine, which is in line with previous reports (De Clercq, 1984; Kit et al., 1987). Maribavir is also a new nucleoside analogue in development against human cytomegalovirus (HHV-5, a betaherpesvirus) that was tested in our screen and was inactive against EHV-1 (data not show) (Price and Prichard, 2011). This result is in line with the lack of activity against the alphaherpesviruses HHV-1, HHV-2 and HHV-3 (Williams et al., 2003). Finally, our study showed that three deoxycytidine analogues, *i.e.* decitabine, gemcitabine and cytarabine, are all effective against EHV-1 infection *in vitro*. Decitabine and gemcitabine are respectively used in the treatment of acute myeloid leukemia (He et al., 2017) and recurrent ovarian cancer (Berg et al., 2019). Gemcitabine was previously characterized for its antiviral

activity against a broad spectrum of RNA viruses (Shin et al., 2018). It has also been reported to be effective against HHV-1 (Denisova et al., 2012). However, to our knowledge, our work is the first reporting the antiviral effect of decitabine against herpesvirus. Of these three deoxycitidine analogues, cytarabine has the lowest activity with an EC_{50} of 4.1 µM as determined by qPCR assay on E. Derm cells. Decitabine and gemcitabine were more potent EHV-1 inhibitors in this cellular model with EC_{50s} of 1.1 µM and 0.7 µM, respectively.

The antiviral activity of the 8 most efficient molecules was also validated in three cell lines and against different strains of EHV-1. E. Derm cells and EEK cells are both equine cell lines and most adapted to identify new antiviral compounds in equid species, especially EEK that was derived from a horse foetus that is one of the target of EHV-1 (Léon et al., 2008; Smith et al., 2010). Even though RK13 cells are not equine cells, they have been most frequently used in EHV1 antiviral studies (Azab et al., 2010; de la Fuente et al., 1992; Gibson, 1992; Rollinson, 1987). All compounds except aciclovir and gemcitabine showed some consistent antiviral activity in the three different cell lines. Aciclovir is the least active of the eight selected compounds, and was inactive when used with EEK cells. More surprisingly, although gemcitabine is very effective on E. Derm cells, it has no antiviral activity on RK13 and EEK cells. This suggests that gemcitabine is not properly phosphorylated by RK13 and EEK cells kinases. In line with this hypothesis, decitabine, which is structurally very close to gemcitabine and also needs to be phosphorylated, is less active on EEK and RK13 cells. Nevertheless, decitabine still exhibits a good efficacy in all three cellular models. The antiviral activity of the eight selected molecules was also evaluated on EEK cells infected with three different EHV-1 strains isolated during outbreaks in France. Each strain exhibits different nucleotide (A/G/C) at position 2254 of ORF-30 (DNA polymerase). EC_{50} values of the compounds were close, independently of the strain used. This suggests that the mutations in the palm domain did not affect the effect of the selected molecule in agreement with

previous reports comparing the susceptibility of A_{2254} and G_{2254} strains (Garre et al., 2007; Thieulent et al., 2019). Only aciclovir, pritelivir and idoxuridine were slightly less efficient on the FR-38991 (A_{2254}) strain. No difference of susceptibility was observed between the three strains for aphidicolin treatment. This result differs from a previous report showing that a strain with the G_{2254} genotype is more sensitive to aphidicolin than a strain with the A_{2254} genotype (Goodman et al., 2007).

In this study, ganciclovir and its prodrug valganciclovir are the most effective compounds *in vitro* against EHV-1. Interestingly, the pharmacokinetic of valganciclovir was previously studied in horse (Carmichael et al., 2013), showing 40% bioavailability after oral administration. This positions valganciclovir as the best candidate in our short list of active molecules for treating horses infected by EHV-1 even if the cost of the molecule could be a limitation. Nevertheless, our results should be considered only as a first step in this direction as the predictive value of *in vitro* models based on continuous cell lines is questionable. For example, the antiviral effect of aciclovir observed *in vitro* on cell lines infected by EHV-1 was not validated *ex vivo* when using respiratory mucosa explants as a model (Glorieux et al., 2012) and was not transposable *in vivo* using valaciclovir, the prodrug of aciclovir (Garre et al., 2009). Although data observed in this study are promising, further investigations are necessary on *ex vivo* models to confirm the antiviral effect of our lead compounds before *in vivo* experiments implementation.

This report is also the first analysis of drug combinations against EHV-1. Of all the combinations tested, only valganciclovir plus decitabine showed a synergic effect. It was previously demonstrated that gemcitabine in association with ganciclovir enhanced the antitumoral effects of HSV-TK suicide gene in a synergistic manner (Wang et al., 2016). This result is in agreement with our study.

To our knowledge, decitabine has never before been reported to inhibit cellular infections by a herpesvirus. This led us to further investigate the mode of action of this compound. The pre-treatment of cells with decitabine did not provide antiviral effects against EHV-1, suggesting that decitabine incorporation in cellular DNA did not mediate the antiviral effect of decitabine. This rather suggests that the antiviral effect of decitabine depend on its incorporation into viral DNA and/or some interference with the viral polymerase. Decitabine is well-known for preventing DNA methylation and this account for its antitumoral properties (Atallah et al., 2007; Schmelz et al., 2005). We thus tested the antiviral effect of RG108, which is a hypomethylation agent acting differently through DNA methyltransferase inhibition. The absence of RG108 activity against EHV-1 suggests that the inhibitory effect of decitabine against EHV-1 is not mediated by viral or cellular DNA hypomethylation. Finally, we showed that the addition of dC to culture medium inhibits decitabine antiviral activity. This strongly suggests that dC and decitabine channel through the same metabolic pathway, including phosphorylation-dependent activation and incorporation into cellular and viral DNA. Based on collected observations, we propose that decitabine is integrated into the EHV-1 DNA and/or jams the viral polymerase, thus leading to the inhibition of viral growth. Interestingly, and as opposed to ganciclovir that needs activation by viral TK (Sullivan et al., 1992), decitabine phosphorylation only relies on cellular kinases to be activated and may represent a good candidate against viral strains resistant to ganciclovir.

In conclusion, the antiviral effect of ganciclovir/valganciclovir and aphidicolin was confirmed against EHV-1. Most importantly, new EHV-1 inhibitors were identified, including idoxuridine, pritelivir and decitabine. The synergy observed between valganciclovir and decitabine is particularly interesting due to the complementarity of their mode of actions, and further investigations *ex vivo* and *in vivo* are warranted.

Conflict of interest

The authors declare no competing interests.

Acknowledgments

This work was supported by LABÉO, IFCE (Institut Français du Cheval et de l'Equitation, project AMIE), Fonds Eperon (project N87-2014, N07-2015, N07-2016, N13-2017 and N62-2017), Région Normandie (CPER R25 P3) and CENTAURE European project co-funded by Normandy County Council, European Union in the framework of the ERDF-ESF operationnal programme 2014-2020. We would like to thank Laurent Lemaitre from Boehringer Ingelheim, France who kindly provided us with EEK cells. We thank Christophe Denoyelle and Emilie Brotin from ImpedanCELL, Normandie Univ, UNICAEN, Caen, France for interacting and for their technical assistance with RTCA technology. We also thank all collaborators of the SAVE project.

References

Allen, G.P., 2002. Respiratory Infections by Equine Herpesvirus Types 1 and 4, 2002. Int. Vet. Inf. Serv.

Atallah, E., Kantarjian, H., Garcia-Manero, G., 2007. The role of decitabine in the treatment of myelodysplastic syndromes. Expert Opin. Pharmacother. 8, 65–73. https://doi.org/10.1517/14656566.8.1.65

Azab, W., Tsujimura, K., Kato, K., Arii, J., Morimoto, T., Kawaguchi, Y., Tohya, Y., Matsumura, T., Akashi, H., 2010. Characterization of a thymidine kinase-deficient mutant of equine herpesvirus 4 and in vitro susceptibility of the virus to antiviral agents. Antiviral Res. 85, 389–395. https://doi.org/10.1016/j.antiviral.2009.11.007

Berg, T., Nøttrup, T.J., Roed, H., 2019. Gemcitabine for recurrent ovarian cancer - a systematic review and meta-analysis. Gynecol. Oncol. 155, 530–537. https://doi.org/10.1016/j.ygyno.2019.09.026

Betz, U.A.K., Fischer, R., Kleymann, G., Hendrix, M., Rubsamen-Waigmann, H., 2002. Potent In Vivo Antiviral Activity of the Herpes Simplex Virus Primase-Helicase Inhibitor BAY 57-1293. Antimicrob. Agents Chemother. 46, 1766–1772. https://doi.org/10.1128/AAC.46.6.1766-1772.2002

Carmichael, R.J., Whitfield, C., Maxwell, L.K., 2013. Pharmacokinetics of ganciclovir and valganciclovir in the adult horse. J. Vet. Pharmacol. Ther. 36, 441–449. https://doi.org/10.1111/jvp.12029

Chou, T.-C., Talalay, P., 1984. Quantitative analysis of dose-effect relationships: the combined effects of multiple drugs or enzyme inhibitors. Adv. Enzyme Regul. 22, 27–55. https://doi.org/10.1016/0065-2571(84)90007-4

De Clercq, E., 1984. The antiviral spectrum of (£)-5-(2-bromovinyl)-2 ’-deoxyuridine. J. Antimicrob. Chemother. 14, 85–95.

De Clercq, E., Li, G., 2016. Approved Antiviral Drugs over the Past 50 Years. Clin. Microbiol. Rev. 29, 695–747. https://doi.org/10.1128/CMR.00102-15

de la Fuente, R., Awan, A.R., Field, H.J., 1992. The acyclic nucleoside analogue penciclovir is a potent inhibitor of equine herpesvirus type 1 (EHV-1) in tissue culture and in a murine model. Antiviral Res. 18, 77–89.

Denisova, O.V., Kakkola, L., Feng, L., Stenman, J., Nagaraj, A., Lampe, J., Yadav, B., Aittokallio, T., Kaukinen, P., Ahola, T., Kuivanen, S., Vapalahti, O., Kantele, A., Tynell, J., Julkunen, I., Kallio-Kokko, H., Paavilainen, H., Hukkanen, V., Elliott, R.M., De Brabander, J.K., Saelens, X., Kainov, D.E., 2012. Obatoclax, Saliphenylhalamide, and Gemcitabine Inhibit Influenza A Virus Infection. J. Biol. Chem. 287, 35324–35332.

Drouot, E., Piret, J., Boivin, G., 2016. Artesunate demonstrates in vitro synergism with several antiviral agents against human cytomegalovirus. Antivir. Ther. 21, 535–539. https://doi.org/10.3851/IMP3028

Fang, Y., Ye, P., Wang, X., Xu, X., Reisen, W., 2011. Real-time monitoring of flavivirus induced cytopathogenesis using cell electric impedance technology. J. Virol. Methods 173, 251–258. https://doi.org/10.1016/j.jviromet.2011.02.013

Feng, J.Y., Ly, J.K., Myrick, F., Goodman, D., White, K.L., Svarovskaia, E.S., Borroto-Esoda, K., Miller, M.D., 2009. The triple combination of tenofovir, emtricitabine and efavirenz shows synergistic anti-HIV-1 activity in vitro: a mechanism of action study. Retrovirology 6, 44. https://doi.org/10.1186/1742-4690-6-44

Ferreira, C.G.T., Campos, M.G., Felix, D.M., Santos, M.R., Carvalho, O.V. de, Diaz, M.A.N., Fietto, J.L.R., Bressan, G.C., Silva-Júnior, A., Almeida, M.R. de, 2018. Evaluation of the antiviral activities of Bacharis dracunculifolia and quercetin on Equid herpesvirus

1 in a murine model. Res. Vet. Sci. 120, 70–77. https://doi.org/10.1016/j.rvsc.2018.09.001

Friday, P.A., Scarratt, W.K., Elvinger, F., Timoney, P.J., Bonda, A., 2000. Ataxia and paresis with equine herpesvirus type 1 infection in a herd of riding school horses. J. Vet. Intern. Med. 14, 197–201.

Garre, B., Gryspeerdt, A., Croubels, S., De Backer, P., Nauwynck, H., 2009. Evaluation of orally administered valacyclovir in experimentally EHV1-infected ponies. Vet. Microbiol. 135, 214–221. https://doi.org/10.1016/j.vetmic.2008.09.062

Garre, B., Vandermeulen, K., Nugent, J., Neyts, J., Croubels, S., Debacker, P., Nauwynck, H., 2007. In vitro susceptibility of six isolates of equine herpesvirus 1 to acyclovir, ganciclovir, cidofovir, adefovir, PMEDAP and foscarnet. Vet. Microbiol. 122, 43–51. https://doi.org/10.1016/j.vetmic.2007.01.004

Gibson, J.S., 1992. The activity of (S)-l-[(3-hydroxy-2-phosphonyl methoxy) propyl] cytosine (HPMPC) against equine herpesvirus- 1 (EHV- 1) in cell cultures, mice and horses. Antiviral Res. 19, 219–232.

Glorieux, S., Vandekerckhove, A.P., Goris, N., Yang, X.-Y., Steukers, L., Van de Walle, G.R., Croubels, S., Neyts, J., Nauwynck, H.J., 2012. Evaluation of the antiviral activity of (1′S,2′R)-9-[[1′,2′-bis(hydroxymethyl)cycloprop-1′-yl]methyl]guanine (A-5021) against equine herpesvirus type 1 in cell monolayers and equine nasal mucosal explants. Antiviral Res. 93, 234–238. https://doi.org/10.1016/j.antiviral.2011.11.016

Goodman, L.B., Loregian, A., Perkins, G.A., Nugent, J., Buckles, E.L., Mercorelli, B., Kydd, J.H., Palù, G., Smith, K.C., Osterrieder, N., Davis-Poynter, N., 2007. A Point Mutation in a Herpesvirus Polymerase Determines Neuropathogenicity. PLoS Pathog. 3, e160. https://doi.org/10.1371/journal.ppat.0030160

Gravina, H.D., Tafuri, N.F., Silva Júnior, A., Fietto, J.L.R., Oliveira, T.T., Diaz, M.A.N., Almeida, M.R., 2011. In vitro assessment of the antiviral potential of trans-cinnamic acid, quercetin and morin against equid herpesvirus 1. Res. Vet. Sci. 91, e158–e162. https://doi.org/10.1016/j.rvsc.2010.11.010

Halbrook, C.J., Pontious, C., Kovalenko, I., Lapienyte, L., Dreyer, S., Lee, H.-J., Thurston, G., Zhang, Y., Lazarus, J., Sajjakulnukit, P., Hong, H.S., Kremer, D.M., Nelson, B.S., Kemp, S., Zhang, L., Chang, D., Biankin, A., Shi, J., Frankel, T.L., Crawford, H.C., Morton, J.P., Pasca di Magliano, M., Lyssiotis, C.A., 2019. Macrophage-Released Pyrimidines Inhibit Gemcitabine Therapy in Pancreatic Cancer. Cell Metab. 29, 1390-1399.e6. https://doi.org/10.1016/j.cmet.2019.02.001

He, P.-F., Zhou, J.-D., Yao, D.-M., Ma, J.-C., Wen, X.-M., Zhang, Z.-H., Lian, X.-Y., Xu, Z.-J., Qian, J., Lin, J., 2017. Efficacy and safety of decitabine in treatment of elderly patients with acute myeloid leukemia: A systematic review and meta-analysis. Oncotarget 8, 41498–41507. https://doi.org/10.18632/oncotarget.17241

Henninger, R.W., Reed, S.M., Saville, W.J., Allen, G.P., Hass, G.F., Kohn, C.W., Sofaly, C., 2007. Outbreak of Neurologic Disease Caused by Equine Herpesvirus-1 at a University Equestrian Center. J. Vet. Intern. Med. 21, 157–165.

James, S., Larson, K., Acosta, E., Prichard, M., 2015. Helicase-Primase as a Target of New Therapies for Herpes Simplex Virus Infections. Clin. Pharmacol. Ther. 97, 66–78. https://doi.org/10.1002/cpt.3

Jiang, Y.-C., Feng, H., Lin, Y.-C., Guo, X.-R., 2016. New strategies against drug resistance to herpes simplex virus. Int. J. Oral Sci. 8, 1–6.

Jourdan, J., Bureau, R., Rochais, C., Dallemagne, P., 2020. Drug repositioning: a brief overview. J. Pharm. Pharmacol. jphp.13273. https://doi.org/10.1111/jphp.13273

Kit, S., Ichimura, H., De Clercq, E., 1987. Phosphorylation of nucleoside analogs by equine herpesvirus type 1 pyrimidine deoxyribonucleoside kinase. Antiviral Res. 7, 53–67. https://doi.org/10.1016/0166-3542(87)90039-8

Kleymann, G., Fischer, R., Betz, U.A.K., Hendrix, M., Bender, W., Schneider, U., Handke, G., Eckenberg, P., Hewlett, G., Pevzner, V., Baumeister, J., Weber, O., Henninger, K., Keldenich, J., Jensen, A., Kolb, J., Bach, U., Popp, A., Mäben, J., Frappa, I., Haebich, D., Lockhoff, O., Rübsamen-Waigmann, H., 2002. New helicase-primase inhibitors as drug candidates for the treatment of herpes simplex disease. Nat. Med. 8, 392–398. https://doi.org/10.1038/nm0402-392

Léon, A., Fortier, G., Fortier, C., Freymuth, F., Tapprest, J., Leclercq, R., Pronost, S., 2008. Detection of equine herpesviruses in aborted foetuses by consensus PCR. Vet. Microbiol. 126, 20–29. https://doi.org/10.1016/j.vetmic.2007.06.019

Liu, Z., Liu, S., Xie, Z., Blum, W., Perrotti, D., Paschka, P., Klisovic, R., Byrd, J., Chan, K.K., Marcucci, G., 2007. Characterization of in vitro and in vivo hypomethylating effects of decitabine in acute myeloid leukemia by a rapid, specific and sensitive LC-MS/MS method. Nucleic Acids Res. 35, e31–e31. https://doi.org/10.1093/nar/gkl1156

Lunn, D.P., Davis-Poynter, N., Flaminio, M.J.B.F., Horohov, D.W., Osterrieder, K., Pusterla, N., Townsend, H.G.G., 2009. Equine Herpesvirus-1 Consensus Statement. J. Vet. Intern. Med. 23, 450–461. https://doi.org/10.1111/j.1939-1676.2009.0304.x

Maggs, D.J., Clarke, H.E., 2004. In vitro efficacy of ganciclovir, cidofovir, penciclovir, foscarnet, idoxuridine, and acyclovir against feline herpesvirus type-1. Am. J. Vet. Res. 65, 399–403. https://doi.org/10.2460/ajvr.2004.65.399

Maxwell, L.K., 2017. Antiherpetic Drugs in Equine Medicine. Vet. Clin. North Am. Equine Pract. 33, 99–125. https://doi.org/10.1016/j.cveq.2016.12.002

Maxwell, L.K., Bentz, B.G., Bourne, D.W.A., Erkert, R.S., 2008. Pharmacokinetics of valacyclovir in the adult horse. J. Vet. Pharmacol. Ther. 31, 312–320. https://doi.org/10.1111/j.1365-2885.2008.00957.x

Momparler, R.L., 2005. Pharmacology of 5-Aza-2′-deoxycytidine (decitabine). Seminars in Hematology 42, S9–S16. https://doi.org/10.1053/j.seminhematol.2005.05.002Murray, M.J., Piero, F., Jeffrey, S.C., Davis, M.S., Furr, M.O., Dubovi, E.J., Mayo, J.A., 1998. Neonatal Equine Herpesvirus Type 1 Infection on a Thoroughbred Breeding Farm. J. Vet. Intern. Med. 12, 36–41. https://doi.org/10.1111/j.1939-1676.1998.tb00494.x

Nugent, J., Birch-Machin, I., Smith, K.C., Mumford, J.A., Swann, Z., Newton, J.R., Bowden, R.J., Allen, G.P., Davis-Poynter, N., 2006. Analysis of Equid Herpesvirus 1 Strain Variation Reveals a Point Mutation of the DNA Polymerase Strongly Associated with Neuropathogenic versus Nonneuropathogenic Disease Outbreaks. J. Virol. 80, 4047–4060. https://doi.org/10.1128/JVI.80.8.4047-4060.2006

Paillot, R., Sutton, G., Thieulent, C., Marcillaud-Pitel, C., Pronost, S., 2020. New EHV-1 variant identified. Vet. Rec. 186, 573.

Pan, T., Huang, B., Zhang, W., Gabos, S., Huang, D.Y., Devendran, V., 2013. Cytotoxicity assessment based on the AUC50 using multi-concentration time-dependent cellular response curves. Anal. Chim. Acta 764, 44–52. https://doi.org/10.1016/j.aca.2012.12.047

Piret, J., Goyette, N., Boivin, G., 2016. Novel Method Based on Real-Time Cell Analysis for Drug Susceptibility Testing of Herpes Simplex Virus and Human Cytomegalovirus. J. Clin. Microbiol. 54, 2120–2127. https://doi.org/10.1128/JCM.03274-15

Price, N.B., Prichard, M.N., 2011. Progress in the development of new therapies for herpesvirus infections. Curr. Opin. Virol. 1, 548–554. https://doi.org/10.1016/j.coviro.2011.10.015

Prichard, M.N., Shipman, C., 1990. A three-dimensional model to analyze drug-drug interactions. Antiviral Res. 14, 181–206.
Pronost, S., Léon, A., Legrand, L., Fortier, C., Miszczak, F., Freymuth, F., Fortier, G., 2010. Neuropathogenic and non-neuropathogenic variants of equine herpesvirus 1 in France. Vet. Microbiol. 145, 329–333. https://doi.org/10.1016/j.vetmic.2010.03.031
Rollinson, E.A., 1987. Comparative efficacy of three 2′-fluoropyrimidine nucleosides and 9-(1,3-dihydroxy-2-propoxymethyl)guanine (BW B759U) against pseudorabies and equine rhinopneumonitis virus infection in vitro and in laboratory animals. Antiviral Res. 7, 25–33. https://doi.org/10.1016/0166-3542(87)90036-2
Schmelz, K., Sattler, N., Wagner, M., Lübbert, M., Dörken, B., Tamm, I., 2005. Induction of gene expression by 5-Aza-2′-deoxycytidine in acute myeloid leukemia (AML) and myelodysplastic syndrome (MDS) but not epithelial cells by DNA-methylation-dependent and -independent mechanisms. Leukemia 19, 103–111. https://doi.org/10.1038/sj.leu.2403552
Shin, H., Kim, C., Cho, S., 2018. Gemcitabine and Nucleos(t)ide Synthesis Inhibitors Are Broad-Spectrum Antiviral Drugs that Activate Innate Immunity. Viruses 10, 1-11. https://doi.org/10.3390/v10040211
Smith, K.L., Allen, G.P., Branscum, A.J., Frank Cook, R., Vickers, M.L., Timoney, P.J., Balasuriya, U.B.R., 2010. The increased prevalence of neuropathogenic strains of EHV-1 in equine abortions. Vet. Microbiol. 141, 5–11. https://doi.org/10.1016/j.vetmic.2009.07.030
Stresemann, C., Lyko, F., 2008. Modes of action of the DNA methyltransferase inhibitors azacytidine and decitabine. Int. J. Cancer 123, 8–13. https://doi.org/10.1002/ijc.23607
Sullivan, V., Talarico, C.L., Stanat, S.C., Davis, M., Coen, D.M., Biron, K.K., 1992. A protein kinase homologue controls phosphorylation of ganciclovir in human cytomegalovirus-infected cells. Nature 358, 162–164.
Sutton, Garvey, Cullinane, Jourdan, Fortier, Moreau, Foursin, Gryspeerdt, Maisonnier, Marcillaud-Pitel, Legrand, Paillot, Pronost, 2019. Molecular Surveillance of EHV-1 Strains Circulating in France during and after the Major 2009 Outbreak in Normandy Involving Respiratory Infection, Neurological Disorder, and Abortion. Viruses 11, 916. https://doi.org/10.3390/v11100916
Tallmadge, R.L., Žygelytė, E., Van de Walle, G.R., Kristie, T.M., Felippe, M.J.B., 2018. Effect of a Histone Demethylase Inhibitor on Equine Herpesvirus-1 Activity In Vitro. Front. Vet. Sci. 5. https://doi.org/10.3389/fvets.2018.00034
Thieulent, C.J., Hue, E.S., Fortier, C.I., Dallemagne, P., Zientara, S., Munier-Lehmann, H., Hans, A., Fortier, G.D., Pitel, P.-H., Vidalain, P.-O., Pronost, S.L., 2019. Screening and evaluation of antiviral compounds against Equid alpha-herpesviruses using an impedance-based cellular assay. Virology 526, 105–116. https://doi.org/10.1016/j.virol.2018.10.013
Vissani, M.A., Thiry, E., Dal Pozzo, F., Barrandeguy, M., 2016. Antiviral agents against equid alphaherpesviruses: Current status and perspectives. Vet. J. 207, 38–44. https://doi.org/10.1016/j.tvjl.2015.06.010
Wang, J., Li, A., Jin, M., Zhang, F., Li, X., 2016. Dual-modality imaging demonstrates the enhanced antitumoral effect of herpes simplex virus-thymidine kinase/ganciclovir plus gemcitabine combination therapy on cholangiocarcinoma. Experimental and Therapeutic Medicine 12, 183–189. https://doi.org/10.3892/etm.2016.3294
Williams, S.L., Hartline, C.B., Kushner, N.L., Harden, E.A., Bidanset, D.J., Drach, J.C., Townsend, L.B., Underwood, M.R., Biron, K.K., Kern, E.R., 2003. In Vitro Activities of Benzimidazole D- and L-Ribonucleosides against Herpesviruses. Antimicrob.

Agents Chemother. 47, 2186–2192. https://doi.org/10.1128/AAC.47.7.2186-2192.2003
Zhang, J.-H., Chung, T.D.Y., Oldenburg Kevin R., 1999. A Simple Statistical Parameter for Use in Evaluation and Validation of High Throughput Screening Assays. J. Biomol. Screen. 4, 67–73.

Table 1: List of compounds presenting an antiviral effect against EHV-1 Kyd strain on E. Derm cell.

Data presented in this table are the mean (S.D.) of three independent experiments. EC_{50}: EC_{50}[a] half maximal effective concentration measured by impedance using Real-Time Cell Analysis (RTCA) system or EC_{50}[d] qPCR assay.

CC_{50}: CC_{50}[b] half maximal cytotoxic concentration measured by impedance using RTCA system or CC_{50}[e] CellTiter-Glo (CTG) method. "CC_{50} > 50" means that the compound did not show toxicity at the highest concentration tested (50 µM).

SI: Selectivity Index is the ration of CC_{50} obtained by RTCA to EC_{50} obtained by RTCA[c] or CC_{50} obtained by CTG to EC_{50} obtained by qPCR[f]. If "CC_{50} > 50", an arbitrary value of SI is calculated with 50 µM, but it is probably underestimated.

Bold compounds are the selected compounds in part 3.1 of results.

Table 2: Combination analysis of compounds against EHV-1 KyD strain on E. Derm cells.

[a]Mean volumes of synergy or antagonism are presented based on 95% confidence levels using MacSynergy II method.

Values determined using MacSynergy II software (Prichard and Shipman, 1990) via area under normalised curves (AUC_n) data from 0 to 96 hours post-infection using impedance measurement. Results are obtained from three independently experiments.

Figure 1: Correlation plot between EC_{50} values obtained by qPCR and RTCA from compounds presenting an antiviral effect against EHV-1 Kyd strain (n=16). Compounds with an EC_{50} well defined by qPCR or RTCA were included in this calculation (we excluded data with EC_{50} >50µM). Spearman correlation coefficient (R), P-value and confidence interval (Conf.Int.) are presented.

Figure 2: Antiviral effect of selected compounds against EHV-1 KyD strain on E. Derm cells. (**A**) Half maximal effective concentration (EC_{50}) measured at different times post-infection using impedance measurement (48 to 120 hpi). The dotted line represents the cut off EC_{50} value. (**B**) Dose-response curves obtained by qPCR (plain line) and RTCA (dotted line) for the eight retained compounds against EHV-1 at 48 hpi. Results are from three independent experiments.

Figure 3: Antiviral effect of the eight selected compounds on different cell lines and against different EHV-1 strains. (**A**) Susceptibility of EHV-1 KyD strain to the 8 selected antiviral compounds on three different cell lines: E Derm, EEK and RK13. Results are from three independent experiments ($^{*}p < 0.05$, $^{**}p < 0.01$). **B**) Susceptibility of three French isolates of EHV-1 to the 8 selected antiviral compounds. G_{2254} indicates a guanine in ORF30 position 2254 (Aspartic Acid (D_{752}) in position 752 of the protein), A_{2254} indicates an adenine (Asparagine (N_{752})) and C_{2254} indicates a cytidine (Histidine (H_{752})). EC_{50} values were obtained by qPCR on EEK cells. Results are from three independent experiments ($^{*}p < 0.05$, $^{**}p < 0.01$).

Figure 4: Synergistic inhibition of EHV-1 KyD strain replication in E. Derm cells by combination of valganciclovir/decitabine. (**A**) Analysis of interaction of VGCV and DTB using impedance measurement with MacSynergy II software. Peaks of statistically significant (95% confidence level) synergy are shown above the plane in colours from grey to blue, with dark blue indicating a strong synergy. The volume of synergy for this interaction is 63.24, which is interpreted as moderate synergy. Results are obtained from three independently experiments performed using impedance measurement. (**B**) Isobologram analysis of the interaction of VGCV and DTB using impedance measurement. The diagonally dotted line in red represents additivity. Values below and above this line are interpreted as synergy or antagonist, respectively. The ADA value for this interaction is -0.30 ($p < 0.001$) which is interpreted as synergy. Results are obtained from three independently experiments performed using impedance measurement. (**C**) Median-effect analysis table representing the interaction of VGCV and DTB at 1:1 ratio using qPCR assay. Combination Index (CI) was calculated using the Chou and Talalay equation (Chou & Talalay, 1984). CI < 1, CI = 1 and CI > 1 indicate synergism, additive and antagonism, respectively. The weighted CI is calculated as follows: $CI_{wt} = (CI_{50} + 2CI_{75} + 3\ CI_{90} + 4CI_{95})/10$. The CI_{wt} value for this interaction is 0.20

which is interpreted as synergy. Results are obtained from three independently experiments performed using qPCR assay.

Figure 5: Effect of decitabine (5µM) pre-infection or post-infection treatment on E. Derm cells infected with EHV-1 KyD strain measured at 48 hpi by (**A**) impedance measurement and (**B**) viral genome copies number quantitation. Results are from three independent experiments ($^{***}p < 0.001$).

Figure 6: Effect of the DNA methyltransferase inhibitor, RG108 and decitabine on E. Derm cells infected with EHV-1 KyD strain measured at 48 hpi by (**A**) impedance measurement and (**B**) viral genome copies number quantitation. Results are from three independent experiments.

Figure 7: Effect of deoxycytidine (dC) on the antiviral effect of decitabine (DTB) against EHV-1. (**A**) Viral genome copies number produces in the cell culture supernatant at 48 hpi in absence (0 µM) or presence (1.6 and 6.4 µM) of DTB with 100 µM of cytidine, uridine, adenosine, guanosine and dC. Results are obtained from five independent experiments ($^{*}p < 0.05$). (**B**) Microscopic observation at 48 hpi of E. Derm cells infected or not by EHV-1 KyD strain in presence of DTB (6.4 µM) with or without dC (100 µM) treatment. (**C**) Impedance measurement at 48 hpi of E. Derm cells infected with EHV-1 KyD strain and treated with increased concentrations of DTB in the presence of the indicated concentration of dC. Results are from three independent experiments.

Supplementary Table 1: Library of selected compounds for their antiviral effects on different families of viruses.

Supplementary Figure 1: Screening of 2,891 chemical compounds in order to identify EHV-1 inhibitors. CERMN library was screened at 10 µM, Prestwick® Chemical library was

screened at 10 µg/mL and in-house antiviral library were screened at 4 concentrations (0.4, 2, 10 and 50 µM). For each library, the percentage of area under normalised Cell Index (CI) from 0 to 96 hpi of treated cells compared to mock-treated cells (%AUC) and the increase of the time required for the CI to decrease by 50% after virus infection (CIT_{50}) of treated cells compared to mock-treated cells (ΔCIT_{50}) were reported graphically. Compounds allowing an increase of the AUCn by 25% (red line) and a delay of the CIT_{50} by >8 h (red line) as compared to non-treated cells were identified as potential antiviral compounds against EHV-1.

Supplementary Figure 2: Cytotoxicity assay of combinations on equine dermal cells. Toxicity evaluation is measured by luminescence assays using CellTiter Glo® kit. Histogram represents the percentage viability of E. Derm cells treated with the highest concentration of compounds compared to mock-treated cells. Each data corresponds to mean ± SD of three independent experiments.

Figure 1

Click here to access/download;Figure;Figure 1.tif

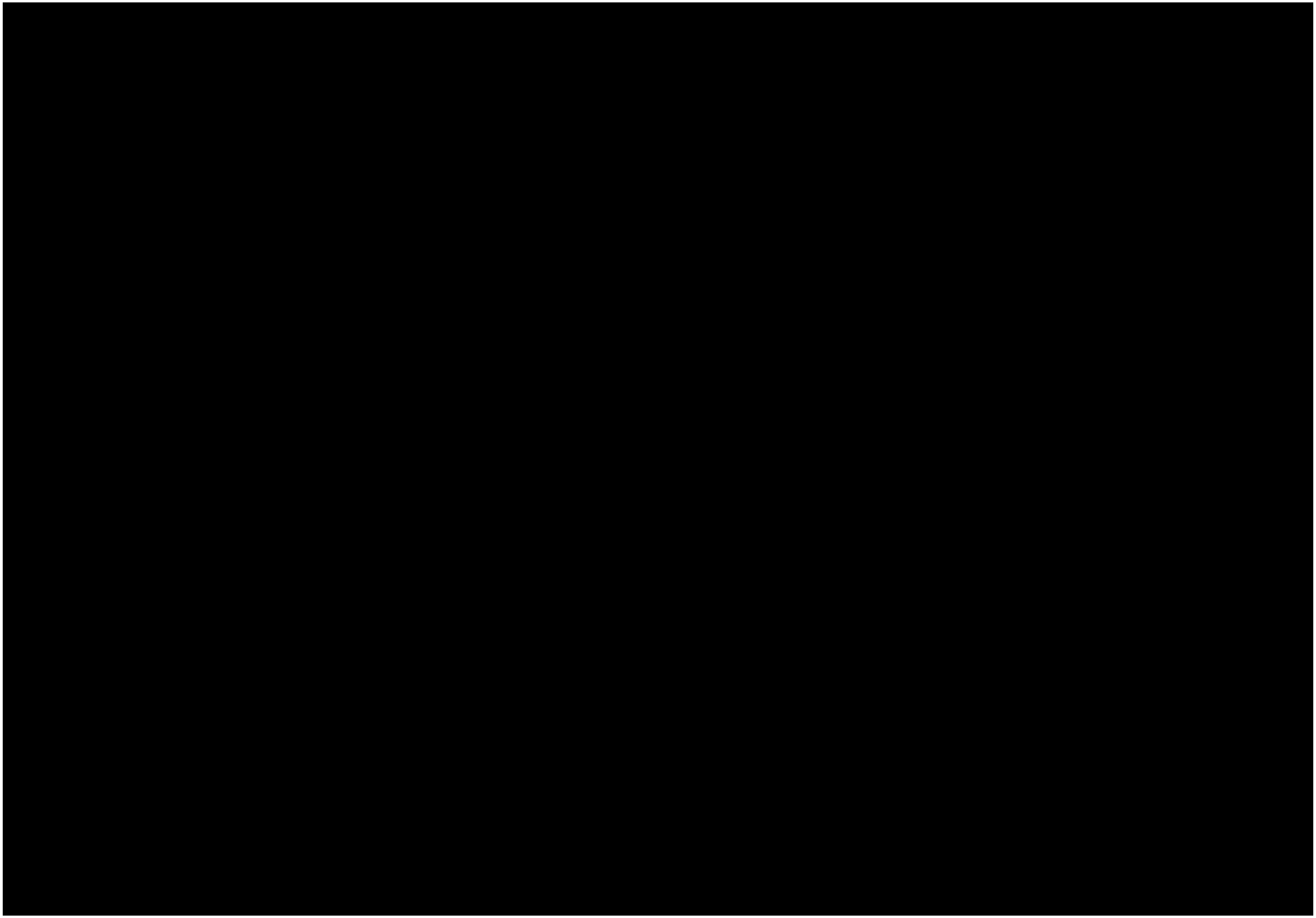

Click here to access/download;Figure;Figure 2.tif

[redacted] [redacted]

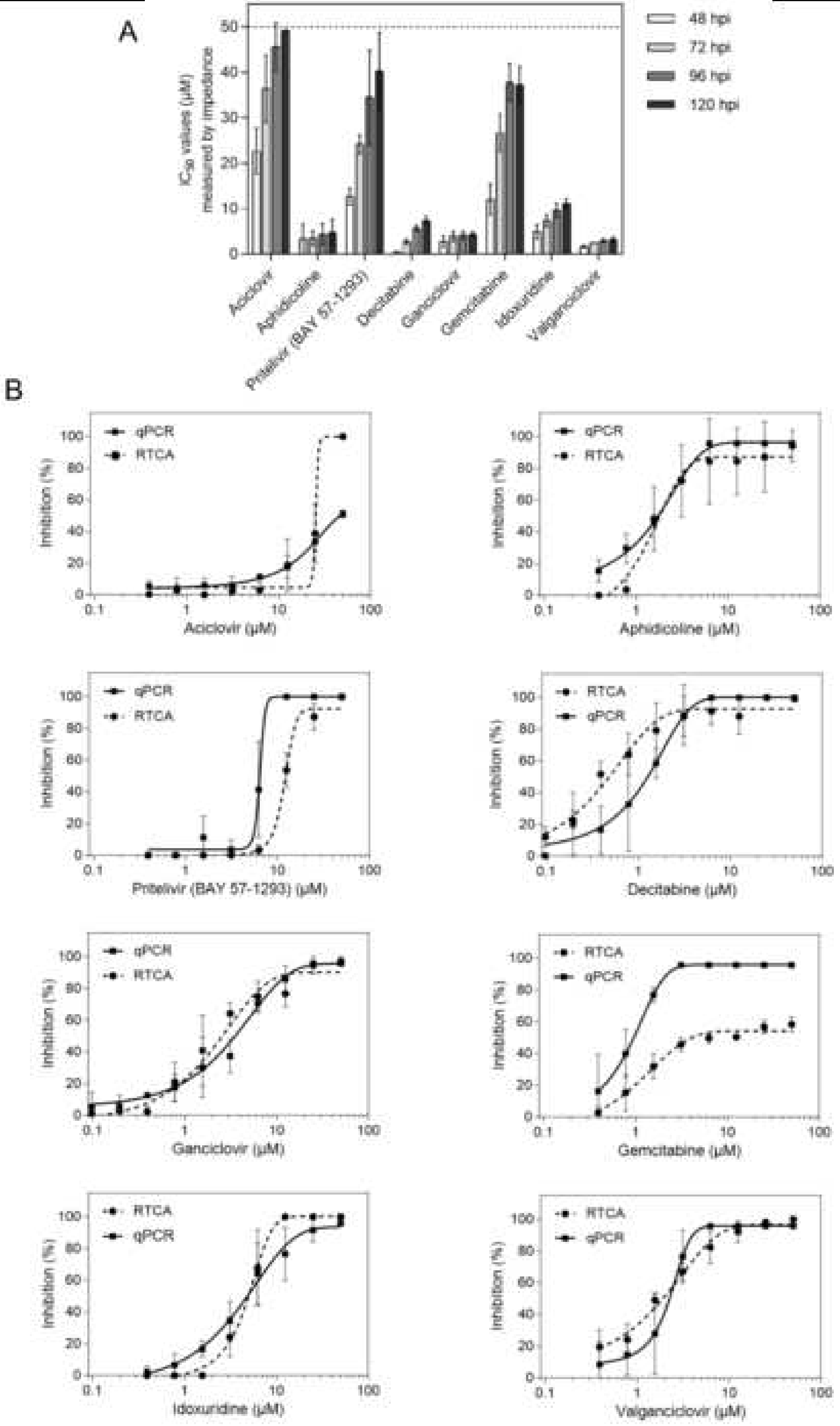

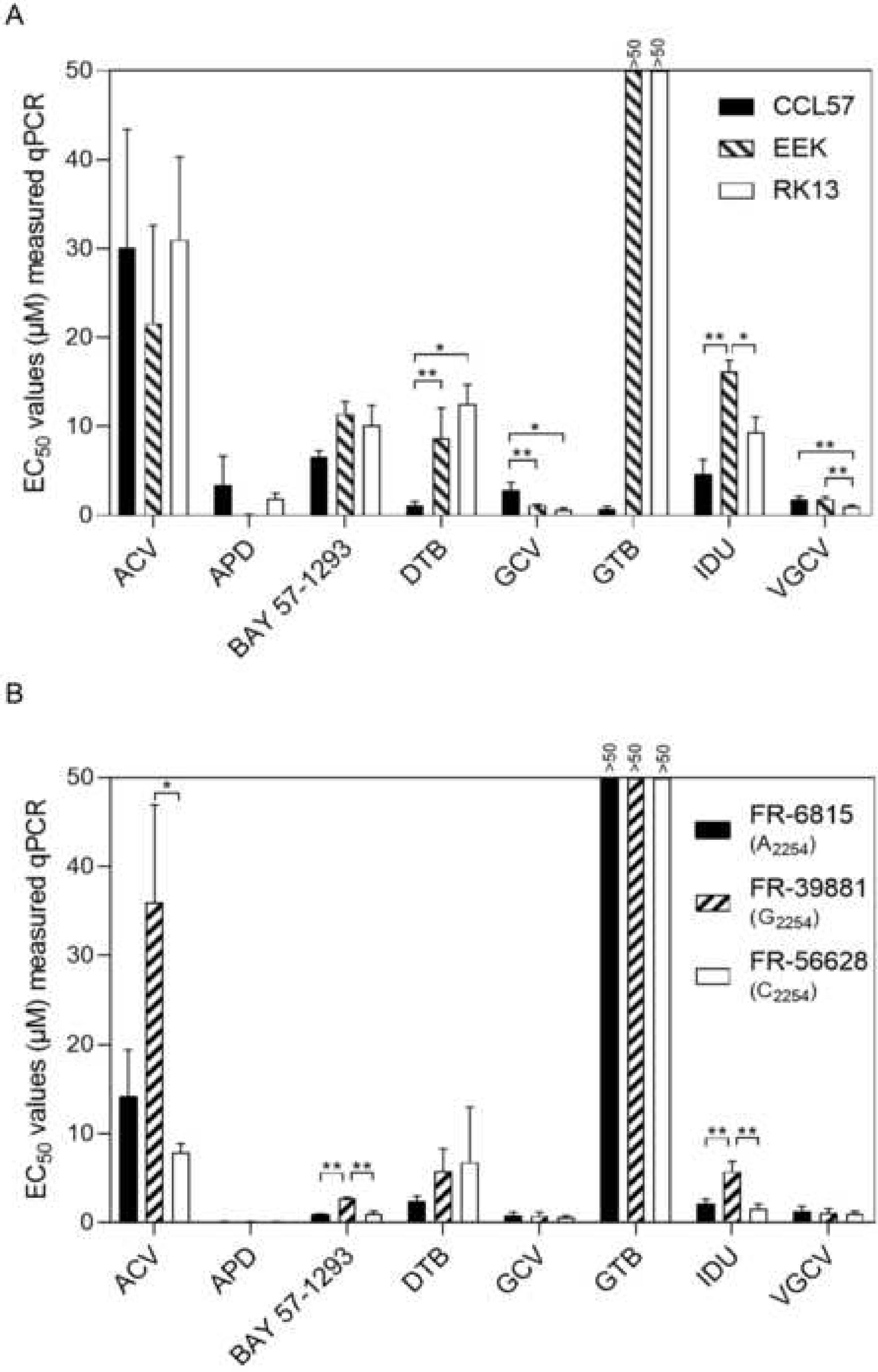
A
EC50 values (µM) measured qPCR
50
40
30
20
10
0
>50
>50
CCL57
EEK
RK13
*
**
*
**
**
*
**
**
ACV
APD
BAY 57-1293
DTB
GCV
GTB
IDU
VGCV
B
EC50 values (µM) measured qPCR
50
40
30
20
10
0
>50
>50
>50
FR-6815
(A2254)
FR-39881
(G2254)
FR-56628
(C2254)
*
**
**
**
**
ACV
APD
BAY 57-1293
DTB
GCV
GTB
IDU
VGCV

Figure 4 Click here to access/download;Figure;Figure 4.tif

A

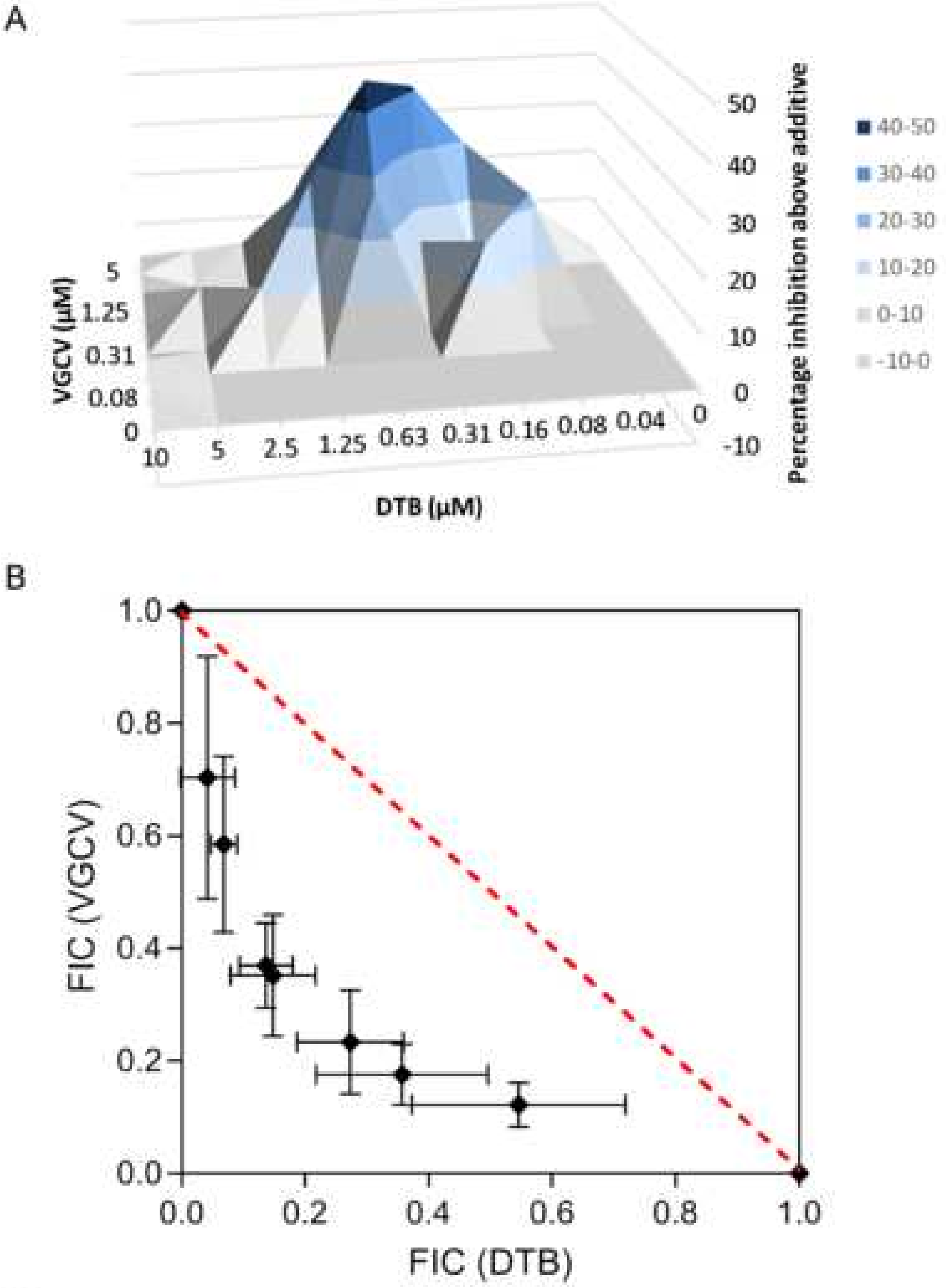

C

	CI values extrapolated at % of virus inhibition				
		CI_{50}	CI_{75}	CI_{90}	CI_{95}
VGCV + DTB (1:1)	Experiment 1	0.44	0.33	0.24	0.20
	Experiment 2	0.31	0.21	0.16	0.13
	Experiment 3	0.33	0.21	0.13	0.10
	Mean (S.D.)	0.36 (0.07)	0.25 (0.07)	0.18 (0.06)	0.14 (0.05)
	CI_{wt}	0.20			
Drug combinatory effect		Synergism			

Figure 5

Click here to access/download;Figure;Figure 5.tif

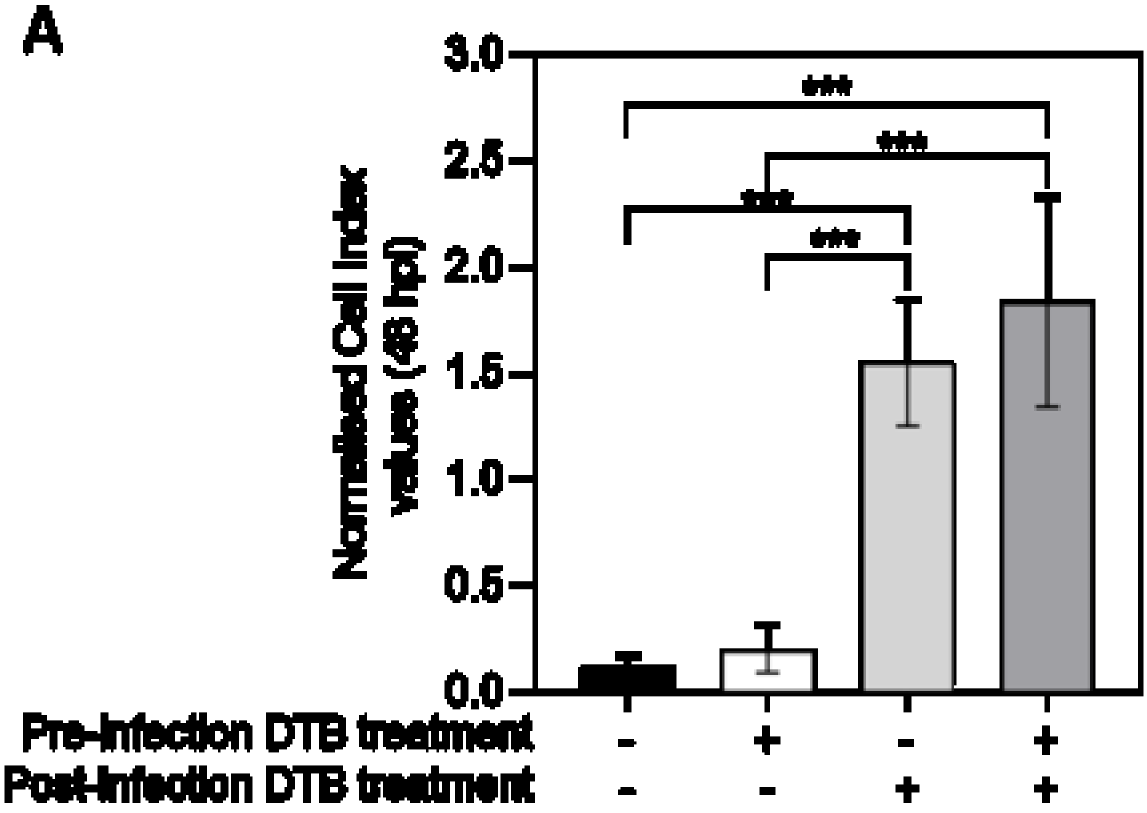

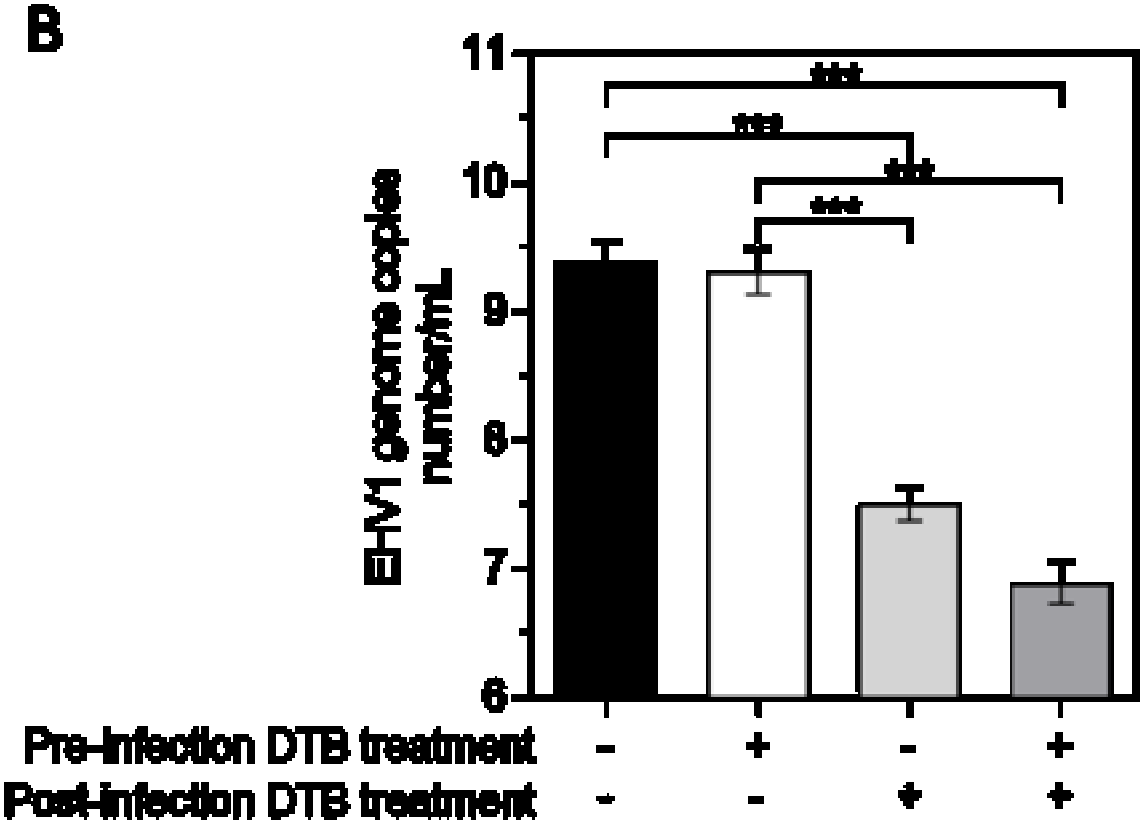

Figure 6 Click here to access/download;Figure;Figure 6.tif

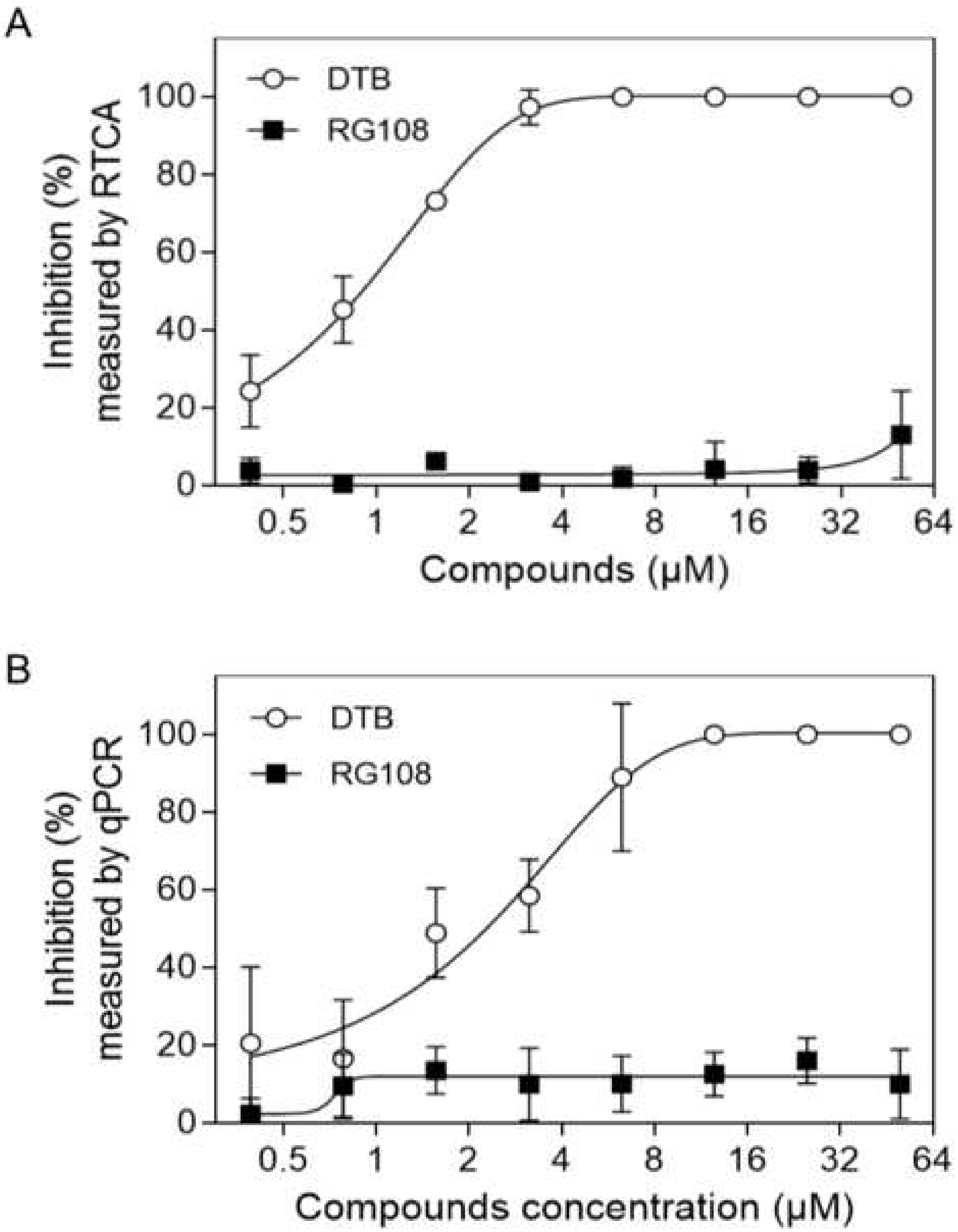

Figure 7

Click here to access/download;Figure;Figure 7.tif

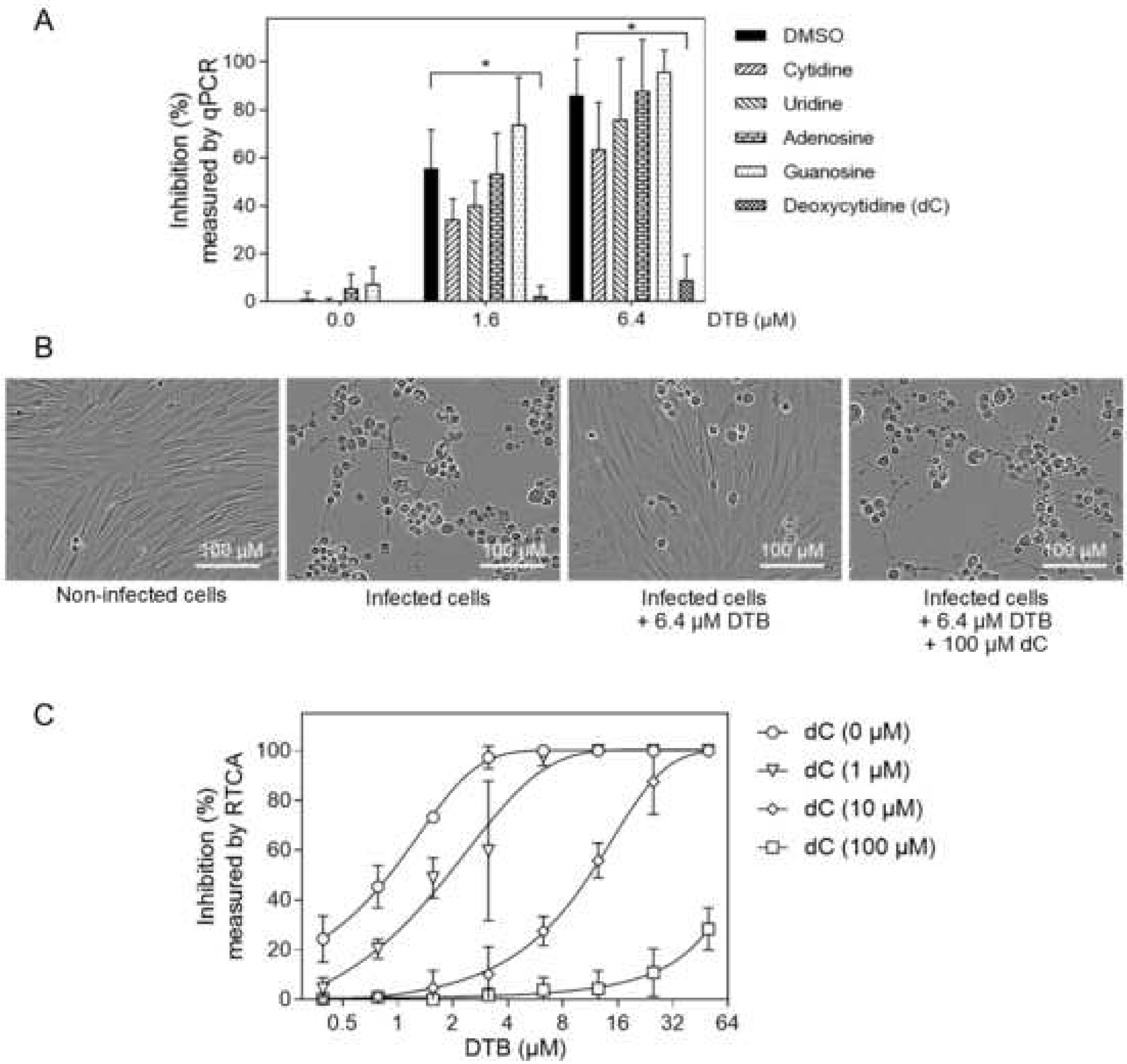

Table 1

Click here to access/download
Table
Table 1.xlsx

Table 2

Click here to access/download

Table

Table 2.xlsx

Supplementary Table 1

Click here to access/download
Supplementary Material
Supplementary Table 1.xlsx

Supplementary Figure 1

Click here to access/download
Supplementary Material
Supplementary Figure 1.tif

Supplementary Figure 2

Click here to access/download
Supplementary Material
Supplementary Figure 2.tif

Click here to access/download
Data in Brief
Data in Brief - Submission.zip
